# Risk of *Plasmodium vivax* parasitaemia after *Plasmodium falciparum* infection: a systematic review and meta-analysis

**DOI:** 10.1016/S1473-3099(18)30596-6

**Published:** 2019-01

**Authors:** Robert J Commons, Julie A Simpson, Kamala Thriemer, Mohammad S Hossain, Nicholas M Douglas, Georgina S Humphreys, Carol H Sibley, Philippe J Guerin, Ric N Price

**Affiliations:** a Global Health Division, Menzies School of Health Research and Charles Darwin University, Darwin, NT, Australia; b WorldWide Antimalarial Resistance Network, Oxford, UK; c Centre for Epidemiology and Biostatistics, Melbourne School of Population and Global Health, The University of Melbourne, Melbourne, VIC, Australia; d International Centre for Diarrhoeal Disease Research, Dhaka, Bangladesh; e Centre for Tropical Medicine and Global Health, Nuffield Department of Clinical Medicine, University of Oxford, Oxford, UK; f Department of Genome Sciences, University of Washington, Seattle, WA, USA

## Abstract

**Background:**

A 14-day course of primaquine is used for radical cure of *Plasmodium vivax* and *Plasmodium ovale* malaria only. We quantified the risk of *P vivax* parasitaemia after treatment of *Plasmodium falciparum* with commonly used antimalarial drugs to assess the potential benefits of radical cure for all patients with uncomplicated malaria in co-endemic regions.

**Methods:**

In this systematic review and meta-analysis, we searched MEDLINE, Embase, Web of Science, and the Cochrane Database of Systematic Reviews for prospective clinical studies in any language, published between Jan 1, 1960, and Jan 5, 2018, assessing drug efficacy in patients with uncomplicated *P falciparum* malaria in countries co-endemic for *P vivax*. Studies were included if the presence or absence of *P vivax* parasitaemia was recorded after treatment. The primary outcome was the risk of *P vivax* parasitaemia between day 7 and day 42 after initiation of antimalarial treatment for *P falciparum*, with the pooled risk calculated by random-effects meta-analysis. We compared the risk of *P vivax* parasitaemia after treatment with different artemisinin-based combination therapies (ACTs). This study is registered with PROSPERO, number CRD42017064838.

**Findings:**

153 of 891 screened studies were included in the analysis, including 31 262 patients from 323 site-specific treatment groups: 130 (85%) studies were from the Asia-Pacific region, 16 (10%) from the Americas, and seven (5%) from Africa. The risk of *P vivax* parasitaemia by day 42 was 5·6% (95% CI 4·0–7·4; *I*^*2*^=92·0%; 117 estimates). The risk of *P vivax* parasitaemia was 6·5% (95% CI 4·6–8·6) in regions of short relapse periodicity compared with 1·9% (0·4–4·0) in regions of long periodicity, and was greater after treatment with a more rapidly eliminated ACT: 15·3% (5·1–29·3) for artemether-lumefantrine compared with 4·5% (1·2–9·3) for dihydroartemisinin-piperaquine and 5·2% (2·9–7·9) for artesunate-mefloquine. Recurrent parasitaemia was delayed in patients treated with ACTs containing mefloquine or piperaquine compared with artemether-lumefantrine, but by day 63 the risk of vivax parasitaemia was more than 15% for all ACTs assessed.

**Interpretation:**

Our findings show a high risk of vivax parasitaemia after treatment of falciparum malaria, particularly in areas with short relapse periodicity and after rapidly eliminated treatment. In co-endemic regions, universal radical cure for all patients with uncomplicated malaria has the potential to substantially reduce recurrent malaria.

**Funding:**

Australian National Health and Medical Research Council, Royal Australasian College of Physicians, Wellcome Trust, and Bill & Melinda Gates Foundation.

## Introduction

In 2016, more than 200 million cases of malaria were attributable to *Plasmodium falciparum*,^1^ of which approximately 8% occurred in countries co-endemic for *P falciparum* and *Plasmodium vivax*. In Thailand, the risk of *P vivax* parasitaemia after *P falciparum* infection is substantially higher than would be expected from background entomological inoculation rates and almost 1·5 times greater than the risk of recurrent *P falciparum*.^2^, ^3^ Hence, in many co-endemic malarious areas, the most commonly transmitted plasmodium parasite after falciparum malaria is likely to be *P vivax*.^4^ Recurrent *P vivax* parasitaemia is associated with substantial morbidity including a cumulative risk of severe anaemia.^5^ Furthermore, since *P vivax* sexual stages (gametocytes) are commonly present with recurrent asexual parasitaemia, there is an increased risk of transmission of *P vivax* to the mosquito vector.^4^, ^6^

In some locations, declining chloroquine efficacy has led national malaria control programmes to adopt a universal policy of artemisinin-based combination therapy (ACT) for blood schizontocidal treatment of both *P falciparum* and *P vivax.*^7^ However, schizontocidal treatment has no activity against *P vivax* hypnozoites. At the time of writing, primaquine is the only widely available hypnozoitocidal treatment, which is required to achieve the radical cure of vivax malaria.^8^ The high risk of vivax parasitaemia after falciparum infection raises the possibility that primaquine radical cure should be prescribed to patients presenting with falciparum infection, as well as those with vivax infection.

**Research in context**

**Evidence before this study**

We searched MEDLINE, Web of Science, Embase, and the Cochrane Database of Systematic Reviews with the terms “falciparum”, “vivax”, and “recurrence”, to identify all articles in any language published between Jan 1, 1960, and Jan 5, 2018, assessing the risk of *Plasmodium vivax* parasitaemia after *Plasmodium falciparum* infection. A pooled analysis of individual patient data from Thailand found that there was a 32% cumulative risk of *P vivax* parasitaemia by day 63 after treatment for *P falciparum*, greater than would be expected on the basis of local background entomological inoculation rates. A review of 19 studies in which patients with *P falciparum* were treated with artemether-lumefantrine identified a high risk of subsequent *P vivax* parasitaemia. To our knowledge, no systematic reviews and pooled analyses have assessed the risk of *P vivax* parasitaemia after treatment of *P falciparum* infection outside of Thailand.

**Added value of this study**

In this systematic review, we identified 31 262 patients from 153 studies across 21 countries to quantify the global risk of *P vivax* parasitaemia after treatment of *P falciparum* malaria. Our meta-analysis provides a comprehensive comparison of the risk of *P vivax* parasitaemia after treatment with different artemisinin-based combination therapies (ACTs). Our findings show a high risk of *P vivax* parasitaemia after *P falciparum* infection in co-endemic regions. The risk of *P vivax* parasitaemia is greatest after rapidly eliminated antimalarial regimens and in regions where *P vivax* has a short relapse periodicity. After day 42, the risk of *P vivax* is high after treatment with all of the major ACTs.

**Implications of all the available evidence**

In co-endemic regions, universal radical cure, such as an ACT plus primaquine or tafenoquine, in all patients with uncomplicated malaria has the potential to prevent subsequent *P vivax* parasitaemia and enhance malaria elimination efforts.

To determine the potential benefits of a universal policy of a 14-day regimen of primaquine radical cure, we quantified the risk of *P vivax* parasitaemia after treatment of *P falciparum* with commonly used antimalarial treatments in a range of co-endemic regions.

## Methods

### Search strategy and selection criteria

We searched MEDLINE, Embase, Web of Science, and the Cochrane Database of Systematic Reviews for prospective studies of drug efficacy in patients with uncomplicated *P falciparum* malaria residing in areas co-endemic for *P falciparum* and *P vivax*. Co-endemic countries were defined as countries where indigenous *P falciparum* and *P vivax* cases were reported or suspected in 2016 (appendix p 2).^1^ In a sensitivity analysis, studies from countries without recorded cases in 2016, but considered to be potentially co-endemic with unstable transmission,^9^ were also screened for the presence of vivax parasitaemia after falciparum infection.

Studies published between Jan 1, 1960, and Jan 5, 2018, in any language were included in the analysis if they explicitly reported the presence or absence of recurrent parasitaemia with both *P vivax* and *P falciparum* after treatment for falciparum infection. Further details of the search strategy including search terms are provided in the appendix (p 3). Studies were excluded if the observation period was less than 28 days, if active follow-up was not undertaken, if the numbers of recurrent *P vivax* and *P falciparum* parasitaemias observed could not be extracted, if only pregnant women or patients with severe malaria were enrolled, or if the full-text manuscript was unavailable. To reduce reporting bias, retrospective studies were not included. Studies were identified (RJC and GSH) and data extracted (RJC and MSH) by two authors, with discrepancies resolved following discussion with a third author (RNP). Findings are reported according to the Preferred Reporting Items for Systematic Reviews and Meta-Analyses guidelines (checklist in the appendix pp 4–6).^10^

### Data extraction and definitions

Data were extracted for individual treatment sites and treatment groups (referred to as records). When data for *P vivax* parasitaemia were aggregated across treatment groups or sites in a publication, the aggregated data for that record were assigned to the largest enrolment site, or to the study country if enrolment site data were not reported. Extracted data included details of the study, site, and treatment; efficacy at day 28, 42, and 63; patient characteristics; and details of *P falciparum* parasitaemia at baseline, including the presence of mixed infection, baseline parasitaemia, and presence of gametocytaemia.

Study sites were categorised into long or short *P vivax* relapse periodicity according to their geographical location and relapse criteria defined by the Malaria Atlas Project.^11^ Short relapse periodicity was defined as a median time to relapse of 47 days or less. Study year was defined as the year that study enrolment was completed, or if not available, the year of publication. The elimination half-life of the antimalarial used was categorised as rapid (<1 day), intermediate (1–7 days), or slow (>7 days), and for combination therapies it was based on the longest acting partner drug (appendix pp 7–9).^2^ Subgroup analyses were undertaken for the three most frequently recommended ACTs: artemether-lumefantrine, dihydroartemisinin-piperaquine, and artesunate-mefloquine. The prevalence of gametocytaemia at baseline was defined as the percentage of patients with *P falciparum* gametocytes present at enrolment, or if this was not recorded, within the first 3 days of treatment. When only the haematocrit was available, it was converted to haemoglobin using the formula: haemoglobin=(haematocrit–5·62)/2·6.^12^

### Data analysis

The primary outcome was risk of *P vivax* parasitaemia before day 42 following initiation of treatment for *P falciparum* infection, defined as any *P vivax* parasitaemia detectable by microscopy between day 7 and day 42. Evaluable patients included patients with subsequent *P vivax* parasitaemia, *P falciparum* parasitaemia, treatment failure before day 7 (defined as failing to clear parasitaemia before day 7), or patients followed until day 42 with adequate clinical and parasitological response. Risk is the percentage of patients with *P vivax* parasitaemia out of the evaluable patients. Secondary outcomes were the risk of *P vivax* malaria before day 28 or 63 and recurrent parasitaemia due to any species before day 28, 42, or 63. If not stated explicitly, *P vivax* and *P falciparum* parasitaemia were assumed to have occurred in separate patients.

Potential bias relating to individual studies was assessed using a tool developed by the Joanna Briggs Institute (appendix pp 10–13).^13^

The pooled risk of recurrent *P vivax* parasitaemia, any Plasmodium parasitaemia, and proportion of recurrent parasitaemia related to *P vivax* at day 28, 42, and 63 were estimated using random-effects meta-analysis with proportions pooled using the Freeman-Tukey double arcsine transformation without adjustment to observed values and with exact methods to calculate confidence intervals.^14^ This method was used to enable proportions equal to zero to be included in the analysis. Bias relating to study effect was assessed with funnel plots and Egger's asymmetry test.^15^ Between-study heterogeneity was quantified by the *I*^2^ statistic and assessed with random effects meta-regression to investigate the association between the study-specific risks and baseline characteristics (age, sex, drug elimination half-life, geographical region, relapse periodicity, presence of mixed infection, baseline parasitaemia, baseline haemoglobin, and baseline gametocyte prevalence). Pooled risks of recurrent *P vivax* parasitaemia after treatment with artemether-lumefantrine, dihydroartemisinin-piperaquine, and artesunate-mefloquine were compared, with random effects meta-regression used to investigate these risks further. Analyses were done with Stata version 15 and R version 3.4.0 using the metafor package.^16^

The study protocol is registered with PROSPERO, number CRD42017064838.

### Role of the funding source

The funder of the study had no role in study design, data collection, data analysis, data interpretation, or writing of the report. The corresponding author had full access to all the data in the study and had final responsibility for the decision to submit for publication.

## Results

**Figure 1:**
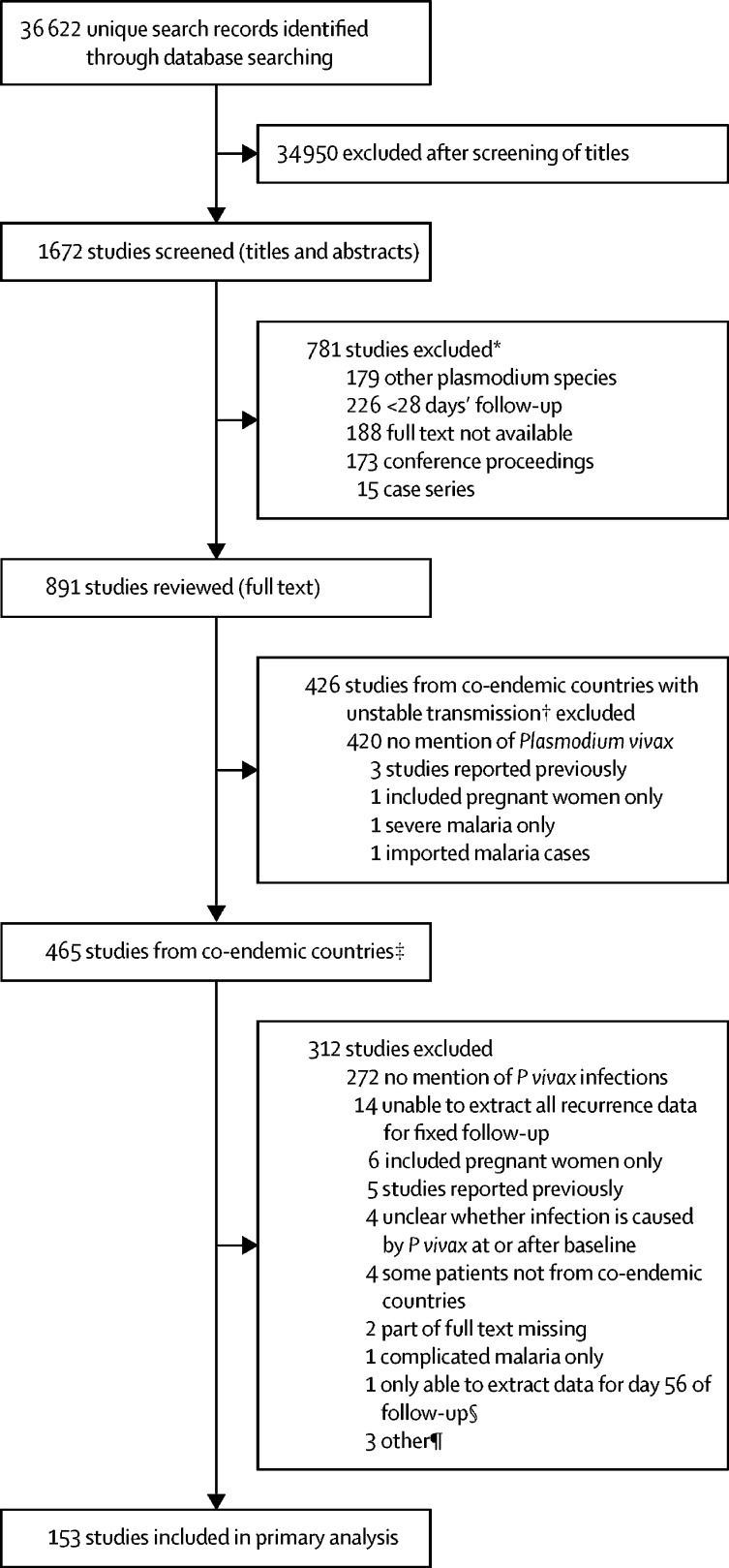
Study selection * Citations and reasons for exclusion are given in the appendix (pp 21, 60–89). †As defined by the Malaria Atlas Project.^9^ ‡Countries that reported or were suspected to have cases of indigenous *Plasmodium falciparum* and *Plasmodium vivax* in 2016 according to WHO's World Malaria Report 2017.^1^ §Outcomes for two treatment groups in the study by Li and colleagues^17^ were not reported and these groups were also excluded. ¶Other reasons for exclusion are listed in the appendix (p 21).

After screening the titles and abstracts of 1672 identified studies published between Jan 1, 1960, and Jan 5, 2018, full texts of 891 (53%) potentially relevant studies were reviewed. None of 426 studies from areas of unstable *P vivax* transmission met the inclusion criteria, mostly because they did not document *P vivax* parasitaemia. 153 (33%) of 465 studies from areas of stable indigenous transmission were included in the analysis (figure 1, appendix pp 14–36). Of the 153 studies, 130 (85%) were from the Asia-Pacific region, 16 (10%) were from the Americas, and seven (5%) were from Africa (table 1, appendix p 37). Data for outcomes could be extracted from 106 studies for day 28, 58 studies for day 42, and 12 studies for day 63.

**Table 1:** Baseline study data, site and treatment data, and patient variables

		**n (%) or median (IQR); range**	**Details**
**Study-level data (153 studies)**			
Randomised		91 (59%)	19 542 patients
Mixed infections included		21 (14%)	556 patients
Region			
	Asia-Pacific	130 (85%)	28 321 patients
	The Americas	16 (10%)	1973 patients
	Africa	7 (5%)	968 patients
Regional relapse periodicity			
	Long	31 (20%)	6930 patients
	Short	122 (80%)	24 332 patients
Result available for day of follow-up			
	Day 28	106 (69%)	16 207 patients
	Day 42	58 (38%)	13 797 patients
	Day 63	12 (8%)	4383 patients
**Site and treatment group level data (323 records)**			
Treatment groups by half-life*			
	Rapid, <24 h	71 (22%)	2566 patients
	Intermediate, 1–7 days	55 (17%)	6130 patients
	Slow, >7 days	192 (59%)	21 844 patients
Major treatment groups			
	Artemisinin monotherapy	41 (13%)	1326 patients
	Artemether-lumefantrine	32 (10%)	4016 patients
	Dihydroartemisinin-piperaquine	24 (7%)	3104 patients
	Artesunate-mefloquine	38 (12%)	6370 patients
	Quinine	3 (1%)	124 patients
	Chloroquine	13 (4%)	774 patients
**Baseline patient data across site and treatment groups (323 records)**			
Age, years		24·0 (20·0–26·8); 2·9–38·4	Available from 250 records†
Female, %		25·8% (0·0–40·0); 0·0–100·0%	Available from 276 records
Baseline parasitaemia, per μL		12 624 (7278–22 044); 518–68 178	Available from 290 records‡
Baseline gametocyte presence, %		14·4% (7·8–25·5); 0·0–52·0%	Available from 104 records
Baseline haemoglobin, g/dL		11·5 (10·9–12·0); 7·8–14·3	Available from 179 records§

n=number of studies or records.

* Treatment could be categorised by drug elimination half-life for 318 of the 323 records.

† Age derived from mean age for 197 records and median age for 53 records.

‡ Baseline parasitaemia derived from geometric mean for 268 records and median for 22 records.

§ Baseline haemoglobin derived from mean baseline haemoglobin for 72 records, median baseline haemoglobin for 22 records, mean baseline haematocrit for 72 records, and median baseline haematocrit for 13 records.

The risk of parasitaemia was available for 323 records from the 153 studies, including 31 262 patients from 21 countries. Of these records, 277 (86%) were from individual sites with a single treatment group and 46 (14%) were from aggregated sites or treatment groups, or both. 32 records included treatment with artemether-lumefantrine, 24 with dihydroartemisinin-piperaquine, and 38 with artesunate-mefloquine (table 1).

There was substantial heterogeneity between study populations (table 1). Information on age was available from 250 (77%) of 323 records, with the mean or median (depending on the value reported in the record) age ranging from 2·9 years to 38·4 years. Mean or median baseline parasitaemia ranged from 518 to 68 178 parasites per μL (290 records), the prevalence of *P falciparum* gametocytes at baseline ranged from 0% to 52% (104 records), and the mean or median baseline haemoglobin ranged from 7·8 g/dL to 14·3 g/dL (179 records).

**Figure 2:**
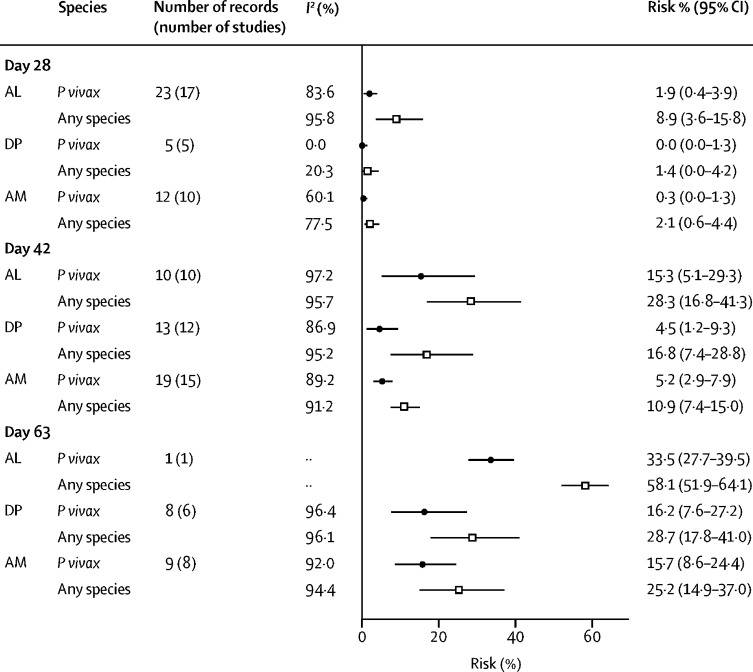
Risk of *Plasmodium vivax* parasitaemia or any parasitaemia after *Plasmodium falciparum* infection by artemisinin-based combination therapy and day of follow-up Risk is the percentage of patients with *P vivax* parasitaemia or any parasitaemia. AL=artemether-lumefantrine. DP=dihydroartemisinin-piperaquine. AM=artesunate-mefloquine.

The overall risk of any recurrent parasitaemia was 15·3% (95% CI 12·4–18·4; *I*^*2*^=95·4%; 213 estimates) by day 28, 18·4% (14·9–22·1; *I*^*2*^=95·8%; 117 estimates) by day 42, and 35·7% (29·0–42·7; *I*^*2*^=94·8%; 30 estimates) by day 63 (table 2, appendix pp 38–39). The pooled estimates of the percentage of recurrent parasitaemia due to *P vivax* were 37·4% (95% CI 30·4–44·6) by day 28, 37·1% (28·2–46·2) by day 42, and 68·6% (60·6–76·1) by day 63. By day 42, the risk of any parasitaemia was 28·3% (95% CI 16·8–41·3) after treatment with artemether-lumefantrine, 16·8% (7·4–28·8) after dihydroartemisinin-piperaquine, and 10·9% (7·4–15·0) after artesunate-mefloquine (table 2, figure 2).

**Table 2:** Pooled risk of parasitaemia at day 42 after *P falciparum* infection

		**Number of records (number of studies)**	***Plasmodium vivax* parasitaemia**		Any parasitaemia*	
			*I*^2^ (%)	Pooled percentage (95% CI)	*I^2^***(%)**	Pooled percentage (95% CI)
Overall		117 (58)	92·0%	5·6% (4·0–7·4)	95·8%	18·4% (14·9–22·1)
Relapse periodicity						
	Long	17 (10)	86·1%	1·9% (0·4–4·0)	98·2%	15·0% (5·3–28·2)
	Short	100 (48)	91·5%	6·5% (4·6–8·6)	94·6%	19·0% (15·4–22·8)
Region						
	Africa	2 (2)	95·2%	2·0% (0·0–14·6)	0	9·1% (6·5–12·0)
	The Americas	6 (5)	80·9%	6·2% (1·7–12·7)	94·6%	13·5% (2·6–30·2)
	Asia-Pacific	109 (51)	92·2%	5·7% (4·0–7·6)	95·8%	18·9% (15·2–22·9)
Country-specific analyses						
	Thailand	31 (16)	91·3%	5·5% (2·9–8·8)	95·5%	15·0% (9·5–21·6)
	Indonesia	2 (2)	91·0%	4·9% (0·0–27·4)	98·2%	32·0% (0·0–94·6)
	India	4 (2)	0	0·1% (0·0–0·5)	94·5%	22·9% (8·0–42·4)
	Brazil	5 (4)	75·2%	4·6% (0·7–10·8)	79·5%	7·4% (2·0–15·5)
	Myanmar	12 (3)	82·2%	9·1% (6·0–12·8)	87·9%	18·2% (13·1–23·9)
	Laos	10 (5)	57·1%	1·9% (0·4–4·1)	87·4%	10·9% (4·8–18·7)
	Cambodia	14 (8)	81·2%	4·9% (1·8–9·2)	90·5%	24·7% (16·1–34·4)
	Ethiopia	2 (2)	95·2%	2·0% (0·0–14·6)	0	9·1% (6·5–12·0)
Drug elimination half-life						
	Rapid	10 (7)	50·4%	14·1% (8·9–20·1)	85·1%	21·3% (10·8–34·1)
	Intermediate	15 (13)	97·0%	14·0% (6·9–22·9)	96·1%	27·2% (18·6–36·7)
	Slow	91 (48)	89·1%	3·7% (2·4–5·3)	95·9%	16·5% (12·6–20·8)
Major ACTs						
	Artemether-lumefantrine	10 (10)	97·2%	15·3% (5·1–29·3)	95·7%	28·3% (16·8–41·3)
	Dihydroartemisinin-piperaquine	13 (12)	86·9%	4·5% (1·2–9·3)	95·2%	16·8% (7·4–28·8)
	Artesunate-mefloquine	19 (15)	89·2%	5·2% (2·9–7·9)	91·2%	10·9% (7·4–15·0)

ACTs=artemisinin-based combination therapies.

* Parasitaemia from *Plasmodium vivax* or *Plasmodium falciparum* after initial infection.

**Figure 3:**
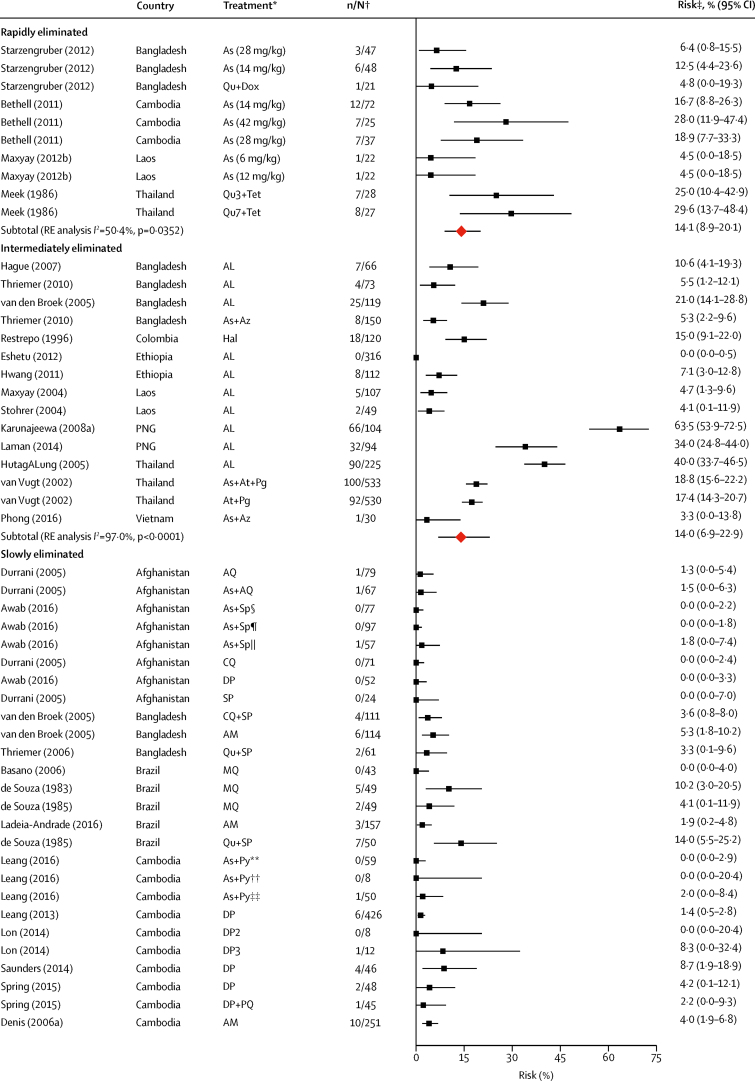

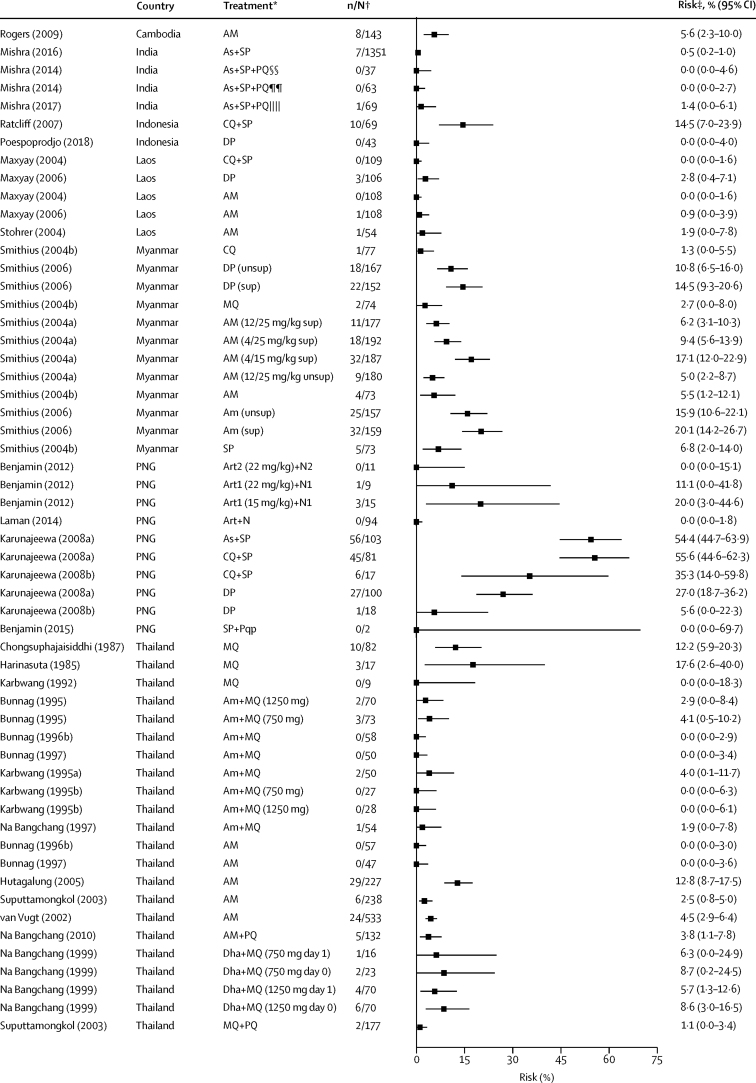

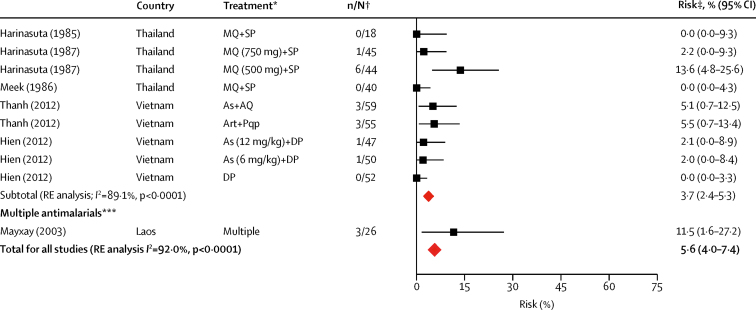
Risk of recurrent *Plasmodium vivax* parasitaemia by day 42 after *Plasmodium falciparum* infection by drug elimination half-life Red diamonds represent subtotals or totals. Full reference citations and study and record details are provided in the appendix pp 14–20, 22–36. As=artesunate. Qu=quinine. Dox=doxycycline. Tet=tetracycline. RE=random effects. AL=artemether-lumefantrine. Az=azithromycin. Hal=halofantrine. PNG=Papua New Guinea. At=atovaquone. Pg=proguanil. AQ=amodiaquine. SP=sulfadoxine-pyrimethamine. CQ=chloroquine. DP=dihydroartemisinin-piperaquine. AM=artesunate-mefloquine. MQ=mefloquine. Py=pyronaridine. PQ=primaquine. unsup=unsupervised. sup=supervised. Art=artemisinin. N=naphthoquine. Pqp=piperaquine. Am=artemether. Dha=dihydroartemisinin. *Treatment group described by drug with number of days given (total dose) if needed to distinguish from other treatment groups. †Data are number of patients with *P vivax* parasitaemia/total evaluable patients at day 42. ‡Risk is the percentage of patients with *P vivax* parasitaemia. §Asadabad, Afghanistan. ¶Jalalabad, Afghanistan. ||Multisite, Afghanistan. **Promoy (Pursat province), Cambodia. ††Tasanh (Battambang province), Cambodia. ‡‡Pailin City (Pailin province), Cambodia. §§Changlang, India. ¶¶Lunglei, India. ||||Gomati, India. ***Multiple antimalarials—studies with aggregated treatment data where drug elimination half-life varies; refer to appendix p 7 for drugs included in rapid, intermediate, and slow half-life elimination categories.

Data on the primary endpoint, *P vivax* parasitaemia by day 42, could be derived from 117 records in 58 studies. The overall risk of *P vivax* parasitaemia at day 42 was 5·6% (95% CI 4·0–7·4; *I*^*2*^=92·0%) with no evidence of publication bias relating to small study effects (p=0·92, table 2, figure 3, appendix p 40). In the univariable meta-regression model, the risk of *P vivax* parasitaemia by day 42 was higher in regions with short *P vivax* relapse periodicity (6·5%, 95% CI 4·6–8·6) compared with regions with long periodicity (1·9%, 0·4–4·0; p=0·0087). The risk of *P vivax* was also greater after treatment with rapidly and intermediately eliminated antimalarial regimens (14·1% [95% CI 8·9–20·1] and 14·0% [6·9–22·9], respectively) compared with slowly eliminated regimens (3·7% [2·4–5·3]; p<0·0001); table 2, table 3, figure 3). In the univariable meta-regression, a higher risk of *P vivax* parasitaemia by day 42 was associated with the proportion of patients with baseline mixed *P falciparum* and *P vivax* infection (p=0·0170), a lower mean age (p=0·0010), higher baseline parasitaemia (p=0·0149), and lower baseline haemoglobin (p<0·0001; table 3).

**Table 3:** Meta-regression of the risk of *Plasmodium vivax* parasitaemia at day 42 after *Plasmodium falciparum* infection

		**Number of records**	**I^2^ (%)**	τ^2^*	**Variance explained (R**^2^**, %)**†	**Univariable**		**Multivariable**‡	
						Coefficient (95% CI)	p value	Coefficient (95% CI)	p value
Overall		117	92·0%	0·0284	..	..	..	..	..
Age (mean, per every 5 years)		88	91·2%	0·0287	11·09%	−0·04 (−0·07 to −0·02)	0·0010	−0·01 (−0·05 to 0·02)	0·5215
Female (%, per every 10%)		103	92·3%	0·0290	2·48%	0·02 (−0·001 to 0·04)	0·0696	0·01 (−0·02 to 0·03)	0·6611
Mixed infection (%, per every 10%)		108	85·7%	0·0148	7·21%	0·08 (0·01 to 0·15)	0·0170	0·16 (−0·05 to 0·37)	0·1282
Baseline parasitaemia (per every 10-fold increase in mean)		108	92·0%	0·0288	4·97%	0·12 (0·02 to 0·22)	0·0149	−0·01 (−0·10 to 0·08)	0·8589
Baseline gametocytaemia (%, per every 10%)		50	89·5%	0·0241	0·00%	0·01 (−0·02 to 0·04)	0·6287	..	..
Baseline haemoglobin (mean, per every 1 g/dL)		71	91·8%	0·0285	21·38%	−0·06 (−0·09 to −0·03)	<0·0001	..	..
Short relapse periodicity§		117	91·2%	0·0268	5·75%	−0·12 (−0·21 to −0·03)	0·0087	−0·11 (−0·19 to −0·03)	0·0072
Region			92·0%	0·0287	0·00%	..	0·6526	..	..
	Asia-Pacific	109	..	..	..	0·26 (0·23 to 0·30)	..	..	..
	The Americas	6	..	..	..	0·00 (−0·15 to 0·15)	..	..	..
	Africa	2	..	..	..	−0·11 (−0·36 to 0·13)	..	..	..
Drug elimination half-life			90·7%	0·0242	15·20%		<0·0001		<0·0001
	Rapid	10	..	..	..	0·40 (0·29 to 0·51)	..	Reference	..
	Intermediate	15	..	..	..	−0·01 (−0·15 to 0·13)	..	−0·06 (−0·18 to 0·06)	..
	Slow	91	..	..	..	−0·18 (−0·30 to −0·06)	..	−0·21 (−0·32 to −0·11)	..
Year data collected (per every 5-year increase)		117	91·9%	0·0284	0·00%	−0·01 (−0·03 to 0·01)	0·4079	−0·02 (−0·04 to 0·002)	0·0738

Results from the univariable meta-regression expressed as θ_i_=β_0_+b_i_+β_1_x_i_+ɛ_i_, where θ_i_ is the Freeman-Tukey double arcsine transformed treatment completion rate from record i, β_0_ is the intercept, b_i_ is the random effect for record i, x_i_ is the value of the covariate from study i, and ɛ_i_ is the within-study error.

* The between-study variance (τ^2^) from a meta-regression model with no covariates can be compared with τ^2^ from univariable meta-regression models to estimate how much variation each covariate explains. For categorical covariates, τ^2^ is provided for the overall covariate.

† For categorical covariates, variance explained is provided for the overall covariate.

‡ Meta-regression includes 74 records, *I*^2^=75·94%, τ^2^=0·0091, R^2^=42·74%. Baseline haemoglobin was not included in the model because of strong correlation with age (Pearson correlation coefficient 0·86; p<0·0001); region was not included in the model because of correlation with relapse periodicity; gametocyte percentage was not included in the model because of low availability of data.

§ Short relapse periodicity is referenced against long relapse periodicity.

Multivariable meta-regression of the risk of *P vivax* parasitaemia at day 42 was done in 74 (63%) of 117 records for which data on all included covariates were available (table 3). The magnitude of effect was derived from pooled estimates of the risk of *P vivax* using these 74 records (appendix pp 44–45). Studies from areas of short relapse periodicity had a risk of 4·9% (95% CI 3·3–6·7) compared with 0·6% (0·0–1·7) in areas of long relapse periodicity (p=0·0072; appendix p 44). Rapidity of drug elimination was the only other significant factor in multivariable analysis with an 11·7% (95% CI 7·1–17·1) risk of *P vivax* in patients treated with a rapidly eliminated drug regimen, a 9·6% (4·1–17·1) risk after treatment with an intermediately eliminated regimen, and a 2·0% (1·2–3·0) risk in patients treated with a slowly eliminated regimen (p<0·0001; table 3, appendix p 45). Baseline haemoglobin was not included in this meta-regression because of collinearity with age. A sensitivity analysis including baseline haemoglobin instead of age identified that, in addition to short relapse periodicity (p=0·0019) and faster drug elimination (p<0·0001), recurrent vivax parasitaemia was associated with lower baseline haemoglobin (p=0·0067) and a lower proportion of female patients (p=0·0225; appendix p 49).

The risk of *P vivax* parasitaemia after treatment for *P falciparum* malaria was 3·8% (95% CI 2·8–4·9; *I*^*2*^=86·1%; 213 records) by day 28 and 24·0% (18·3–30·1; *I*^*2*^=94·4; 30 records) by day 63 (appendix pp 38–39, 50–52). Although there was potential publication bias related to small study effects at day 28 (p=0·0018), this was not apparent after adjusting for drug elimination half-life (p=0·2185; appendix pp 41–42). The majority of estimates for day 63 were for rapidly eliminated drug regimens (26 [87%] of 30) and from regions of short relapse periodicity (25 [83%] of 30). There was no evidence of publication bias relating to small study effects at day 63 (p=0·3586; appendix p 43).

In univariable meta-regression analyses, studies in regions with short relapse periodicity had a greater risk of *P vivax* parasitaemia at both day 28 (p=0·0069) and day 63 (p=0·0381) compared with regions with long relapse periodicity; however, in multivariable analyses these effects were no longer apparent (appendix pp 53–54). In multivariable analysis, the risk of *P vivax* parasitaemia at day 28 was greater in patients treated with rapidly eliminated drugs than in those treated with intermediately or slowly eliminated drugs (p<0·0001), but this was no longer significant at day 63 (p=0·6039; appendix pp 46, 53–54). Additional factors associated with *P vivax* parasitaemia at day 28 and 63 are described in the appendix (pp 47–48, 53–54).

The effect of the speed of drug elimination was explored for three commonly recommended ACTs: artemether-lumefantrine, an intermediately eliminated drug regimen, and dihydroartemisinin-piperaquine and artesunate-mefloquine, both of which are slowly eliminated. At day 28, the risks of *P vivax* were less than 2·0% for all these ACTs, but by day 42 the risk of *P vivax* had risen to 15·3% (95% CI 5·1–29·3; *I*^*2*^=97·2%; ten estimates) for artemether-lumefantrine compared with 4·5% (1·2–9·3; *I*^*2*^=86·9%; 13 estimates) for dihydroartemisinin-piperaquine, and 5·2% (2·9–7·9; *I*^*2*^=89·2%; 19 estimates) for artesunate-mefloquine (figure 2, appendix pp 55–57). The risk of *P vivax* was greater after treatment with artemether-lumefantrine than with dihydroartemisinin-piperaquine or artesunate-mefloquine at day 28, day 42, and day 63. By day 63, the risk of *P vivax* parasitaemia was high after treatment with all of the ACTs: 33·5% (95% CI 27·7–39·5; one estimate) after artemether-lumefantrine, 16·2% (7·6–27·2; *I*^*2*^=96·4%; eight estimates) after dihydroartemisinin-piperaquine, and 15·7% (8·6–24·4; *I*^*2*^=92·0%; nine estimates) after artesunate-mefloquine (figure 2, appendix p 39).

The risk of *P vivax* parasitaemia at day 42 after treatment with artemether-lumefantrine versus the more slowly eliminated ACTs (artesunate-mefloquine and dihydroartemisinin-piperaquine) was compared in 25 studies using multivariable meta-regression analysis controlling for age, baseline parasitaemia, presence of mixed infection at baseline, and regional relapse periodicity. The risk of *P vivax* parasitaemia was higher after treatment with artemether-lumefantrine (9·0%, 95% CI 1·8–20·3) than with dihydroartemisinin-piperaquine or artesunate-mefloquine (2·2%, 0·9–3·8; p=0·0049; appendix pp 58–59). Only one study provided data for day 63 in patients treated with artemether-lumefantrine, precluding comparison between ACTs at this timepoint.

## Discussion

Our systematic review and meta-analysis of 31 262 patients treated for falciparum malaria shows a high risk of subsequent *P vivax* parasitaemia across a range of co-endemic settings. *P vivax* parasitaemia occurred more frequently after treatment with rapidly eliminated drugs and in regions with short relapse periodicity. The risk was particularly apparent after treatment with artemether-lumefantrine (15·3% by day 42), accounting for more than half of all recurrent parasitaemias.

The high risk of *P vivax* after treatment for *P falciparum* interaction has been described previously in a pooled analysis of patients from Thailand with a 32% cumulative risk of *P vivax* parasitaemia 63 days after treatment.^2^ Our meta-analysis highlights similar findings across studies from 21 countries, demonstrating that at day 63 the risk of *P vivax* was 24% and accounted for almost 70% of all recurrent parasitaemias. Although these observations could reflect simultaneous infection of patients by a mosquito carrying both species, entomological data suggest that this is unlikely.^18^ The timing of recurrent vivax parasitaemia and the high efficacy of the initial falciparum treatment against the blood stages, but not liver stages, of *P vivax* suggest that the vivax recurrences were attributable to reactivation of *P vivax* hypnozoites.^18^ The mechanisms underlying the reactivation of dormant parasites are unknown, although acute febrile illness and parasite-induced haemolysis have been proposed.^19^, ^20^, ^21^

Previous studies have shown that the risk of *P vivax* infection after radical cure of primary vivax infection with primaquine and chloroquine, a slowly eliminated schizontocidal drug, is 0·4 infections per person per year in Ethiopia^22^ and 0·26 infections per year on the Thailand–Myanmar border.^23^ Assuming that these are all new infections and that slowly eliminated ACTs provide 4 weeks of prophylactic suppression of parasitaemia, the expected risk of *P vivax* recurrence by day 63 would be estimated conservatively to be 3·8% in Ethiopia and 2·5% on the Thailand–Myanmar border. The pooled risks in our study were far greater, suggesting that new infections alone could not have accounted for these parasitaemias. Assuming that the excessive risk of *P vivax* parasitaemia after *P falciparum* infection is from reactivation of hypnozoites, the number needed to treat with radical cure to prevent one vivax recurrence by day 63 after *P falciparum* would be between 4·7 and 5·0.

In the previous study from Thailand, the risk of *P vivax* parasitaemia varied significantly with the elimination half-life of the longer-acting drug of the antimalarial combination used.^2^ In equatorial regions, relapse of *P vivax* usually has a short periodicity, the first relapse occurring 21 days or more after the initial infection.^6^, ^11^, ^22^ Lumefantrine has an elimination half-life of 4 days and thus blood concentrations will be minimal after day 16 (four half-lives),^24^ therefore providing almost no post-treatment prophylaxis against *P vivax* emerging from reactivation of dormant hypnozoites. In our analysis, artemether-lumefantrine was associated with a four times greater risk of *P vivax* parasitaemia by day 42 compared with dihydroartemisinin-piperaquine and artesunate-mefloquine, which have substantially longer elimination half-lives, providing greater post-treatment prophylaxis against vivax parasitaemia.^6^, ^25^, ^26^ Similar observations have been made in *P vivax* efficacy studies in which artemether-lumefantrine is associated with high risks of recurrent *P vivax* after treatment,^22^, ^27^ far greater than that for artesunate-mefloquine or dihydroartemisinin-piperaquine.^6^, ^27^

Although artesunate-mefloquine and dihydroartemisinin-piperaquine delayed *P vivax* parasitaemia in our meta-analysis, the risk of *P vivax* by day 63 was greater than 15% after treatment with all of the ACTs assessed. WHO antimalarial guidelines recommend changing antimalarial treatment policy if the risk of *P falciparum* recrudescence exceeds a 10% threshold.^8^ At day 42, the risk of *P vivax* parasitaemia was 15·3% after treatment with artemether-lumefantrine and the risk of any parasitaemia was 10·9% or greater after treatment with any of the major ACTs. Hence, although slowly eliminated ACTs provide an early benefit over more rapidly eliminated ACTs, this effect is transient. Therefore, consideration should be given to co-administration of primaquine or tafenoquine to ensure eradication of *P vivax* hypnozoites in all patients presenting with malaria in co-endemic regions. However, our findings cannot necessarily be extrapolated to returning travellers in whom the risk of co-infection might be substantially lower.

Primaquine, the only widely available hypnozoitocidal agent for *P vivax*, has blood schizontocidal activity against *P vivax*^28^ and gametocytocidal activity against *P falciparum.*^29^ In co-endemic regions, radical cure combining an effective ACT with a 14-day primaquine regimen would potentially reduce the risk of recurrent *P falciparum* and *P vivax*, and decrease ongoing transmission in patients presenting with *P falciparum* monoinfection. Primaquine can induce substantial haemolysis in patients with glucose-6-phosphate-dehydrogenase deficiency;^30^ this risk needs to be weighed against the benefits of reducing *P vivax* parasitaemia and preventing the cumulative morbidity associated with multiple relapses.^5^, ^31^ The licensing of tafenoquine as a single-dose hypnozoitocidal agent provides an alternative approach, avoiding the problems of poor adherence to prolonged primaquine regimens.^32^ A policy of universal radical cure of malaria would be most beneficial in equatorial regions where the risk of *P vivax* parasitaemia and frequency of relapse are greatest, although this will need to be confirmed by prospective clinical trials. Additional modelling of the risks and benefits of universal radical cure will be needed to define priority locations where such a policy should be advocated.

Our study has some limitations. Comparison of studies with variable follow-up and the use of aggregated results could potentially bias our results. Studies were only included if they explicitly documented the occurrence or not of *P vivax*, hence patients without any *P vivax* parasitaemia might have been under-represented if the study did not report this finding, leading to an artificial increase in the derived risk of *P vivax* parasitaemia after *P falciparum* infection. However, this bias is likely to be relatively minor, given that our results from studies from Thailand (appendix p 39) were similar to those published previously in a pooled analysis of individual patient data.^2^ We also assumed that recurrent *P falciparum* and *P vivax* parasitaemia occurred in separate patients unless specified in the report. Although this might have led to an elevated risk of parasitological failure, the risk of more than one recurrence within the 42 days after treatment is very low. Our study is also limited by heterogeneity of the studies and changes in epidemiology over the duration of the review, hence our findings might not necessarily be generalisable to all co-endemic regions. Finally, confidence intervals for pooling risk estimates include both between-study and within-study variation, and should be interpreted cautiously. Future meta-analyses pooling individual patient data will avoid many of these limitations and provide greater understanding regarding the factors associated with *P vivax* parasitaemia after *P falciparum* infection.

In summary, there is a high risk of *P vivax* parasitaemia after treatment for *P falciparum* infection in co-endemic regions. The risk is apparent for the major ACTs used in most *P falciparum* endemic regions and greatest in southeast Asia where *P vivax* relapse periodicity is short. Our findings suggest that in some regions co-endemic for both *P falciparum* and *P vivax*, the introduction of a universal policy of radical cure for all patients with uncomplicated malaria has potential to prevent recurrent parasitaemia, reduce ongoing transmission, and enhance malaria elimination efforts.
