# Supplementary material for: Risk of Plasmodium vivax parasitaemia after Plasmodium falciparum infection: a systematic review and meta-analysis
Source: Lancet Infect Dis. 2019 Jan;19(1):91–101. doi: 10.1016/S1473-3099(18)30596-6 (PMC6300482; doi:10.1016/S1473-3099(18)30596-6)
Supplement: Supplementary appendix [file mmc1.pdf]

THE LANCET Infectious Diseases

Supplementary webappendix

This webappendix formed part of the original submission and has been peer reviewed. We post it as supplied by the authors.

Supplement to: Commons RJ, Simpson JA, Thriemer K, et al. Risk of Plasmodium vivax parasitaemia after Plasmodium falciparum infection: a systematic review and meta-analysis. *Lancet Infect Dis* 2019; **19:** 91–101.

Appendix

Commons *et al*, The risk of *Plasmodium vivax* parasitaemia after *P. falciparum* infection: a systematic review and meta-analysis

	Page
**1. Countries co-endemic for *P. falciparum* and *P. vivax***	2
Table S1. Countries co-endemic for *P. falciparum* and *P. vivax* with stable transmission	2
**2. Search strategy**	3
**3. PRISMA checklist**	4
**4. Elimination half-life of antimalarial drugs**	7
Table S2. Individual drugs categorised by elimination half-life	7
Table S3. Treatment arms categorised by elimination half-life	8
**5. Assessment of risk of bias relating to individual studies**	10
Table S4. Risk of bias assessment – Minimum criteria for low risk	10
Figure S1. Risk of bias assessment	11
**6. References of included studies**	14
**7. Studies and study sites**	21
Table S5. Studies excluded from meta-analysis	21
Table S6. Studies included in meta-analysis	22
Table S7. Study sites and treatment arms (records) included in meta-analysis	27
**8. Map of study sites**	37
Figure S2. Location of study sites	37
**9. Tables of overall pooled estimates**	38
Table S8. Day 28	38
Table S9. Day 63	39
**10. Funnel plots**	40
Figure S3. Day 42	40
Figure S4. Day 28	41
Figure S5. Day 28 adjusted for drug elimination half-life	42
Figure S6. Day 63	43
**11. Forest plots categorised by independent risk factors using the subset of records included in the multivariable meta-regressions**	44
Figure S7. Day 42 by relapse periodicity	44
Figure S8. Day 42 by drug elimination half-life	45
Figure S9. Day 28 by drug elimination half-life	46
Figure S10. Day 28 by baseline haemoglobin	47
Figure S11. Day 63 by percentage of mixed infections at baseline	48
**12. Day 42 multivariable meta-regression sensitivity analysis**	49
Table S10. Day 42 multivariable meta-regression with baseline haemoglobin	49
**13. Forest plots of overall pooled estimates including data from all available records**	50
Figure S12. Day 28	50
Figure S13. Day 63	52
**14. Meta-regression analyses for all treatments**	53
Table S11. Day 28	53
Table S12. Day 63	54
**15. Forest plots of pooled estimates for artemisinin-based combination therapy**	55
Figure S14. Day 28	55
Figure S15. Day 42	56
Figure S16. Day 63	57
**16. Meta-regression analyses for artemisinin-based combination therapy**	58
Table S13. Day 42	58
Figure S17. Forest plot of day 42 risk by ACT	59
**17. Excluded references**	60

Appendix 1. Countries co-endemic for *P. falciparum* and *P. vivax*

Table S1. Countries co-endemic for *P. falciparum* and *P. vivax* with stable transmission

Country	Number of full text studies reviewed*	Number of studies included in review*
Co-endemic		
Afghanistan	2	2
Bangladesh	10	5
Bhutan	0	0
Bolivia (Plurinational State of)	1	1
Brazil	20	10
Cambodia	15	12
Colombia	10	2
Djibouti	0	0
Ecuador	1	0
Eritrea	1	0
Ethiopia	13	7
French Guiana	0	0
Guatemala	0	0
Guyana	2	2
Honduras	1	0
India	51	7
Indonesia	26	12
Iran (Islamic Republic of)	3	0
Lao People's Democratic Republic	10	7
Malaysia	2	0
Mayotte†	1	0
Myanmar	16	7
Nepal	1	1
Nicaragua	0	0
Pakistan	5	0
Panama	0	0
Papua New Guinea	14	6
Peru	6	2
Philippines	5	1
Saudi Arabia	0	0
Solomon Islands	1	0
Somalia†	2	0
Sudan	26	0
Suriname	1	0
Thailand	135	59
Timor-Leste	2	1
Vanuatu	2	1
Venezuela (Bolivarian Republic of)	1	0
Viet Nam	24	4
Yemen	2	0
Previously co-endemic		
China‡	10	2
Madagascar§	4	0
Sri Lanka¶	3	0

Co-endemicity defined as countries where indigenous *P. falciparum* and *P. vivax* cases were reported or suspected in 2016[1]; * Studies with patients from multiple countries are not included; † Number of cases in 2016 not available[1]; ‡ No *P. falciparum* reported in 2016[1]; § *P. vivax* reported in recent publications but number of cases in 2016 not available[1]; ¶ Indigenous malaria has now been eliminated.

Appendix 2. Search Strategy

Search strategy
All prospective *P. falciparum* antimalarial clinical studies published in any language between January 1, 1960 and January 5, 2018 that explicitly reported both *P. vivax* and *P. falciparum* recurrent parasitaemias following *P. falciparum* infection were identified by the application of the key terms (listed below) through Medline (Pubmed), Web of Science, Embase and the Cochrane Database of Systematic Reviews. Abstracts of all references containing any mention of studies of antimalarial drugs were manually checked to confirm prospective clinical studies, with review of full text when needed. Full texts of all potential references were manually checked for explicit reporting of *P. vivax* and *P. falciparum* recurrence. Studies were excluded if the observation period was <28 days, they assessed prevention or prophylaxis, only pregnant women or patients with severe/complicated malaria were enrolled, the numbers of recurrent *P. vivax* and *P. falciparum* parasitaemias observed within the study observation period could not be extracted, follow up was not active, data were extracted retrospectively from medical records outside of a planned trial, or if the full text manuscript was unavailable.

Key terms:
Literature search (conducted January 2018) with the following key terms (version undertaken in Pubmed): falciparum AND (allopurinol OR amodiaquine OR atovaquone OR artemisinin OR arteether OR artesunate OR artemether OR artemotil OR atovaquone OR azithromycin OR artekin OR chloroquine OR chlorproguanil OR cycloguanil OR clindamycin OR coartem OR dapsone OR dihydroartemisinin OR duo-cotecxin OR doxycycline OR halofantrine OR lumefantrine OR lariam OR malarone OR mefloquine OR naphthoquine OR naphthoquinone OR pafuramidine OR piperaquine OR primaquine OR proguanil OR pyrimethamine OR pyronaridine OR proguanil OR quinidine OR quinine OR riamet OR sulphadoxine OR sulfamethoxazole OR tetracycline OR tafenoquine).

Appendix 3. PRISMA Checklist

Section/topic	#	Checklist item	Reported on page #
**TITLE**			
Title	1	Identify the report as a systematic review, meta-analysis, or both.	1
**ABSTRACT**			
Structured summary	2	Provide a structured summary including, as applicable: background; objectives; data sources; study eligibility criteria, participants, and interventions; study appraisal and synthesis methods; results; limitations; conclusions and implications of key findings; systematic review registration number.	2
**INTRODUCTION**			
Rationale	3	Describe the rationale for the review in the context of what is already known.	4
Objectives	4	Provide an explicit statement of questions being addressed with reference to participants, interventions, comparisons, outcomes, and study design (PICOS).	4
**METHODS**			
Protocol and registration	5	Indicate if a review protocol exists, if and where it can be accessed (e.g., Web address), and, if available, provide registration information including registration number.	6
Eligibility criteria	6	Specify study characteristics (e.g., PICOS, length of follow-up) and report characteristics (e.g., years considered, language, publication status) used as criteria for eligibility, giving rationale.	5
Information sources	7	Describe all information sources (e.g., databases with dates of coverage, contact with study authors to identify additional studies) in the search and date last searched.	4-5
Search	8	Present full electronic search strategy for at least one database, including any limits used, such that it could be repeated.	Appendix page 3
Study selection	9	State the process for selecting studies (i.e., screening, eligibility, included in systematic review, and, if applicable, included in the meta-analysis).	4-5, Fig 1, Appendix page 21
Data collection process	10	Describe method of data extraction from reports (e.g., piloted forms, independently, in duplicate) and any processes for obtaining and confirming data from investigators.	5
Data items	11	List and define all variables for which data were sought (e.g., PICOS, funding sources) and any assumptions and simplifications made.	5

Risk of bias in individual studies	12	Describe methods used for assessing risk of bias of individual studies (including specification of whether this was done at the study or outcome level), and how this information is to be used in any data synthesis.	6
Summary measures	13	State the principal summary measures (e.g., risk ratio, difference in means).	5-6
Synthesis of results	14	Describe the methods of handling data and combining results of studies, if done, including measures of consistency (e.g., I^2) for each meta-analysis.	6

Section/topic	#	Checklist item	Reported on page #
Risk of bias across studies	15	Specify any assessment of risk of bias that may affect the cumulative evidence (e.g., publication bias, selective reporting within studies).	Appendix pages 10-13
Additional analyses	16	Describe methods of additional analyses (e.g., sensitivity or subgroup analyses, meta-regression), if done, indicating which were pre-specified.	6
**RESULTS**			
Study selection	17	Give numbers of studies screened, assessed for eligibility, and included in the review, with reasons for exclusions at each stage, ideally with a flow diagram.	6-7, Fig 1, Appendix pages 21-26
Study characteristics	18	For each study, present characteristics for which data were extracted (e.g., study size, PICOS, follow-up period) and provide the citations.	Appendix pages 14-36
Risk of bias within studies	19	Present data on risk of bias of each study and, if available, any outcome level assessment (see item 12).	Appendix pages 10-13
Results of individual studies	20	For all outcomes considered (benefits or harms), present, for each study: (a) simple summary data for each intervention group (b) effect estimates and confidence intervals, ideally with a forest plot.	Fig 3, Appendix pages 50-52
Synthesis of results	21	Present results of each meta-analysis done, including confidence intervals and measures of consistency.	Table 2, Appendix pages 38-39

Risk of bias across studies	22	Present results of any assessment of risk of bias across studies (see Item 15).	Table 3, Appendix pages 53-54
Additional analysis	23	Give results of additional analyses, if done (e.g., sensitivity or subgroup analyses, meta-regression [see Item 16]).	Appendix pages 44-49, 53-59
**DISCUSSION**			
Summary of evidence	24	Summarize the main findings including the strength of evidence for each main outcome; consider their relevance to key groups (e.g., healthcare providers, users, and policy makers).	9-11
Limitations	25	Discuss limitations at study and outcome level (e.g., risk of bias), and at review-level (e.g., incomplete retrieval of identified research, reporting bias).	10
Conclusions	26	Provide a general interpretation of the results in the context of other evidence, and implications for future research.	10-12
**FUNDING**			
Funding	27	Describe sources of funding for the systematic review and other support (e.g., supply of data); role of funders for the systematic review.	6

From: Moher D, Liberati A, Tetzlaff J, Altman DG, The PRISMA Group (2009). Preferred Reporting Items for Systematic Reviews and Meta-Analyses: The PRISMA Statement. PLoS Med 6(7): e1000097. doi:10.1371/journal.pmed1000097

For more information, visit: **www.prisma-statement.org**.

Appendix 4. Elimination half-life of antimalarial drugs

Table S2. Individual drugs categorised by elimination half-life

Rapid (<1 day)	Intermediate (1-7 days)	Slow (>7 days)
Artemisinins	Atovaquone	Amodiaquine
Clindamycin	Azithromycin	Chloroquine
Doxycycline	Dapsone	Mefloquine
Primaquine	Halofantrine	Naphthoquine
Proguanil	Lumefantrine	Piperaquine
Quinine	Pafuramidine	Pyronaridine
Sulfamethoxazole	Pyrimethamine	Sulfadoxine
Tetracycline		

Categorisation of combination therapies based upon the drug with the longest elimination half-life

Table S3. Treatment arms categorised by drug elimination half-life

Treatment arm	Number of arms (% (n)) (n=323)	Number of patients
**Slow elimination half-life regimens**		
Amodiaquine	1.2% (4)	194
Artemether + mefloquine	3.1% (10)	606
Artemisinin + mefloquine	0.3% (1)	20
Artemisinin + mefloquine + sulfadoxine-pyrimethamine	0.3% (1)	20
Artemisinin + napthoquine	1.6% (5)	201
Artemisinin + piperaquine	0.3% (1)	63
Artemisinin + piperaquine + primaquine	0.3% (1)	65
Arterolane + piperaquine	1.2% (4)	160
Artesunate + amodiaquine	0.6% (2)	144
Artesunate + amodiaquine +/- primaquine	0.3% (1)	155
Artesunate + chloroquine	0.9% (3)	66
Artesunate + dihydroartemisinin-piperaquine	0.9% (3)	178
Artesunate-mefloquine	11.8% (38)	6370
Artesunate-mefloquine + primaquine	0.3% (1)	150
Artesunate-mefloquine +/- primaquine	0.6% (2)	330
Artesunate + pyronaridine	0.9% (3)	123
Artesunate + sulfadoxine-pyrimethamine	4.1% (13)	3527
Artesunate + sulfadoxine-pyrimethamine + primaquine	0.9% (3)	190
Chloroquine	4.0% (13)	774
Chloroquine + sulfadoxine-pyrimethamine	1.9% (6)	495
Chloroquine + sulfadoxine-pyrimethamine +/- primaquine	0.3% (1)	88
Dihydroartemisinin + mefloquine	1.2% (4)	207
Dihydroartemisinin + napthoquine + trimethoprim	0.3% (1)	89
Dihydroartemisinin-piperaquine	7.4% (24)	3104
Dihydroartemisinin-piperaquine + primaquine	0.3% (1)	50
Dihydroartemisinin-piperaquine +/- primaquine	0.3% (1)	161
Dihydroartemisinin-piperaquine + trimethoprim+primaquine	0.3% (1)	234
Mefloquine	5.3% (17)	1098
Mefloquine + doxycycline	0.3% (1)	54
Mefloquine + primaquine	0.3% (1)	236
Mefloquine + sulfadoxine-pyrimethamine	1.9% (6)	1892
Mefloquine +/- sulfadoxine-pyrimethamine	0.3% (1)	75
Mefloquine + sulfadoxine-pyrimethamine + tetracycline	0.3% (1)	26
Mefloquine + tetracycline	0.3% (1)	25
Pyronaridine	0.6% (2)	101
Quinine + sulfadoxine-pyrimethamine	0.6% (2)	113
Sulfadoxine-pyrimethamine	3.4% (11)	458
Sulfadoxine-pyrimethamine + piperaquine	0.3% (1)	2
**Intermediate elimination half-life regimens**		
Artemether + azithromycin	0.3% (1)	30
Artemether + pyrimethamine	0.9% (3)	60
Artemether-lumefantrine	9.9% (32)	4016
Artemether-lumefantrine +/- primaquine	0.3% (1)	162
Artesunate + atovaquone-proguanil	0.3% (1)	533
Artesunate + azithromycin	0.6% (2)	182
Atovaquone-proguanil	1.2% (4)	730
Azithromycin + quinine	0.9% (3)	50
Dapsone – pyrimethamine	0.3% (1)	22
Halofantrine	1.9% (6)	322
Pafuramidine	0.3% (1)	23

**Rapid elimination half-life regimens**		
Arteether	0.3% (1)	37
Artemether	3.4% (11)	340
Artemether + doxycycline	0.3% (1)	30
Artemisinin	0.3% (1)	20
Artesunate	8.4% (27)	876
Artesunate + doxycycline	0.3% (1)	55
Artesunate + primaquine	0.6% (2)	77
Artesunate + tetracycline	0.3% (1)	88
Clindamycin	1.6% (5)	97
Dihydroartemisinin	0.3% (1)	53
Proguanil + sulfamethoxazole	0.3% (1)	31
Quinine	0.9% (3)	124
Quinine + clindamycin	0.3% (1)	68
Quinine + doxycycline	0.6% (2)	35
Quinine + primaquine	0.6% (2)	66
Quinine + tetracycline	3.4% (11)	569
**Multiple antimalarials – variable elimination half-life**		
Artesunate +/- sulfadoxine-pyrimethamine	0.3% (1)	197
WR 33063	0.3% (1)	25
Mixed treatment regimens	0.9% (3)	500

Appendix 5. Assessment of risk of bias relating to individual studies

The Joanne Briggs Institute Critical Appraisal Tool for Systematic Reviews of Prevalence Studies[13] was used to assess bias for each study within nine domains: 1) Sample frame, 2) Recruitment, 3) Sample size, 4) Subject and setting description, 5) Sufficient coverage, 6) Method of identification, 7) Method reliability, 8) Analysis, and 9) Response rate. Risk of bias was assessed for the prevalence of recurrent *P. vivax* and *P. falciparum* parasitaemia rather than the study itself. Risk of bias was assessed by one independent reviewer (RJC) with a second independent reviewer (ND) assessing 10% of studies with >95% inter-reviewer agreement.

Table S4. Risk of bias assessment – Minimum criteria for low risk

Domain	Minimum criteria for low risk	Comments
1. Sample frame	Male and female patients, and children and adults included. No other significant bias in inclusion criteria	
2. Recruitment	Site of recruitment (eg hospital, clinic) described and did not exclude specific groups (eg drug sensitive patients)	
3. Sample size	Sample size was adequate (considered to be >138 patients in each treatment arm).	Derived using the formula by Naing *et al* described in the Joanne Briggs Institute tool.[13,33] *P. vivax* parasitaemia assumed to be 10%.
4. Subject and setting description	Setting and subjects described including age, gender, baseline parasitaemia and presence of mixed infection.	
5. Sufficient coverage (Group comparison)	Loss to follow up similar between treatment arms or not related to outcome.	
6. Method of identification	Microscopy used to identify recurrent parasitaemia.	Microscopy was used in every study
7. Method reliability	Microscopy used to identify recurrent parasitaemia.	Microscopy was used in every study
8. Analysis	Numerator and denominator presented or able to be calculated for recurrent *P. vivax* and *P. falciparum* parasitaemia for each individual treatment arm and site.	
9. Response rate	<10% of enrolled patients lost to follow up or reasons for lost to follow up explained and unrelated to recurrent parasitaemia.	

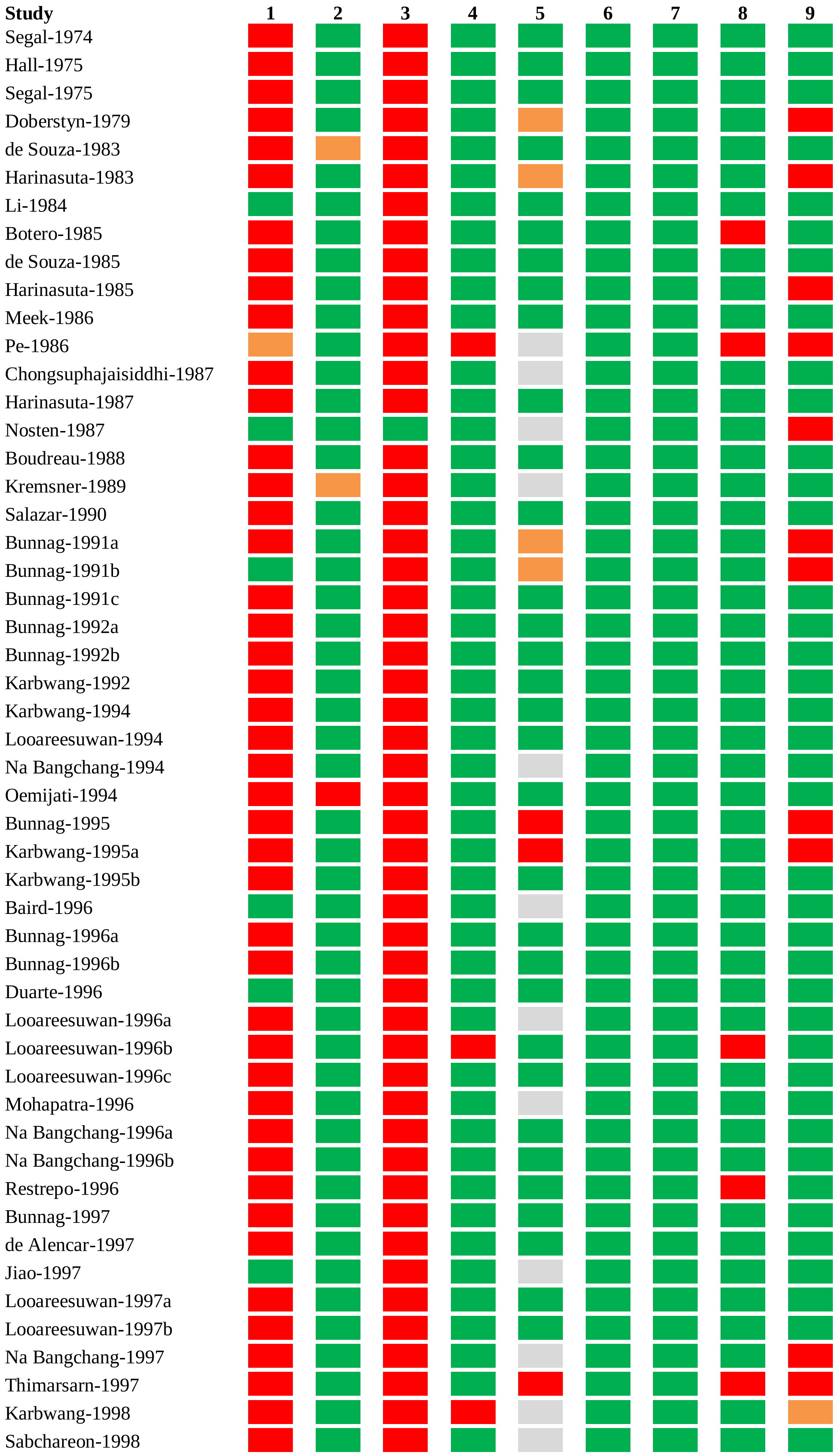
Study
1
2
3
4
5
6
7
8
9
Segal-1974
Hall-1975
Segal-1975
Doberstyn-1979
de Souza-1983
Harinasuta-1983
Li-1984
Botero-1985
de Souza-1985
Harinasuta-1985
Meek-1986
Pe-1986
Chongsuphajaisiddhi-1987
Harinasuta-1987
Nosten-1987
Boudreau-1988
Kremsner-1989
Salazar-1990
Bunnag-1991a
Bunnag-1991b
Bunnag-1991c
Bunnag-1992a
Bunnag-1992b
Karbwang-1992
Karbwang-1994
Looareesuwan-1994
Na Bangchang-1994
Oemijati-1994
Bunnag-1995
Karbwang-1995a
Karbwang-1995b
Baird-1996
Bunnag-1996a
Bunnag-1996b
Duarte-1996
Looareesuwan-1996a
Looareesuwan-1996b
Looareesuwan-1996c
Mohapatra-1996
Na Bangchang-1996a
Na Bangchang-1996b
Restrepo-1996
Bunnag-1997
de Alencar-1997
Jiao-1997
Looareesuwan-1997a
Looareesuwan-1997b
Na Bangchang-1997
Thimarsarn-1997
Karbwang-1998
Sabchareon-1998

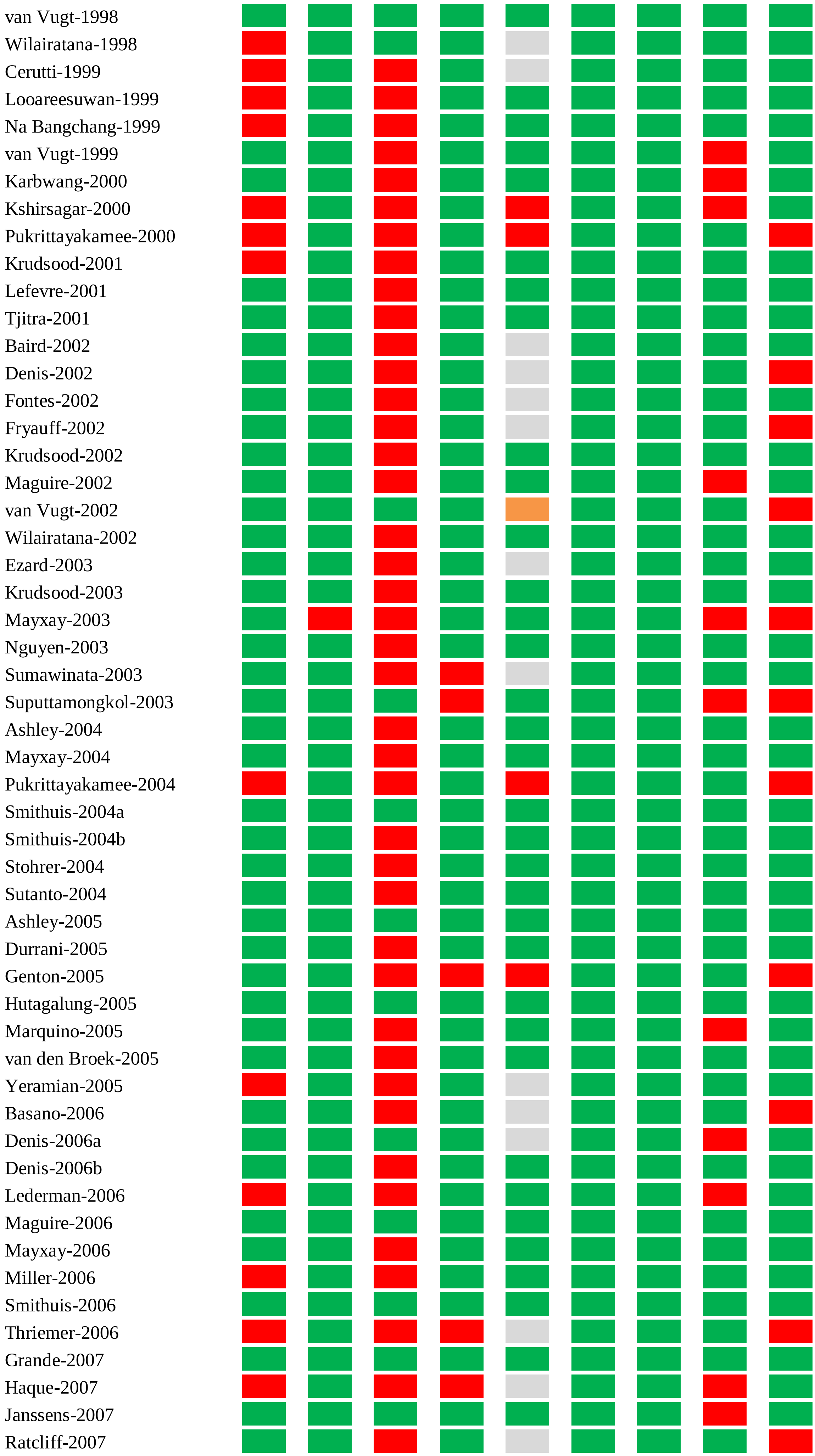
van Vugt-1998
Wilairatana-1998
Cerutti-1999
Looareesuwan-1999
Na Bangchang-1999
van Vugt-1999
Karbwang-2000
Kshirsagar-2000
Pukrittayakamee-2000
Krudsood-2001
Lefevre-2001
Tjitra-2001
Baird-2002
Denis-2002
Fontes-2002
Fryauff-2002
Krudsood-2002
Maguire-2002
van Vugt-2002
Wilairatana-2002
Ezard-2003
Krudsood-2003
Mayxay-2003
Nguyen-2003
Sumawinata-2003
Suputtamongkol-2003
Ashley-2004
Mayxay-2004
Pukrittayakamee-2004
Smithuis-2004a
Smithuis-2004b
Stohrer-2004
Sutanto-2004
Ashley-2005
Durrani-2005
Genton-2005
Hutagalung-2005
Marquino-2005
van den Broek-2005
Yeramian-2005
Basano-2006
Denis-2006a
Denis-2006b
Lederman-2006
Maguire-2006
Mayxay-2006
Miller-2006
Smithuis-2006
Thriemer-2006
Grande-2007
Haque-2007
Janssens-2007
Ratcliff-2007

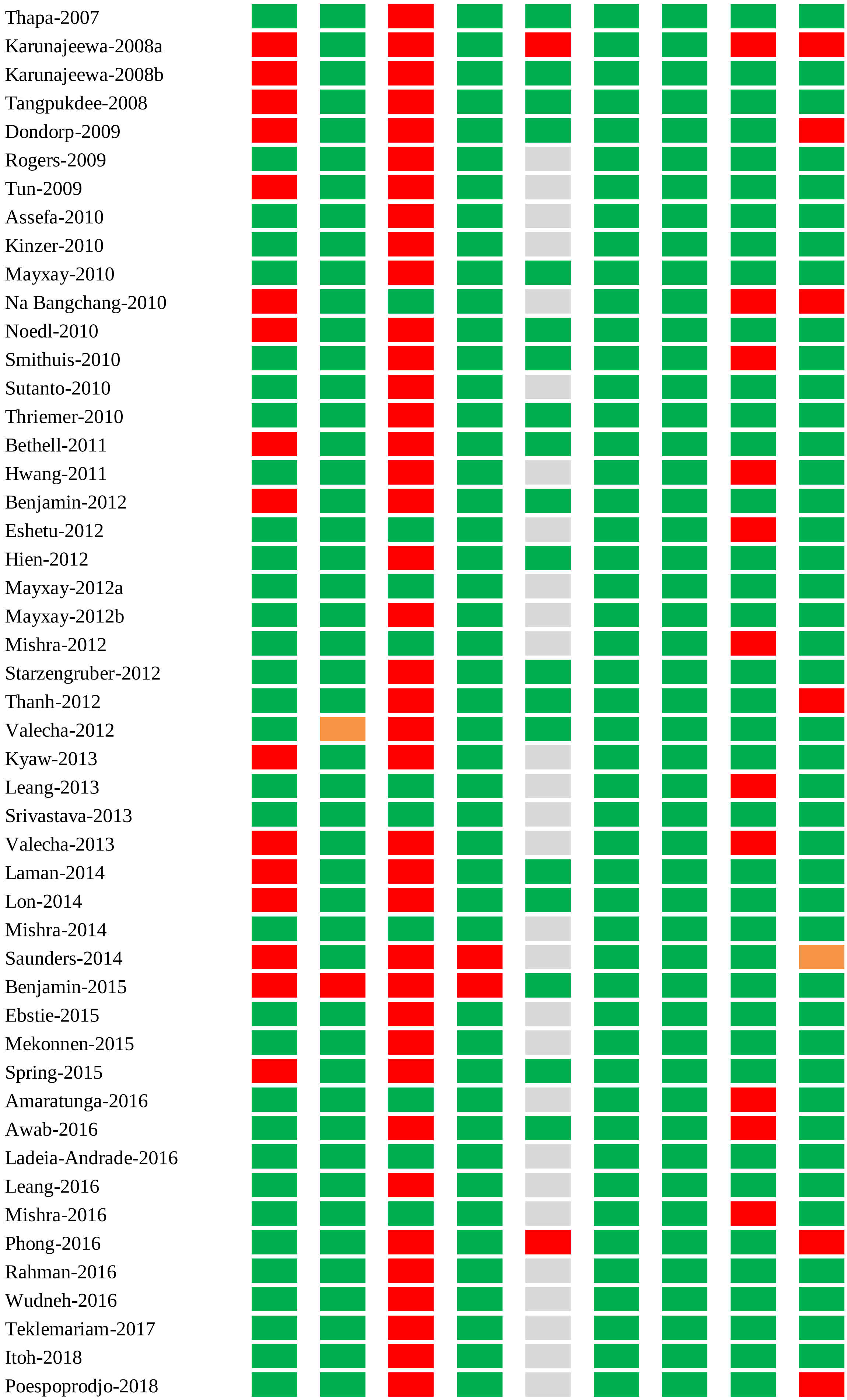

Figure S1. Risk of bias assessment.
Red – High risk; Orange – Unknown; Green – Low risk; Grey – not applicable; Categories of bias 1-9 described in Table S4.

Appendix 6.

References for included studies

34. Amaratunga C, Lim P, Suon S, et al. Dihydroartemisinin-piperaquine resistance in *Plasmodium falciparum* malaria in Cambodia: a multisite prospective cohort study. *Lancet Infect Dis* 2016; **16**(3): 357-65.
35. Ashley EA, Krudsood S, Phaiphun L, et al. Randomized, controlled dose-optimization studies of dihydroartemisinin-piperaquine for the treatment of uncomplicated multidrug-resistant falciparum malaria in Thailand. *J Infect Dis* 2004; **190**(10): 1773-82.
36. Ashley EA, McGready R, Hutagalung R, et al. A randomized, controlled study of a simple, once-daily regimen of dihydroartemisinin-piperaquine for the treatment of uncomplicated, multidrug-resistant falciparum malaria. *Clin Infect Dis* 2005; **41**(4): 425-32.
37. Assefa A, Kassa M, Tadese G, Mohamed H, Animut A, Mengesha T. Therapeutic efficacy of Artemether/Lumefantrine (Coartem(R)) against *Plasmodium falciparum* in Kersa, South West Ethiopia. *Parasit Vectors* 2010; **3**(1): 1.
38. Awab GR, Imwong M, Pukrittayakamee S, et al. Clinical trials of artesunate plus sulfadoxine-pyrimethamine for *Plasmodium falciparum* malaria in Afghanistan: maintained efficacy a decade after introduction. *Malar J* 2016; **15**: 121.
39. Baird JK, Sismadi P, Masbar S, et al. Chloroquine sensitive *Plasmodium falciparum* and *P. vivax* in central Java, Indonesia. *Trans R Soc Trop Med Hyg* 1996; **90**(4): 412-3.
40. Baird JK, Tiwari T, Martin GJ, et al. Chloroquine for the treatment of uncomplicated malaria in Guyana. *Ann Trop Med Parasitol* 2002; **96**(4): 339-48.
41. Basano SA, Bianco A, Taylor WR, Olliaro P, Camargo LM. An in vivo test to assess mefloquine 25 mg/kg for the treatment of uncomplicated falciparum malaria in Rondonia, Brazil. *Braz J Infect Dis* 2006; **10**(4): 279-82.
42. Benjamin J, Moore B, Lee ST, et al. Artemisinin-naphthoquine combination therapy for uncomplicated pediatric malaria: a tolerability, safety, and preliminary efficacy study. *Antimicrob Agents Chemother* 2012; **56**(5): 2465-71.
43. Benjamin JM, Moore BR, Salman S, et al. Population pharmacokinetics, tolerability, and safety of dihydroartemisinin-piperaquine and sulfadoxine-pyrimethamine-piperaquine in pregnant and nonpregnant Papua New Guinean women. *Antimicrob Agents Chemother* 2015; **59**(7): 4260-71.
44. Bethell D, Se Y, Lon C, et al. Artesunate dose escalation for the treatment of uncomplicated malaria in a region of reported artemisinin resistance: a randomized clinical trial. *PLoS One* 2011; **6**(5): e19283.
45. Botero D, Restrepo M, Montoya A. Prospective double-blind trial of two different doses of mefloquine plus pyrimethamine-sulfadoxine compared with pyrimethamine-sulfadoxine alone in the treatment of falciparum malaria. *Bull World Health Organ* 1985; **63**(4): 731-7.
46. Boudreau EF, Pang LW, Dixon KE, et al. Malaria: treatment efficacy of halofantrine (WR 171,669) in initial field trials in Thailand. *Bull World Health Organ* 1988; **66**(2): 227-35.
47. Bunnag D, Viravan C, Looareesuwan S, Karbwang J, Harinasuta T. Double blind randomised clinical trial of oral artesunate at once or twice daily dose in falciparum malaria. *Southeast Asian J Trop Med Public Health* 1991; **22**(4): 539-43.
48. Bunnag D, Viravan C, Looareesuwan S, Karbwang J, Harinasuta T. Double blind randomised clinical trial of two different regimens of oral artesunate in falciparum malaria. *Southeast Asian J Trop Med Public Health* 1991; **22**(4): 534-8.
49. Bunnag D, Viravan C, Looareesuwan S, Karbwang J, Harinasuta T. Clinical trial of artesunate and artemether on multidrug resistant falciparum malaria in Thailand. A preliminary report. *Southeast Asian J Trop Med Public Health* 1991; **22**(3): 380-5.
50. Bunnag D, Karbwang J, Viravan C, Chitamas S, Harinasuta T. Clinical trials of mefloquine with tetracycline. *Southeast Asian J Trop Med Public Health* 1992; **23**(3): 377-82.
51. Bunnag D, Karbwang J, Harinasuta T. Artemether in the treatment of multiple drug resistant falciparum malaria. *Southeast Asian J Trop Med Public Health* 1992; **23**(4): 762-7.
52. Bunnag D, Kanda T, Karbwang J, Thimasarn K, Pungpak S, Harinasuta T. Artemether-mefloquine combination in multidrug resistant falciparum malaria. *Trans R Soc Trop Med Hyg* 1995; **89**(2): 213-5.
53. Bunnag D, Karbwang J, Na-Bangchang K, Thanavibul A, Chittamas S, Harinasuta T. Quinine-tetracycline for multidrug resistant falciparum malaria. *Southeast Asian J Trop Med Public Health* 1996; **27**(1): 15-8.
54. Bunnag D, Kanda T, Karbwang J, Thimasarn K, Pungpak S, Harinasuta T. Artemether or artesunate followed by mefloquine as a possible treatment for multidrug resistant falciparum malaria. *Trans R Soc Trop Med Hyg* 1996; **90**(4): 415-7.

55. Bunnag D, Kanda T, Karbwang J, Thimasarn K, Pungpak S, Harinasuta T. Two doses of artemether/mefloquine or artesunate/mefloquine combination for multidrug resistant falciparum Malaria. *Southeast Asian J Trop Med Public Health* 1997; **28**(4): 727-30.

56. Cerutti C, Jr., Durlacher RR, de Alencar FE, Segurado AA, Pang LW. In vivo efficacy of mefloquine for the treatment of Falciparum malaria in Brazil. *J Infect Dis* 1999; **180**(6): 2077-80.

57. Chongsuphajaisiddhi T, Sabchareon A, Chantavanich P, et al. A phase-III clinical trial of mefloquine in children with chloroquine-resistant falciparum malaria in Thailand. *Bull World Health Organ* 1987; **65**(2): 223-6.

58. de Alencar FE, Cerutti C, Jr., Durlacher RR, et al. Atovaquone and proguanil for the treatment of malaria in Brazil. *J Infect Dis* 1997; **175**(6): 1544-7.

59. de Souza JM. A phase II clinical trial of mefloquine in Brazilian male subjects. *Bull World Health Organ* 1983; **61**(5): 815-20.

60. de Souza JM, Sheth UK, de Oliveira RM, Roulet H, de Souza SD. An open, randomized, phase III clinical trial of mefloquine and of quinine plus sulfadoxine-pyrimethamine in the treatment of symptomatic falciparum malaria in Brazil. *Bull World Health Organ* 1985; **63**(3): 603-9.

61. Denis MB, Davis TM, Hewitt S, et al. Efficacy and safety of dihydroartemisinin-piperaquine (Artekin) in Cambodian children and adults with uncomplicated falciparum malaria. *Clin Infect Dis* 2002; **35**(12): 1469-76.

62. Denis MB, Tsuyuoka R, Poravuth Y, et al. Surveillance of the efficacy of artesunate and mefloquine combination for the treatment of uncomplicated falciparum malaria in Cambodia. *Trop Med Int Health* 2006; **11**(9): 1360-6.

63. Denis MB, Tsuyuoka R, Lim P, et al. Efficacy of artemether-lumefantrine for the treatment of uncomplicated falciparum malaria in northwest Cambodia. *Trop Med Int Health* 2006; **11**(12): 1800-7.

64. Doberstyn EB, Phintuyothin P, Noeypatimanondh S, Teerakiartkamjorn C. Single-dose therapy of falciparum malaria with mefloquine or pyrimethamine-sulfadoxine. *Bull World Health Organ* 1979; **57**(2): 275-9.

65. Dondorp AM, Nosten F, Yi P, et al. Artemisinin resistance in *Plasmodium falciparum* malaria. *N Engl J Med* 2009; **361**(5): 455-67.

66. Duarte EC, Fontes CJ, Gyorkos TW, Abrahamowicz M. Randomized controlled trial of artesunate plus tetracycline versus standard treatment (quinine plus tetracycline) for uncomplicated *Plasmodium falciparum* malaria in Brazil. *Am J Trop Med Hyg* 1996; **54**(2): 197-202.

67. Durrani N, Leslie T, Rahim S, Graham K, Ahmad F, Rowland M. Efficacy of combination therapy with artesunate plus amodiaquine compared to monotherapy with chloroquine, amodiaquine or sulfadoxine-pyrimethamine for treatment of uncomplicated *Plasmodium falciparum* in Afghanistan. *Trop Med Int Health* 2005; **10**(6): 521-9.

68. Ebstie YA, Zeynudin A, Belachew T, Desalegn Z, Suleman S. Assessment of therapeutic efficacy and safety of artemether-lumefantrine (Coartem(R)) in the treatment of uncomplicated *Plasmodium falciparum* malaria patients in Bahir Dar district, Northwest Ethiopia: an observational cohort study. *Malar J* 2015; **14**: 236.

69. Eshetu T, Abdo N, Bedru KH, et al. Open-label trial with artemether-lumefantrine against uncomplicated *Plasmodium falciparum* malaria three years after its broad introduction in Jimma Zone, Ethiopia. *Malar J* 2012; **11**: 240.

70. Ezard N, Burns M, Lynch C, Cheng Q, Edstein MD. Efficacy of chloroquine in the treatment of uncomplicated *Plasmodium falciparum* infection in East Timor, 2000. *Acta Trop* 2003; **88**(1): 87-90.

71. Fontes CJ, Ribeiro LC, Pang LW. Proguanil plus sulfamethoxazole in the treatment of uncomplicated *Plasmodium falciparum* malaria. *Southeast Asian J Trop Med Public Health* 2002; **33**(4): 685-8.

72. Fryauff DJ, Leksana B, Masbar S, et al. The drug sensitivity and transmission dynamics of human malaria on Nias Island, North Sumatra, Indonesia. *Ann Trop Med Parasitol* 2002; **96**(5): 447-62.

73. Genton B, Baea K, Lorry K, Ginny M, Wines B, Alpers MP. Parasitological and clinical efficacy of standard treatment regimens against *Plasmodium falciparum*, *P. vivax* and *P. malariae* in Papua New Guinea. *P N G Med J* 2005; **48**(3-4): 141-50.

74. Grande T, Bernasconi A, Erhart A, et al. A randomised controlled trial to assess the efficacy of dihydroartemisinin-piperaquine for the treatment of uncomplicated falciparum malaria in Peru. *PLoS One* 2007; **2**(10): e1101.

75. Haque R, Thriemer K, Wang Z, et al. Therapeutic efficacy of artemether-lumefantrine for the treatment of uncomplicated *Plasmodium falciparum* malaria in Bangladesh. *Am J Trop Med Hyg* 2007; **76**(1): 39-41.

76. Harinasuta T, Bunnag D, Wernsdorfer WH. A phase II clinical trial of mefloquine in patients with chloroquine-resistant falciparum malaria in Thailand. *Bull World Health Organ* 1983; **61**(2): 299-305.

77. Harinasuta T, Bunnag D, Lasserre R, Leimer R, Vinijanont S. Trials of mefloquine in vivax and of mefloquine plus 'fansidar' in falciparum malaria. *Lancet* 1985; **1**(8434): 885-8.

78. Harinasuta T, Bunnag D, Vanijanond S, et al. Mefloquine, sulfadoxine, and pyrimethamine in the treatment of symptomatic falciparum malaria: a double-blind trial for determining the most effective dose. *Bull World Health Organ* 1987; **65**(3): 363-7.

79. Hall AP, Segal HE, Pearlman EJ, Phintuyothin P, Kosakal S. Amodiaquine resistant falciparum malaria in Thailand. *Am J Trop Med Hyg* 1975; **24**(4): 575-80.

80. Hien TT, Thuy-Nhien NT, Phu NH, et al. *In vivo* susceptibility of *Plasmodium falciparum* to artesunate in Binh Phuoc Province, Vietnam. *Malar J* 2012; **11**: 355.

81. Hwang J, Alemayehu BH, Hoos D, et al. In vivo efficacy of artemether-lumefantrine against uncomplicated *Plasmodium falciparum* malaria in Central Ethiopia. *Malar J* 2011; **10**: 209.

82. Hutagalung R, Paiphun L, Ashley EA, et al. A randomized trial of artemether-lumefantrine versus mefloquine-artesunate for the treatment of uncomplicated multi-drug resistant *Plasmodium falciparum* on the western border of Thailand. *Malar J* 2005; **4**: 46.

83. Itoh M, Negreiros do Valle S, Farias S, et al. Efficacy of Artemether-Lumefantrine for Uncomplicated *Plasmodium falciparum* Malaria in Cruzeiro do Sul, Brazil, 2016. *Am J Trop Med Hyg* 2018; **98**(1): 88-94.

84. Janssens B, van Herp M, Goubert L, et al. A randomized open study to assess the efficacy and tolerability of dihydroartemisinin-piperaquine for the treatment of uncomplicated falciparum malaria in Cambodia. *Trop Med Int Health* 2007; **12**(2): 251-9.

85. Jiao X, Liu GY, Shan CO, et al. Phase II trial in China of a new, rapidly-acting and effective oral antimalarial, CGP 56697, for the treatment of *Plasmodium falciparum* malaria. *Southeast Asian J Trop Med Public Health* 1997; **28**(3): 476-81.

86. Karbwang J, Bangchang KN, Thanavibul A, Bunnag D, Chongsuphajaisiddhi T, Harinasuta T. Comparison of oral artemether and mefloquine in acute uncomplicated falciparum malaria. *Lancet* 1992; **340**(8830): 1245-8.

87. Karbwang J, Na-Bangchang K, Thanavibul A, Bunnag D, Chongsuphajaisiddhi T, Harinasuta T. Comparison of oral artesunate and quinine plus tetracycline in acute uncomplicated falciparum malaria. *Bull World Health Organ* 1994; **72**(2): 233-8.

88. Karbwang J, Na-Bangchang K, Thanavibul A, Laothavorn P, Ditta-in M, Harinasuta T. A comparative clinical trial of artemether and the sequential regimen of artemether-mefloquine in multidrug resistant falciparum malaria. *J Antimicrob Chemother* 1995; **36**(6): 1079-83.

89. Karbwang J, Na-Bangchang K, Thanavibul A, Ditta-in M, Harinasuta T. A comparative clinical trial of two different regimens of artemether plus mefloquine in multidrug resistant falciparum malaria. *Trans R Soc Trop Med Hyg* 1995; **89**(3): 296-8.

90. Karbwang J, Na-Bangchang K, Congpuong K, Thanavibul A, Wattanakoon Y, Molunto P. Pharmacokinetics of oral artemether in Thai patients with uncomplicated falciparum malaria. *Fundam Clin Pharmacol* 1998; **12**(2): 242-4.

91. Karbwang J, Na-Bangchang K, Thanavibul A, Mull R, Gathmann I. Dose-finding study of the efficacy of fixed-combination artemether/lumefantrine for the treatment of multidrug-resistant *Plasmodium falciparum* malaria in Thailand. *Clin Drug Inv* 2000; **19**(5): 343-8.

26. Karunajeewa HA, Mueller I, Senn M, et al. A trial of combination antimalarial therapies in children from Papua New Guinea. *N Engl J Med* 2008; **359**(24): 2545-57.

92. Karunajeewa HA, Ilett KF, Mueller I, et al. Pharmacokinetics and efficacy of piperaquine and chloroquine in Melanesian children with uncomplicated malaria. *Antimicrob Agents Chemother* 2008; **52**(1): 237-43.

93. Kinzer MH, Chand K, Basri H, et al. Active case detection, treatment of falciparum malaria with combined chloroquine and sulphadoxine/pyrimethamine and vivax malaria with chloroquine and molecular markers of anti-malarial resistance in the Republic of Vanuatu. *Malar J* 2010; **9**: 89.

94. Kremsner PG, Zotter GM, Graninger W, Rocha RM, Bienzle U, Feldmeier H. Clindamycin is effective against *Plasmodium falciparum* but not against *P. vivax* in mixed infections. *Trans R Soc Trop Med Hyg* 1989; **83**(3): 332-3.

95. Krudsood S, Singhasivanon P, Silachamroon U, et al. Clinical trial of halofantrine with modified doses for treatment of malaria in the hospital for tropical diseases. *Southeast Asian J Trop Med Public Health* 2001; **32**(2): 255-61.

96. Krudsood S, Looareesuwan S, Silachamroon U, et al. Artesunate and mefloquine given simultaneously for three days via a prepacked blister is equally effective and tolerated as a standard sequential treatment of uncomplicated acute *Plasmodium falciparum* malaria: randomized, double-blind study in Thailand. *Am J Trop Med Hyg* 2002; **67**(5): 465-72.

97. Krudsood S, Chalermrut K, Pengruksa C, et al. Comparative clinical trial of two-fixed combinations dihydroartemisinin-napthoquine-trimethoprim (DNP) and artemether-lumefantrine (Coartem/Riamet) in the treatment of acute uncomplicated falciparum malaria in Thailand. *Southeast Asian J Trop Med Public Health* 2003; **34**(2): 316-21.

98. Kshirsagar NA, Gogtay NJ, Moorthy NS, et al. A randomized, double-blind, parallel-group, comparative safety, and efficacy trial of oral co-artemether versus oral chloroquine in the treatment of acute uncomplicated *Plasmodium falciparum* malaria in adults in India. *Am J Trop Med Hyg* 2000; **62**(3): 402-8.

99. Kyaw MP, Nyunt MH, Chit K, et al. Reduced susceptibility of *Plasmodium falciparum* to artesunate in southern Myanmar. *PLoS One* 2013; **8**(3): e57689.

100. Ladeia-Andrade S, de Melo GN, de Souza-Lima Rde C, et al. No Clinical or Molecular Evidence of *Plasmodium falciparum* Resistance to Artesunate-Mefloquine in Northwestern Brazil. *Am J Trop Med Hyg* 2016; **95**(1): 148-54.

101. Laman M, Moore BR, Benjamin JM, et al. Artemisinin-naphthoquine versus artemether-lumefantrine for uncomplicated malaria in Papua New Guinean children: an open-label randomized trial. *PLoS Med* 2014; **11**(12): e1001773.

102. Leang R, Barrette A, Bouth DM, et al. Efficacy of dihydroartemisinin-piperaquine for treatment of uncomplicated *Plasmodium falciparum* and *Plasmodium vivax* in Cambodia, 2008 to 2010. *Antimicrob Agents Chemother* 2013; **57**(2): 818-26.

103. Leang R, Canavati SE, Khim N, et al. Efficacy and Safety of Pyronaridine-Artesunate for Treatment of Uncomplicated *Plasmodium falciparum* Malaria in Western Cambodia. *Antimicrob Agents Chemother* 2016; **60**(7): 3884-90.

104. Lederman ER, Maguire JD, Sumawinata IW, et al. Combined chloroquine, sulfadoxine/pyrimethamine and primaquine against *Plasmodium falciparum* in Central Java, Indonesia. *Malar J* 2006; **5**: 108.

105. Lefevre G, Looareesuwan S, Treeprasertsuk S, et al. A clinical and pharmacokinetic trial of six doses of artemether-lumefantrine for multidrug-resistant *Plasmodium falciparum* malaria in Thailand. *Am J Trop Med Hyg* 2001; **64**(5-6): 247-56.

32. Li GQ, Arnold K, Guo XB, Jian HX, Fu LC. Randomised comparative study of mefloquine, qinghaosu, and pyrimethamine-sulfadoxine in patients with falciparum malaria. *Lancet* 1984; **2**(8416): 1360-1.

106. Lon C, Manning JE, Vanachayangkul P, et al. Efficacy of two versus three-day regimens of dihydroartemisinin-piperaquine for uncomplicated malaria in military personnel in northern Cambodia: an open-label randomized trial. *PLoS One* 2014; **9**(3): e93138.

107. Looareesuwan S, Viravan C, Vanijanonta S, et al. Randomized trial of mefloquine-doxycycline, and artesunate-doxycycline for treatment of acute uncomplicated falciparum malaria. *Am J Trop Med Hyg* 1994; **50**(6): 784-9.

108. Looareesuwan S, Wilairatana P, Vanijanonta S, Pitisuttithum P, Viravan C, Kraisintu K. Treatment of acute, uncomplicated, falciparum malaria with oral dihydroartemisinin. *Ann Trop Med Parasitol* 1996; **90**(1): 21-8.

109. Looareesuwan S, Viravan C, Webster HK, Kyle DE, Hutchinson DB, Canfield CJ. Clinical studies of atovaquone, alone or in combination with other antimalarial drugs, for treatment of acute uncomplicated malaria in Thailand. *Am J Trop Med Hyg* 1996; **54**(1): 62-6.

110. Looareesuwan S, Kyle DE, Viravan C, Vanijanonta S, Wilairatana P, Wernsdorfer WH. Clinical study of pyronaridine for the treatment of acute uncomplicated falciparum malaria in Thailand. *Am J Trop Med Hyg* 1996; **54**(2): 205-9.

111. Looareesuwan S, Wilairatana P, Viravan C, Vanijanonta S, Pitisuttithum P, Kyle DE. Open randomized trial of oral artemether alone and a sequential combination with mefloquine for acute uncomplicated falciparum malaria. *Am J Trop Med Hyg* 1997; **56**(6): 613-7.

112. Looareesuwan S, Wilairatana P, Vanijanonta S, Pitisuttithum P, Ratanapong Y, Andrial M. Monotherapy with sodium artesunate for uncomplicated falciparum malaria in Thailand: a comparison of 5- and 7-day regimens. *Acta Trop* 1997; **67**(3): 197-205.

113. Looareesuwan S, Wilairatana P, Chalermarut K, Rattanapong Y, Canfield CJ, Hutchinson DB. Efficacy and safety of atovaquone/proguanil compared with mefloquine for treatment of acute *Plasmodium falciparum* malaria in Thailand. *Am J Trop Med Hyg* 1999; **60**(4): 526-32.

114. Maguire JD, Lacy MD, Sururi, et al. Chloroquine or sulfadoxine-pyrimethamine for the treatment of uncomplicated, *Plasmodium falciparum* malaria during an epidemic in Central Java, Indonesia. *Ann Trop Med Parasitol* 2002; **96**(7): 655-68.

115. Maguire JD, Krisin, Marwoto H, Richie TL, Fryauff DJ, Baird JK. Mefloquine is highly efficacious against chloroquine-resistant *Plasmodium vivax* malaria and *Plasmodium falciparum* malaria in Papua, Indonesia. *Clin Infect Dis* 2006; **42**(8): 1067-72.

116. Marquino W, Ylquimiche L, Hermenegildo Y, et al. Efficacy and tolerability of artesunate plus sulfadoxine-pyrimethamine and sulfadoxine-pyrimethamine alone for the treatment of uncomplicated *Plasmodium falciparum* malaria in Peru. *Am J Trop Med Hyg* 2005; **72**(5): 568-72.

117. Mayxay M, Phetsouvanh R, Phompida S, et al. A randomized comparison of oral chloroquine and sulfadoxine-pyrimethamine for the treatment of uncomplicated *Plasmodium falciparum* malaria in Laos. *Trans R Soc Trop Med Hyg* 2003; **97**(3): 343-4.

118. Mayxay M, Khanthavong M, Lindegardh N, et al. Randomized comparison of chloroquine plus sulfadoxine-pyrimethamine versus artesunate plus mefloquine versus artemether-lumefantrine in the treatment of uncomplicated falciparum malaria in the Lao People's Democratic Republic. *Clin Infect Dis* 2004; **39**(8): 1139-47.

119. Mayxay M, Thongpraseuth V, Khanthavong M, et al. An open, randomized comparison of artesunate plus mefloquine vs. dihydroartemisinin-piperaquine for the treatment of uncomplicated *Plasmodium falciparum* malaria in the Lao People's Democratic Republic (Laos). *Trop Med Int Health* 2006; **11**(8): 1157-65.

120. Mayxay M, Keomany S, Khanthavong M, et al. A phase III, randomized, non-inferiority trial to assess the efficacy and safety of dihydroartemisinin-piperaquine in comparison with artesunate-mefloquine in patients with uncomplicated *Plasmodium falciparum* malaria in southern Laos. *Am J Trop Med Hyg* 2010; **83**(6): 1221-9.

121. Mayxay M, Khanthavong M, Chanthongthip O, et al. No evidence for spread of *Plasmodium falciparum* artemisinin resistance to Savannakhet Province, Southern Laos. *Am J Trop Med Hyg* 2012; **86**(3): 403-8.

122. Mayxay M, Khanthavong M, Chanthongthip O, et al. Efficacy of artemether-lumefantrine, the nationally-recommended artemisinin combination for the treatment of uncomplicated falciparum malaria, in southern Laos. *Malar J* 2012; **11**: 184.

123. Meek SR, Doberstyn EB, Gauzere BA, Thanapanich C, Nordlander E, Phuphaisan S. Treatment of falciparum malaria with quinne and tetracycline or combined mefloquine/sulfadoxine/pyrimethamine on the Thai-Kampuchean border. *Am J Trop Med Hyg* 1986; **35**(2): 246-50.

124. Mekonnen SK, Medhin G, Berhe N, Clouse RM, Aseffa A. Efficacy of artemether-lumefantrine therapy for the treatment of uncomplicated *Plasmodium falciparum* malaria in Southwestern Ethiopia. *Malar J* 2015; **14**: 317.

125. Miller RS, Wongsrichanalai C, Buathong N, et al. Effective treatment of uncomplicated *Plasmodium falciparum* malaria with azithromycin-quinine combinations: a randomized, dose-ranging study. *Am J Trop Med Hyg* 2006; **74**(3): 401-6.

126. Mishra N, Singh JP, Srivastava B, et al. Monitoring antimalarial drug resistance in India via sentinel sites: outcomes and risk factors for treatment failure, 2009-2010. *Bull World Health Organ* 2012; **90**(12): 895-904.

127. Mishra N, Kaitholia K, Srivastava B, et al. Declining efficacy of artesunate plus sulphadoxine-pyrimethamine in northeastern India. *Malar J* 2014; **13**: 284.

128. Mishra N, Srivastava B, Bharti RS, et al. Monitoring the efficacy of antimalarial medicines in India via sentinel sites: Outcomes and risk factors for treatment failure. *J Vector Borne Dis* 2016; **53**(2): 168-78.

129. Mohapatra PK, Khan AM, Prakash A, Mahanta J, Srivastava VK. Effect of arteether alpha/beta on uncomplicated falciparum malaria cases in Upper Assam. *Indian J Med Res* 1996; **104**: 284-7.

130. Na Bangchang K, Karbwang J, Thomas CG, et al. Pharmacokinetics of artemether after oral administration to healthy Thai males and patients with acute, uncomplicated falciparum malaria. *Br J Clin Pharmacol* 1994; **37**(3): 249-53.

131. Na-Bangchang K, Tipwangso P, Thanavibul A, et al. Artemether-pyrimethamine in the treatment of pyrimethamine-resistant falciparum malaria. *Southeast Asian J Trop Med Public Health* 1996; **27**(1): 19-23.

132. Na-Bangchang K, Kanda T, Tipawangso P, et al. Activity of artemether-azithromycin versus artemether-doxycycline in the treatment of multiple drug resistant falciparum malaria. *Southeast Asian J Trop Med Public Health* 1996; **27**(3): 522-5.

133. Na-Bangchang K, Congpuong K, Sirichaisinthop J, Suprakorb K, Karbwang J. Compliance with a 2 day course of artemether-mefloquine in an area of highly multi-drug resistant *Plasmodium falciparum* malaria. *Br J Clin Pharmacol* 1997; **43**(6): 639-42.

134. Na-Bangchang K, Tippanangkosol P, Ubalee R, Chaovanakawee S, Saenglertsilapachai S, Karbwang J. Comparative clinical trial of four regimens of dihydroartemisinin-mefloquine in multidrug-resistant falciparum malaria. *Trop Med Int Health* 1999; **4**(9): 602-10.

135. Na-Bangchang K, Ruengweerayut R, Mahamad P, Ruengweerayut K, Chaijaroenkul W. Declining in efficacy of a three-day combination regimen of mefloquine-artesunate in a multi-drug resistance area along the Thai-Myanmar border. *Malar J* 2010; **9**: 273.

136. Nguyen MH, Davis TM, Cox-Singh J, et al. Treatment of uncomplicated falciparum malaria in southern Vietnam: can chloroquine or sulfadoxine-pyrimethamine be reintroduced in combination with artesunate? *Clin Infect Dis* 2003; **37**(11): 1461-6.

137. Noedl H, Se Y, Sriwichai S, et al. Artemisinin resistance in Cambodia: a clinical trial designed to address an emerging problem in Southeast Asia. *Clin Infect Dis* 2010; **51**(11): e82-9.

138. Nosten F, Imvithaya S, Vincenti M, et al. Malaria on the Thai-Burmese border: treatment of 5192 patients with mefloquine-sulfadoxine-pyrimethamine. *Bull World Health Organ* 1987; **65**(6): 891-6.

139. Oemijati S, Pribadi W, Wartati K, Arbani P, Suprijanto S, Rasisi R. Treatment of chloroquine-resistant *Plasmodium falciparum* infections with clindamycin hydrochloride in Dili, East Timor, Indonesia. *Curr Ther Res Clin Exp* 1994; **55**(4): 468-79.

140. Pe Than M, Tin S. The efficacy of artemether (qinghaosu) in *Plasmodium falciparum* and *P. vivax* in Burma. *Southeast Asian J Trop Med Public Health* 1986; **17**(1): 19-22.

141. Phong NC, Quang HH, Thanh NX, et al. In Vivo Efficacy and Tolerability of Artesunate-Azithromycin for the Treatment of Falciparum Malaria in Vietnam. *Am J Trop Med Hyg* 2016; **95**(1): 164-7.

142. Poespoprodjo JR, Kenangalem E, Wafom J, et al. Therapeutic Response to Dihydroartemisinin-Piperaquine for *P. falciparum* and *P. vivax* Nine Years after Its Introduction in Southern Papua, Indonesia. *Am J Trop Med Hyg* 2018; **98**(3): 677-82.

143. Pukrittayakamee S, Chantra A, Vanijanonta S, Clemens R, Looareesuwan S, White NJ. Therapeutic responses to quinine and clindamycin in multidrug-resistant falciparum malaria. *Antimicrob Agents Chemother* 2000; **44**(9): 2395-8.

144. Pukrittayakamee S, Chotivanich K, Chantra A, Clemens R, Looareesuwan S, White NJ. Activities of artesunate and primaquine against asexual- and sexual-stage parasites in falciparum malaria. *Antimicrob Agents Chemother* 2004; **48**(4): 1329-34.

145. Rahman R, Martin MJ, Persaud S, et al. Continued Sensitivity of *Plasmodium falciparum* to Artemisinin in Guyana, With Absence of Kelch Propeller Domain Mutant Alleles. *Open Forum Infect Dis* 2016; **3**(3): ofw185.

146. Ratcliff A, Siswantoro H, Kenangalem E, et al. Therapeutic response of multidrug-resistant *Plasmodium falciparum* and *P. vivax* to chloroquine and sulfadoxine-pyrimethamine in southern Papua, Indonesia. *Trans R Soc Trop Med Hyg* 2007; **101**(4): 351-9.

147. Restrepo M, Botero D, Marquez RE, Boudreau EF, Navaratnam V. A clinical trial with halofantrine on patients with falciparum malaria in Colombia. *Bull World Health Organ* 1996; **74**(6): 591-7.

148. Rogers WO, Sem R, Tero T, et al. Failure of artesunate-mefloquine combination therapy for uncomplicated *Plasmodium falciparum* malaria in southern Cambodia. *Malar J* 2009; **8**: 10.

149. Sabchareon A, Attanath P, Phanuaksook P, et al. Efficacy and pharmacokinetics of atovaquone and proguanil in children with multidrug-resistant *Plasmodium falciparum* malaria. *Trans R Soc Trop Med Hyg* 1998; **92**(2): 201-6.

150. Salazar NP, Saniel MC, Estoque MH, et al. Oral clindamycin in the treatment of acute uncomplicated falciparum malaria. *Southeast Asian J Trop Med Public Health* 1990; **21**(3): 397-403.

151. Saunders DL, Vanachayangkul P, Lon C, et al. Dihydroartemisinin-piperaquine failure in Cambodia. *N Engl J Med* 2014; **371**(5): 484-5.

152. Segal HE, Chinvanthananond P, Laixuthai B, et al. Preliminary study of WR 33063 in the treatment of falciparum malaria in northeast Thailand. *Am J Trop Med Hyg* 1974; **23**(4): 560-4.

153. Segal HE, Chinvanthananond P, Laixuthai B, et al. Comparison of diaminodiphenylsulphonepyrimethamine and sulfadoxine-pyrimethamine combinations in the treatment of falciparum malaria in Thailand. *Trans R Soc Trop Med Hyg* 1975; **69**(1): 139-42.

154. Smithuis F, van der Broek I, Katterman N, et al. Optimising operational use of artesunate-mefloquine: a randomised comparison of four treatment regimens. *Trans R Soc Trop Med Hyg* 2004; **98**(3): 182-92.

155. Smithuis F, Shahmanesh M, Kyaw MK, Savran O, Lwin S, White NJ. Comparison of chloroquine, sulfadoxine/pyrimethamine, mefloquine and mefloquine-artesunate for the treatment of falciparum malaria in Kachin State, North Myanmar. *Trop Med Int Health* 2004; **9**(11): 1184-90.

156. Smithuis F, Kyaw MK, Phe O, et al. Efficacy and effectiveness of dihydroartemisinin-piperaquine versus artesunate-mefloquine in falciparum malaria: an open-label randomised comparison. *Lancet* 2006; **367**(9528): 2075-85.

28. Smithuis F, Kyaw MK, Phe O, et al. Effectiveness of five artemisinin combination regimens with or without primaquine in uncomplicated falciparum malaria: an open-label randomised trial. *Lancet Infect Dis* 2010; **10**(10): 673-81.

157. Spring MD, Lin JT, Manning JE, et al. Dihydroartemisinin-piperaquine failure associated with a triple mutant including *kelch13* C580Y in Cambodia: an observational cohort study. *Lancet Infect Dis* 2015; **15**(6): 683-91.

158. Srivastava P, Ratha J, Shah NK, et al. A clinical and molecular study of artesunate + sulphadoxine-pyrimethamine in three districts of central and eastern India. *Malar J* 2013; **12**: 247.

159. Starzengruber P, Swoboda P, Fuehrer HP, et al. Current status of artemisinin-resistant falciparum malaria in South Asia: a randomized controlled artesunate monotherapy trial in Bangladesh. *PLoS One* 2012; **7**(12): e52236.

160. Stohrer JM, Dittrich S, Thongpaseuth V, et al. Therapeutic efficacy of artemether-lumefantrine and artesunate-mefloquine for treatment of uncomplicated *Plasmodium falciparum* malaria in Luang Namtha Province, Lao People's Democratic Republic. *Trop Med Int Health* 2004; **9**(11): 1175-83.

161. Sumawinata IW, Bernadeta, Leksana B, et al. Very high risk of therapeutic failure with chloroquine for uncomplicated *Plasmodium falciparum* and *P. vivax* malaria in Indonesian Papua. *Am J Trop Med Hyg* 2003; **68**(4): 416-20.

162. Suputtamongkol Y, Chindarat S, Silpasakorn S, et al. The efficacy of combined mefloquine-artesunate versus mefloquine-primaquine on subsequent development of *Plasmodium falciparum* gametocytemia. *Am J Trop Med Hyg* 2003; **68**(5): 620-3.

163. Sutanto I, Supriyanto S, Ruckert P, Purnomo, Maguire JD, Bangs MJ. Comparative efficacy of chloroquine and sulfadoxine-pyrimethamine for uncomplicated *Plasmodium falciparum* malaria and impact on gametocyte carriage rates in the East Nusatenggara province of Indonesia. *Am J Trop Med Hyg* 2004; **70**(5): 467-73.

164. Sutanto I, Endawati D, Ling LH, Laihad F, Setiabudy R, Baird JK. Evaluation of chloroquine therapy for vivax and falciparum malaria in southern Sumatra, western Indonesia. *Malar J* 2010; **9**: 52.

165. Tangpukdee N, Krudsood S, Thanachartwet V, et al. Efficacy of Artequick versus artesunate-mefloquine in the treatment of acute uncomplicated falciparum malaria in Thailand. *Southeast Asian J Trop Med Public Health* 2008; **39**(1): 1-8.

166. Teklemariam M, Assefa A, Kassa M, Mohammed H, Mamo H. Therapeutic efficacy of artemether-lumefantrine against uncomplicated *Plasmodium falciparum* malaria in a high-transmission area in northwest Ethiopia. *PLoS One* 2017; **12**(4): e0176004.

167. Thanh NX, Trung TN, Phong NC, et al. The efficacy and tolerability of artemisinin-piperaquine (Artequick(R)) versus artesunate-amodiaquine (Coarsucam) for the treatment of uncomplicated *Plasmodium falciparum* malaria in south-central Vietnam. *Malar J* 2012; **11**: 217.

168. Thapa S, Hollander J, Linehan M, et al. Comparison of artemether-lumefantrine with sulfadoxine-pyrimethamine for the treatment of uncomplicated falciparum malaria in eastern Nepal. *Am J Trop Med Hyg* 2007; **77**(3): 423-30.

169. Thimasarn K, Sirichaisinthop J, Chanyakhun P, Palananth C, Rooney W. A comparative study of artesunate and artemether in combination with mefloquine on multidrug resistant falciparum malaria in eastern Thailand. *Southeast Asian J Trop Med Public Health* 1997; **28**(3): 465-71.

170. Thriemer K, Haque R, Wagatsuma Y, et al. Therapeutic efficacy of quinine plus sulfadoxine-pyremethamine for the treatment of uncomplicated falciparum malaria in Bangladesh. *Am J Trop Med Hyg* 2006; **75**(4): 645-9.

171. Thriemer K, Starzengruber P, Khan WA, et al. Azithromycin combination therapy for the treatment of uncomplicated falciparum malaria in Bangladesh: an open-label randomized, controlled clinical trial. *J Infect Dis* 2010; **202**(3): 392-8.

172. Tjitra E, Suprianto S, Currie BJ, Morris PS, Saunders JR, Anstey NM. Therapy of uncomplicated falciparum malaria: a randomized trial comparing artesunate plus sulfadoxine-pyrimethamine versus sulfadoxine-pyrimethamine alone in Irian Jaya, Indonesia. *Am J Trop Med Hyg* 2001; **65**(4): 309-17.

173. Tun T, Tint HS, Lin K, et al. Efficacy of oral single dose therapy with artemisinin-naphthoquine phosphate in uncomplicated falciparum malaria. *Acta Trop* 2009; **111**(3): 275-8.

174. Valecha N, Krudsood S, Tangpukdee N, et al. Arterolane maleate plus piperaquine phosphate for treatment of uncomplicated *Plasmodium falciparum* malaria: a comparative, multicenter, randomized clinical trial. *Clin Infect Dis* 2012; **55**(5): 663-71.

175. Valecha N, Srivastava B, Dubhashi NG, et al. Safety, efficacy and population pharmacokinetics of fixed-dose combination of artesunate-mefloquine in the treatment of acute uncomplicated *Plasmodium falciparum* malaria in India. *J Vector Borne Dis* 2013; **50**(4): 258-64.

176. van den Broek IV, Maung UA, Peters A, et al. Efficacy of chloroquine + sulfadoxine--pyrimethamine, mefloquine + artesunate and artemether + lumefantrine combination therapies to treat *Plasmodium falciparum* malaria in the Chittagong Hill Tracts, Bangladesh. *Trans R Soc Trop Med Hyg* 2005; **99**(10): 727-35.

177. van Vugt M, Brockman A, Gemperli B, et al. Randomized comparison of artemether-benflumetol and artesunate-mefloquine in treatment of multidrug-resistant falciparum malaria. *Antimicrob Agents Chemother* 1998; **42**(1): 135-9.

178. Vugt MV, Wilairatana P, Gemperli B, et al. Efficacy of six doses of artemether-lumefantrine (benflumetol) in multidrug-resistant *Plasmodium falciparum* malaria. *Am J Trop Med Hyg* 1999; **60**(6): 936-42.

179. van Vugt M, Leonardi E, Phaipun L, et al. Treatment of uncomplicated multidrug-resistant falciparum malaria with artesunate-atovaquone-proguanil. *Clin Infect Dis* 2002; **35**(12): 1498-504.

180. Wilairatana P, Krudsood S, Chokejindachai W, et al. A clinical trial of combination of artesunate and mefloquine in the treatment of acute uncomplicated falciparum malaria: a short and practical regimen. *Southeast Asian J Trop Med Public Health* 1998; **29**(4): 696-701.

181. Wilairatana P, Krudsood S, Chalermrut K, et al. An open randomized clinical trial of Artecom vs artesunate-mefloquine in the treatment of acute uncomplicated falciparum malaria in Thailand. *Southeast Asian J Trop Med Public Health* 2002; **33**(3): 519-24.

182. Wudneh F, Assefa A, Nega D, et al. Open-label trial on efficacy of artemether/lumefantrine against the uncomplicated *Plasmodium falciparum* malaria in Metema district, Northwestern Ethiopia. *Ther Clin Risk Manag* 2016; **12**: 1293-300.

183. Yeramian P, Meshnick SR, Krudsood S, et al. Efficacy of DB289 in Thai patients with *Plasmodium vivax* or acute, uncomplicated *Plasmodium falciparum* infections. *J Infect Dis* 2005; **192**(2): 319-22.

Appendix 7.

Table S5. Studies excluded from meta-analysis

Reason for exclusion	Number of studies	References*
Countries with unstable *P. vivax* transmission†	426	
No mention of *P. vivax*	420	[184-603]
Studies reported previously	3	[604-606]
Only included pregnant women	1	[607]
Severe malaria only	1	[608]
Imported malaria cases	1	[609]
Countries with stable *P. vivax* transmission‡	312	
No mention of *P. vivax*	272	[610-881]
Unable to extract all recurrence data for fixed follow up	14	[6,882-894]
Only included pregnant women	6	[895-900]
Studies reported previously	5	[901-905]
Unclear whether *P. vivax* infection at or after baseline	4	[906-909]
Some patients not from co-endemic countries‡	4	[910-913]
Part of full text missing	2	[914,915]
Complicated malaria only	1	[916]
Only able to extract day 56 follow up data§	1	[917]
Other¶	3	[918-920]

* Excluded studies are listed in Appendix, page 60; † As defined by the Malaria Atlas Project[9]; ‡ Countries that reported or were suspected to have indigenous *P. falciparum* and *P. vivax* cases in 2016 according to the World Health Organization's World Malaria Report 2017[1]; § Two treatment arms in Li *et al*[32] did not report outcomes and these arms were excluded; ¶ Two studies were phase 1 trials and enrolled mainly healthy adults and one study only enrolled patients with *P. vivax* asexual parasites +/- *P. falciparum* gametocytes.

Table S6. Studies included in meta-analysis

Author - Year	Country	Region	Years enrolled	Randomised	Blinded	Ages enrolled (years)			Mixed infections	Follow up (days)	Enrolled*	Treatment arms	Sites
						<5	5-15	>15					
Segal - 1974[152]	Thailand	Asia-Pacific	1972	Yes	No	No	No	Yes	No	28	51	2	1
Hall - 1975[79]	Thailand	Asia-Pacific	1973-1974	No	No	No	Yes	Yes	No	28	47	3	1
Segal - 1975[153]	Thailand	Asia-Pacific	1972-1973	Yes	No	No	No	Yes	No	28	44	2	1
Doberstyn - 1979[64]	Thailand	Asia-Pacific	1976	No	No	No	No	Yes	No	60	75	2	1
de Souza - 1983[59]	Brazil	The Americas		Yes	Yes	No	No	Yes	No	63	99	2	1
Harinasuta - 1983[76]	Thailand	Asia-Pacific	1980-1981	Yes	Yes	No	No	Yes	No	63	147	3	1
Li - 1984[32]	China	Asia-Pacific	1982-1984	Yes	No	No	Yes	Yes	No	28	90	6	1
Botero - 1985[45]	Colombia	The Americas	1982-1983	Yes	Yes	No	No	Yes	No	28	75	3	1
de Souza - 1985[60]	Brazil	The Americas		Yes	No	No	No	Yes	No	42	100	2	1
Harinasuta - 1985[77]	Thailand	Asia-Pacific	1981-1982	Yes	Yes	No	Yes	Yes	No	42	40	2	1
Meek - 1986[123]	Thailand	Asia-Pacific	1983	No	No	No	No	Yes	No	56	150	3	1
Pe - 1986[140]	Myanmar	Asia-Pacific		No	No	No	Yes	Yes	Yes	28	25	1	3
Chongsuphajaisiddhi - 1987[57]	Thailand	Asia-Pacific	1982-1984	No	No	No	Yes	No	No	42	84	1	1
Harinasuta - 1987[78]	Thailand	Asia-Pacific	1983-1984	Yes	Yes	No	No	Yes	No	42	94	2	1
Nosten - 1987[138]	Thailand	Asia-Pacific	1986	No	No	Yes	Yes	Yes	Yes	28	1707	1	1
Boudreau - 1988[46]	Thailand	Asia-Pacific	1982-1983	Yes	Yes	No	No	Yes	No	28	147	4	1
Kremsner - 1989[94]	Brazil	The Americas	1987	No	No	No	No	Yes	Yes	28	10	1	1
Salazar - 1990[150]	Philippines	Asia-Pacific	1984-1985	Yes	No	No	Yes	Yes	No	28	48	3	2
Bunnag - 1991a[47]	Thailand	Asia-Pacific		Yes	Yes	No	No	Yes	No	28	69	2	1
Bunnag - 1991b[48]	Thailand	Asia-Pacific		Yes	Yes	No	Yes	Yes	No	28	89	2	1
Bunnag - 1991c[49]	Thailand	Asia-Pacific	1989	Yes	No	No	No	Yes	No	28	68	9	1
Bunnag - 1992a[50]	Thailand	Asia-Pacific		Yes	No	No	No	Yes	No	35	51	2	1
Bunnag - 1992b[51†]	Thailand	Asia-Pacific	1989-1990	Yes	No	No	No	Yes	No	28	61	2	1
Karbwang - 1992[86]	Thailand	Asia-Pacific		Yes	No	No	No	Yes	No	28	46	2	1
Karbwang - 1994[87]	Thailand	Asia-Pacific		Yes	No	No	No	Yes	No	28	64	2	1
Looareesuwan - 1994[107]	Thailand	Asia-Pacific	1992	Yes	No	No	No	Yes	No	28	109	2	1
Na Bangchang - 1994[130]	Thailand	Asia-Pacific		No	No	No	No	Yes	No	28	8	1	1
Oemijati - 1994[139]	Indonesia	Asia-Pacific	1988-1989	No	No	No	No	Yes	No	28	46	2	1
Bunnag - 1995[52]	Thailand	Asia-Pacific	1992-1994	Yes	Yes	No	No	Yes	No	42	159	2	1
Karbwang - 1995a[88]	Thailand	Asia-Pacific	1993	Yes	No	No	Yes	Yes	No	42‡	109	2	1
Karbwang - 1995b[89]	Thailand	Asia-Pacific		Yes	No	No	No	Yes	No	42	57	2	1
Baird - 1996[39]	Indonesia	Asia-Pacific	1994	No	No	Yes	Yes	Yes	No	28	11	1	1

Bunnag - 1996a[53]	Thailand	Asia-Pacific	1990-1992	Yes	No	No	No	Yes	No	28	90	2	1
Bunnag - 1996b[54]	Thailand	Asia-Pacific	1992-1994	Yes	No	No	No	Yes	No	42	120	2	1
Duarte - 1996[66]	Brazil	The Americas	1992-1993	Yes	Yes	No	Yes	Yes	No	28	176	2	1
Looareesuwan - 1996a[108]	Thailand	Asia-Pacific	1994-1995	No	No	No	No	Yes	No	28	53	1	1
Looareesuwan - 1996b[109]	Thailand	Asia-Pacific	1990-1993	No	No	No	No	Yes	No	28	292	13	1
Looareesuwan - 1996c[110]	Thailand	Asia-Pacific	1992	No	No	No	No	Yes	No	28	101	2	1
Mohapatra - 1996[129]	India	Asia-Pacific	1995	No	No	No	No	Yes	No	28	37	1	1
Na Bangchang - 1996a[131]	Thailand	Asia-Pacific	1995	Yes	No	No	No	Yes	No	28	60	3	1
Na Bangchang - 1996b[132]	Thailand	Asia-Pacific	1995	Yes	No	No	No	Yes	No	28	60	2	1
Restrepo - 1996[147]	Colombia	The Americas		Yes	Yes	No	Yes	Yes	No	42	120	5	1
Bunnag - 1997[55]	Thailand	Asia-Pacific	1996	Yes	No	No	No	Yes	No	42	99	2	1
de Alencar - 1997[58]	Brazil	The Americas	1995-1996	Yes	No	No	No	Yes	No	28	154	2	1
Jiao - 1997[85]	China	Asia-Pacific		No	No	No	Yes	Yes	No	28	102	1	1
Looareesuwan - 1997a[111]	Thailand	Asia-Pacific	1992	Yes	No	No	No	Yes	No	28	151	3	1
Looareesuwan - 1997b[112]	Thailand	Asia-Pacific	1995	Yes	No	No	No	Yes	No	28	91	2	1
Na Bangchang - 1997[133]	Thailand	Asia-Pacific	1994-1995	No	No	No	No	Yes	No	42	126	1	1
Thimarsarn - 1997[169]	Thailand	Asia-Pacific	1993-1994	Yes	No	No	No	Yes	No	28	394	7	2
Karbwang - 1998[90]	Thailand	Asia-Pacific		No	No	No	No	Yes	No	28	13	1	1
Sabchareon - 1998[149]	Thailand	Asia-Pacific	1994-1995	No	No	Yes	Yes	No	No	28	32	1	1
van Vugt - 1998[177]	Thailand	Asia-Pacific	1995-1996	Yes	No	Yes	Yes	Yes	No	63	617	2	1
Wilairatana - 1998[180]	Thailand	Asia-Pacific	1997-1998	No	No	No	No	Yes	No	28	150	1	1
Cerutti - 1999[56]	Brazil	The Americas	1996-1997	No	No	No	No	Yes	No	28	109	1	1
Looareesuwan - 1999[113]	Thailand	Asia-Pacific	1993-1994	Yes	No	No	No	Yes	No	28	182	2	1
Na Bangchang - 1999[134]	Thailand	Asia-Pacific	1996-1998	Yes	No	No	No	Yes	No	42	207	4	1
van Vugt - 1999[178]	Thailand	Asia-Pacific	1996-1997	Yes	Yes	Yes	Yes	Yes	No	28	359	3	2
Karbwang - 2000[91]	Thailand	Asia-Pacific		Yes	Yes	No	Yes	Yes	No	28	260	3	2
Kshirsagar - 2000[98]	Cambodia	Asia-Pacific	1996-1997	Yes	Yes	No	No	Yes	No	29	179	2	1
Pukrittayakamee - 2000[143]	Thailand	Asia-Pacific	1995-1997	Yes	No	No	No	Yes	No	28	204	3	1
Krudsood - 2001[95]	Thailand	Asia-Pacific	1992-1993	Yes	No	No	No	Yes	No	28	120	3	1
Lefevre - 2001[105]	Thailand	Asia-Pacific	1998-1999	Yes	No	No	Yes	Yes	Yes	28	219	2	1

Tjitra - 2001[172]	Indonesia	Asia-Pacific	1999	Yes	No	Yes	Yes	Yes	No	28	105	2	1
Baird - 2002[40]	Guyana	The Americas	1998	No	No	No	Yes	Yes	Yes	28	33	1	1
Denis - 2002[61]	Cambodia	Asia-Pacific	2001-2002	No	No	No	No	No	No	28	106	1	2
Fontes - 2002[71]	Brazil	The Americas		No	No	No	Yes	Yes	No	28	31	1	2
Fryauff - 2002[72]	Indonesia	Asia-Pacific	1998	No	No	Yes	Yes	Yes	Yes	28	39	1	5
Krudsood - 2002[96]	Thailand	Asia-Pacific	2001	Yes	Yes	No	Yes	Yes	Yes	28	204	2	1
Maguire - 2002[114]	Indonesia	Asia-Pacific		No	No	Yes	Yes	Yes	Yes	28	167	2	4
van Vugt - 2002[179]	Thailand	Asia-Pacific	1998-2000	Yes	No	Yes	Yes	Yes	No	42	1596	3	1
Wilairatana - 2002[181]	Thailand	Asia-Pacific		Yes	No	No	Yes	Yes	No	28	352	2	1
Ezard - 2003[70]	East Timor	Asia-Pacific	2000	No	No	Yes	Yes	Yes	No	28	48	1	1
Krudsood - 2003[97]	Thailand	Asia-Pacific		Yes	No	No	Yes	Yes	No	28	130	2	1
Mayxay - 2003[117]	Laos	Asia-Pacific	2000	Yes	No	No	Yes	Yes	No	42	29	2	1
Nguyen - 2003[136]	Vietnam	Asia-Pacific		Yes	No	No	No	No	No	28	123	2	2
Sumawinata - 2003[161]	Indonesia	Asia-Pacific	1995	No	No	Yes	Yes	Yes	Yes	28	75	1	1
Suputtamongkol - 2003[162]	Thailand	Asia-Pacific	1999-2001	Yes	No	No	Yes	Yes	No	42	556	2	7
Ashley - 2004[35]	Thailand	Asia-Pacific	2002-2003	Yes	No	Yes	Yes	Yes	Yes	63	731	4	2
Mayxay - 2004[118]	Laos	Asia-Pacific	2002-2003	Yes	No	No	No	No	No	42	330	3	1
Pukrittayakamee - 2004[144]	Thailand	Asia-Pacific		Yes	No	No	Yes	Yes	No	28	176	6	1
Smithuis - 2004a[154]	Myanmar	Asia-Pacific	2000-2001	Yes	Yes	No	No	No	No	42	803	4	2
Smithuis - 2004b[155]	Myanmar	Asia-Pacific	1998	Yes	No	Yes	Yes	Yes	No	42	317	4	1
Stohrer - 2004[160]	India	Asia-Pacific	2003	Yes	No	Yes	Yes	Yes	No	42	108	2	1
Sutanto - 2004[163]	Indonesia	Asia-Pacific	2001-2002	Yes	No	Yes	Yes	Yes	No	28	114	2	1
Ashley - 2005[36]	Thailand	Asia-Pacific	2003-2004	Yes	No	Yes	Yes	Yes	No	63	499	3	4
Durrani - 2005[67]	Afghanistan	Asia-Pacific	2002-2003	Yes	No	Yes	Yes	Yes	No	42	268	4	1
Genton - 2005[73]	Papua New Guinea	Asia-Pacific	1994-1995	No	No	Yes	Yes	Yes	No	28	154	2	1
Hutagalung - 2005[82]	Thailand	Asia-Pacific	2001-2002	Yes	No	Yes	Yes	Yes	No	42	490	2	1
Marquino - 2005[116]	Peru	The Americas	2000	Yes	No	No	Yes	Yes	No	28	197	2	1
van den Broek - 2005[176]	Thailand	Asia-Pacific	2003	Yes	No	Yes	Yes	Yes	No	42	364	3	1
Yeramian - 2005[183]	Thailand	Asia-Pacific	2003	No	No	No	No	Yes	No	28	23	1	1
Basano - 2006[41]	Brazil	The Americas	2000	No	No	Yes	Yes	Yes	No	42	50	1	1
Denis – 2006a[62]	Cambodia	Asia-Pacific	2001-2004	No	No	No	No	No	No	28	1025	1	9
Denis – 2006b[63]	Cambodia	Asia-Pacific	2003-2004	Yes	No	No	Yes	Yes	No	28	190	3	1
Lederman - 2006[104]	Indonesia	Asia-Pacific	2001	Yes	No	No	No	Yes	No	28	117	4	1
Maguire - 2006[115]	Indonesia	Asia-Pacific	1996-1999	Yes	No	No	Yes	Yes	Yes	28	491	2	1
Mayxay - 2006[119]	Laos	Asia-Pacific	2004	Yes	No	No	No	No	No	42	220	2	1
Miller - 2006[125]	Thailand	Asia-Pacific	1999-2001	Yes	No	No	No	Yes	No	28	61	4	1

Smithuis - 2006[156]	Myanmar	Asia-Pacific	2003-2004	Yes	No	No	No	No	No	42	652	4	1
Thriemer - 2006[170]	Bangladesh	Asia-Pacific	2004	No	No	No	No	Yes	No	42	63	1	1
Grande - 2007[74]	Peru	The Americas	2003-2005	Yes	No	No	No	No	No	63	522	2	1
Haque - 2007[75]	Bangladesh	Asia-Pacific	2005	No	No	No	No	No	No	42	67	1	2
Janssens - 2007[84]	Cambodia	Asia-Pacific	2002-2003	Yes	No	Yes	Yes	Yes	No	63	464	2	2
Ratcliff - 2007[146]	Indonesia	Asia-Pacific	2004	No	No	Yes	Yes	Yes	Yes	28	103	1	1
Thapa - 2007[168]	Nepal	Asia-Pacific	2005	Yes	No	No	No	No	No	28	102	2	1
Karunajeewa - 2008a[26]	Papua New Guinea	Asia-Pacific	2005-2007	Yes	No	Yes	No	No	No	42	482	4	2
Karunajeewa - 2008b[92]	Papua New Guinea	Asia-Pacific	2005-2006	Yes	No	No	Yes	No	No	42	35	2	1
Tangpukdee - 2008[165]	Thailand	Asia-Pacific	2006-2007	Yes	No	No	No	Yes	No	28	130	2	1
Dondorp - 2009[65]	Multicentred	Asia-Pacific	2007-2008	Yes	No	No	No	No	No	63	80	2	2
Rogers - 2009[148]	Cambodia	Asia-Pacific	2006-2008	No	No	Yes	Yes	Yes	No	42	150	1	1
Tun - 2009[173]	Myanmar	Asia-Pacific	2007	No	No	No	No	Yes	No	28	55	1	1
Assefa - 2010[37]	Ethiopia	Africa	2007-2008	No	No	Yes	Yes	Yes	No	28	90	1	1
Kinzer - 2010[93]	Vanuatu	Asia-Pacific	2005	No	No	Yes	Yes	Yes	No	28	33	1	1
Mayxay - 2010[120]	Laos	Asia-Pacific	2005-2006	Yes	No	Yes	Yes	Yes	No	63	300	2	1
Na Bangchang - 2010[135]	Thailand	Asia-Pacific	2008-2009	No	No	No	No	Yes	No	42	150	1	1
Noedl - 2010[137]	Cambodia	Asia-Pacific	2006-2007	Yes	No	No	No	Yes	No	28	94	2	1
Smithuis - 2010[28]	Cambodia	Asia-Pacific	2008-2009	Yes	No	Yes	Yes	Yes	Yes	63	808	10	3
Sutanto - 2010[164]	Indonesia	Asia-Pacific	2002	No	No	Yes	Yes	Yes	No	28	42	1	1
Thriemer - 2010[171]	Bangladesh	Asia-Pacific		Yes	No	No	Yes	Yes	No	42	228	2	1
Bethell - 2011[44]	Cambodia	Asia-Pacific	2008-2009	Yes	No	No	No	Yes	No	42	143	3	1
Hwang - 2011[81]	Ethiopia	Africa	2009	No	No	Yes	Yes	Yes	No	42	120	1	2
Benjamin - 2012[42]	Papua New Guinea	Asia-Pacific		Yes	No	No	Yes	No	Yes	42	48	3	1
Eshetu - 2012[69]	Ethiopia	Africa	2008-2009	No	No	Yes	Yes	Yes	No	42	348	1	4
Hien - 2012[80]	Vietnam	Asia-Pacific	2010-2011	Yes	No	No	Yes	Yes	No	42	166	3	1
Mayxay - 2012a[121]	Laos	Asia-Pacific	2010	Yes	No	No	Yes	Yes	No	42	44	2	1
Mayxay - 2012b[122]	Laos	Asia-Pacific	2008-2010	No	No	Yes	Yes	Yes	Yes	42	549	1	1
Mishra - 2012[126]	India	Asia-Pacific	2009-2010	No	No	Yes	Yes	Yes	No	28	1454	1	25
Starzengruber - 2012[159]	Bangladesh	Asia-Pacific		Yes	No	No	Yes	Yes	No	42	126	3	1
Thanh - 2012[167]	Vietnam	Asia-Pacific	2008-2009	Yes	No	No	Yes	Yes	No	42	128	2	1
Valecha -2012[174]	Multinational	Asia-Pacific	2007-2008	Yes	No	No	Yes	Yes	No	28	240	2	1
Kyaw - 2013[99]	Myanmar	Asia-Pacific	2011	No	No	No	No	Yes	No	28	53	1	1
Leang - 2013[102]	Cambodia	Asia-Pacific	2008-2011	No	No	Yes	Yes	Yes	Yes	42	438	1	4
Srivastava - 2013[158]	India	Asia-Pacific	2007-2010	No	No	Yes	Yes	Yes	No	28	149	1	3

Valecha - 2013[175]	India	Asia-Pacific	2007-2008	No	No	No	No	Yes	No	63	77	1	2
Laman - 2014[101]	Papua New Guinea	Asia-Pacific	2011-2013	Yes	No	Yes	No	No	Yes	42	198	2	1
Lon - 2014[106]	Cambodia	Asia-Pacific	2010-2011	Yes	No	No	No	Yes	Yes	42	20	2	1
Mishra - 2014[127]	India	Asia-Pacific	2012	No	No	Yes	Yes	Yes	No	42	190	1	3
Saunders - 2014[151]	Cambodia	Asia-Pacific	2013	Yes	No	No	No	Yes	No	42	50	1	1
Benjamin - 2015[43§]	Papua New Guinea	Asia-Pacific		Yes	No	No	No	Yes	No	42	4	2	1
Ebstie - 2015[68]	Ethiopia	Africa	2012	No	No	No	Yes	Yes	No	28	134	1	1
Mekonnen - 2015[124]	Ethiopia	Africa	2011	No	No	Yes	Yes	Yes	No	28	93	1	1
Spring - 2015[157]	Cambodia	Asia-Pacific	2012-2014	No	No	No	No	Yes	Yes	42	101	2	1
Amaratunga - 2016[34]	Cambodia	Asia-Pacific	2012-2013	No	No	Yes	Yes	Yes	No	63	241	1	3
Awab - 2016[38]	Afghanistan	Asia-Pacific	2007-2014	Yes	No	Yes	Yes	Yes	No	56	303	2	4
Ladeia-Andrade - 2016[100]	Brazil	The Americas	2010-2013	No	No	Yes	Yes	Yes	No	42	162	1	1
Leang - 2016[103]	Cambodia	Asia-Pacific	2014-2015	No	No	No	Yes	Yes	No	42	123	1	1
Mishra - 2016[128]	India	Asia-Pacific	2011-2012	No	No	Yes	Yes	Yes	No	42	1438	1	29
Phong - 2016[141]	Vietnam	Asia-Pacific	2010	No	No	No	Yes	Yes	No	42	60	2	1
Rahman - 2016[145]	Guyana	The Americas	2014	No	No	Yes	Yes	Yes	No	28	50	1	1
Wudneh - 2016[182]	Ethiopia	Africa	2014-2015	No	No	Yes	Yes	Yes	No	28	91	1	1
Teklemariam - 2017[166]	Ethiopia	Africa	2014-2015	No	No	Yes	Yes	Yes	No	28	92	1	1
Itoh - 2018[83]	Brazil	The Americas	2015-2016	No	No	No	Yes	Yes	No	28	85	1	1
Poespoprodjo - 2018[142]	Indonesia	Asia-Pacific	2015-2016	No	No	Yes	Yes	Yes	No	42	61	1	1

* Includes patients with *P. falciparum* mono-infection and mixed *P. falciparum* and *P. vivax*; † Excluded 106 patients with severe malaria; ‡ Artemether-mefloquine arm followed for 42 days and artemether arm followed for 28 days; § Two pregnant women excluded from analysis.

Table S7. Study sites and treatment arms (records) included in meta-analysis

Author-Year	Location	Country	Relapse periodicity category	Multiple sites	Years enrolled	Treatment arm	Multiple treatment arms	Total enrolled	Enrolled with mixed infection	Before day 7*	Day 28†			Day 42†			Day 63†		
											Pf	Pv	Total	Pf	Pv	Total	Pf	Pv	Total
Segal - 1974[152]	Prachinburi Province	Thailand	10		1972	Quinine		26	0	1	0	1	22						
Segal - 1974[152]	Prachinburi Province	Thailand	10		1972	WR 33063		25	0	1	1	3	25						
Hall - 1975[79]	Trat	Thailand	10		1973-1974	Amodiaquine		24	0	2	11	0	21						
Hall - 1975[79]	Trat	Thailand	10		1973-1974	Amodiaquine		17	0	2	6	0	13						
Hall - 1975[79]	Trat	Thailand	10		1973-1974	Chloroquine		13	0	10	1	0	11						
Segal - 1975[153]	Prachinburi Province	Thailand	10		1972-1973	Dapsone-Pyrimethamine		22	0	0	17	1	21						
Segal - 1975[153]	Prachinburi Province	Thailand	10		1972-1973	Sulfadoxine-Pyrimethamine		22	0	3	1	2	17						
Doberstyn - 1979[64]	Saraburi Province	Thailand	10		1976	Mefloquine		37	0	0	0	0	37						
Doberstyn - 1979[64]	Saraburi Province	Thailand	10		1976	Sulfadoxine-Pyrimethamine		38	0	2	2	3	38						
de Souza - 1983[59]	Paragominas	Brazil	3		NS	Mefloquine		49	0	0	0	0	49	1	5	49	1	11	49
de Souza - 1983[59]	Paragominas	Brazil	3		NS	Sulfadoxine-Pyrimethamine		50	0	5							8	15	47
Harinasuta - 1983[76]	Bangkok	Thailand	10		1980-1981	Mefloquine		50	0	0							2	11	40
Harinasuta - 1983[76]	Bangkok	Thailand	10		1980-1981	Mefloquine		49	0	0							3	16	40
Harinasuta - 1983[76]	Bangkok	Thailand	10		1980-1981	Mefloquine		48	0	0							0	16	38
Li - 1984[32‡]	Hainan Island	China	10		1982-1984	Artemisinin		20	0	NS	7	3	20						
Li - 1984[32‡]	Hainan Island	China	10		1982-1984	Mefloquine+Artemisinin		20	0	NS	0	0	20						
Li - 1984[32‡]	Hainan Island	China	10		1982-1984	Mefloquine+Sulfadoxine-Pyrimethamine		20	0	NS	0	2	20						
Li - 1984[32‡]	Hainan Island	China	10		1982-1984	Mefloquine+Sulfadoxine-Pyrimethamine+Artemisinin		20	0	NS	0	0	20						
Botero - 1985[45]	Medellin	Colombia	3		1982-1983	Mefloquine+/-Sulfadoxine-Pyrimethamine	Yes	75	0	6	0	2	74						
de Souza - 1985[60]	Paragominas	Brazil	3		NS	Mefloquine		50	0	0	0	0	49	0	2	49			
de Souza - 1985[60]	Paragominas	Brazil	3		NS	Quinine+Sulfadoxine-Pyrimethamine		50	0	0	4	3	50	4	7	50			
Harinasuta - 1985[77]	Bangkok	Thailand	10		1981-1982	Mefloquine		20	0	0	4	0	19	4	3	17			
Harinasuta - 1985[77]	Bangkok	Thailand	10		1981-1982	Mefloquine+Sulfadoxine-Pyrimethamine		20	0	2	0	0	19	1	0	18			
Meek - 1986[123]	Kap Choeng, Rongphayaban	Thailand	10		1983	Mefloquine+Sulfadoxine-Pyrimethamine		51	0	1	0	0	51	0	0	40			
Meek - 1986[123]	Kap Choeng, Rongphayaban	Thailand	10		1983	Quinine+Tetracycline		50	0	0	7	6	50	11	7	28			
Meek - 1986[123]	Kap Choeng, Rongphayaban	Thailand	10		1983	Quinine+Tetracycline		49	0	0	3	8	49	5	8	27			
Pe - 1986[140§]	Tharrawaddy	Myanmar	10	Yes		Artemether		25	2	0	4	2	23						
Chongsuphajaisiddhi - 1987[57]	Bangkok	Thailand	10		1982-1984	Mefloquine		84	0	0	2	3	82	2	10	82			
Harinasuta - 1987[78]	Bangkok	Thailand	10		1983-1984	Mefloquine+Sulfadoxine-Pyrimethamine		46	0	0	3	0	44	3	6	44			
Harinasuta - 1987[78]	Bangkok	Thailand	10		1983-1984	Mefloquine+Sulfadoxine-Pyrimethamine		48	0	0	1	0	45	1	1	45			
Nosten - 1987[138]	Shoklo	Thailand	10		1986	Mefloquine+Sulfadoxine-Pyrimethamine		1707	NS	0	13	6	1706						

Boudreau - 1988[46]	Thai-Cambodian border	Thailand	10	1982-1982	Mefloquine	40	0	1	0	1	39			
Boudreau - 1988[46]	Thai-Cambodian border	Thailand	10	1982-1983	Mefloquine	25	0	0	3	0	24			
Boudreau - 1988[46]	Thai-Cambodian border	Thailand	10	1982-1983	Halofantrine	20	0	1	6	0	20			
Boudreau - 1988[46]	Thai-Cambodian border	Thailand	10	1982-1983	Halofantrine	62	0	0	7	7	65			
Kremsner - 1989[94]	Rio Branco City	Brazil	3	1987	Clindamycin	10	5	0	0	5	10			
Salazar - 1990[150]	Muntinlupa City	Philippines	10	1984-1985	Clindamycin	8	0	2	0	0	7			
Salazar - 1990[150]	Muntinlupa City	Philippines	10	1984-1985	Clindamycin	12	0	1	0	1	9			
Salazar - 1990[150]	Muntinlupa City	Philippines	10	1984-1985	Clindamycin	28	0	3	0	1	18			
Bunnag - 1991a[47]	Bangkok	Thailand	10	NS	Artesunate	25	0	0	7	8	25			
Bunnag - 1991a[47]	Bangkok	Thailand	10	NS	Artesunate	25	0	0	6	7	25			
Bunnag - 1991b[48]	Bangkok	Thailand	10	NS	Artesunate	43	0	0	3	15	40			
Bunnag - 1991b[48]	Bangkok	Thailand	10	NS	Artesunate	46	0	0	5	8	40			
Bunnag - 1991c[49]	Bangkok	Thailand	10	1989	Artemether	6	0	0	0	1	5			
Bunnag - 1991c[49]	Bangkok	Thailand	10	1989	Artesunate	5	0	0	5	0	5			
Bunnag - 1991c[49]	Bangkok	Thailand	10	1989	Artesunate	5	0	0	5	0	5			
Bunnag - 1991c[49]	Bangkok	Thailand	10	1989	Artesunate	5	0	0	4	0	5			
Bunnag - 1991c[49]	Bangkok	Thailand	10	1989	Artesunate	6	0	0	0	1	5			
Bunnag - 1991c[49]	Bangkok	Thailand	10	1989	Artesunate	10	0	0	1	2	10			
Bunnag - 1991c[49]	Bangkok	Thailand	10	1989	Artesunate	21	0	0	1	8	20			
Bunnag - 1991c[49]	Bangkok	Thailand	10	1989	Artesunate+Chloroquine	5	0	0	5	0	5			
Bunnag - 1991c[49]	Bangkok	Thailand	10	1989	Artesunate+Sulfadoxine-Pyrimethamine	5	0	0	5	0	5			
Bunnag - 1992a[50]	Bangkok	Thailand	10	NS	Mefloquine+Sulfadoxine-Pyrimethamine+Tetracycline	26	0	0	6	0	25			
Bunnag - 1992a[50]	Bangkok	Thailand	10	NS	Mefloquine+Tetracycline	25	0	1	6	0	25			
Bunnag - 1992b[51]	Bangkok	Thailand	10	1989-1990	Artemether	33	0	0	5	2	32			
Bunnag - 1992b[51]	Bangkok	Thailand	10	1989-1990	Artemether	28	0	0	2	5	29			
Karbwang - 1992[86]	Bangkok	Thailand	10	NS	Artemether	34	0	0	1	9	30			
Karbwang - 1992[86]	Bangkok	Thailand	10	NS	Mefloquine	12	0	2	2	1	9	2	0	9
Karbwang - 1994[87]	Bangkok	Thailand	10	NS	Artesunate	31	0	0	1	5	30			
Karbwang - 1994[87]	Bangkok	Thailand	10	NS	Quinine+Tetracycline	33	0	0	0	9	30			
Looareesuwan - 1994[107]	Bangkok	Thailand	10	1992	Artesunate+Doxycycline	55	0	0	10	16	49			
Looareesuwan - 1994[107]	Bangkok	Thailand	10	1992	Mefloquine+Doxycycline	54	0	0	2	1	48			
Na Bangchang - 1994[130§]	Bangkok	Thailand	10		Artemether	8	0	0	1	3	8			
Oemijati - 1994[139]	Dili	East Timor	10	1988-1989	Clindamycin	39	0	0	0	3	33			
Oemijati - 1994[139]	Dili	East Timor	10	1988-1989	Sulfadoxine-Pyrimethamine	7	0	0	0	0	5			
Bunnag - 1995[52]	Makarm District, Chantaburi	Thailand	10	1992-1994	Mefloquine+Artemether	80	0	0	2	2	70	2	2	70
Bunnag - 1995[52]	Makarm District, Chantaburi	Thailand	10	1992-1994	Mefloquine+Artemether	79	0	0	7	3	73	7	3	73
Karbwang - 1995a[88]	Bangkok	Thailand	10	1993	Artemether	53	0	0	6	10	50			
Karbwang - 1995a[88]	Bangkok	Thailand	10	1993	Mefloquine+Artemether	56	0	0	2	2	50	2	2	50
Karbwang - 1995b[89]	Bangkok	Thailand	10	NS	Mefloquine+Artemether	27	0	0	7	0	27	7	0	27

Karbwang - 1995b[89]	Bangkok	Thailand	10		NS	Mefloquine+Artemether		30	0	0	1	0	28	1	0	28
Baird - 1996[39]	Yogyakarta	Indonesia	10		1994	Chloroquine		11	0	0	0	0	11			
Bunnag - 1996a[53]	Bangkok	Thailand	10		1990-1992	Quinine+Tetracycline		48	0	0	6	3	46			
Bunnag - 1996a[53]	Bangkok	Thailand	10		1990-1992	Quinine+Tetracycline		42	0	0	0	3	40			
Bunnag - 1996b[54]	Chantaburi	Thailand	10		1992-1994	Mefloquine+Artesunate		60	0	0				2	0	57
Bunnag - 1996b[54]	Chantaburi	Thailand	10		1992-1994	Mefloquine+Artemether		60	0	0				1	0	58
Duarte - 1996[66]	Cuiaba, Mato Grosso	Brazil	3		1992-1993	Artesunate+Tetracycline		88	0	0	2	13	72			
Duarte - 1996[66]	Cuiaba, Mato Grosso	Brazil	3		1992-1993	Quinine+Tetracycline		88	0	0	1	8	69			
Looareesuwan - 1996a[108]	Bangkok	Thailand	10		1994-1995	Dihydroartemisinin		53	0	0	5	8	49			
Looareesuwan - 1996b[109]	Bangkok	Thailand	10		1990-1993	Multiple	Yes	292	NS	4	49	76	277			
Looareesuwan - 1996c[110]	Bangkok	Thailand	10		1992	Pyronaridine		69	0	0	22	1	60			
Looareesuwan - 1996c[110]	Bangkok	Thailand	10		1992	Pyronaridine		32	0	0	3	0	26			
Mohapatra - 1996[129]	Upper Assam	India	10		1995	Arteether		37	0	0	2	2	30			
Na Bangchang - 1996a[131]	Mae Sot	Thailand	10		1995	Artemether+Pyrimethamine		20	0	0	15	0	15			
Na Bangchang - 1996a[131]	Mae Sot	Thailand	10		1995	Artemether+Pyrimethamine		20	0	0	13	0	18			
Na Bangchang - 1996a[131]	Mae Sot	Thailand	10		1995	Artemether+Pyrimethamine		20	0	0	5	2	20			
Na Bangchang - 1996b[132]	Mae Sot	Thailand	10		1995	Artemether+Azithromycin		30	0	0	23	0	27			
Na Bangchang - 1996b[132]	Mae Sot	Thailand	10		1995	Artemether+Doxycycline		30	0	0	14	0	30			
Restrepo - 1996[147¶]	Medellin	Colombia	3			Halofantrine	Yes	120	NS	NS				46	18	120
Bunnag - 1997[55]	Chantaburi	Thailand	10		1996	Mefloquine+Artesunate		49	0	0	0	0	47	0	0	47
Bunnag - 1997[55]	Chantaburi	Thailand	10		1996	Mefloquine+Artemether		50	0	0	0	0	50	0	0	50
de Alencar - 1997[58]	Peixoto de Azevedo	Brazil	3		1995-1996	Atovaquone-Proguanil		77	0	0	1	4	77			
de Alencar - 1997[58]	Peixoto de Azevedo	Brazil	3		1995-1996	Quinine+Tetracycline		77	0	0	0	8	77			
Jiao - 1997[85]	Hainan Island	China	10		NS	Artemether-Lumefantrine		102	0	0	4	0	102			
Looareesuwan - 1997a[111]	Bangkok	Thailand	10		1992	Artemether		40	0	0	10	15	38			
Looareesuwan - 1997a[111]	Bangkok	Thailand	10		1992	Artemether		58	0	0	1	23	49			
Looareesuwan - 1997a[111]	Bangkok	Thailand	10		1992	Mefloquine+Artemether		53	0	0	1	0	44			
Looareesuwan - 1997b[112]	Bangkok	Thailand	10		1995	Artesunate		45	0	0	0	5	42			
Looareesuwan - 1997b[112]	Bangkok	Thailand	10		1995	Artesunate		46	0	0	1	8	40			
Na Bangchang - 1997[133]	Mae Sot	Thailand	10		1994-1995	Mefloquine+Artemether		126	0	0	4	1	54	4	1	54
Thimarsarn - 1997[169]	Pong Nam Ron District of Chanthaburi Province	Thailand	10	Yes	1993-1994	Artemether		42	0	0	1	6	39			

Thimarsarn - 1997[169]	Pong Nam Ron District of Chanthaburi Province	Thailand	10	Yes	1993-1994	Artesunate		55	0	0	1	4	54						
Thimarsarn - 1997[169]	Pong Nam Ron District of Chanthaburi Province	Thailand	10	Yes	1993-1994	Mefloquine+Artesunate		80	0	0	1	1	78						
Thimarsarn - 1997[169]	Pong Nam Ron District of Chanthaburi Province	Thailand	10	Yes	1993-1994	Mefloquine+Artesunate		68	0	0	3	1	57						
Thimarsarn - 1997[169]	Pong Nam Ron District of Chanthaburi Province	Thailand	10	Yes	1993-1994	Mefloquine+Artemether		45	0	0	1	0	40						
Thimarsarn - 1997[169]	Pong Nam Ron District of Chanthaburi Province	Thailand	10	Yes	1993-1994	Mefloquine		45	0	0	0	1	43						
Thimarsarn - 1997[169]	Pong Nam Ron District of Chanthaburi Province	Thailand	10	Yes	1993-1994	Quinine+Tetracycline		47	0	1	2	4	66						
Karbwang - 1998[90]	Bangkok	Thailand	10			Artemether		13	0	0	2	4	13						
Sabchareon - 1998[149]	Kanchanaburi	Thailand	10		1994-1995	Atovaquone-Proguanil		32	0	0	0	2	28						
van Vugt - 1998[177]	Mae La	Thailand	10		1995-1996	Mefloquine+Artesunate		309	0	0							28	57	230
van Vugt - 1998[177]	Mae La	Thailand	10		1995-1996	Artemether-Lumefantrine		308	0	0							61	83	248
Wilairatana - 1998[180]	Bangkok	Thailand	10		1997-1998	Mefloquine+Artesunate		150	0	0	4	9	130						
Cerutti - 1999[56]	Peixoto de Azevedo	Brazil	3		1996-1997	Mefloquine		109	0	1	1	9	96						
Looareesuwan - 1999[113]	Bangkok	Thailand	10		1993-1994	Atovaquone-Proguanil		91	0	0	0	8	79						
Looareesuwan - 1999[113]	Bangkok	Thailand	10		1993-1994	Mefloquine		91	0	0	11	0	79						
Na Bangchang - 1999[134]	Bangkok	Thailand	10		1996-1998	Mefloquine+Dihydroartemisinin		26	0	0				8	2	23			
Na Bangchang - 1999[134]	Bangkok	Thailand	10		1996-1998	Mefloquine+Dihydroartemisinin		22	0	0				9	1	16			
Na Bangchang - 1999[134]	Bangkok	Thailand	10		1996-1998	Mefloquine+Dihydroartemisinin		78	0	0				2	4	70			
Na Bangchang - 1999[134]	Bangkok	Thailand	10		1996-1998	Mefloquine+Dihydroartemisinin		81	0	0				3	6	70			
van Vugt - 1999[178]	Mae La	Thailand	10	Yes	1996-1997	Artemether-Lumefantrine	Yes	359	NS	0	25	54	306						
Karbwang - 2000[91]	Mae Sot	Thailand	10	Yes		Artemether-Lumefantrine		87	2	0	19	14	81						
Karbwang - 2000[91]	Mae Sot	Thailand	10	Yes		Artemether-Lumefantrine		87	0	0	35	6	76						
Karbwang - 2000[91]	Mae Sot	Thailand	10	Yes		Artemether-Lumefantrine		86	4	0	37	10	79						
Kshirsagar - 2000[98§]	Mumbai	India	8		1996-1997	Multiple	Yes	179	0	12	52	1	142						
Pukrittayakamee - 2000[143]	Bangkok	Thailand	10		1995-1997	Quinine		68	0	0	7	12	53						
Pukrittayakamee - 2000[143]	Bangkok	Thailand	10		1995-1997	Quinine+Tetracycline		68	0	0	1	9	48						
Pukrittayakamee - 2000[143]	Bangkok	Thailand	10		1995-1997	Quinine+Clindamycin		68	0	0	0	12	60						
Krudsood - 2001[95]	Bangkok	Thailand	10		1992-1993	Halofantrine		40	0	0	4	2	35						
Krudsood - 2001[95]	Bangkok	Thailand	10		1992-1993	Halofantrine		40	0	0	6	0	28						
Krudsood - 2001[95]	Bangkok	Thailand	10		1992-1993	Halofantrine		40	0	0	1	0	33						
Lefevre - 2001[105]	Bangkok	Thailand	10		1998-1999	Artemether-Lumefantrine		164	16	0	7	6	155						
Lefevre - 2001[105]	Bangkok	Thailand	10		1998-1999	Mefloquine+Artesunate		55	7	0	0	0	53						
Tjitra - 2001[172]	Irian Jaya	Indonesia	12		1999	Artesunate+Sulfadoxine-Pyrimethamine		53	0	0	4	0	47						

Tjitra - 2001[172]	Irian Jaya	Indonesia	12		1999	Sulfadoxine-Pyrimethamine		52	0	2	7	0	46						
Baird - 2002[40]	Georgetown	Guyana	3		1998	Chloroquine		33	17	4	11	0	27						
Denis - 2002[61]	Oddor Meanchey	Cambodia	10		2001-2002	Dihydroartemisinin-Piperaquine		61	0	0	1	0	58						
Denis - 2002[61]	Snoul District	Cambodia	10		2001-2002	Dihydroartemisinin-Piperaquine		45	0	0	3	1	39						
Fontes - 2002[71]	Mato grosso	Brazil	3		NS	Proguanil+Sulfamethoxazole		31	0	0	4	1	22						
Fryauff - 2002[72]	Teluk Dalam, Nias	Indonesia	10		1998	Sulfadoxine-Pyrimethamine		39	0	15	14	3	38						
Krudsood - 2002[96]	Bangkok	Thailand	10		2001	Mefloquine+Artesunate		102	2	0	0	0	101						
Krudsood - 2002[96]	Bangkok	Thailand	10		2001	Mefloquine+Artesunate		102	0	0	1	0	102						
Maguire - 2002[114]	Purworejo	Indonesia	10	Yes	NS	Chloroquine		40	15	2	22	3	38						
Maguire - 2002[114]	Purworejo	Indonesia	10	Yes	NS	Sulfadoxine-Pyrimethamine		54	5	0	17	7	53						
van Vugt - 2002[179]	Mae Sot	Thailand	10		1998-2000	Mefloquine+Artesunate		533	0	NS				35	24	533			
van Vugt - 2002[179]	Mae Sot	Thailand	10		1998-2000	Artesunate+Atovaquone-Proguanil		533	0	NS				18	100	533			
van Vugt - 2002[179]	Mae Sot	Thailand	10		1998-2000	Atovaquone-Proguanil		530	0	NS				28	92	530			
Wilairatana - 2002[181]	Bangkok	Thailand	10		NS	Dihydroartemisinin-Piperaquine+Trimethoprim +Primaquine		234	0	0	5	2	194						
Wilairatana - 2002[181]	Bangkok	Thailand	10		NS	Mefloquine+Artesunate		118	0	0	2	0	82						
Ezard - 2003[70]	Los Palos	East Timor	10		2000	Chloroquine		48	0	NS	31	2	47						
Krudsood - 2003[97]	Bangkok	Thailand	10		NS	Artemether-Lumefantrine		41	0	0	1	0	34						
Krudsood - 2003[97§]	Bangkok	Thailand	10		NS	Dihydroartemisinin-Naphthoquine-Trimethoprim		89	0	0	1	1	80						
Mayxay - 2003[117]	Feuang	Laos	10		2000	Multiple	Yes	29	0	9				7	3	26			
Nguyen - 2003[136]	Dac Lac, Vietnam	Vietnam	10		NS	Artesunate+Chloroquine		28	0	0	1	0	28						
Nguyen - 2003[136]	Dac Lac, Vietnam	Vietnam	10		NS	Artesunate+Sulfadoxine-Pyrimethamine		29	0	0	0	0	29						
Nguyen - 2003[136]	Binh Phuoc Province	Vietnam	10		NS	Artesunate+Chloroquine		33	0	0	16	0	33						
Nguyen - 2003[136]	Binh Phuoc Province	Vietnam	10		NS	Artesunate+Sulfadoxine-Pyrimethamine		33	0	0	20	0	33						
Sumawinata - 2003[161]	Arso	Indonesia	12		1995	Chloroquine		75	20	38	31	4	74						
Suputtamongkol - 2003[162‖]	Saiyok District in Kanchanaburi	Thailand	10	Yes	1999-2001	Mefloquine+Artesunate		320	0	NS				15	6	238			
Suputtamongkol - 2003[162‖]	Saiyok District in Kanchanaburi	Thailand	10	Yes	1999-2001	Mefloquine+Primaquine		236	0	NS				26	2	177			
Ashley - 2004[35]	Bangkok	Thailand	10		2002-2003	Mefloquine+Artesunate		67	2	0	0	0	59						
Ashley - 2004[35]	Bangkok	Thailand	10		2002-2003	Dihydroartemisinin-Piperaquine		67	3	0	1	0	59						
Ashley - 2004[35]	Bangkok	Thailand	10		2002-2003	Artesunate+Dihydroartemisinin-Piperaquine		67	2	0	0	0	59						
Ashley - 2004[35]	Mae Sot	Thailand	10		2002-2003	Mefloquine+Artesunate		176	19	1							28	42	151
Ashley - 2004[35]	Mae Sot	Thailand	10		2002-2003	Dihydroartemisinin-Piperaquine		179	13	0							26	52	154
Ashley - 2004[35]	Mae Sot	Thailand	10		2002-2003	Dihydroartemisinin-Piperaquine		174	15	0							21	43	149
Mayxay - 2004[118]	Savannakhet Province	Laos	10		2002-2003	Chloroquine+Sulfadoxine-Pyrimethamine		110	0	6				9	0	109			
Mayxay - 2004[118]	Savannakhet Province	Laos	10		2002-2003	Mefloquine+Artesunate		110	0	0				2	0	108			
Mayxay - 2004[118]	Savannakhet Province	Laos	10		2002-2003	Artemether-Lumefantrine		110	0	0				14	5	107			
Pukrittayakamee - 2004[144]	Bangkok	Thailand	10		NS	Quinine		30	0	NS	4	2	25						
Pukrittayakamee - 2004[144]	Bangkok	Thailand	10		NS	Quinine+Tetracycline		30	0	NS	0	4	22						

Pukrittayakamee - 2004[144]	Bangkok	Thailand	10		NS	Quinine+Primaquine		29	0	NS	5	3	18						
Pukrittayakamee - 2004[144]	Bangkok	Thailand	10		NS	Quinine+Primaquine		37	0	NS	8	5	31						
Pukrittayakamee - 2004[144]	Bangkok	Thailand	10		NS	Artesunate		23	0	NS	2	5	21						
Pukrittayakamee - 2004[144]	Bangkok	Thailand	10		NS	Artesunate+Primaquine		27	0	NS	4	3	25						
Smithuis - 2004a[154]	Sittwe in Rakhine State	Myanmar	10		2000-2001	Mefloquine+Artesunate		203	8	4				12	32	187			
Smithuis - 2004a[154]	Sittwe in Rakhine State	Myanmar	10		2000-2001	Mefloquine+Artesunate		206	5	0				8	18	192			
Smithuis - 2004a[154]	Sittwe in Rakhine State	Myanmar	10		2000-2001	Mefloquine+Artesunate		196	19	3				7	9	180			
Smithuis - 2004a[154]	Sittwe in Rakhine State	Myanmar	10		2000-2001	Mefloquine+Artesunate		198	14	0				0	11	177			
Smithuis - 2004b[155]	Kachin state	Myanmar	10		1998	Chloroquine		79	0	32				29	1	77			
Smithuis - 2004b[155]	Kachin state	Myanmar	10		1998	Sulfadoxine-Pyrimethamine		79	0	18				41	5	73			
Smithuis - 2004b[155]	Kachin state	Myanmar	10		1998	Mefloquine		80	0	2				15	2	74			
Smithuis - 2004b[155]	Kachin state	Myanmar	10		1998	Mefloquine+Artesunate		78	0	3				12	4	73			
Stohrer - 2004[160]	Luang Namtha	Laos	10		2003	Artemether-Lumefantrine		53	0	0				13	2	49			
Stohrer - 2004[160]	Luang Namtha	Laos	10		2003	Mefloquine+Artesunate		55	0	0				8	1	54			
Sutanto - 2004[163]	East Nusa tenggara Province	Indonesia	10		2001-2002	Chloroquine		57	0	3	26	7	49						
Sutanto - 2004[163]	East Nusa tenggara Province	Indonesia	10		2001-2002	Sulfadoxine-Pyrimethamine		57	0	1	3	5	52						
Ashley - 2005[36]	Thai-Myanmar border	Thailand	10		2003-2004	Mefloquine+Artesunate		166	16	NS							27	38	157
Ashley - 2005[36]	Thai-Myanmar border	Thailand	10		2003-2004	Dihydroartemisinin-Piperaquine		164	15	NS							17	43	156
Ashley - 2005[36]	Thai-Myanmar border	Thailand	10		2003-2004	Dihydroartemisinin-Piperaquine		169	16	NS							10	46	161
Durrani - 2005[67]	Nangahar	Afghanistan	11		2002-2003	Chloroquine		79	0	3				60	0	71			
Durrani - 2005[67]	Nangahar	Afghanistan	11		2002-2003	Sulfadoxine-Pyrimethamine		27	0	1				1	0	24			
Durrani - 2005[67§]	Nangahar	Afghanistan	11		2002-2003	Amodiaquine		83	0	7				64	1	79			
Durrani - 2005[67‖]	Nangahar	Afghanistan	11		2002-2003	Amodiaquine+Artesunate		79	0	0				19	1	67			
Genton - 2005[73]	Kunjungini, East Sepik	Papua New Guinea	12		1994-1995	Amodiaquine		70	9	0	11	2	41						
Genton - 2005[73]	Kunjungini, East Sepik	Papua New Guinea	12		1994-1995	Chloroquine		79	1	2	7	0	52						
Hutagalung - 2005[82]	Mae Sot	Thailand	10		2001-2002	Artemether-Lumefantrine		245	0	0				27	90	225			
Hutagalung - 2005[82]	Mae Sot	Thailand	10		2001-2002	Mefloquine+Artesunate		245	0	0				24	29	227			
Marquino - 2005[116]	Sullana	Peru	3		2000	Artesunate+/-Sulfadoxine-Pyrimethamine	Yes	197	0	0	4	1	186						
van den Broek - 2005[176]	Khagrachari	Bangladesh	10		2003	Chloroquine+Sulfadoxine-Pyrimethamine		122	0	3				55	4	111			
van den Broek - 2005[176]	Khagrachari	Bangladesh	10		2003	Mefloquine+Artesunate		121	0	0				9	6	114			
van den Broek - 2005[176]	Khagrachari	Bangladesh	10		2003	Artemether-Lumefantrine		121	0	0				20	25	119			
Yeramian - 2005[183]	Bangkok	Thailand	10		2003	Pafuramidine		23	0	0	2	3	23						
Basano - 2006[41§]	Rondonia	Brazil	3		2000	Mefloquine		50	0	1				2	0	43			
Denis – 2006a[62§]	Multicentre	Cambodia	10	Yes	2001-2004	Mefloquine+Artesunate		1025	0	0	56	1	977	26	10	251			
Denis – 2006b[63§]	Sampov Lun	Cambodia	10		2002	Artemether-Lumefantrine		55	0	0	15	2	49						

Denis – 2006b[63§]	Sampov Lun	Cambodia	10		2003-2004	Artemether-Lumefantrine		80	0	0	12	1	77						
Denis – 2006b[63§]	Sampov Lun	Cambodia	10		2003-2004	Mefloquine+Artesunate		55	0	0	4	0	52						
Lederman - 2006[104§]	Menoreh Hills, Central Java	Indonesia	10		2001	Chloroquine		29	0	0	11	3	28						
Lederman - 2006[104§]	Menoreh Hills, Central Java	Indonesia	10		2001	Chloroquine+Sulfadoxine-Pyrimethamine+/-Primaquine	Yes	88	0	1	5	1	82						
Maguire – 2006[115**]	Armopa	Indonesia	12		1996-1999	Chloroquine		189	28	NS	124	11	185						
Maguire - 2006[115††]	Armopa	Indonesia	12		1996-1999	Mefloquine		259	27	1	11	1	249						
Mayxay - 2006[119]	Savannakhet Province	Laos	10		2004	Mefloquine+Artesunate		110	0	1				4	1	108			
Mayxay - 2006[119]	Savannakhet Province	Laos	10		2004	Dihydroartemisinin-Piperaquine		110	0	1				3	3	106			
Miller - 2006[125]	Sangkhlaburi District, Kanchanaburi	Thailand	10		1999-2001	Quinine+Doxycycline		10	0	0	0	0	10						
Miller - 2006[125]	Sangkhlaburi District, Kanchanaburi	Thailand	10		1999-2001	Azithromycin+Quinine		10	0	0	1	1	10						
Miller - 2006[125]	Sangkhlaburi District, Kanchanaburi	Thailand	10		1999-2001	Azithromycin+Quinine		20	0	0	0	2	20						
Miller - 2006[125]	Sangkhlaburi District, Kanchanaburi	Thailand	10		1999-2001	Azithromycin+Quinine		20	0	0	0	0	20						
Smithuis - 2006[156]	Rakhine state	Myanmar	10		2003-2004	Dihydroartemisinin-Piperaquine		156	21	0				4	22	152			
Smithuis - 2006[156]	Rakhine state	Myanmar	10		2003-2004	Dihydroartemisinin-Piperaquine		171	19	0				2	18	167			
Smithuis - 2006[156]	Rakhine state	Myanmar	10		2003-2004	Mefloquine+Artesunate		162	23	0				1	25	157			
Smithuis - 2006[156]	Rakhine state	Myanmar	10		2003-2004	Mefloquine+Artesunate		163	24	0				0	32	159			
Thriemer - 2006[170§]	Chittagong	Bangladesh	10		2004	Quinine+Sulfadoxine-Pyrimethamine		63	0	1				10	2	61			
Grande - 2007[74]	Iquitos	Peru	3		2003-2005	Dihydroartemisinin-Piperaquine		262	0	0							8	11	239
Grande - 2007[74]	Iquitos	Peru	3		2003-2005	Mefloquine+Artesunate		260	0	0							1	10	245
Haque - 2007[75‖]	Bandarban	Bangladesh	10	Yes	2005	Artemether-Lumefantrine		67	0	0				10	7	66			
Janssens - 2007[84]	Siem Reap	Cambodia	10	Yes	2002-2003	Mefloquine+Artesunate		236	NS	0							21	47	209
Janssens - 2007[84]	Siem Reap	Cambodia	10	Yes	2002-2003	Dihydroartemisinin-Piperaquine		228	NS	0							22	39	207
Ratcliff - 2007[146‖]	Timika	Indonesia	12		2004	Chloroquine+Sulfadoxine-Pyrimethamine		103	NS	4				43	10	69			
Thapa - 2007[168‡‡]	Jhapa District	Nepal	10		2005	Sulfadoxine-Pyrimethamine		33	0	1	3	0	33						
Thapa - 2007[168‡‡]	Jhapa District	Nepal	10		2005	Artemether-Lumefantrine		66	5	0	0	0	66						
Karunajeewa - 2008a[26]	East Sepik	Papua New Guinea	12	Yes	2005-2007	Chloroquine+Sulfadoxine-Pyrimethamine		110	NS	3				12	45	81			
Karunajeewa - 2008a[26]	East Sepik	Papua New Guinea	12	Yes	2005-2007	Artesunate+Sulfadoxine-Pyrimethamine		122	NS	2				13	56	103			
Karunajeewa - 2008a[26]	East Sepik	Papua New Guinea	12	Yes	2005-2007	Dihydroartemisinin-Piperaquine		123	NS	0				12	27	100			
Karunajeewa - 2008a[26]	East Sepik	Papua New Guinea	12	Yes	2005-2007	Artemether-Lumefantrine		127	NS	0				5	66	104			
Karunajeewa - 2008b[92]	Madang	Papua New Guinea	12		2005-2006	Dihydroartemisinin-Piperaquine		18	0	0	0	0	18	6	1	18			
Karunajeewa - 2008b[92]	Madang	Papua New Guinea	12		2005-2006	Chloroquine+Sulfadoxine-Pyrimethamine		17	0	0	2	6	17	4	6	17			
Tangpukdee - 2008[165]	Bangkok	Thailand	10		2006-2007	Mefloquine+Artesunate		65	0	0	0	0	65						
Tangpukdee - 2008[165]	Bangkok	Thailand	10		2006-2007	Piperaquine+Artemisinin +Primaquine		65	0	0	1	0	64						

Dondorp - 2009[65]	Pailin	Cambodia	10		2007-2008	Artesunate		20	0	3							7	7	20
Dondorp - 2009[65]	Pailin	Cambodia	10		2007-2008	Mefloquine+Artesunate		20	0	0							1	6	19
Dondorp - 2009[65]	Wang Pha	Thailand	10		2007-2008	Artesunate		20	0	0							10	4	15
Dondorp - 2009[65]	Wang Pha	Thailand	10		2007-2008	Mefloquine+Artesunate		20	0	0							5	2	18
Rogers - 2009[148]	Chumkiri	Cambodia	10		2006-2008	Mefloquine+Artesunate		150	0	4				28	8	143			
Tun - 2009[173]	Myanmar	Myanmar	10		2007	Artemisinin+Naphthoquine		55	0	0	1	2	55						
Assefa - 2010[37]	Kersa	Ethiopia	7		2007-2008	Artemether-Lumefantrine		90	0	0	3	5	85						
Kinzer - 2010[93]	Malo Island	Vanuatu	12		2005	Chloroquine+Sulfadoxine-Pyrimethamine		33	0	0	1	0	32						
Mayxay - 2010[120]	Xepon	Laos	10		2005-2006	Mefloquine+Artesunate		98	0	1							8	2	98
Mayxay - 2010[120]	Xepon	Laos	10		2005-2006	Dihydroartemisinin-Piperaquine		202	0	0							12	4	197
Na Bangchang - 2010[135]	Mae Tao	Thailand	10	Yes	2008-2009	Mefloquine+Artesunate +Primaquine		150	0	0				39	5	132			
Noedl - 2010[137]	Tasanh	Cambodia	10		2006-2007	Artesunate		74	0	0	4	12	72						
Noedl - 2010[137]	Tasanh	Cambodia	10		2006-2007	Quinine+Tetracycline		37	0	0	0	1	35						
Smithuis - 2010[28]	Kachin state	Myanmar	10	Yes	2008-2009	Amodiaquine+Artesunate+/-Primaquine	Yes	155	26	NS							28	59	150
Smithuis - 2010[28]	Kachin state	Myanmar	10	Yes	2008-2009	Artemether-Lumefantrine+/-Primaquine	Yes	162	25	NS							15	85	153
Smithuis - 2010[28]	Kachin state	Myanmar	10	Yes	2008-2009	Mefloquine+Artesunate+/-Primaquine	Yes	169	21	NS							5	55	162
Smithuis - 2010[28]	Kachin state	Myanmar	10	Yes	2008-2009	Mefloquine+Artesunate+/-Primaquine	Yes	161	31	NS							7	75	149
Smithuis - 2010[28]	Kachin state	Myanmar	10	Yes	2008-2009	Dihydroartemisinin-Piperaquine+/-Primaquine	Yes	161	26	NS							14	56	155
Sutanto - 2010[164]	Lampung	Indonesia	10		2002	Chloroquine		42	0	6	28	2	40						
Thriemer - 2010[171]	Bandarban	Bangladesh	10		NS	Artesunate+Azithromycin		152	0	0				22	8	150			
Thriemer - 2010[171]	Bandarban	Bangladesh	10		NS	Artemether-Lumefantrine		76	0	0				6	4	73			
Bethell - 2011[44]	Tasanh	Cambodia	10		2008-2009	Artesunate		75	0	5	2	6	73	3	12	72			
Bethell - 2011[44]	Tasanh	Cambodia	10		2008-2009	Artesunate		40	0	1	1	2	38	2	7	37			
Bethell - 2011[44]	Tasanh	Cambodia	10		2008-2009	Artesunate		28	0	2	2	6	25	2	7	25			
Hwang - 2011[81]	Oromia	Ethiopia	7	Yes	2009	Artemether-Lumefantrine		120	0	0	1	3	115	1	8	112			
Benjamin - 2012[42]	Madang	Papua New Guinea	12		NS	Artemisinin+Naphthoquine		15	1	0				5	3	15			
Benjamin - 2012[42]	Madang	Papua New Guinea	12		NS	Artemisinin+Naphthoquine		17	0	0				0	1	9			
Benjamin - 2012[42]	Madang	Papua New Guinea	12		NS	Artemisinin+Naphthoquine		16	0	0				0	0	11			
Eshetu - 2012[69]	Asendabo	Ethiopia	7	Yes	2008-2009	Artemether-Lumefantrine		348	0	0	3	0	317	28	0	316			
Hien - 2012[80]	Phuoc Long	Vietnam	10		2010-2011	Artesunate+Dihydroartemisinin-Piperaquine		55	0	2				1	1	50			
Hien - 2012[80]	Phuoc Long	Vietnam	10		2010-2011	Artesunate+Dihydroartemisinin-Piperaquine		56	0	0				0	1	47			
Hien - 2012[80]	Phuoc Long	Vietnam	10		2010-2011	Dihydroartemisinin-Piperaquine		55	0	0				1	0	52			
Mayxay - 2012a[121]	Savannakhet	Laos	10		2010	Artesunate		22	0	0	0	0	22	0	1	22			
Mayxay - 2012a[121]	Savannakhet	Laos	10		2010	Artesunate		22	0	0	0	1	22	1	1	22			
Mayxay - 2012b[122]	Xepon	Laos	10		2008-2010	Artemether-Lumefantrine		549	11	NS	43	18	541						
Mishra - 2012[126]	Multisite	India	8	Yes	2009-2010	Artesunate+Sulfadoxine-Pyrimethamine		1454	0	3	20	7	1397						

Starzengruber - 2012[159]	Bandarban	Bangladesh	10		NS	Artesunate	51	0	0				1	6	48			
Starzengruber - 2012[159]	Bandarban	Bangladesh	10		NS	Artesunate	50	0	0				1	3	47			
Starzengruber - 2012[159]	Bandarban	Bangladesh	10		NS	Quinine+Doxycycline	25	0	0				0	1	21			
Thanh - 2012[167]	Phuoc Chien	Vietnam	10		2008-2009	Artemisinin+Piperaquine	63	2	0				8	3	55			
Thanh - 2012[167]	Phuoc Chien	Vietnam	10		2008-2009	Amodiaquine+Artesunate	65	1	0				10	3	59			
Valecha -2012[174]	Rourkela	India	8		2007-2008	Arterolane+Piperaquine	51	0	0	0	0	46						
Valecha -2012[174]	Rourkela	India	8		2007-2008	Artemether-Lumefantrine	25	0	0	0	0	25						
Valecha -2012[174]	Ranchi	India	8		2007-2008	Arterolane+Piperaquine	3	0	0	0	0	3						
Valecha -2012[174]	Ranchi	India	8		2007-2008	Artemether-Lumefantrine	2	0	0	0	0	2						
Valecha -2012[174]	Jamshedpur	India	8		2007-2008	Arterolane+Piperaquine	44	0	0	0	0	41						
Valecha -2012[174]	Jamshedpur	India	8		2007-2008	Artemether-Lumefantrine	21	0	0	0	0	19						
Valecha -2012[174]	Bangkok	Thailand	10		2007-2008	Arterolane+Piperaquine	62	0	0	0	0	61						
Valecha -2012[174]	Bangkok	Thailand	10		2007-2008	Artemether-Lumefantrine	32	0	0	1	0	32						
Kyaw - 2013[99]	Kawthaung	Myanmar	10		2011	Artesunate	53	0	0	4	25	52						
Leang - 2013[102]	Multisite	Cambodia	10	Yes	2008-2011	Dihydroartemisinin-Piperaquine	438	0	0				26	6	426			
Srivastava - 2013[158]	Ranchi	India	8		2007-2010	Artesunate+Sulfadoxine-Pyrimethamine	53	0	0	2	0	49						
Srivastava - 2013[158]	Keonjhar, Orissa	India	8		2007-2010	Artesunate+Sulfadoxine-Pyrimethamine	71	0	0	0	1	67						
Srivastava - 2013[158]	West Garo Hills	India	10		2007-2010	Artesunate+Sulfadoxine-Pyrimethamine	25	0	0	0	0	25						
Valecha - 2013[175]	Panjim	India	8	Yes	2007-2008	Mefloquine+Artesunate	77	0	0							1	7	66
Laman - 2014[101]	Madang	Papua New Guinea	12		2011-2013	Artemether-Lumefantrine	100	NS	0				5	32	94			
Laman - 2014[101]	Madang	Papua New Guinea	12		2011-2013	Artemisinin+Naphthoquine	98	NS	0				0	0	94			
Lon - 2014[106]	Oddar Meanchay	Cambodia	10		2010-2011	Dihydroartemisinin-Piperaquine	8	2	0				2	0	8			
Lon - 2014[106]	Oddar Meanchay	Cambodia	10		2010-2011	Dihydroartemisinin-Piperaquine	12	3	0				2	1	12			
Mishra - 2014[127]	Gomati	India	10		2012	Artesunate+Sulfadoxine-Pyrimethamine+Primaquine	77	0	4				21	1	69			
Mishra - 2014[127]	Lunglei	India	10		2012	Artesunate+Sulfadoxine-Pyrimethamine+Primaquine	71	0	0				16	0	63			
Mishra - 2014[127]	Changlang	India	10		2012	Artesunate+Sulfadoxine-Pyrimethamine+Primaquine	42	0	0				15	0	37			
Saunders - 2014[151]	Oddar Meanchay	Cambodia	10		2013	Dihydroartemisinin-Piperaquine	50	NS	0				20	4	46			
Benjamin - 2015[43]	Madang	Papua New Guinea	12		NS	Sulfadoxine-Pyrimethamine+Piperaquine	2	0	0	0	0	2	0	0	2			
Ebstie - 2015[68]	Bahir Dar	Ethiopia	7		2012	Artemether-Lumefantrine	134	0	0	2	1	131						
Mekonnen - 2015[124]	Nada	Ethiopia	7		2011	Artemether-Lumefantrine	93	0	0	4	1	89						
Spring - 2015[157§]	Oddar Meanchey	Cambodia	10		2012-2014	Dihydroartemisinin-Piperaquine+Primaquine	50	4	0				24	1	45			
Spring - 2015[157§]	Oddar Meanchey	Cambodia	10		2012-2014	Dihydroartemisinin-Piperaquine	51	4	0				22	2	48			
Amaratunga - 2016[34]	Pursat	Cambodia	10	Yes	2012-2013	Dihydroartemisinin-Piperaquine	241	0	0							56	9	221
Awab - 2016[38]	Multisite	Afghanistan	11	Yes	2007-2010	Dihydroartemisinin-Piperaquine	59	0	0	0	0	53	0	0	52			
Awab - 2016[38]	Multisite	Afghanistan	11	Yes	2007-2010	Artesunate+Sulfadoxine-Pyrimethamine	61	0	0	0	1	58	0	1	57			

Awab - 2016[38]	Jalalabad	Afghanistan	11		2010-2011	Artesunate+Sulfadoxine-Pyrimethamine	100	0	0	1	0	97	7	0	97
Awab - 2016[38]	Asadabad	Afghanistan	11		2012-2014	Artesunate+Sulfadoxine-Pyrimethamine	83	0	0	0	0	78	1	0	77
Ladeia-Andrade - 2016[100]	Cruzeiro do Sul	Brazil	3		2010-2013	Mefloquine+Artesunate	162	0	0				0	3	157
Leang - 2016[103]	Promoy	Cambodia	10		2014-2015	Artesunate+Pyronaridine	60	0	0				6	0	59
Leang - 2016[103]	Pailin City	Cambodia	10		2014-2015	Artesunate+Pyronaridine	55	0	0				8	1	50
Leang - 2016[103]	Tasanh	Cambodia	10		2014-2015	Artesunate+Pyronaridine	8	0	0				0	0	8
Mishra - 2016[128]	Multisite	India	8	Yes	2011-2012	Artesunate+Sulfadoxine-Pyrimethamine	1438	0	7				66	7	1351
Phong - 2016[141]	Thuan Bac District	Vietnam	10		2010	Artesunate	30	0	0	3	2	30			
Phong - 2016[141]	Thuan Bac District	Vietnam	10		2010	Artesunate+Azithromycin	30	0	0	1	1	30	2	1	30
Rahman - 2016[145]	Georgetown	Guyana	3		2014	Artesunate+Primaquine	50	0	0	0	3	50			
Wudneh - 2016[182]	Metema	Ethiopia	7		2014-2015	Artemether-Lumefantrine	91	0	0	1	1	82			
Teklemariam - 2017[166]	Setit Humera	Ethiopia	7		2014-2015	Artemether-Lumefantrine	92	0	0	1	2	81			
Itoh - 2018[83]	Cruzeiro do Sul	Brazil	3		2015-2016	Artemether-Lumefantrine	85	0	0	1	1	75			
Poespoprodjo - 2018[142]	Timika	Indonesia	12		2015-2016	Dihydroartemisinin-Piperaquine	61	0	0				2	0	43

NS – not stated; * Number of patients with persistent or recurrent parasitaemia before day 7; † Pf – *P. falciparum*, Pv – *P. vivax*, Total – Number of evaluable patients at day; ‡ Two treatment arms did not report outcomes and were excluded; § *P. falciparum* and *P. vivax* recurrence(s) occurred in separate patients; ¶ Four recurrences were mixed *P. falciparum* and *P. vivax* parasitaemia; || *P. falciparum* and *P. vivax* parasitaemia occurred as mixed recurrence(s); ** Only 112 patients treated with chloroquine but number of episodes recorded; †† Only 111 patients treated with mefloquine but number of episodes recorded; ‡‡ *P. vivax* recurrences identified by PCR but not microscopy.

Appendix 8. Map of study sites

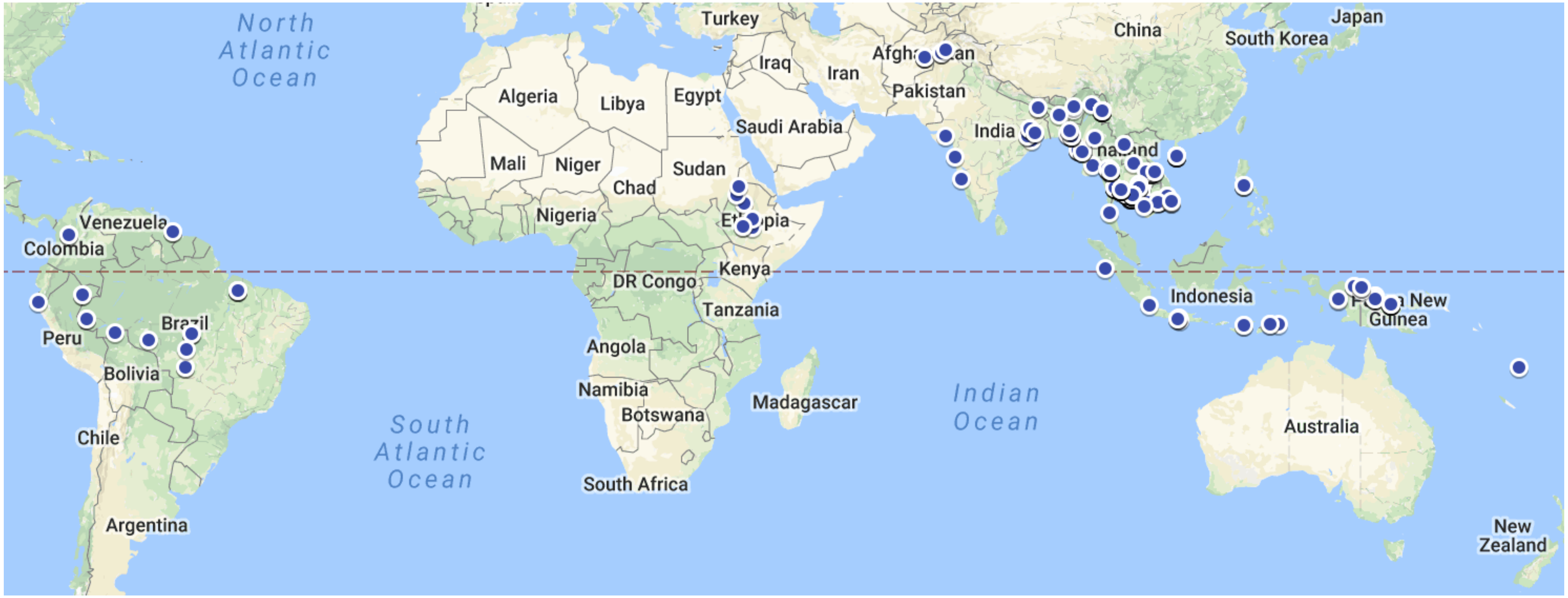

Figure S2. Location of study sites

Appendix 9. Tables of overall pooled estimates

Table S8. Pooled risk of parasitaemia at day 28 following *P. falciparum* infection by species

Subgroup	Records (studies)	*P. vivax* parasitaemia		Any parasitaemia[#]	
		I^2 (%)	Pooled percentage (95% CI)	I^2 (%)	Pooled percentage (95% CI)
Overall	213 (106)	86.1	3.8 (2.8-4.9)	95.4	15.3 (12.4-18.4)
Relapse periodicity					
Long	36 (24)	78.3	1.1 (0.2-2.4)	95.2	5.5 (2.0-10.2)
Short	177 (84)	85.4	4.5 (3.3-5.9)	94.8	17.7 (14.4-21.3)
Region					
Africa	7 (7)	64.3	1.4 (0.2-3.2)	59.7	3.3 (1.5-5.6)
The Americas	15 (12)	80.1	4.9 (1.9-8.8)	95.4	14.0 (4.9-26.2)
Asia-Pacific	191 (87)	86.3	3.9 (2.8-5.1)	95.3	16.0 (12.9-19.4)
Country specific analyses					
Thailand	119 (51)	86.1	5.3 (3.6-7.2)	94.8	18.5 (14.3-23.1)
Indonesia	14 (9)	72.0	4.3 (1.8-7.6)	96.1	33.0 (18.4-49.4)
India	12 (5)	5.9	0.0 (0.0-0.0)	89.9	1.4 (0.0-7.2)
Brazil	11 (8)	81.5	6.6 (2.4-12.2)	85.3	9.4 (3.9-16.6)
Myanmar	3 (3)	93.2	17.1 (0.1-48.9)	91.9	24.7 (3.5-55.4)
Laos	3 (2)	0	2.1 (0.9-3.7)	62.2	5.5 (0.4-14.4)
Cambodia	11 (5)	85.6	3.6 (0.7-8.0)	86.3	11.7 (6.3-18.4)
Ethiopia	7 (7)	64.3	1.4 (0.2-3.2)	59.7	3.3 (1.5-5.6)
Drug elimination half life					
Rapid	66 (36)	68.0	14.5 (11.7-17.6)	85.3	26.1 (21.0-31.4)
Intermediate	41 (28)	74.5	2.5 (1.2-4.1)	96.6	15.0 (8.2-23.1)
Slow	102 (60)	60.3	0.5 (0.2-1.0)	95.8	9.4 (6.2-13.1)
Major ACTs					
Artemether-lumefantrine	23 (17)	83.6	1.9 (0.4-3.9)	95.8	8.9 (3.6-15.8)
Dihydroartemisinin-piperaquine	5 (5)	0	0.0 (0.0-1.3)	20.3	1.4 (0.0-4.2)
Artesunate-mefloquine	12 (10)	60.1	0.3 (0.0-1.3)	77.5	2.1 (0.6-4.4)

CI – confidence interval; ACT – artemisinin-based combination therapy; * Parasitaemia from *P. vivax* or *P. falciparum* after initial infection.

Table S9. Pooled risk of parasitaemia at day 63 following *P. falciparum* infection by species

Subgroup	Records (studies)	*P. vivax* parasitaemia		Any parasitaemia*	
		I^2 (%)	Pooled percentage (95% CI)	I^2 (%)	Pooled percentage (95% CI)
Overall	30 (12)	94.4	24.0 (18.3-30.1)	94.8	35.7 (29.0-42.7)
Relapse periodicity					
Long	5 (3)	92.4	12.3 (4.0-24.1)	95.3	16.7 (4.9-33.5)
Short	25 (9)	93.7	26.6 (20.5-33.2)	92.3	39.9 (33.7-46.3)
Region					
Africa	0 (0)	-	-	-	-
The Americas	4 (2)	94.8	12.9 (2.6-28.8)	96.7	18.1 (3.4-40.4)
Asia-Pacific	26 (10)	93.7	25.9 (20.0-32.3)	92.9	38.7 (32.4-45.3)
Country specific analyses					
Thailand	13 (5)	17.8	28.8 (26.2-31.5)	69.7	43.4 (38.6-48.4)
Indonesia	0 (0)	-	-	-	-
India	1 (1)	-	10.6 (4.1-19.4)	-	12.1 (5.2-21.3)
Brazil	2 (1)	5.1	27.0 (18.2-36.7)	83.7	36.2 (14.6-61.1)
Myanmar	5 (1)	81.9	43.0 (34.8-51.3)	86.9	51.9 (42.1-61.6)
Laos	2 (1)	0	2.0 (0.6-4.0)	0	8.8 (5.7-12.3)
Cambodia	5 (3)	92.3	19.1 (8.4-32.6)	0.1	30.7 (27.2-34.3)
Ethiopia	0 (0)	-	-	-	-
Drug elimination half life					
Rapid	2 (1)	0	31.4 (16.5-48.2)	45.0	61.0 (37.8-82.1)
Intermediate	2 (1)	94.7	44.2 (23.6-65.9)	52.4	61.3 (54.1-68.3)
Slow	26 (12)	94.3	22.1 (16.4-28.4)	94.1	32.2 (25.8-39.0)
Major ACTs					
Artemether-lumefantrine	1 (1)	-	33.5 (27.7-39.5)	-	58.1 (51.9-64.2)
Dihydroartemisinin-piperaquine	8 (6)	96.4	16.2 (7.6-27.2)	96.1	28.7 (17.8-41.0)
Artesunate-mefloquine	9 (8)	92.0	15.7 (8.6-24.4)	94.4	25.2 (14.9-37.0)

CI – confidence interval; ACT – artemisinin-based combination therapy; * Parasitaemia from *P. vivax* or *P. falciparum* after initial infection.

Appendix 10. Funnel plots

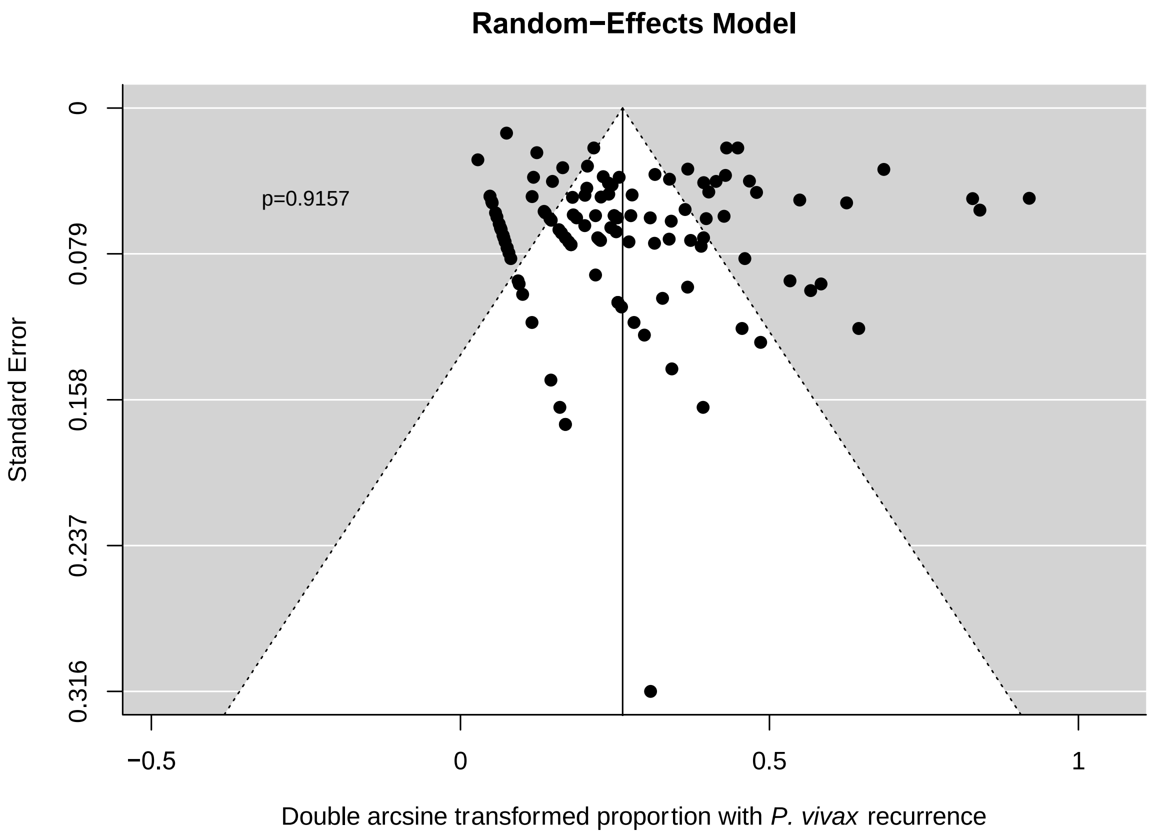

Figure S3. Funnel plot of the studies included in the overall meta-analysis of the risk of *P. vivax* parasitaemia at day 42 following *P. falciparum* infection

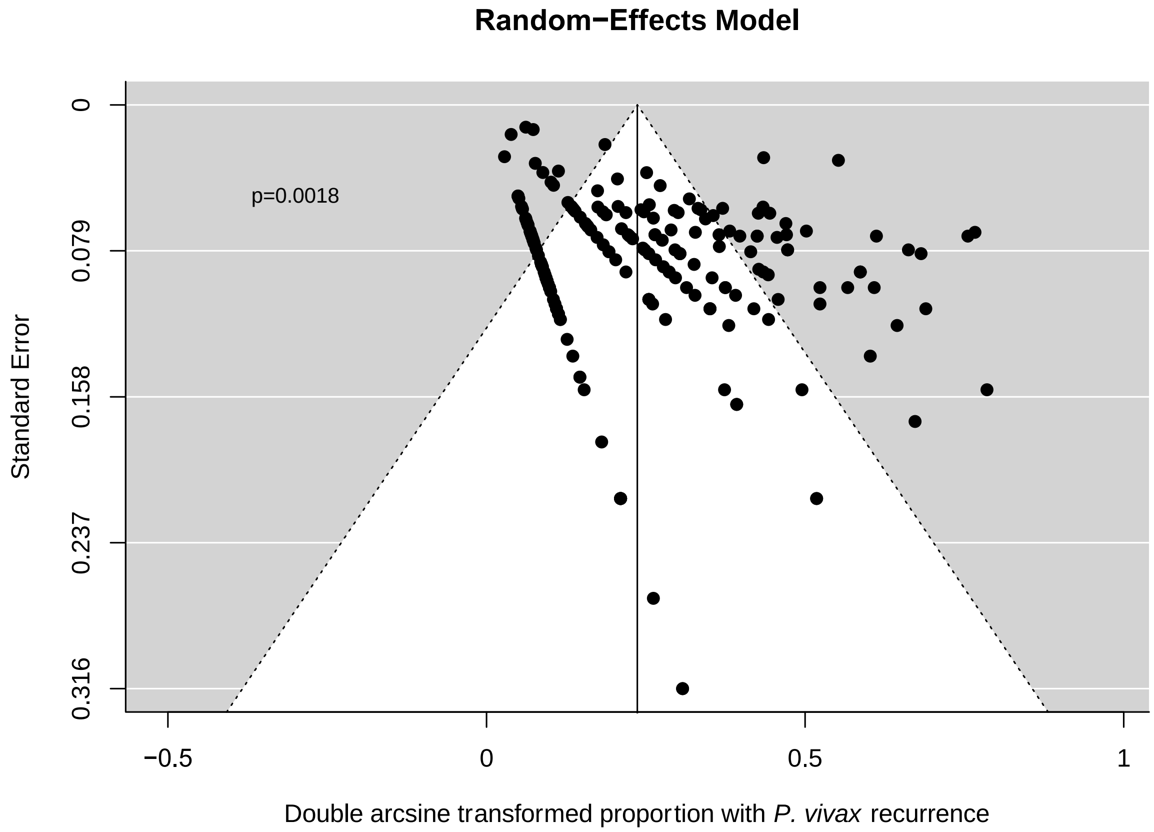

Figure S4. Funnel plot of the studies included in the overall meta-analysis of the risk of *P. vivax* parasitaemia at day 28 following *P. falciparum* infection

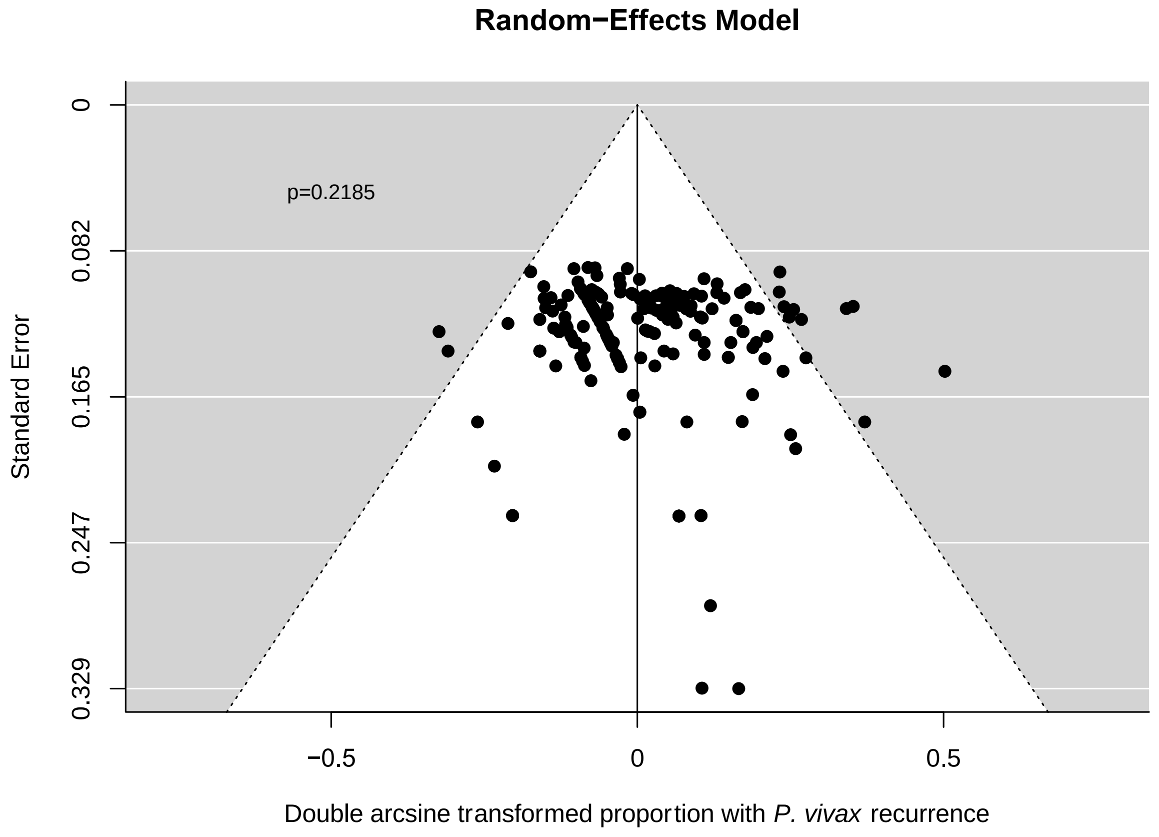

Figure S5. Funnel plot of the studies included in the overall meta-analysis of the risk of *P. vivax* parasitaemia at day 28 following *P. falciparum* infection adjusted for confounding related to drug elimination half-life

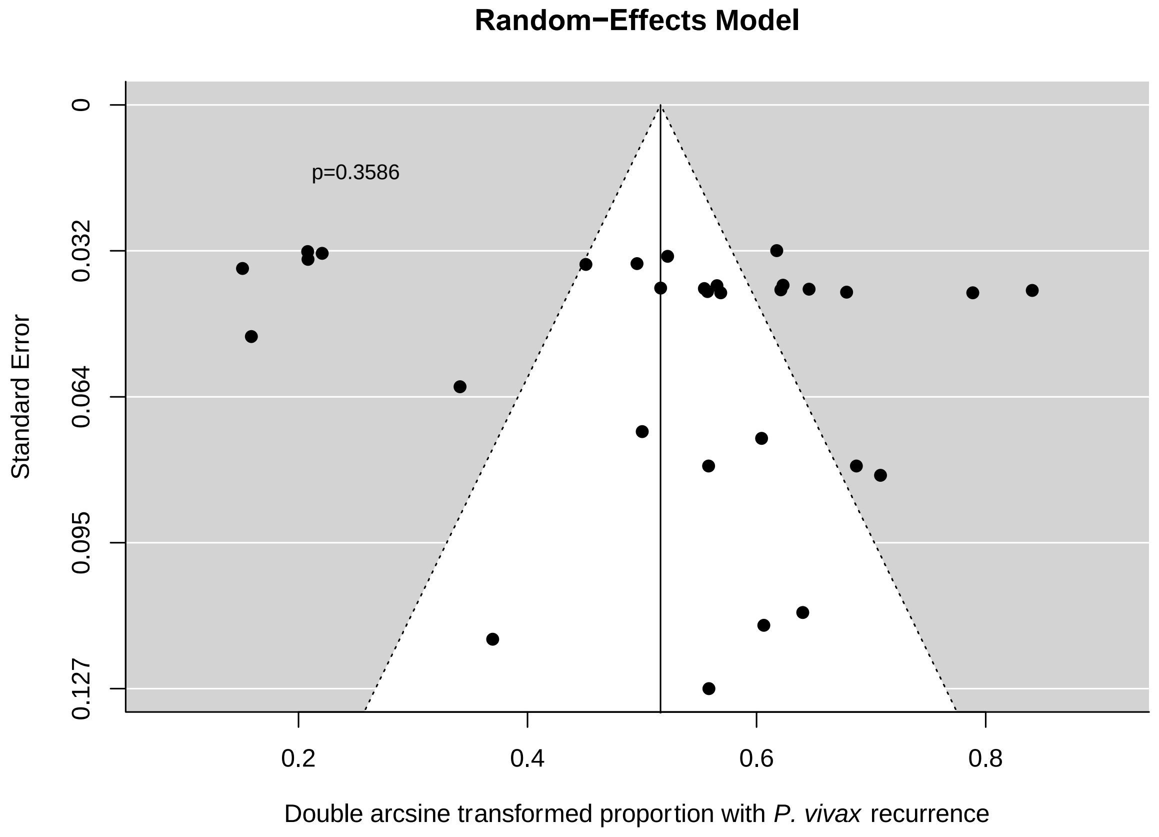

Figure S6. Funnel plot of the studies included in the overall meta-analysis of the risk of *P. vivax* parasitaemia at day 63 following *P. falciparum* infection

Appendix 11. Forest plots categorised by independent risk factors using the subset of records included in the multivariable meta-regressions

Author-Year *	Country	Treatment†	n/N	Risk‡, % (95% CI)
**Long periodicity**				
Durrani-2005	Afghanistan	AQ	1/79	1.27 [0.00, 5.36]
Durrani-2005	Afghanistan	As+AQ	1/67	1.49 [0.00, 6.30]
Awab-2016	Afghanistan	As+SP§	0/77	0.00 [0.00, 2.22]
Awab-2016	Afghanistan	As+SP¶	0/97	0.00 [0.00, 1.76]
Awab-2016	Afghanistan	As+SP‖	1/57	1.75 [0.00, 7.38]
Durrani-2005	Afghanistan	CQ	0/71	0.00 [0.00, 2.41]
Awab-2016	Afghanistan	DP	0/52	0.00 [0.00, 3.28]
Durrani-2005	Afghanistan	SP	0/24	0.00 [0.00, 7.04]
Basano-2006	Brazil	MQ	0/43	0.00 [0.00, 3.96]
Ladeia-Andrade-2016	Brazil	AM	3/157	1.91 [0.24, 4.79]
Eshetu-2012	Ethiopia	AL	0/316	0.00 [0.00, 0.54]
Hwang-2011	Ethiopia	AL	8/112	7.14 [2.99, 12.76]
Subtotal (RE analysis; I^2=56.0%, p=0.0057)				0.56 [0.00, 1.73]
**Short periodicity**				
Thriemer-2010	Bangladesh	AL	4/73	5.48 [1.20, 12.11]
van den Broek-2005a	Bangladesh	AL	25/119	21.01 [14.12, 28.83]
Starzengruber-2012	Bangladesh	As(28mg/kg)	3/47	6.38 [0.83, 15.55]
Starzengruber-2012	Bangladesh	As(14mg/kg)	6/48	12.50 [4.39, 23.58]
Thriemer-2010	Bangladesh	As+Az	8/150	5.33 [2.22, 9.59]
van den Broek-2005a	Bangladesh	CQ+SP	4/111	3.60 [0.78, 8.05]
van den Broek-2005a	Bangladesh	AM	6/114	5.26 [1.78, 10.25]
Starzengruber-2012	Bangladesh	Qu+Dox	1/21	4.76 [0.00, 19.32]
Bethell-2011	Cambodia	As(14mg/kg)	12/72	16.67 [8.84, 26.26]
Bethell-2011	Cambodia	As(42mg/kg)	7/25	28.00 [11.85, 47.45]
Bethell-2011	Cambodia	As(28mg/kg)	7/37	18.92 [7.67, 33.34]
Leang-2016	Cambodia	As+Py**	0/59	0.00 [0.00, 2.89]
Leang-2016	Cambodia	As+Py††	0/8	0.00 [0.00, 20.44]
Leang-2016	Cambodia	As+Py‡‡	1/50	2.00 [0.00, 8.39]
Leang-2013	Cambodia	DP	6/426	1.41 [0.47, 2.79]
Spring-2015	Cambodia	DP	2/48	4.17 [0.07, 12.15]
Spring-2015	Cambodia	DP+PQ	1/45	2.22 [0.00, 9.29]
Rogers-2009	Cambodia	AM	8/143	5.59 [2.33, 10.05]
Poespoprodjo-2018	Indonesia	DP	0/43	0.00 [0.00, 3.96]
Mayxay-2004	Laos	AL	5/107	4.67 [1.33, 9.64]
Stohrer-2004	Laos	AL	2/49	4.08 [0.07, 11.91]
Mayxay-2012b	Laos	As(6mg/kg)	1/22	4.55 [0.00, 18.49]
Mayxay-2012b	Laos	As(12mg/kg)	1/22	4.55 [0.00, 18.49]
Mayxay-2004	Laos	CQ+SP	0/109	0.00 [0.00, 1.57]
Mayxay-2006	Laos	DP	3/106	2.83 [0.35, 7.05]
Mayxay-2004	Laos	AM	0/108	0.00 [0.00, 1.59]
Mayxay-2006	Laos	AM	1/108	0.93 [0.00, 3.93]
Stohrer-2004	Laos	AM	1/54	1.85 [0.00, 7.78]
Smithuis-2004b	Myanmar	CQ	1/77	1.30 [0.00, 5.49]
Smithuis-2004b	Myanmar	MQ	2/74	2.70 [0.04, 7.97]
Smithuis-2004b	Myanmar	AM	4/73	5.48 [1.20, 12.11]
Smithuis-2004b	Myanmar	SP	5/73	6.85 [1.98, 13.99]
Benjamin-2012	PNG	Art2(22mg/kg)+N2	0/11	0.00 [0.00, 15.07]
Benjamin-2012	PNG	Art1(22mg/kg)+N1	1/9	11.11 [0.00, 41.77]
Benjamin-2012	PNG	Art1(15mg/kg)+N1	3/15	20.00 [2.98, 44.60]
Karunajeewa-2008b	PNG	CQ+SP	6/17	35.29 [14.00, 59.80]
Karunajeewa-2008b	PNG	DP	1/18	5.56 [0.00, 22.32]
Hutagalung-2005	Thailand	AL	90/225	40.00 [33.68, 46.49]
van Vugt-2002	Thailand	As+At+Pg	100/533	18.76 [15.56, 22.19]
van Vugt-2002	Thailand	At+Pg	92/530	17.36 [14.25, 20.71]
Karbwang-1992	Thailand	MQ	0/9	0.00 [0.00, 18.27]
Bunnag-1995	Thailand	Am+MQ(1250mg)	2/70	2.86 [0.05, 8.42]
Bunnag-1995	Thailand	Am+MQ(750mg)	3/73	4.11 [0.52, 10.16]
Bunnag-1996b	Thailand	Am+MQ	0/58	0.00 [0.00, 2.94]
Bunnag-1997	Thailand	Am+MQ	0/50	0.00 [0.00, 3.41]
Karbwang-1995a	Thailand	Am+MQ	2/50	4.00 [0.07, 11.68]
Bunnag-1996b	Thailand	AM	0/57	0.00 [0.00, 2.99]
Bunnag-1997	Thailand	AM	0/47	0.00 [0.00, 3.63]
Hutagalung-2005	Thailand	AM	29/227	12.78 [8.72, 17.46]
van Vugt-2002	Thailand	AM	24/533	4.50 [2.89, 6.44]
Na Bangchang-1999	Thailand	Dha+MQ(750mg day 1)	1/16	6.25 [0.00, 24.90]
Na Bangchang-1999	Thailand	Dha+MQ(750mg day 0)	2/23	8.70 [0.17, 24.46]
Na Bangchang-1999	Thailand	Dha+MQ(1250mg day 1)	4/70	5.71 [1.25, 12.61]
Na Bangchang-1999	Thailand	Dha+MQ(1250mg day 0)	6/70	8.57 [2.95, 16.45]
Harinasuta-1987	Thailand	MQ(750mg)+SP	1/45	2.22 [0.00, 9.29]
Harinasuta-1987	Thailand	MQ(500mg)+SP	6/44	13.64 [4.82, 25.59]
Thanh-2012	Vietnam	As+AQ	3/59	5.08 [0.65, 12.49]
Thanh-2012	Vietnam	Art+Pqp	3/55	5.45 [0.70, 13.37]
Phong-2016	Vietnam	As+Az	1/30	3.33 [0.00, 13.76]
Hien-2012	Vietnam	As(12mg/kg)+DP	1/47	2.13 [0.00, 8.91]
Hien-2012	Vietnam	As(6mg/kg)+DP	1/50	2.00 [0.00, 8.39]
Hien-2012	Vietnam	DP	0/52	0.00 [0.00, 3.28]
Subtotal (RE analysis; I^2=85.1%, p<0.0001)				4.85 [3.25, 6.71]

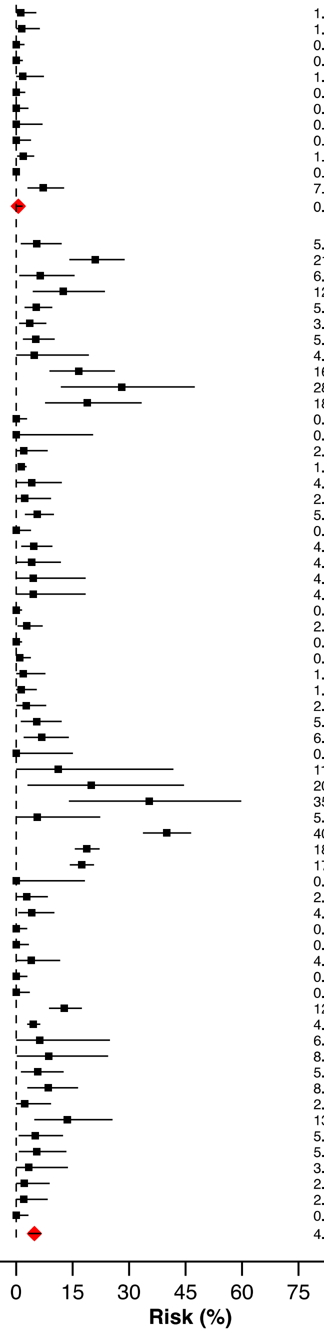

Figure S7. Day 42 risk of *P. vivax* parasitaemia following *P. falciparum* infection by relapse periodicity of region for studies included in multivariable meta-regression

Meta-regression results presented in Table 3; n/N – *P. vivax* parasitaemia/total evaluable patients at day 42; CI – confidence interval; AQ – amodiaquine; As – artesunate; SP – sulfadoxine-pyrimethamine; CQ – chloroquine; DP– dihydroartemisinin-piperaquine; MQ – mefloquine; AM – artesunate+mefloquine; AL – artemether-lumefantrine; RE – random effects; Az – azithromycin; Qu – quinine; Dox – doxycycline; Py – pyronaridine; PQ – primaquine; PNG – Papua New Guinea; Art – artemisinin; N – naphthoquine; At – atovaquone; Pg – proguanil; Am – artemether; Dha – dihydroartemisinin; Pqp – piperaquine; * Study and record details are provided in Appendix, pages 14-36; † Treatment arm described by drug with number of days given (total dose) where needed to distinguish from other treatment arms; ‡ Risk – percentage of patients with *P. vivax* parasitaemia; § Asadabad, Afghanistan; ¶ Jalalabad, Afghanistan; || Multisite, Afghanistan; ** Promoy, Cambodia; †† Tasanh, Cambodia; ‡‡ Pailin City, Cambodia.

Author-Year *	Country	Treatment†	n/N	Risk‡, % (95% CI)
**Rapidly eliminated**				
Starzengruber-2012	Bangladesh	As(28mg/kg)	3/47	6.38 [0.83, 15.55]
Starzengruber-2012	Bangladesh	As(14mg/kg)	6/48	12.50 [4.39, 23.58]
Starzengruber-2012	Bangladesh	Qu+Dox	1/21	4.76 [0.00, 19.32]
Bethell-2011	Cambodia	As(14mg/kg)	12/72	16.67 [8.84, 26.26]
Bethell-2011	Cambodia	As(42mg/kg)	7/25	28.00 [11.85, 47.45]
Bethell-2011	Cambodia	As(28mg/kg)	7/37	18.92 [7.67, 33.34]
Mayxay-2012b	Laos	As(12mg/kg)	1/22	4.55 [0.00, 18.49]
Mayxay-2012b	Laos	As(6mg/kg)	1/22	4.55 [0.00, 18.49]
Subtotal (RE analysis; I^2=35.7%, p=0.1255)				11.71 [7.12, 17.12]
**Intermediately eliminated**				
Thriemer-2010	Bangladesh	AL	4/73	5.48 [1.20, 12.11]
van den Broek-2005a	Bangladesh	AL	25/119	21.01 [14.12, 28.83]
Thriemer-2010	Bangladesh	As+Az	8/150	5.33 [2.22, 9.59]
Eshetu-2012	Ethiopia	AL	0/316	0.00 [0.00, 0.54]
Hwang-2011	Ethiopia	AL	8/112	7.14 [2.99, 12.76]
Mayxay-2004	Laos	AL	5/107	4.67 [1.33, 9.64]
Stohrer-2004	Laos	AL	2/49	4.08 [0.07, 11.91]
Hutagalung-2005	Thailand	AL	90/225	40.00 [33.68, 46.49]
van Vugt-2002	Thailand	As+At+Pg	100/533	18.76 [15.56, 22.19]
van Vugt-2002	Thailand	At+Pg	92/530	17.36 [14.25, 20.71]
Phong-2016	Vietnam	As+Az	1/30	3.33 [0.00, 13.76]
Subtotal (RE analysis; I^2=95.9%, p<0.0001)				9.64 [4.08, 17.07]
**Slowly eliminated**				
Durrani-2005	Afghanistan	AQ	1/79	1.27 [0.00, 5.36]
Durrani-2005	Afghanistan	As+AQ	1/67	1.49 [0.00, 6.30]
Awab-2016	Afghanistan	As+SP§	0/77	0.00 [0.00, 2.22]
Awab-2016	Afghanistan	As+SP¶	0/97	0.00 [0.00, 1.76]
Awab-2016	Afghanistan	As+SP‖	1/57	1.75 [0.00, 7.38]
Durrani-2005	Afghanistan	CQ	0/71	0.00 [0.00, 2.41]
Awab-2016	Afghanistan	DP	0/52	0.00 [0.00, 3.28]
Durrani-2005	Afghanistan	SP	0/24	0.00 [0.00, 7.04]
van den Broek-2005a	Bangladesh	CQ+SP	4/111	3.60 [0.78, 8.05]
van den Broek-2005a	Bangladesh	AM	6/114	5.26 [1.78, 10.25]
Basano-2006	Brazil	MQ	0/43	0.00 [0.00, 3.96]
Ladeia-Andrade-2016	Brazil	AM	3/157	1.91 [0.24, 4.79]
Leang-2016	Cambodia	As+Py**	0/59	0.00 [0.00, 2.89]
Leang-2016	Cambodia	As+Py††	0/8	0.00 [0.00, 20.44]
Leang-2016	Cambodia	As+Py‡‡	1/50	2.00 [0.00, 8.39]
Leang-2013	Cambodia	DP	6/426	1.41 [0.47, 2.79]
Spring-2015	Cambodia	DP	2/48	4.17 [0.07, 12.15]
Spring-2015	Cambodia	DP+PQ	1/45	2.22 [0.00, 9.29]
Rogers-2009	Cambodia	AM	8/143	5.59 [2.33, 10.05]
Poespoprodjo-2018	Indonesia	DP	0/43	0.00 [0.00, 3.96]
Mayxay-2004	Laos	CQ+SP	0/109	0.00 [0.00, 1.57]
Mayxay-2006	Laos	DP	3/106	2.83 [0.35, 7.05]
Mayxay-2004	Laos	AM	0/108	0.00 [0.00, 1.59]
Mayxay-2006	Laos	AM	1/108	0.93 [0.00, 3.93]
Stohrer-2004	Laos	AM	1/54	1.85 [0.00, 7.78]
Smithuis-2004b	Myanmar	CQ	1/77	1.30 [0.00, 5.49]
Smithuis-2004b	Myanmar	MQ	2/74	2.70 [0.04, 7.97]
Smithuis-2004b	Myanmar	AM	4/73	5.48 [1.20, 12.11]
Smithuis-2004b	Myanmar	SP	5/73	6.85 [1.98, 13.99]
Benjamin-2012	PNG	Art2(22mg/kg)+N2	0/11	0.00 [0.00, 15.07]
Benjamin-2012	PNG	Art1(22mg/kg)+N1	1/9	11.11 [0.00, 41.77]
Benjamin-2012	PNG	Art1(15mg/kg)+N1	3/15	20.00 [2.98, 44.60]
Karunajeewa-2008b	PNG	CQ+SP	6/17	35.29 [14.00, 59.80]
Karunajeewa-2008b	PNG	DP	1/18	5.56 [0.00, 22.32]
Karbwang-1992	Thailand	MQ	0/9	0.00 [0.00, 18.27]
Bunnag-1995	Thailand	Am+MQ(1250mg)	2/70	2.86 [0.05, 8.42]
Bunnag-1995	Thailand	Am+MQ(750mg)	3/73	4.11 [0.52, 10.16]
Bunnag-1996b	Thailand	Am+MQ	0/58	0.00 [0.00, 2.94]
Bunnag-1997	Thailand	Am+MQ	0/50	0.00 [0.00, 3.41]
Karbwang-1995a	Thailand	Am+MQ	2/50	4.00 [0.07, 11.68]
Bunnag-1996b	Thailand	AM	0/57	0.00 [0.00, 2.99]
Bunnag-1997	Thailand	AM	0/47	0.00 [0.00, 3.63]
Hutagalung-2005	Thailand	AM	29/227	12.78 [8.72, 17.46]
van Vugt-2002	Thailand	AM	24/533	4.50 [2.89, 6.44]
Na Bangchang-1999	Thailand	Dha+MQ(750mg day 1)	1/16	6.25 [0.00, 24.90]
Na Bangchang-1999	Thailand	Dha+MQ(750mg day 0)	2/23	8.70 [0.17, 24.46]
Na Bangchang-1999	Thailand	Dha+MQ(1250mg day 1)	4/70	5.71 [1.25, 12.61]
Na Bangchang-1999	Thailand	Dha+MQ(1250mg day 0)	6/70	8.57 [2.95, 16.45]
Harinasuta-1987	Thailand	MQ(750mg)+SP	1/45	2.22 [0.00, 9.29]
Harinasuta-1987	Thailand	MQ(500mg)+SP	6/44	13.64 [4.82, 25.59]
Thanh-2012	Vietnam	As+AQ	3/59	5.08 [0.65, 12.49]
Thanh-2012	Vietnam	Art+Pqp	3/55	5.45 [0.70, 13.37]
Hien-2012	Vietnam	As(12mg/kg)+DP	1/47	2.13 [0.00, 8.91]
Hien-2012	Vietnam	As(6mg/kg)+DP	1/50	2.00 [0.00, 8.39]
Hien-2012	Vietnam	DP	0/52	0.00 [0.00, 3.28]
Subtotal (RE analysis; I^2=63.1%, p<0.0001)				2.00 [1.18, 2.98]

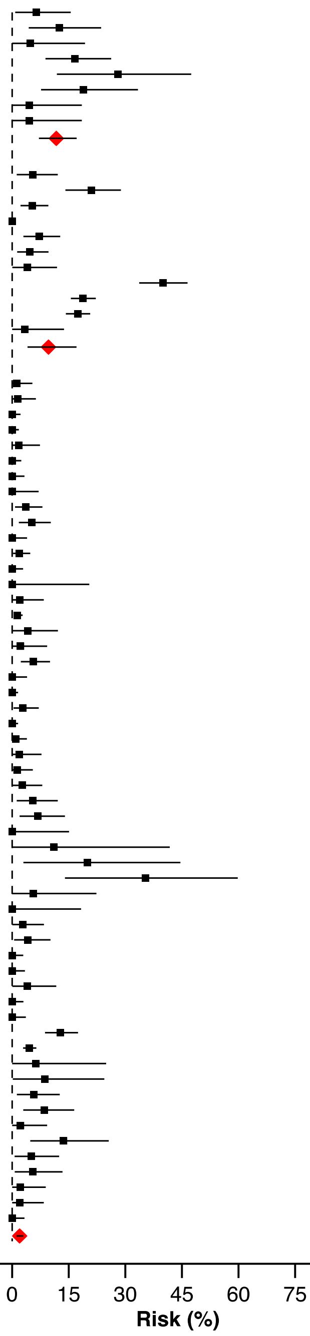

Figure S8. Day 42 risk of *P. vivax* parasitaemia following *P. falciparum* infection by drug elimination half-life for studies included in multivariable meta-regression

Meta-regression results presented in Table 3; n/N – *P. vivax* parasitaemia/total evaluable patients at day 42; CI – confidence interval; As – artesunate; Qu – quinine; Dox – doxycycline; RE – random effects; AL – artemether-lumefantrine; Az – azithromycin; At – atovaquone; Pg – proguanil; AQ – amodiaquine; SP – sulfadoxine-pyrimethamine; CQ – chloroquine; DP– dihydroartemisinin-piperaquine; AM – artesunate+mefloquine; MQ – mefloquine; Py – pyronaridine; PQ – primaquine; PNG – Papua New Guinea; Art – artemisinin; N – naphthoquine; Am – artemether; Dha – dihydroartemisinin; Pqp – piperaquine; * Study and record details are provided in Appendix, pages 14-36; † Treatment arm described by drug with number of days given (total dose) where needed to distinguish from other treatment arms; ‡ Risk – percentage of patients with *P. vivax* parasitaemia; § Asadabad, Afghanistan; ¶ Jalalabad, Afghanistan; || Multisite, Afghanistan; ** Promoy, Cambodia; †† Tasanh, Cambodia; ‡‡ Pailin City, Cambodia; Refer to Table S2 for drugs included in rapid, intermediate and slow half-life elimination categories.

Author-Year *	Country	Treatment†	n/N	Risk‡, % (95% CI)
**Rapidly eliminated**				
Duarte-1996	Brazil	As+Tet	13/72	18.06 [9.92, 27.88]
Duarte-1996	Brazil	Qu+Tet	8/69	11.59 [4.95, 20.36]
de Alencar-1997	Brazil	Qu+Tet	8/77	10.39 [4.41, 18.33]
Bethell-2011	Cambodia	As(28mg/kg)	2/38	5.26 [0.09, 15.21]
Bethell-2011	Cambodia	As(42mg/kg)	6/25	24.00 [8.95, 42.95]
Bethell-2011	Cambodia	As(14mg/kg)	6/73	8.22 [2.83, 15.80]
Mohapatra-1996	India	At	2/30	6.67 [0.12, 19.06]
Mayxay-2012b	Laos	As(6mg/kg)	0/22	0.00 [0.00, 7.67]
Mayxay-2012b	Laos	As(12mg/kg)	1/22	4.55 [0.00, 18.49]
Kyaw-2013	Myanmar	As	25/52	48.08 [34.54, 61.75]
Salazar-1990	Philippines	Cn(3000mg)	0/7	0.00 [0.00, 23.19]
Salazar-1990	Philippines	Cn(community)	1/18	5.56 [0.00, 22.32]
Salazar-1990	Philippines	Cn(6000mg)	1/9	11.11 [0.00, 41.77]
Bunnag-1991c	Thailand	Am	1/5	20.00 [0.00, 67.51]
Karbwang-1992	Thailand	Am	9/30	30.00 [14.72, 47.79]
Karbwang-1995a	Thailand	Am	10/50	20.00 [9.92, 32.36]
Looareesuwan-1997a	Thailand	Am5	15/38	39.47 [24.40, 55.59]
Looareesuwan-1997a	Thailand	Am7	23/49	46.94 [33.06, 61.06]
Bunnag-1991c	Thailand	As2(600mg)	0/5	0.00 [0.00, 31.73]
Bunnag-1991c	Thailand	As1(600mg)	0/5	0.00 [0.00, 31.73]
Bunnag-1991c	Thailand	As3(300mg)	0/5	0.00 [0.00, 31.73]
Bunnag-1991c	Thailand	As5(1200mg)	1/5	20.00 [0.00, 67.51]
Bunnag-1991c	Thailand	As5(600mg)	2/10	20.00 [0.54, 51.32]
Bunnag-1991c	Thailand	As5(650mg)	8/20	40.00 [19.35, 62.52]
Karbwang-1994	Thailand	As	5/30	16.67 [5.10, 32.43]
Looareesuwan-1997b	Thailand	As5	5/42	11.90 [3.55, 23.72]
Looareesuwan-1997b	Thailand	As7	8/40	20.00 [8.84, 34.00]
Looareesuwan-1994	Thailand	As+Dox	16/49	32.65 [20.13, 46.51]
Looareesuwan-1996a	Thailand	Dha	8/49	16.33 [7.11, 28.16]
Pukrittayakamee-2000	Thailand	Qu	12/53	22.64 [12.25, 34.99]
Segal-1974	Thailand	Qu	1/22	4.55 [0.00, 18.49]
Pukrittayakamee-2000	Thailand	Qu+Cn	12/60	20.00 [10.72, 31.17]
Karbwang-1994	Thailand	Qu+Tet	9/30	30.00 [14.72, 47.79]
Pukrittayakamee-2000	Thailand	Qu+Tet	9/48	18.75 [8.79, 31.18]
Subtotal (RE analysis; I^2=71.8%, p<0.0001)				16.29 [11.88, 21.15]
**Intermediately eliminated**				
Itoh-2018	Brazil	AL	1/75	1.33 [0.00, 5.64]
de Alencar-1997	Brazil	At+Pg	4/77	5.19 [1.14, 11.50]
Denis-2006b	Cambodia	AL(plus food)	1/77	1.30 [0.00, 5.49]
Denis-2006b	Cambodia	AL	2/49	4.08 [0.07, 11.91]
Jiao-1997	China	AL	0/102	0.00 [0.00, 1.68]
Assefa-2010	Ethiopia	AL	5/85	5.88 [1.69, 12.07]
Hwang-2011	Ethiopia	AL	3/115	2.61 [0.33, 6.51]
Mekonnen-2015	Ethiopia	AL	1/89	1.12 [0.00, 4.76]
Teklemariam-2017	Ethiopia	AL	2/81	2.47 [0.04, 7.30]
Wudneh-2016	Ethiopia	AL	1/82	1.22 [0.00, 5.16]
Mayxay-2012a	Laos	AL	18/541	3.33 [1.96, 5.02]
Thapa-2007	Nepal	AL	0/66	0.00 [0.00, 2.59]
Lefevre-2001	Thailand	AL	6/155	3.87 [1.30, 7.58]
Looareesuwan-1999	Thailand	At+Pg	8/79	10.13 [4.30, 17.88]
Sabchareon-1998	Thailand	At+Pg	2/28	7.14 [0.13, 20.34]
Krudsood-2001	Thailand	Hal3(3000mg)	0/28	0.00 [0.00, 6.05]
Krudsood-2001	Thailand	Hal7(4500mg)	0/33	0.00 [0.00, 5.15]
Krudsood-2001	Thailand	Hal3(4500mg)	2/35	5.71 [0.10, 16.46]
Subtotal (RE analysis; I^2=45.1%, p=0.0302)				2.35 [1.30, 3.64]
**Slowly eliminated**				
Awab-2016	Afghanistan	As+SP§	0/78	0.00 [0.00, 2.19]
Awab-2016	Afghanistan	As+SP¶	0/97	0.00 [0.00, 1.76]
Awab-2016	Afghanistan	As+SP‖	1/58	1.72 [0.00, 7.25]
Awab-2016	Afghanistan	DP	0/53	0.00 [0.00, 3.22]
Denis-2002	Cambodia	DP**	0/58	0.00 [0.00, 2.94]
Denis-2002	Cambodia	DP††	1/39	2.56 [0.00, 10.68]
Denis-2006b	Cambodia	AM	0/52	0.00 [0.00, 3.28]
Tjitra-2001	Indonesia	As+SP	0/47	0.00 [0.00, 3.63]
Fryauff-2002	Indonesia	SP	3/38	7.89 [1.04, 19.05]
Tjitra-2001	Indonesia	SP	0/46	0.00 [0.00, 3.70]
Thapa-2007	Nepal	SP	0/33	0.00 [0.00, 5.15]
Karunajeewa-2008b	PNG	CQ+SP	6/17	35.29 [14.00, 59.80]
Karunajeewa-2008b	PNG	DP	0/18	0.00 [0.00, 9.34]
Bunnag-1991c	Thailand	As+CQ	0/5	0.00 [0.00, 31.73]
Ashley-2004	Thailand	As+DP	0/59	0.00 [0.00, 2.89]
Bunnag-1991c	Thailand	As+SP	0/5	0.00 [0.00, 31.73]
Ashley-2004	Thailand	DP(6/48 mg/kg)	0/59	0.00 [0.00, 2.89]
Wilairatana-2002	Thailand	DP+Tri+PQ	2/194	1.03 [0.02, 3.08]
Karbwang-1992	Thailand	MQ	1/9	11.11 [0.00, 41.77]
Looareesuwan-1999	Thailand	MQ	0/79	0.00 [0.00, 2.16]
Karbwang-1995a	Thailand	Am+MQ	2/50	4.00 [0.07, 11.68]
Looareesuwan-1997a	Thailand	Am+MQ	0/44	0.00 [0.00, 3.87]
Ashley-2004	Thailand	AM	0/59	0.00 [0.00, 2.89]
Lefevre-2001	Thailand	AM	0/53	0.00 [0.00, 3.22]
Tangpukdee-2008	Thailand	AM	0/65	0.00 [0.00, 2.63]
Wilairatana-1998	Thailand	AM	9/130	6.92 [3.10, 12.01]
Wilairatana-2002	Thailand	AM	0/82	0.00 [0.00, 2.09]
Looareesuwan-1994	Thailand	MQ+Dox	1/48	2.08 [0.00, 8.73]
Tangpukdee-2008	Thailand	Art+Pqp+PQ	0/64	0.00 [0.00, 2.67]
Looareesuwan-1996c	Thailand	Py(1800mg)	0/26	0.00 [0.00, 6.51]
Looareesuwan-1996c	Thailand	Py(1200mg)	1/60	1.67 [0.00, 7.02]
Subtotal (RE analysis; I^2=41.7%, p=0.0019)				0.22 [0.00, 0.91]

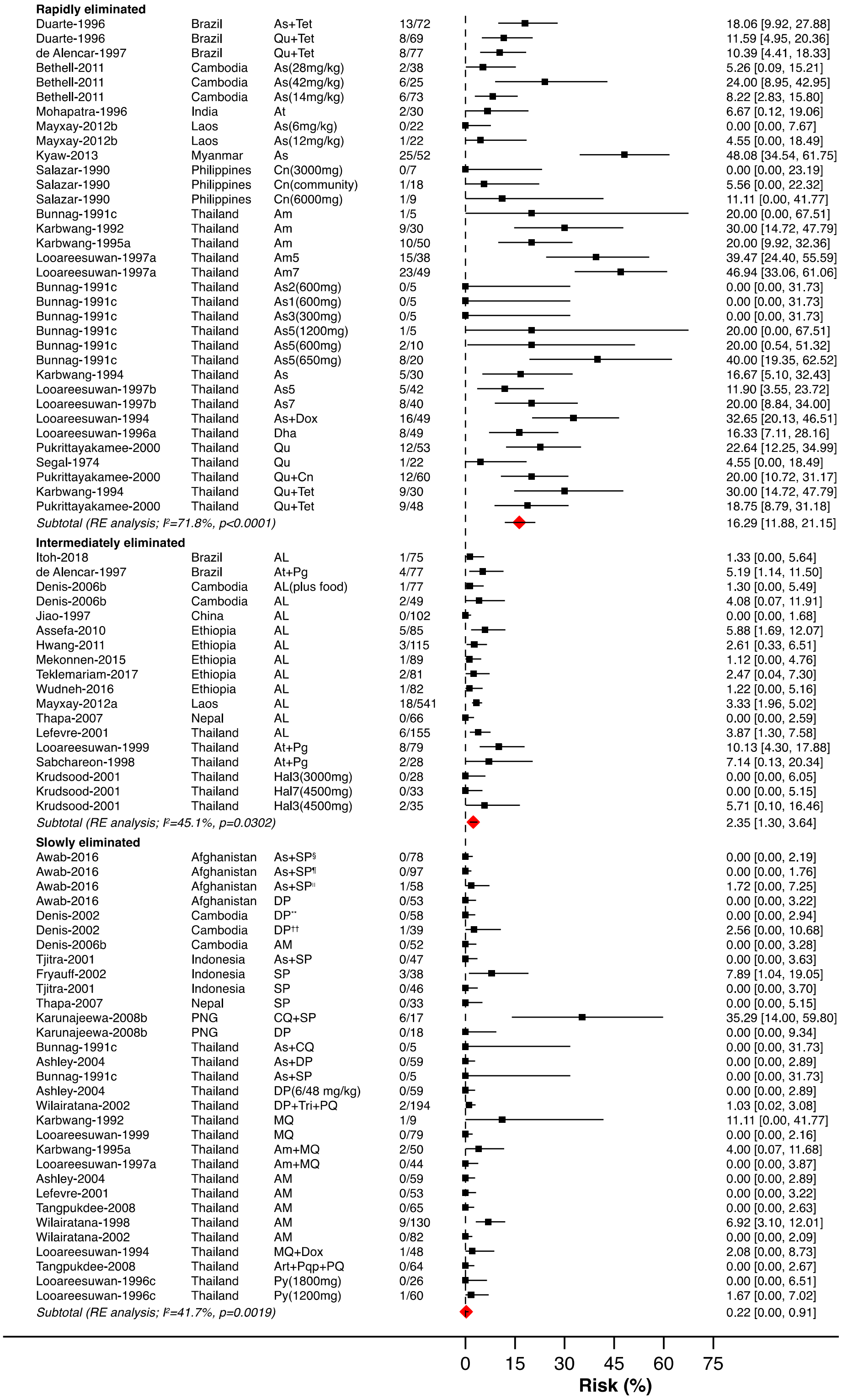

Figure S9. Day 28 risk of *P. vivax* parasitaemia following *P. falciparum* infection by drug elimination half-life for studies included in multivariable meta-regression

Meta-regression results presented in Table S11; n/N – *P. vivax* parasitaemia/total evaluable patients at day 28; CI – confidence interval; As – artesunate; Tet – tetracycline; Qu – quinine; At – atovaquone; Cn – clindamycin; Am – artemether; Dox – doxycycline; Dha – Dihydroartemisinin; RE – random effects; AL – artemether-lumefantrine; Pg – proguanil; Hal – halofantrine; SP – sulfadoxine- pyrimethamine; DP– dihydroartemisinin-piperaquine; AM – artesunate+mefloquine; PNG – Papua New Guinea; CQ – chloroquine; Tri – trimethoprim; PQ – primaquine; MQ – mefloquine; Pqp – piperaquine; Py – pyronaridine; * Study and record details are provided in Appendix, pages 14-36; † Treatment arm described by drug with number of days given (total dose) where needed to distinguish from other treatment arms; ‡ Risk – percentage of patients with *P. vivax* parasitaemia; § Asadabad, Afghanistan; ¶ Jalalabad, Afghanistan; || Multisite, Afghanistan; ** Oddor Meanchey, Cambodia; †† Snoul, Cambodia; Refer to Table S2 for drugs included in rapid, intermediate and slow half-life elimination categories.

Author-Year*	Country	Treatment†	n/N	Risk‡, % (95% CI)
**Hb <11 g/dL**				
Denis-2002	Cambodia	DP§	0/58	0.00 [0.00, 2.94]
Denis-2002	Cambodia	DP¶	1/39	2.56 [0.00, 10.68]
Denis-2006b	Cambodia	AM	0/52	0.00 [0.00, 3.28]
Mohapatra-1996	India	At	2/30	6.67 [0.12, 19.06]
Tjitra-2001	Indonesia	As+SP	0/47	0.00 [0.00, 3.63]
Fryauff-2002	Indonesia	SP	3/38	7.89 [1.04, 19.05]
Tjitra-2001	Indonesia	SP	0/46	0.00 [0.00, 3.70]
Karunajeewa-2008b	PNG	CQ+SP	6/17	35.29 [14.00, 59.80]
Karunajeewa-2008b	PNG	DP	0/18	0.00 [0.00, 9.34]
Salazar-1990	Philippines	Cn(community)	1/18	5.56 [0.00, 22.32]
Karbwang-1995a	Thailand	Am	10/50	20.00 [9.92, 32.36]
Looareesuwan-1997b	Thailand	As5	5/42	11.90 [3.55, 23.72]
Looareesuwan-1997b	Thailand	As7	8/40	20.00 [8.84, 34.00]
Sabchareon-1998	Thailand	At+Pg	2/28	7.14 [0.13, 20.34]
Segal-1974	Thailand	Qu	1/22	4.55 [0.00, 18.49]
Segal-1974	Thailand	WR33063	3/25	12.00 [1.65, 28.20]
Subtotal (RE analysis; I^2=75.0%, p<0.0001)				5.52 [1.93, 10.39]
**Hb >=11 g/dL**				
Awab-2016	Afghanistan	As+SP‖	0/78	0.00 [0.00, 2.19]
Awab-2016	Afghanistan	As+SP**	0/97	0.00 [0.00, 1.76]
Awab-2016	Afghanistan	As+SP††	1/58	1.72 [0.00, 7.25]
Awab-2016	Afghanistan	DP	0/53	0.00 [0.00, 3.22]
Itoh-2018	Brazil	AL	1/75	1.33 [0.00, 5.64]
Duarte-1996	Brazil	As+Tet	13/72	18.06 [9.92, 27.88]
de Alencar-1997	Brazil	At+Pg	4/77	5.19 [1.14, 11.50]
Duarte-1996	Brazil	Qu+Tet	8/69	11.59 [4.95, 20.36]
de Alencar-1997	Brazil	Qu+Tet	8/77	10.39 [4.41, 18.33]
Denis-2006b	Cambodia	AL(plus food)	1/77	1.30 [0.00, 5.49]
Denis-2006b	Cambodia	AL	2/49	4.08 [0.07, 11.91]
Bethell-2011	Cambodia	As(28mg/kg)	2/38	5.26 [0.09, 15.21]
Bethell-2011	Cambodia	As(42mg/kg)	6/25	24.00 [8.95, 42.95]
Bethell-2011	Cambodia	As(14mg/kg)	6/73	8.22 [2.83, 15.80]
Jiao-1997	China	AL	0/102	0.00 [0.00, 1.68]
Assefa-2010	Ethiopia	AL	5/85	5.88 [1.69, 12.07]
Hwang-2011	Ethiopia	AL	3/115	2.61 [0.33, 6.51]
Mekonnen-2015	Ethiopia	AL	1/89	1.12 [0.00, 4.76]
Teklemariam-2017	Ethiopia	AL	2/81	2.47 [0.04, 7.30]
Wudneh-2016	Ethiopia	AL	1/82	1.22 [0.00, 5.16]
Mayxay-2012a	Laos	AL	18/541	3.33 [1.96, 5.02]
Mayxay-2012b	Laos	As(6mg/kg)	0/22	0.00 [0.00, 7.67]
Mayxay-2012b	Laos	As(12mg/kg)	1/22	4.55 [0.00, 18.49]
Kyaw-2013	Myanmar	As	25/52	48.08 [34.54, 61.75]
Thapa-2007	Nepal	AL	0/66	0.00 [0.00, 2.59]
Thapa-2007	Nepal	SP	0/33	0.00 [0.00, 5.15]
Marquino-2005	Peru	As+/-SP	1/186	0.54 [0.00, 2.29]
Salazar-1990	Philippines	Cn(3000mg)	0/7	0.00 [0.00, 23.19]
Salazar-1990	Philippines	Cn(6000mg)	1/9	11.11 [0.00, 41.77]
Bunnag-1991c	Thailand	Am	1/5	20.00 [0.00, 67.51]
Karbwang-1992	Thailand	Am	9/30	30.00 [14.72, 47.79]
Looareesuwan-1997a	Thailand	Am5	15/38	39.47 [24.40, 55.59]
Looareesuwan-1997a	Thailand	Am7	23/49	46.94 [33.06, 61.06]
Lefevre-2001	Thailand	AL	6/155	3.87 [1.30, 7.58]
Bunnag-1991c	Thailand	As3(300mg)	0/5	0.00 [0.00, 31.73]
Bunnag-1991c	Thailand	As2(600mg)	0/5	0.00 [0.00, 31.73]
Bunnag-1991c	Thailand	As1(600mg)	0/5	0.00 [0.00, 31.73]
Bunnag-1991c	Thailand	As5(1200mg)	1/5	20.00 [0.00, 67.51]
Bunnag-1991c	Thailand	As5(600mg)	2/10	20.00 [0.54, 51.32]
Bunnag-1991c	Thailand	As5(650mg)	8/20	40.00 [19.35, 62.52]
Karbwang-1994	Thailand	As	5/30	16.67 [5.10, 32.43]
Bunnag-1991c	Thailand	As+CQ	0/5	0.00 [0.00, 31.73]
Ashley-2004	Thailand	As+DP	0/59	0.00 [0.00, 2.89]
Looareesuwan-1994	Thailand	As+Dox	16/49	32.65 [20.13, 46.51]
Bunnag-1991c	Thailand	As+SP	0/5	0.00 [0.00, 31.73]
Looareesuwan-1999	Thailand	At+Pg	8/79	10.13 [4.30, 17.88]
Looareesuwan-1996a	Thailand	Dha	8/49	16.33 [7.11, 28.16]
Ashley-2004	Thailand	DP(6/48 mg/kg)	0/59	0.00 [0.00, 2.89]
Wilairatana-2002	Thailand	DP+Tri+PQ	2/194	1.03 [0.02, 3.08]
Krudsood-2001	Thailand	Hal3(3000mg)	0/28	0.00 [0.00, 6.05]
Krudsood-2001	Thailand	Hal7(4500mg)	0/33	0.00 [0.00, 5.15]
Krudsood-2001	Thailand	Hal3(4500mg)	2/35	5.71 [0.10, 16.46]
Karbwang-1992	Thailand	MQ	1/9	11.11 [0.00, 41.77]
Looareesuwan-1999	Thailand	MQ	0/79	0.00 [0.00, 2.16]
Karbwang-1995a	Thailand	Am+MQ	2/50	4.00 [0.07, 11.68]
Looareesuwan-1997a	Thailand	Am+MQ	0/44	0.00 [0.00, 3.87]
Ashley-2004	Thailand	AM	0/59	0.00 [0.00, 2.89]
Lefevre-2001	Thailand	AM	0/53	0.00 [0.00, 3.22]
Tangpukdee-2008	Thailand	AM	0/65	0.00 [0.00, 2.63]
Wilairatana-1998	Thailand	AM	9/130	6.92 [3.10, 12.01]
Wilairatana-2002	Thailand	AM	0/82	0.00 [0.00, 2.09]
Looareesuwan-1994	Thailand	MQ+Dox	1/48	2.08 [0.00, 8.73]
Tangpukdee-2008	Thailand	Art+Pqp+PQ	0/64	0.00 [0.00, 2.67]
Looareesuwan-1996c	Thailand	Py(1800mg)	0/26	0.00 [0.00, 6.51]
Looareesuwan-1996c	Thailand	Py(1200mg)	1/60	1.67 [0.00, 7.02]
Pukrittayakamee-2000	Thailand	Qu	12/53	22.64 [12.25, 34.99]
Pukrittayakamee-2000	Thailand	Qu+Cn	12/60	20.00 [10.72, 31.17]
Karbwang-1994	Thailand	Qu+Tet	9/30	30.00 [14.72, 47.79]
Pukrittayakamee-2000	Thailand	Qu+Tet	9/48	18.75 [8.79, 31.18]
Subtotal (RE analysis; I^2=80.0%, p<0.0001)				4.58 [2.47, 7.13]

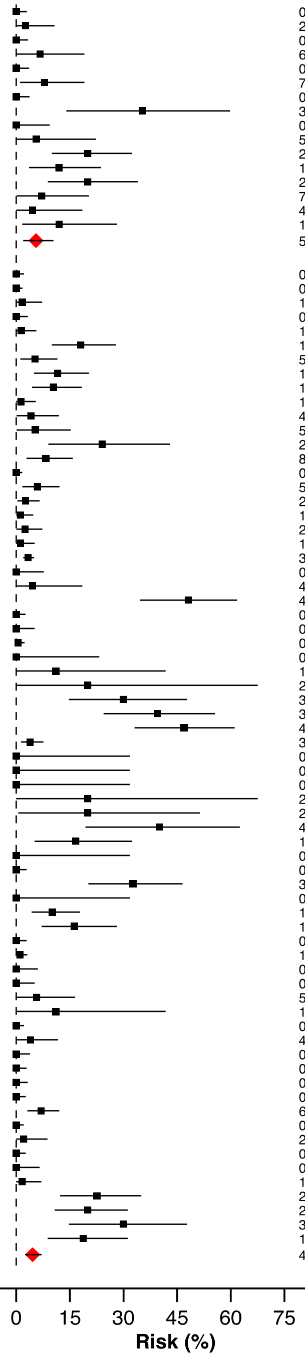

Figure S10. Day 28 risk of *P. vivax* parasitaemia following *P. falciparum* infection by haemoglobin for studies included in multivariable meta-regression

Meta-regression results presented in Table S11; n/N – *P. vivax* parasitaemia/total evaluable patients at day 28; CI – confidence interval; DP– dihydroartemisinin-piperaquine; AM – artesunate+mefloquine; At – atovaquone; As – artesunate; SP – sulfadoxine- pyrimethamine; PNG – Papua New Guinea; CQ – chloroquine; Cn – clindamycin; Am – artemether; Pg – proguanil; Qu – quinine; RE – random effects; AL – artemether-lumefantrine; Tet – tetracycline; Dox – doxycycline; Dha – Dihydroartemisinin; Tri – trimethoprim; PQ – primaquine; Hal – halofantrine; MQ – mefloquine; Art – artemisinin; Pqp – piperaquine; Py – pyronaridine; * Study and record details are provided in Appendix, pages 14-36; † Treatment arm described by drug with number of days given (total dose) where needed to distinguish from other treatment arms; ‡ Risk – percentage of patients with *P. vivax* parasitaemia; § Oddor Meanchey, Cambodia; ¶ Snoul, Cambodia; || Asadabad, Afghanistan; ** Jalalabad, Afghanistan; †† Multisite, Afghanistan.

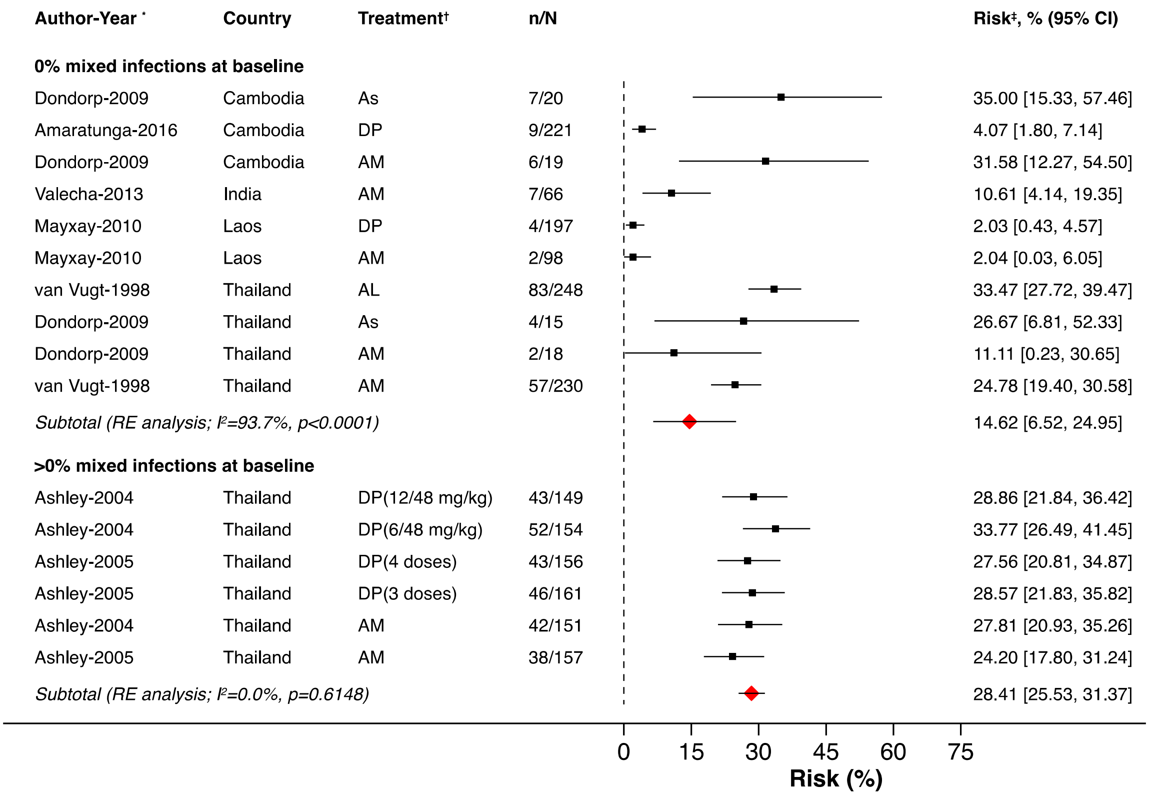

Figure S11. Day 63 risk of *P. vivax* parasitaemia following *P. falciparum* infection by percentage of mixed infections at baseline for studies included in multivariable meta-regression

Meta-regression results presented in Table S12; n/N – *P. vivax* parasitaemia/total evaluable patients at day 63; CI – confidence interval; As – artesunate; DP– dihydroartemisinin-piperaquine; AM – artesunate+mefloquine; AL – artemether-lumefantrine; RE – random effects; * Study and record details are provided in Appendix, pages 14-36; † Treatment arm described by drug with number of days given (total dose) where needed to distinguish from other treatment arms; ‡ Risk – percentage of patients with *P. vivax* parasitaemia.

Appendix 12. Day 42 multivariable meta-regression sensitivity analysis

Table S10. Multivariable meta-regression of the risk of *P. vivax* parasitaemia at day 42 following *P. falciparum* infection including baseline haemoglobin as a covariate

Variable	Multivariable‡	
	Coefficient (95% CI)	p value
Overall		
Baseline haemoglobin (mean, g/dL)	-0·05 (-0·08, -0·01)	0·0067
Female (%, per every 10%)	-0·03 (-0·05, 0·00)	0·0225
Mixed infection (%, per every 10%)	0·08 (-0·01, 0·15)	0·0814
Baseline parasitaemia (per every 10-fold increase in mean)	-0·11 (-0·21, 0·00)	0·0512
Short relapse periodicity§	-0·11 (-0·19, -0·04)	0·0019
Drug elimination half life		<0·0001
Rapid	Referent	
Intermediate	-0·03 (-0·17, 0·10)	
Slow	-0·28 (-0·39, -0·16)	
Year data collected (per every 5-year increase)	-0·01 (-0·04, 0·01)	0·2961

CI – Confidence Interval; Results from the univariable meta-regression expressed as $\theta_i = \beta_0 + b_i + \beta_1 x_i + \varepsilon_i$, where θ_i is the Freeman-Tukey double arcsine transformed treatment completion rate from record i, β_0 is the intercept, b_i is the random effect for record i, x_i is the value of the covariate from study i, and ε_i is the within study error; * The between- study variance (τ^2) from a meta-regression model with no covariates can be compared to τ^2 from univariable meta-regression models to estimate how much variation each covariate explains. For categorical covariates τ^2 is provided for the overall covariate; † For categorical covariates, variance explained is provided for the overall covariate; ‡ Meta-regression includes 61 records, I^2=70·53%, τ^2=0·0062, R^2=66·94%; Similar analysis to Table 3 except baseline haemoglobin was included in the model and age was not. Age was not included due to strong correlation with baseline haemoglobin (Pearson correlation coefficient 0·86 (p<0·0001)); Region was not included in model due to correlation with relapse periodicity; Gametocyte percentage was not included in model due to low availability of data; § Short relapse periodicity is referenced against long relapse periodicity

Appendix 13. Forest plots of overall pooled estimates including data from all available records

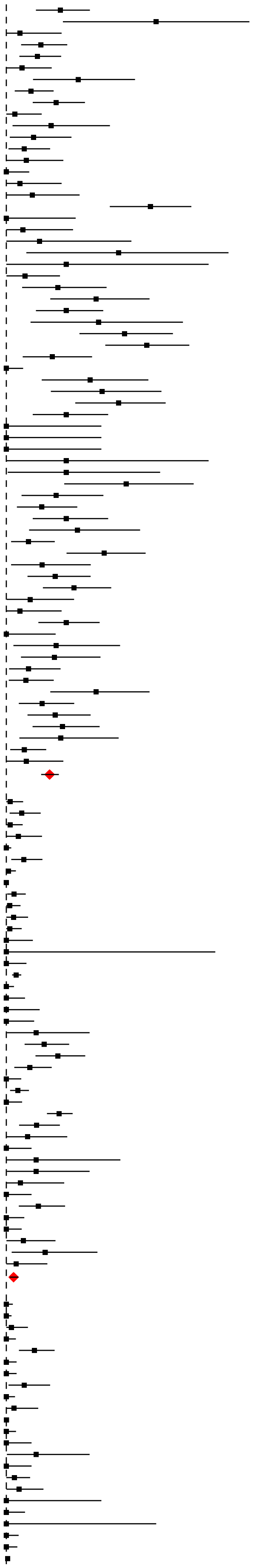

Author-Year [*]	Country	Treatment[†]	n/N	Risk[‡], % (95% CI)
**Rapidly eliminated**				
Duarte-1996	Brazil	As+Tet	13/72	18.06 [9.92, 27.88]
Kremsner-1989	Brazil	Cn	5/10	50.00 [18.92, 81.08]
Fontes-2002	Brazil	Pg+Sz	1/22	4.55 [0.00, 18.49]
Duarte-1996	Brazil	Qu+Tet	8/69	11.59 [4.95, 20.36]
de Alencar-1997	Brazil	Qu+Tet	8/77	10.39 [4.41, 18.33]
Bethell-2011	Cambodia	As(28mg/kg)	2/38	5.26 [0.09, 15.21]
Bethell-2011	Cambodia	As(42mg/kg)	6/25	24.00 [8.95, 42.95]
Bethell-2011	Cambodia	As(14mg/kg)	6/73	8.22 [2.83, 15.80]
Noedl-2010	Cambodia	As	12/72	16.67 [8.84, 26.26]
Noedl-2010	Cambodia	Qu+Tet	1/35	2.86 [0.00, 11.86]
Li-1984	China	Art	3/20	15.00 [2.13, 34.57]
Oemijati-1994	East Timor	Cn	3/33	9.09 [1.21, 21.77]
Rahman-2016	Guyana	As+PQ	3/50	6.00 [0.78, 14.65]
Mohapatra-1996	India	At	2/30	6.67 [0.12, 19.06]
Mayxay-2012b	Laos	As(6mg/kg)	0/22	0.00 [0.00, 7.67]
Mayxay-2012b	Laos	As(12mg/kg)	1/22	4.55 [0.00, 18.49]
Pe-1986	Myanmar	Am	2/23	8.70 [0.17, 24.46]
Kyaw-2013	Myanmar	As	25/52	48.08 [34.54, 61.75]
Salazar-1990	Philippines	Cn(3000mg)	0/7	0.00 [0.00, 23.19]
Salazar-1990	Philippines	Cn(community)	1/18	5.56 [0.00, 22.32]
Salazar-1990	Philippines	Cn(6000mg)	1/9	11.11 [0.00, 41.77]
Na Bangchang-1994	Thailand	Am	3/8	37.50 [6.72, 74.11]
Bunnag-1991c	Thailand	Am	1/5	20.00 [0.00, 67.51]
Bunnag-1992b	Thailand	Am(480mg)	2/32	6.25 [0.11, 17.92]
Bunnag-1992b	Thailand	Am(600mg)	5/29	17.24 [5.30, 33.46]
Karbwang-1992	Thailand	Am	9/30	30.00 [14.72, 47.79]
Karbwang-1995a	Thailand	Am	10/50	20.00 [9.92, 32.36]
Karbwang-1998	Thailand	Am	4/13	30.77 [8.11, 58.93]
Looareesuwan-1997a	Thailand	Am5	15/38	39.47 [24.40, 55.59]
Looareesuwan-1997a	Thailand	Am7	23/49	46.94 [33.06, 61.06]
Thimarsarn-1997	Thailand	Am	6/39	15.38 [5.49, 28.65]
Na Bangchang-1996b	Thailand	Am+Dox	0/30	0.00 [0.00, 5.65]
Bunnag-1991a	Thailand	As(2x daily)	7/25	28.00 [11.85, 47.45]
Bunnag-1991a	Thailand	As(1x daily)	8/25	32.00 [14.91, 51.78]
Bunnag-1991b	Thailand	As5	15/40	37.50 [23.03, 53.15]
Bunnag-1991b	Thailand	As7	8/40	20.00 [8.84, 34.00]
Bunnag-1991c	Thailand	As1(600mg)	0/5	0.00 [0.00, 31.73]
Bunnag-1991c	Thailand	As2(600mg)	0/5	0.00 [0.00, 31.73]
Bunnag-1991c	Thailand	As3(300mg)	0/5	0.00 [0.00, 31.73]
Bunnag-1991c	Thailand	As5(1200mg)	1/5	20.00 [0.00, 67.51]
Bunnag-1991c	Thailand	As5(600mg)	2/10	20.00 [0.54, 51.32]
Bunnag-1991c	Thailand	As5(650mg)	8/20	40.00 [19.35, 62.52]
Karbwang-1994	Thailand	As	5/30	16.67 [5.10, 32.43]
Looareesuwan-1997b	Thailand	As5	5/42	11.90 [3.55, 23.72]
Looareesuwan-1997b	Thailand	As7	8/40	20.00 [8.84, 34.00]
Pukrittayakamee-2004	Thailand	As	5/21	23.81 [7.61, 44.69]
Thimarsarn-1997	Thailand	As	4/54	7.41 [1.65, 16.20]
Looareesuwan-1994	Thailand	As+Dox	16/49	32.65 [20.13, 46.51]
Pukrittayakamee-2004	Thailand	As+PQ	3/25	12.00 [1.65, 28.20]
Looareesuwan-1996a	Thailand	Dha	8/49	16.33 [7.11, 28.16]
Pukrittayakamee-2000	Thailand	Qu	12/53	22.64 [12.25, 34.99]
Pukrittayakamee-2004	Thailand	Qu	2/25	8.00 [0.15, 22.62]
Segal-1974	Thailand	Qu	1/22	4.55 [0.00, 18.49]
Pukrittayakamee-2000	Thailand	Qu+Cn	12/60	20.00 [10.72, 31.17]
Miller-2006	Thailand	Qu+Dox	0/10	0.00 [0.00, 16.52]
Pukrittayakamee-2004	Thailand	Qu+PQ(0.25mg)	3/18	16.67 [2.40, 38.00]
Pukrittayakamee-2004	Thailand	Qu+PQ(0.5mg)	5/31	16.13 [4.92, 31.47]
Bunnag-1996a	Thailand	Qu7+Tet	3/40	7.50 [0.98, 18.14]
Bunnag-1996a	Thailand	Qu5+Tet	3/46	6.52 [0.85, 15.87]
Karbwang-1994	Thailand	Qu+Tet	9/30	30.00 [14.72, 47.79]
Meek-1986	Thailand	Qu3+Tet	6/50	12.00 [4.21, 22.69]
Meek-1986	Thailand	Qu7+Tet	8/49	16.33 [7.11, 28.16]
Pukrittayakamee-2000	Thailand	Qu+Tet	9/48	18.75 [8.79, 31.18]
Pukrittayakamee-2004	Thailand	Qu+Tet	4/22	18.18 [4.36, 37.47]
Thimarsarn-1997	Thailand	Qu+Tet	4/66	6.06 [1.33, 13.35]
Phong-2016	Vietnam	As	2/30	6.67 [0.12, 19.06]
Subtotal (RE analysis; I^2=68.0%, p<0.0001)				14.50 [11.66, 17.56]
**Intermediately eliminated**				
Itoh-2018	Brazil	AL	1/75	1.33 [0.00, 5.64]
de Alencar-1997	Brazil	At+Pg	4/77	5.19 [1.14, 11.50]
Denis-2006b	Cambodia	AL(plus food)	1/77	1.30 [0.00, 5.49]
Denis-2006b	Cambodia	AL	2/49	4.08 [0.07, 11.91]
Jiao-1997	China	AL	0/102	0.00 [0.00, 1.68]
Assefa-2010	Ethiopia	AL	5/85	5.88 [1.69, 12.07]
Ebstie-2015	Ethiopia	AL	1/131	0.76 [0.00, 3.25]
Eshetu-2012	Ethiopia	AL	0/317	0.00 [0.00, 0.54]
Hwang-2011	Ethiopia	AL	3/115	2.61 [0.33, 6.51]
Mekonnen-2015	Ethiopia	AL	1/89	1.12 [0.00, 4.76]
Teklemariam-2017	Ethiopia	AL	2/81	2.47 [0.04, 7.30]
Wudneh-2016	Ethiopia	AL	1/82	1.22 [0.00, 5.16]
Valecha-2012	India	AL[§]	0/19	0.00 [0.00, 8.86]
Valecha-2012	India	AL[¶]	0/2	0.00 [0.00, 69.73]
Valecha-2012	India	AL[‖]	0/25	0.00 [0.00, 6.77]
Mayxay-2012a	Laos	AL	18/541	3.33 [1.96, 5.02]
Thapa-2007	Nepal	AL	0/66	0.00 [0.00, 2.59]
Na Bangchang-1996b	Thailand	Am+Az	0/27	0.00 [0.00, 6.27]
Na Bangchang-1996a	Thailand	Am1+P	0/15	0.00 [0.00, 11.16]
Na Bangchang-1996a	Thailand	Am2+P	0/18	0.00 [0.00, 9.34]
Na Bangchang-1996a	Thailand	Am3+P	2/20	10.00 [0.20, 27.83]
Karbwang-2000	Thailand	AL2(240/1440mg)	10/79	12.66 [6.11, 21.01]
Karbwang-2000	Thailand	AL3(320/1920mg)	14/81	17.28 [9.74, 26.37]
Karbwang-2000	Thailand	AL3(160/960mg)	6/76	7.89 [2.71, 15.20]
Krudsood-2003	Thailand	AL	0/34	0.00 [0.00, 5.00]
Lefevre-2001	Thailand	AL	6/155	3.87 [1.30, 7.58]
Valecha-2012	Thailand	AL	0/32	0.00 [0.00, 5.31]
van Vugt-1999	Thailand	AL	54/306	17.65 [13.57, 22.13]
Looareesuwan-1999	Thailand	At+Pg	8/79	10.13 [4.30, 17.88]
Sabchareon-1998	Thailand	At+Pg	2/28	7.14 [0.13, 20.34]
Miller-2006	Thailand	Az+Qu(5000mg)	0/20	0.00 [0.00, 8.43]
Miller-2006	Thailand	Az+Qu(3000mg)	1/10	10.00 [0.00, 38.09]
Miller-2006	Thailand	Az+Qu(4500mg)	2/20	10.00 [0.20, 27.83]
Segal-1975	Thailand	Dap+P	1/21	4.76 [0.00, 19.32]
Boudreau-1988	Thailand	Hal(2 doses)	0/20	0.00 [0.00, 8.43]
Boudreau-1988	Thailand	Hal(2 doses)	7/65	10.77 [4.21, 19.63]
Krudsood-2001	Thailand	Hal3(3000mg)	0/28	0.00 [0.00, 6.05]
Krudsood-2001	Thailand	Hal7(4500mg)	0/33	0.00 [0.00, 5.15]
Krudsood-2001	Thailand	Hal3(4500mg)	2/35	5.71 [0.10, 16.46]
Yeramian-2005	Thailand	Paf	3/23	13.04 [1.81, 30.45]
Phong-2016	Vietnam	As+Az	1/30	3.33 [0.00, 13.76]
Subtotal (RE analysis; I^2=74.5%, p<0.0001)				2.46 [1.16, 4.09]
**Slowly eliminated**				
Awab-2016	Afghanistan	As+SP[**]	0/78	0.00 [0.00, 2.19]
Awab-2016	Afghanistan	As+SP[††]	0/97	0.00 [0.00, 1.76]
Awab-2016	Afghanistan	As+SP[‡‡]	1/58	1.72 [0.00, 7.25]
Awab-2016	Afghanistan	DP	0/53	0.00 [0.00, 3.22]
Cerutti-1999	Brazil	MQ	9/96	9.38 [4.24, 16.13]
de Souza-1983	Brazil	MQ	0/49	0.00 [0.00, 3.48]
de Souza-1985	Brazil	MQ	0/49	0.00 [0.00, 3.48]
de Souza-1985	Brazil	Qu+SP	3/50	6.00 [0.78, 14.65]
Denis-2002	Cambodia	DP[§§]	0/58	0.00 [0.00, 2.94]
Denis-2002	Cambodia	DP[¶¶]	1/39	2.56 [0.00, 10.68]
Denis-2006a	Cambodia	AM	1/977	0.10 [0.00, 0.44]
Denis-2006b	Cambodia	AM	0/52	0.00 [0.00, 3.28]
Li-1984	China	Art+MQ	0/20	0.00 [0.00, 8.43]
Li-1984	China	MQ+SP	2/20	10.00 [0.20, 27.83]
Li-1984	China	Art+MQ+SP	0/20	0.00 [0.00, 8.43]
Botero-1985	Colombia	MQ+/-SP	2/74	2.70 [0.04, 7.97]
Ezard-2003	East Timor	CQ	2/47	4.26 [0.07, 12.40]
Oemijati-1994	East Timor	SP	0/5	0.00 [0.00, 31.73]
Baird-2002	Guyana	CQ	0/27	0.00 [0.00, 6.27]
Valecha-2012	India	Aon+Pqp[¶]	0/3	0.00 [0.00, 50.03]
Valecha-2012	India	Aon+Pqp[§]	0/41	0.00 [0.00, 4.15]
Valecha-2012	India	Aon+Pqp[‖]	0/46	0.00 [0.00, 3.70]
Mishra-2012	India	As+SP	7/1397	0.50 [0.19, 0.95]
[illegible]	[illegible]	[illegible]	[illegible]	[illegible]

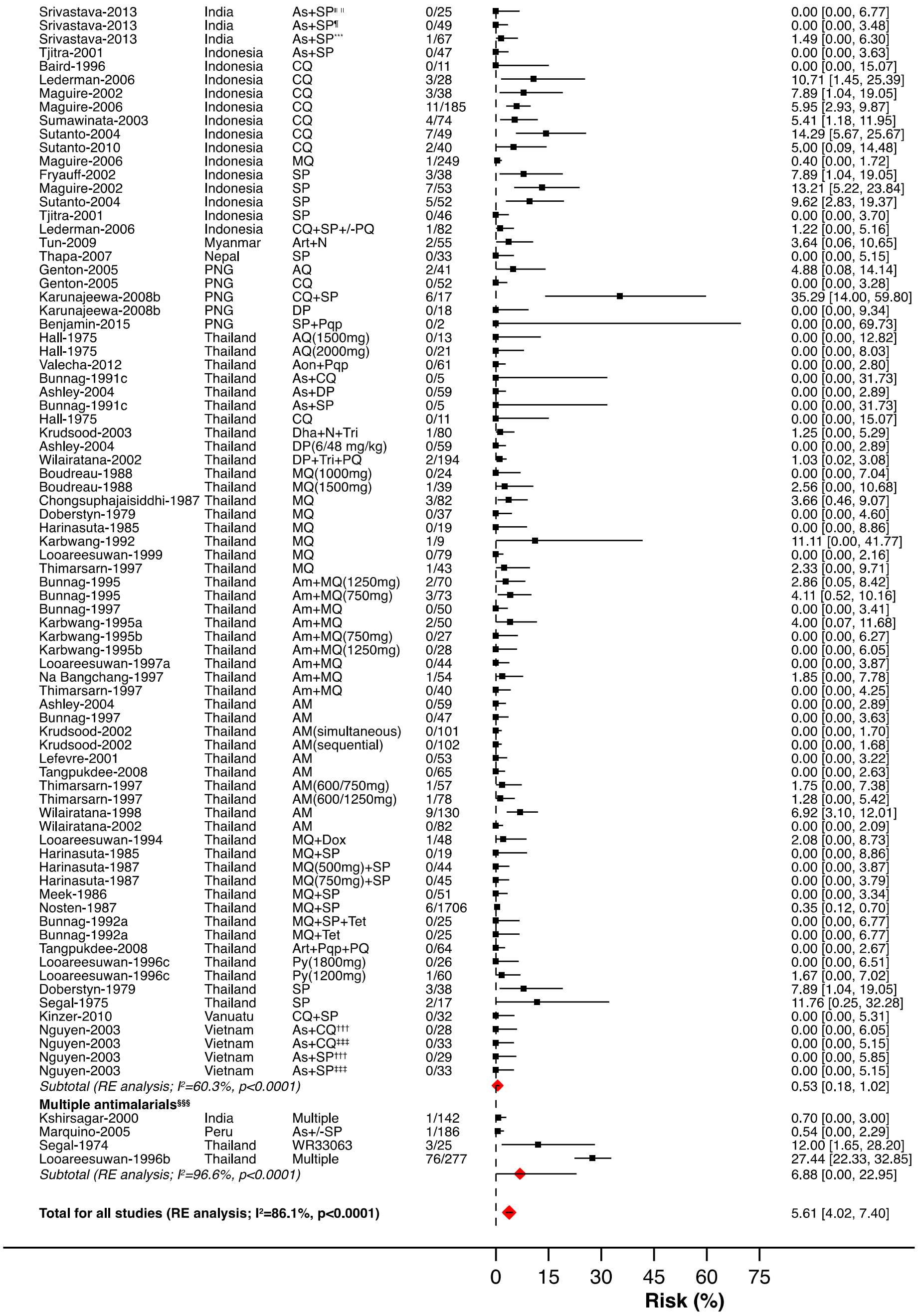

Figure S12. Risk of recurrent *P. vivax* parasitaemia by day 28 following *P. falciparum* infection by drug elimination half-life

n/N – *P. vivax* parasitaemia/total evaluable patients at day 28; CI – confidence interval; As – artesunate; Tet – tetracycline; Cn – clindamycin; Pg – proguanil; Sz – Sulfamethoxazole; Qu – quinine; Art – artemisinin; PQ – primaquine; At – atovaquone; Am – artemether; Dox – doxycycline; Dha – Dihydroartemisinin; RE – random effects; AL – artemether-lumefantrine; Az – azithromycin; P – pyrimethamine; Dap – dapsone; Hal – halofantrine; Paf – Pafuramidine; SP – sulfadoxine- pyrimethamine; DP– dihydroartemisinin-piperaquine; MQ – mefloquine; AM – artesunate+mefloquine; CQ – chloroquine; Aon – Arterolane; Pqp – piperaquine; N – naphthoquine; PNG – Papua New Guinea; AQ – amodiaquine; Tri – trimethoprim; Py – pyronaridine; * Study and record details are provided in Appendix, pages 14-36; † Treatment arm described by drug with number of days given (total dose) where needed to distinguish from other treatment arms; ‡ Risk – percentage of patients with *P. vivax* parasitaemia; § Jamshedpur, India; ¶ Ranchi, India; || Rourkela, India; ** Asadabad, Afghanistan; †† Jalalabad, Afghanistan; ‡‡ Multisite, Afghanistan; §§ Oddor Meanchey, Cambodia; ¶¶ Snoul, Cambodia; || || West Garo Hills, India; *** Orissa, India; ††† Dac Lac, Vietnam; ‡‡‡ Binh Phuoc Province, Vietnam; §§§ Multiple antimalarials – studies with aggregated treatment data where drug elimination half-life varies; Refer to Table S2 for drugs included in rapid, intermediate and slow half-life elimination categories.

Author-Year *	Country	Treatment†	n/N	Risk‡, % (95% CI)
**Rapidly eliminated**				
Dondorp-2009	Cambodia	As	7/20	35.00 [15.33, 57.46]
Dondorp-2009	Thailand	As	4/15	26.67 [6.81, 52.33]
Subtotal (RE analysis; I^2=0.0%, p=0.6264)				31.36 [16.51, 48.20]
**Intermediately eliminated**				
Smithuis-2010	Myanmar	AL+/-PQ	85/153	55.56 [47.61, 63.37]
van Vugt-1998	Thailand	AL	83/248	33.47 [27.72, 39.47]
Subtotal (RE analysis; I^2=94.7%, p<0.0001)				44.22 [23.62, 65.90]
**Slowly eliminated**				
de Souza-1983	Brazil	MQ	11/49	22.45 [11.71, 35.31]
de Souza-1983	Brazil	SP	15/47	31.91 [19.25, 46.04]
Amaratunga-2016	Cambodia	DP	9/221	4.07 [1.80, 7.14]
Janssens-2007	Cambodia	DP	39/207	18.84 [13.78, 24.47]
Dondorp-2009	Cambodia	AM	6/19	31.58 [12.27, 54.50]
Janssens-2007	Cambodia	AM	47/209	22.49 [17.07, 28.41]
Valecha-2013	India	AM	7/66	10.61 [4.14, 19.35]
Mayxay-2010	Laos	DP	4/197	2.03 [0.43, 4.57]
Mayxay-2010	Laos	AM	2/98	2.04 [0.03, 6.05]
Smithuis-2010	Myanmar	As+AQ+/-PQ	59/150	39.33 [31.64, 47.29]
Smithuis-2010	Myanmar	DP+/-PQ	56/155	36.13 [28.73, 43.87]
Smithuis-2010	Myanmar	AM+/-PQ(fixed)	55/162	33.95 [26.84, 41.44]
Smithuis-2010	Myanmar	AM+/-PQ(loose)	75/149	50.34 [42.30, 58.37]
Grande-2007	Peru	DP	11/239	4.60 [2.26, 7.67]
Grande-2007	Peru	AM	10/245	4.08 [1.91, 6.97]
Ashley-2004	Thailand	DP(12/48 mg/kg)	43/149	28.86 [21.84, 36.42]
Ashley-2004	Thailand	DP(6/48 mg/kg)	52/154	33.77 [26.49, 41.45]
Ashley-2005	Thailand	DP(4 doses)	43/156	27.56 [20.81, 34.87]
Ashley-2005	Thailand	DP(3 doses)	46/161	28.57 [21.83, 35.82]
Harinasuta-1983	Thailand	MQ(500mg)	11/40	27.50 [14.61, 42.51]
Harinasuta-1983	Thailand	MQ(1000mg)	16/38	42.11 [26.75, 58.24]
Harinasuta-1983	Thailand	MQ(750mg)	16/40	40.00 [25.24, 55.70]
Ashley-2004	Thailand	AM	42/151	27.81 [20.93, 35.26]
Ashley-2005	Thailand	AM	38/157	24.20 [17.80, 31.24]
Dondorp-2009	Thailand	AM	2/18	11.11 [0.23, 30.65]
van Vugt-1998	Thailand	AM	57/230	24.78 [19.40, 30.58]
Subtotal (RE analysis; I^2=94.3%, p<0.0001)				22.11 [16.41, 28.38]
**Total for all studies (RE analysis; I^2=94.4%, p<0.0001)**				23.96 [18.33, 30.05]

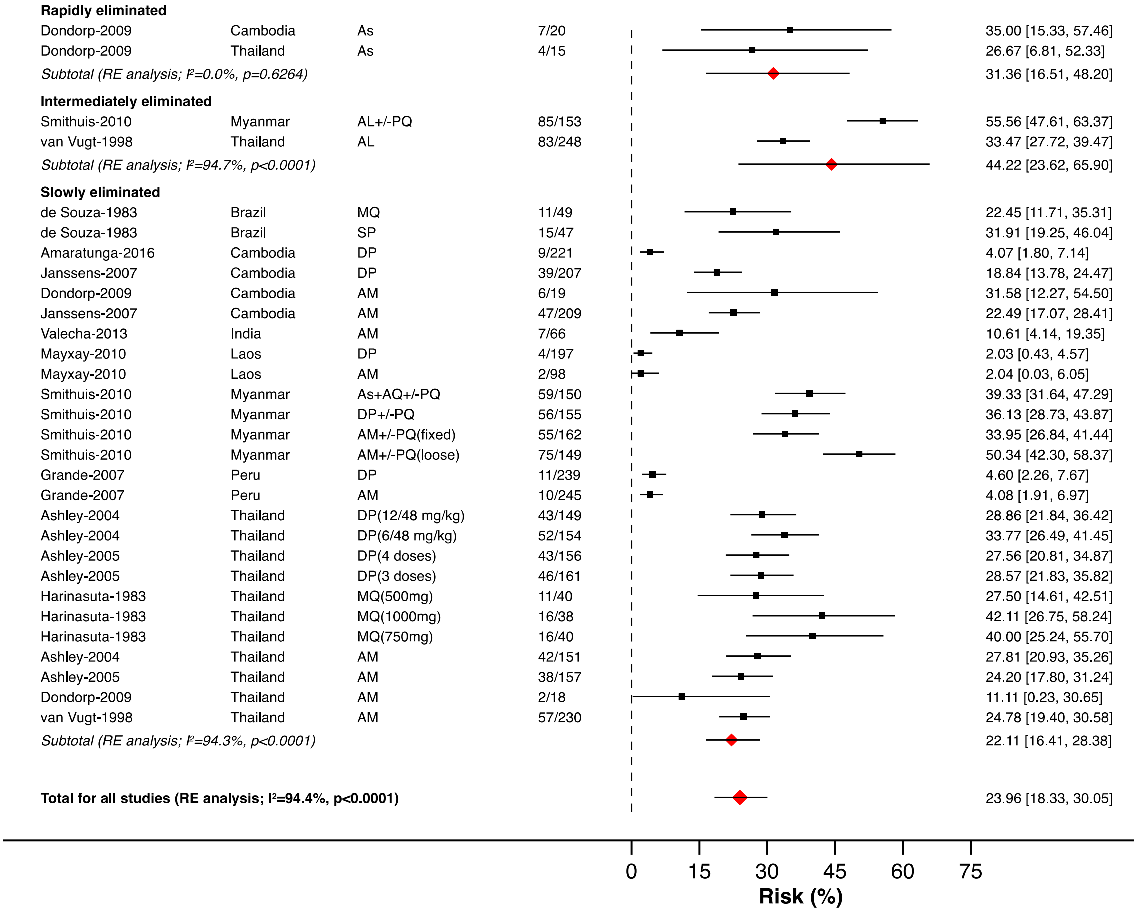

Figure S13. Risk of recurrent *P. vivax* parasitaemia by day 63 following *P. falciparum* infection by drug elimination half-life

n/N – *P. vivax* parasitaemia/total evaluable patients at day 63; CI – confidence interval; As – artesunate; RE – random effects; AL – artemether-lumefantrine; PQ - primaquine; MQ – mefloquine; SP – sulfadoxine-pyrimethamine; DP – dihydroartemisinin-piperaquine; AM – artesunate+mefloquine; AQ – amodiaquine; * Study and record details are provided in Appendix, pages 14-36; † Treatment arm described by drug with number of days given (total dose) where needed to distinguish from other treatment arms; ‡ Risk – percentage of patients with *P. vivax* parasitaemia; Refer to Table S2 for drugs included in rapid, intermediate and slow half-life elimination categories.

Appendix 14. Meta-regression analyses for all treatments

Table S11. Meta-regression of the risk of *P. vivax* parasitaemia at day 28 following *P. falciparum* infection

Variable	Records	I^2 (%)	τ^{2*}	Variance explained (R^2 (%))†	Univariable		Multivariable‡	
					Coefficient (95% CI)	p value	Coefficient (95% CI)	p value
Overall	213	86.09	0.0223					
Age (mean, per every 5 years)	170	84.41	0.0245	0.96	0.02 (-0.01, 0.04)	0.1316	0.00 (-0.03, 0.03)	0.9093
Female (%, per every 10%)	174	83.38	0.0213	4.32	-0.02 (-0.03, 0)	0.0173	-0.01 (-0.03, 0.01)	0.3882
Mixed infection (%, per every 10%)	210	83.84	0.0216	0.00	0.01 (-0.03, 0.05)	0.5486	0.00 (-0.14, 0.14)	0.9660
Baseline parasitaemia (per every 10-fold increase in mean)	186	85.34	0.0241	0.00	0.02 (-0.05, 0.09)	0.5709	0.07 (-0.02, 0.16)	0.1535
Baseline gametocytemia (%, per every 10%)	47	87.07	0.0211	0.00	-0.01 (-0.05, 0.02)	0.4612	-	-
Baseline haemoglobin (mean, g/dL)	101	88.18	0.0315	0.00	-0.01 (-0.04, 0.03)	0.7294	-0.04 (-0.08, -0.01)	0.0217
Short relapse periodicity§	213	85.15	0.0213	4.27	0.08 (0, 0.14)	0.0069	0.01 (-0.07, 0.09)	0.8171
Region		85.88	0.0222	0.46		0.2529		-
Asia-Pacific	191				0.24 (0.22, 0.26)	-	-	-
The Americas	15				0.01 (-0.08, 0.09)	-	-	-
Africa	7				-0.01 (-0.22, 0.02)	-	-	-
Drug elimination half life		68.99	0.0084	61.40		<0.0001		<0.0001
Rapid	66				0.41 (0.38, 0.45)	-	Referent	-
Intermediate	41				-0.21 (-0.26, -0.16)	-	-0.25 (-0.33, -0.17)	-
Slow	102				-0.27 (-0.31, -0.23)	-	-0.34 (-0.41, -0.27)	-
Year data collected (per every 5-year increase)	213	85.11	0.0214	3.91	-0.02 (-0.03, 0.00)	0.0121	0.00 (-0.02, 0.03)	0.7514

Results from the univariable meta-regression expressed as $\theta_i = \beta_0 + b_i + \beta_1 x_i + \varepsilon_i$, where θ_i is the Freeman-Tukey double arcsine transformed treatment completion rate from record i, β_0 is the intercept, b_i is the random effect for record i, x_i is the value of the covariate from study i, and ε_i is the within study error; * The between- study variance (τ^2) from a meta-regression model with no covariates can be compared to τ^2 from univariable meta-regression models to estimate how much variation each covariate explains. For categorical covariates τ^2 is provided for the overall covariate; † For categorical covariates, variance explained is provided for the overall covariate; ‡ Meta-regression includes 83 records, I^2=60.30%, τ^2=0.0074, R^2=75.69%; Region was not included in model due to correlation with relapse periodicity; Gametocyte percentage was not included in model due to low availability of data; § Short relapse periodicity is referenced against long relapse periodicity; CI – confidence interval.

Table S12. Meta-regression of the risk of *P. vivax* parasitaemia at day 63 following *P. falciparum* infection

Variable	Records	I^2 (%)	τ^{2*}	Variance explained (R^2 (%))†	Univariable		Multivariable‡	
					Coefficient (95% CI)	p value	Coefficient (95% CI)	p value
Overall	30	94.35	0.0320					
Age (mean, per every 5 years)	18	92.63	0.0231	1.95	0.05 (-0.04, 0.14)	0.2870	0.11 (-0.07, 0.27)	0.1901
Female (%, per every 10%)	29	94.57	0.0322	0.00	-0.02 (-0.07, 0.03)	0.3932	-	-
Mixed infection (%, per every 10%)	28	91.68	0.0224	35.10	0.17 (0.08, 0.26)	0.0003	0.21 (0.08, 0.34)	0.0016
Baseline parasitaemia (per every 10-fold increase in mean)	30	94.45	0.0325	0.00	0.07 (-0.09, 0.23)	0.4046	0.27 (-0.37, 0.92)	0.4073
Baseline gametocytemia (%, per every 10%)	26	94.16	0.0311	15.86	0.08 (0.01, 0.14)	0.0234	-0.01 (-0.22, 0.21)	0.9562
Baseline haemoglobin (mean, g/dL)	27	94.76	0.0325	2.31	-0.09 (-0.23, 0.05)	0.2208	0.06 (-0.20, 0.33)	0.6484
Short relapse periodicity§	30	93.55	0.0283	11.68	0.18 (0.01, 0.35)	0.0381	0.32 (-0.61, 1.25)	0.5052
Region		93.80	0.0296	7.57		0.0874		-
Asia-Pacific	26				0.54 (0.47, 0.61)	-	-	-
The Americas	4				-0.17 (-0.36, 0.02)	-	-	-
Africa	0				-	-	-	-
Drug elimination half life		94.03	0.0299	6.52		0.1659		0.6039
Rapid	2				0.60 (0.31, 0.89)	-	Referent	-
Intermediate	2				0.13 (-0.25, 0.51)	-	0.06 (-0.34, 0.45)	-
Slow	26				-0.11 (-0.41, 0.19)	-	-0.03 (-0.34, 0.27)	-
Year data collected (per every 5-year increase)	30	94.30	0.0316	1.49	-0.02 (-0.06, 0.01)	0.2352	-0.17 (-0.28, -0.05)	0.0049

Results from the univariable meta-regression expressed as $\theta_i = \beta_0 + b_i + \beta_1 x_i + \varepsilon_i$, where θ_i is the Freeman-Tukey double arcsine transformed treatment completion rate from record i, β_0 is the intercept, b_i is the random effect for record i, x_i is the value of the covariate from study i, and ε_i is the within study error; * The between- study variance (τ^2) from a meta-regression model with no covariates can be compared to τ^2 from univariable meta-regression models to estimate how much variation each covariate explains. For categorical covariates τ^2 is provided for the overall covariate; † For categorical covariates, variance explained is provided for the overall covariate; ‡ Meta-regression includes 16 records, I^2=55.88%, τ^2=0.0028, R^2=89.78%; Female percentage was not included in the model due to strong correlation with age (Pearson correlation coefficient -0.87 (p<0.0001); Region was not included in model due to correlation with relapse periodicity; § Short relapse periodicity is referenced against long relapse periodicity; CI – confidence interval.

Appendix 15. Forest plots of pooled estimates for artemisinin-based combination therapy

Author-Year *	Country	Treatment†	n/N	Risk‡, % (95% CI)
**Artemether-Lumefantrine**				
Itoh-2018	Brazil	AL	1/75	1.33 [0.00, 5.64]
Denis-2006b	Cambodia	AL(plus food)	1/77	1.30 [0.00, 5.49]
Denis-2006b	Cambodia	AL	2/49	4.08 [0.07, 11.91]
Jiao-1997	China	AL	0/102	0.00 [0.00, 1.68]
Assefa-2010	Ethiopia	AL	5/85	5.88 [1.69, 12.07]
Ebstie-2015	Ethiopia	AL	1/131	0.76 [0.00, 3.25]
Eshetu-2012	Ethiopia	AL	0/317	0.00 [0.00, 0.54]
Hwang-2011	Ethiopia	AL	3/115	2.61 [0.33, 6.51]
Mekonnen-2015	Ethiopia	AL	1/89	1.12 [0.00, 4.76]
Teklemariam-2017	Ethiopia	AL	2/81	2.47 [0.04, 7.30]
Wudneh-2016	Ethiopia	AL	1/82	1.22 [0.00, 5.16]
Valecha-2012	India	AL§	0/19	0.00 [0.00, 8.86]
Valecha-2012	India	AL¶	0/2	0.00 [0.00, 69.73]
Valecha-2012	India	AL‖	0/25	0.00 [0.00, 6.77]
Mayxay-2012a	Laos	AL	18/541	3.33 [1.96, 5.02]
Thapa-2007	Nepal	AL	0/66	0.00 [0.00, 2.59]
Karbwang-2000	Thailand	AL2(240/1440mg)	10/79	12.66 [6.11, 21.01]
Karbwang-2000	Thailand	AL3(320/1920mg)	14/81	17.28 [9.74, 26.37]
Karbwang-2000	Thailand	AL3(160/960mg)	6/76	7.89 [2.71, 15.20]
Krudsood-2003	Thailand	AL	0/34	0.00 [0.00, 5.00]
Lefevre-2001	Thailand	AL	6/155	3.87 [1.30, 7.58]
Valecha-2012	Thailand	AL	0/32	0.00 [0.00, 5.31]
van Vugt-1999	Thailand	AL	54/306	17.65 [13.57, 22.13]
Subtotal (RE analysis; I^2=83.6%, p<0.0001)				1.86 [0.43, 3.92]
**Dihydroartemisinin-piperaquine**				
Awab-2016	Afghanistan	DP	0/53	0.00 [0.00, 3.22]
Denis-2002	Cambodia	DP**	0/58	0.00 [0.00, 2.94]
Denis-2002	Cambodia	DP††	1/39	2.56 [0.00, 10.68]
Karunajeewa-2008b	PNG	DP	0/18	0.00 [0.00, 9.34]
Ashley-2004	Thailand	DP(6/48 mg/kg)	0/59	0.00 [0.00, 2.89]
Subtotal (RE analysis; I^2=0.0%, p=0.7190)				0.04 [0.00, 1.33]
**Artesunate-mefloquine**				
Denis-2006a	Cambodia	AM	1/977	0.10 [0.00, 0.44]
Denis-2006b	Cambodia	AM	0/52	0.00 [0.00, 3.28]
Ashley-2004	Thailand	AM	0/59	0.00 [0.00, 2.89]
Bunnag-1997	Thailand	AM	0/47	0.00 [0.00, 3.63]
Krudsood-2002	Thailand	AM(simultaneous)	0/101	0.00 [0.00, 1.70]
Krudsood-2002	Thailand	AM(sequential)	0/102	0.00 [0.00, 1.68]
Lefevre-2001	Thailand	AM	0/53	0.00 [0.00, 3.22]
Tangpukdee-2008	Thailand	AM	0/65	0.00 [0.00, 2.63]
Thimarsarn-1997	Thailand	AM(600/750mg)	1/57	1.75 [0.00, 7.38]
Thimarsarn-1997	Thailand	AM(600/1250mg)	1/78	1.28 [0.00, 5.42]
Wilairatana-1998	Thailand	AM	9/130	6.92 [3.10, 12.01]
Wilairatana-2002	Thailand	AM	0/82	0.00 [0.00, 2.09]
Subtotal (RE analysis; I^2=60.1%, p=0.0023)				0.30 [0.00, 1.25]

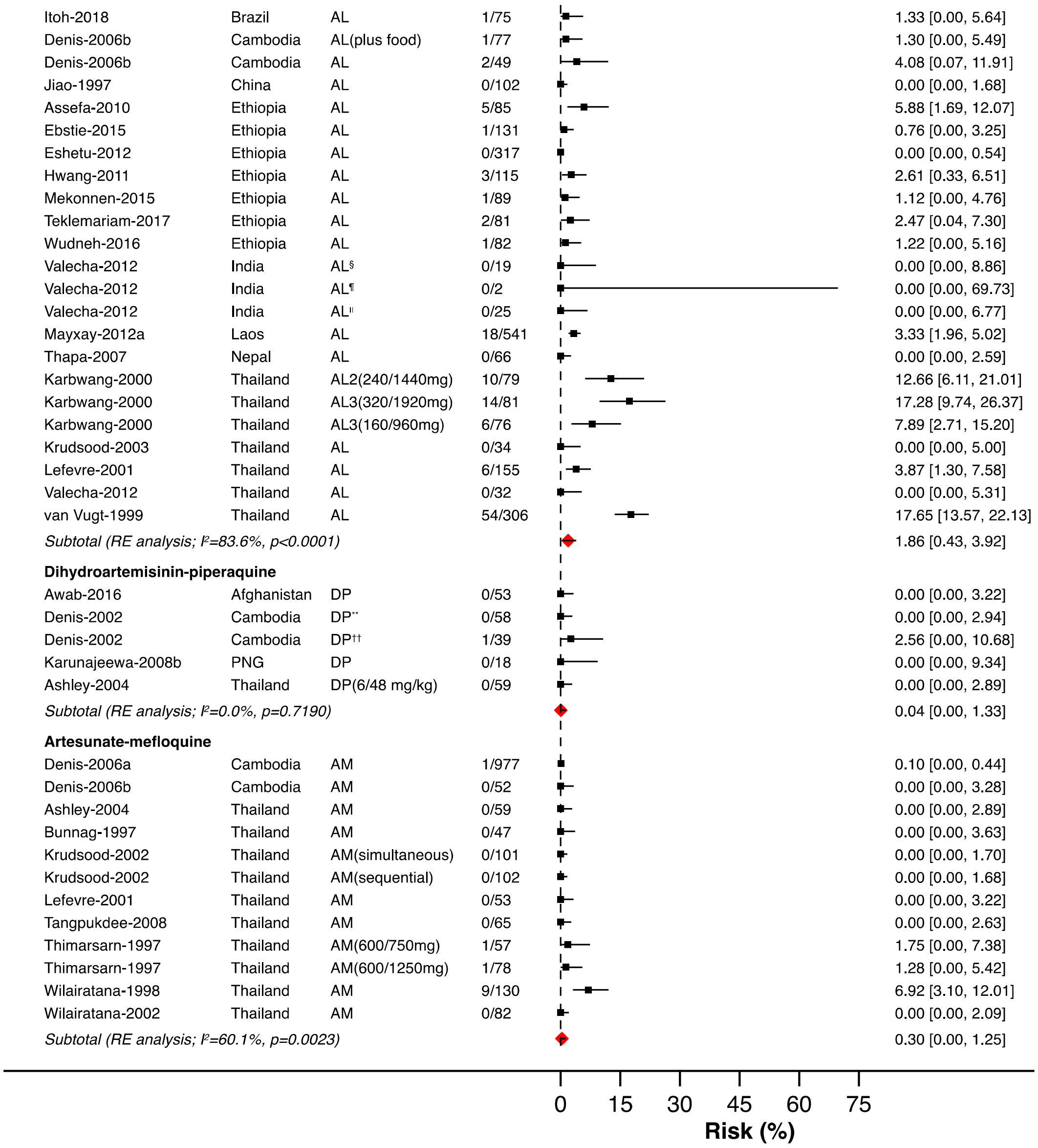

Figure S14. Risk of *P. vivax* parasitaemia by day 28 following *P. falciparum* infection by artemisinin-based combination therapy

n/N – *P. vivax* parasitaemia/total evaluable patients at day 28; CI – confidence interval; AL – artemether-lumefantrine; RE – random effects; DP– dihydroartemisinin-piperaquine; PNG – Papua New Guinea; AM – artesunate+mefloquine; * Study and record details are provided in Appendix, pages 14-36; † Treatment arm described by drug with number of days given (total dose) where needed to distinguish from other treatment arms; ‡ Risk – percentage of patients with *P. vivax* parasitaemia; § Jamshedpur, India; ¶ Ranchi, India; || Rourkela, India; ** Oddor Meanchey, Cambodia; †† Snoul, Cambodia.

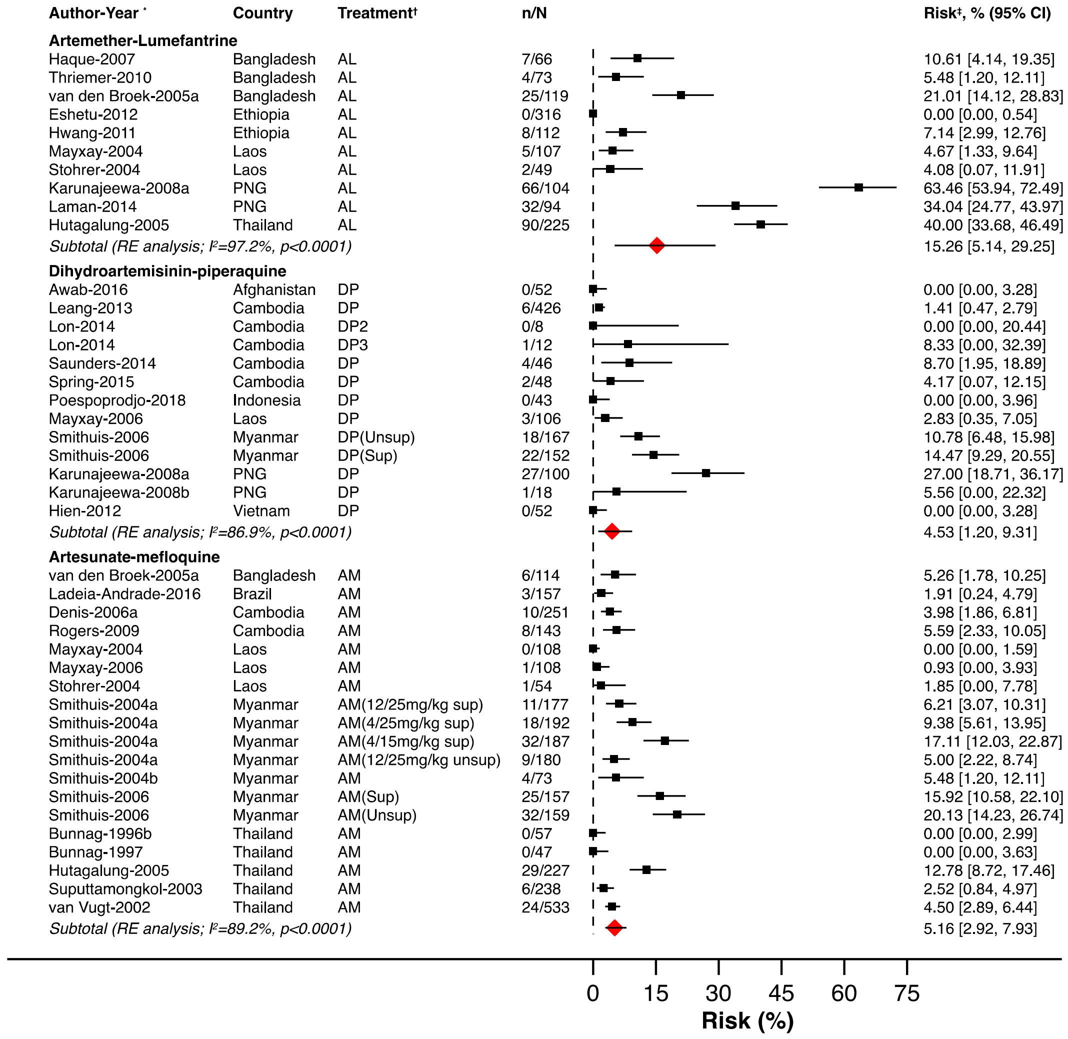

Figure S15. Risk of recurrent *P. vivax* parasitaemia by day 42 following *P. falciparum* infection by artemisinin-based combination therapy

n/N – *P. vivax* parasitaemia/total evaluable patients at day 42; CI – confidence interval; AL – artemether-lumefantrine; RE – random effects; DP– dihydroartemisinin-piperaquine; Unsup – unsupervised; Sup – supervised; PNG – Papua New Guinea; AM – artesunate+mefloquine; * Study and record details are provided in Appendix, pages 14-36; † Treatment arm described by drug with number of days given (total dose) where needed to distinguish from other treatment arms; ‡ Risk – percentage of patients with *P. vivax* parasitaemia.

Author-Year *	Country	Treatment†	n/N	Risk‡, % (95% CI)
**Artemether-Lumefantrine**				
van Vugt-1998	Thailand	AL	83/248	33.47 [27.72, 39.47]
**Dihydroartemisinin-piperaquine**				
Amaratunga-2016	Cambodia	DP	9/221	4.07 [1.80, 7.14]
Janssens-2007	Cambodia	DP	39/207	18.84 [13.78, 24.47]
Mayxay-2010	Laos	DP	4/197	2.03 [0.43, 4.57]
Grande-2007	Peru	DP	11/239	4.60 [2.26, 7.67]
Ashley-2004	Thailand	DP(12/48 mg/kg)	43/149	28.86 [21.84, 36.42]
Ashley-2004	Thailand	DP(6/48 mg/kg)	52/154	33.77 [26.49, 41.45]
Ashley-2005	Thailand	DP(4 doses)	43/156	27.56 [20.81, 34.87]
Ashley-2005	Thailand	DP(3 doses)	46/161	28.57 [21.83, 35.82]
Subtotal (RE analysis; I²=96.4%, p<0.0001)				16.19 [7.57, 27.21]
**Artesunate-mefloquine**				
Dondorp-2009	Cambodia	AM	6/19	31.58 [12.27, 54.50]
Janssens-2007	Cambodia	AM	47/209	22.49 [17.07, 28.41]
Valecha-2013	India	AM	7/66	10.61 [4.14, 19.35]
Mayxay-2010	Laos	AM	2/98	2.04 [0.03, 6.05]
Grande-2007	Peru	AM	10/245	4.08 [1.91, 6.97]
Ashley-2004	Thailand	AM	42/151	27.81 [20.93, 35.26]
Ashley-2005	Thailand	AM	38/157	24.20 [17.80, 31.24]
Dondorp-2009	Thailand	AM	2/18	11.11 [0.23, 30.65]
van Vugt-1998	Thailand	AM	57/230	24.78 [19.40, 30.58]
Subtotal (RE analysis; I²=92.0%, p<0.0001)				15.74 [8.62, 24.40]

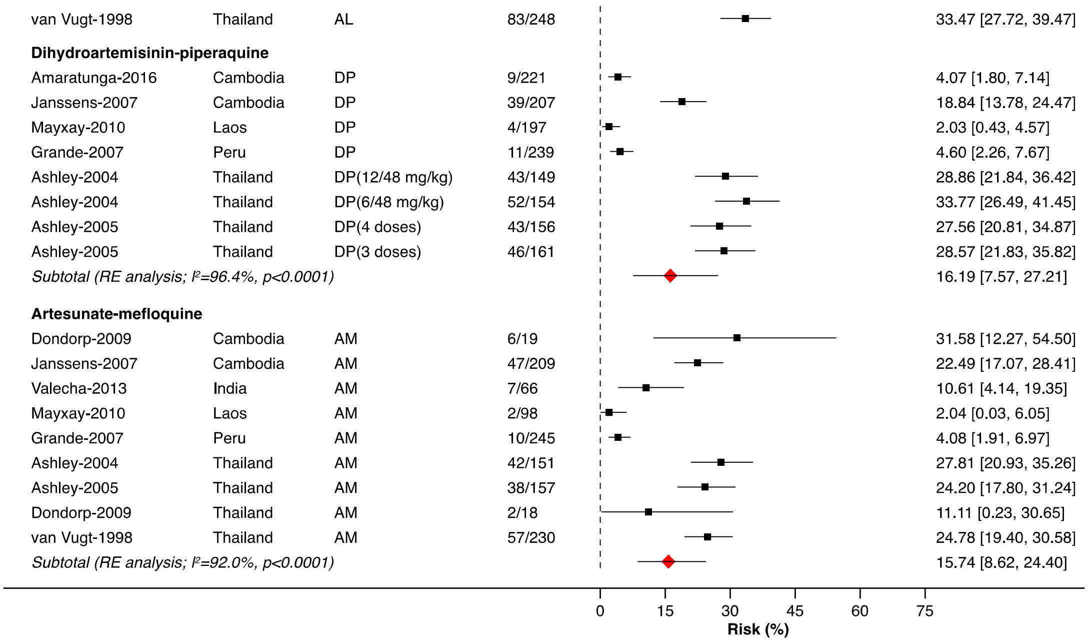

Figure S16. Risk of *P. vivax* parasitaemia by day 63 following *P. falciparum* infection by artemisinin-based combination therapy

n/N – *P. vivax* parasitaemia/total evaluable patients at day 63; CI – confidence interval; AL – artemether-lumefantrine; RE – random effects; DP– dihydroartemisinin-piperaquine; AM – artesunate+mefloquine; * Study and record details are provided in Appendix, pages 14-36; † Treatment arm described by drug with number of days given (total dose) where needed to distinguish from other treatment arms; ‡ Risk – percentage of patients with *P. vivax* parasitaemia.

Appendix 16. Meta-regression analyses for artemisinin-combination therapy

Table S13. Meta-regression of the risk of *P. vivax* parasitaemia at day 42 following *P. falciparum* infection after treatment with an ACT

Variable	Records	I^2 (%)	τ^{2*}	Variance explained (R^2 (%))†	Univariable Coefficient (95% CI)	Univariable p value	Multivariable‡ Coefficient (95% CI)	Multivariable‡ p value
Overall	42	94.50	0.0322					
Age (mean, per every 5 years)	28	94.71	0.0352	20.73	-0.07 (-0.12, -0.02)	0.0057	0.00 (-0.05, 0.05)	0.9172
Female (%, per every 10%)	36	94.72	0.0323	11.62	0.05 (0.01, 0.09)	0.0208	-	-
Mixed infection (%, per every 10%)	38	89.97	0.0163	12.17	0.08 (0.01, 0.16)	0.0320	0.08 (-0.31, 0.47)	0.6889
Baseline parasitaemia (per every 10-fold increase in mean)	38	94.54	0.0334	5.33	0.16 (-0.03, 0.34)	0.0930	-0.08 (-0.28, 0.13)	0.4686
Baseline gametocytemia (%, per every 10%)	19	93.85	0.0265	1.11	0.04 (-0.03, 0.10)	0.2453	-	-
Baseline haemoglobin (mean, g/dL)	28	93.51	0.0266	31.71	-0.07 (-0.11, -0.03)	0.0004	-	-
Short relapse periodicity§	42	94.13	0.0306	5.22	-0.16 (-0.35, 0.02)	0.0855	-0.15 (-0.30, 0.00)	0.0569
Region		94.39	0.0325	0.00		0.4528		-
Asia-Pacific	39				0.29 (0.23, 0.35)	-	-	-
The Americas	1				-0.14 (-0.51, 0.23)	-	-	-
Africa	2				-0.14 (-0.40, 0.12)	-	-	-
Artemisinin combination therapy		93.61	0.0278	13.82		0.0076		0.0049
Dihydroartemisinin-piperaquine or artesunate-mefloquine	32				0.24 (0.18, 0.30)	-	Referent	-
Artemether-lumefantrine	10				0.17 (0.05, 0.29)	-	0.18 (0.06, 0.31)	-
Year data collected (per every 5-year increase)	42	94.55	0.0331	18.35	0.00 (-0.06, 0.06)	0.9948	-	-

Results from the univariable meta-regression expressed as $\theta_i = \beta_0 + b_i + \beta_1 x_i + \varepsilon_i$, where θ_i is the Freeman-Tukey double arcsine transformed treatment completion rate from record i, β_0 is the intercept, b_i is the random effect for record i, x_i is the value of the covariate from study i, and ε_i is the within study error; * The between- study variance (τ^2) from a meta-regression model with no covariates can be compared to τ^2 from univariable meta-regression models to estimate how much variation each covariate explains. For categorical covariates this is provided for the overall covariate; † For categorical covariates, variance explained is provided for the overall covariate; ‡ Meta-regression includes 25 records, I^2=88.74%, τ^2=0.0163, R^2=21.01%; Region was not included in model due to correlation with relapse periodicity; Baseline Hb and female percentage were no included in model due to correlation with age; Gametocyte percentage was not included in model due to low availability of data; Year of study was not included as it was considered a less significant confounders and given the small number of total studies; § Short relapse periodicity is referenced against long relapse periodicity; CI – confidence interval.

Author-Year *	Country	Treatment†	n/N	Risk‡, % (95% CI)
**Artemether-Lumefantrine**				
Thriemer-2010	Bangladesh	AL	4/73	5.48 [1.20, 12.11]
van den Broek-2005a	Bangladesh	AL	25/119	21.01 [14.12, 28.83]
Eshetu-2012	Ethiopia	AL	0/316	0.00 [0.00, 0.54]
Hwang-2011	Ethiopia	AL	8/112	7.14 [2.99, 12.76]
Mayxay-2004	Laos	AL	5/107	4.67 [1.33, 9.64]
Stohrer-2004	Laos	AL	2/49	4.08 [0.07, 11.91]
Hutagalung-2005	Thailand	AL	90/225	40.00 [33.68, 46.49]
Subtotal (RE analysis; I^2=96.0%, p<0.0001)				8.98 [1.82, 20.34]
**Dihydroartemisinin-piperaquine or Artesunate-mefloquine**				
Awab-2016	Afghanistan	DP	0/52	0.00 [0.00, 3.28]
Leang-2013	Cambodia	DP	6/426	1.41 [0.47, 2.79]
Spring-2015	Cambodia	DP	2/48	4.17 [0.07, 12.15]
Poespoprodjo-2018	Indonesia	DP	0/43	0.00 [0.00, 3.96]
Mayxay-2006	Laos	DP	3/106	2.83 [0.35, 7.05]
Karunajeewa-2008b	PNG	DP	1/18	5.56 [0.00, 22.32]
Hien-2012	Vietnam	DP	0/52	0.00 [0.00, 3.28]
van den Broek-2005a	Bangladesh	AM	6/114	5.26 [1.78, 10.25]
Ladeia-Andrade-2016	Brazil	AM	3/157	1.91 [0.24, 4.79]
Rogers-2009	Cambodia	AM	8/143	5.59 [2.33, 10.05]
Mayxay-2004	Laos	AM	0/108	0.00 [0.00, 1.59]
Mayxay-2006	Laos	AM	1/108	0.93 [0.00, 3.93]
Stohrer-2004	Laos	AM	1/54	1.85 [0.00, 7.78]
Smithuis-2004b	Myanmar	AM	4/73	5.48 [1.20, 12.11]
Bunnag-1996b	Thailand	AM	0/57	0.00 [0.00, 2.99]
Bunnag-1997	Thailand	AM	0/47	0.00 [0.00, 3.63]
Hutagalung-2005	Thailand	AM	29/227	12.78 [8.72, 17.46]
van Vugt-2002	Thailand	AM	24/533	4.50 [2.89, 6.44]
Subtotal (RE analysis; I^2=74.7%, p<0.0001)				2.15 [0.91, 3.78]

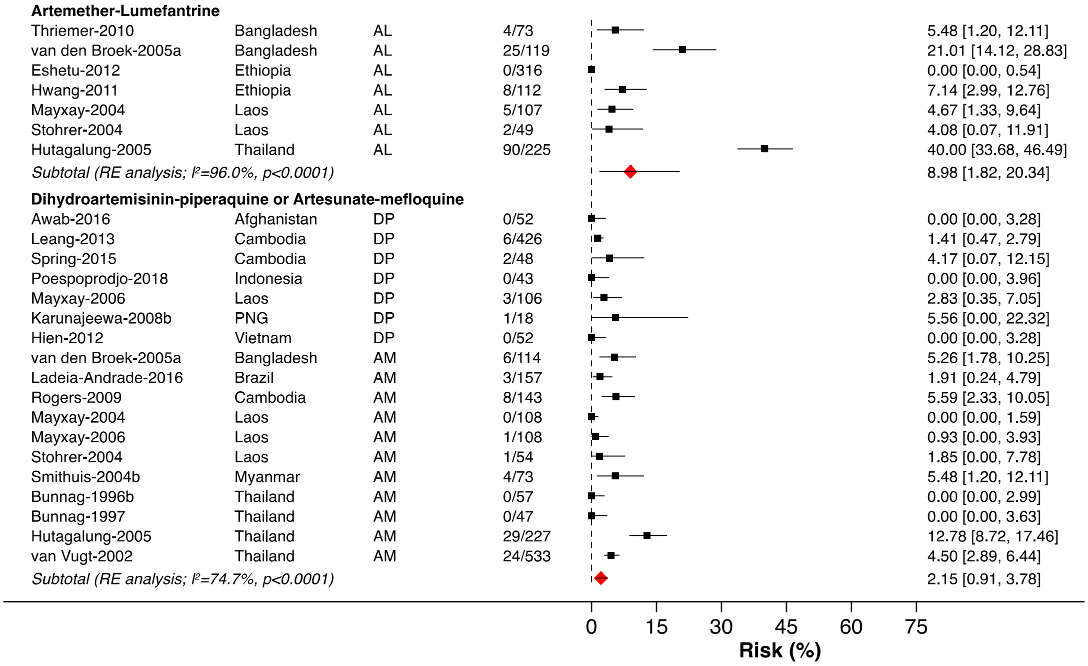

Figure S17. Day 42 risk of *P. vivax* parasitaemia following *P. falciparum* infection by artemisinin-based combination therapies for studies included in multivariable meta-regression

Meta-regression results presented in Table S13; n/N – *P. vivax* parasitaemia/total evaluable patients at day 42; CI – confidence interval; AL – artemether-lumefantrine; RE – random effects; DP–dihydroartemisinin-piperaquine; PNG – Papua New Guinea; AM – artesunate+mefloquine; * Study and record details are provided in Appendix, pages 14-36; † Treatment arm described by drug with number of days given (total dose) where needed to distinguish from other treatment arms; ‡ Risk – percentage of patients with *P. vivax* parasitaemia.

Appendix 17. Excluded references

184. Storey J, Rossi-Espagnet A, Mandel SP, et al. Sulfalene with pyrimethamine and chloroquine with pyrimethamine in single-dose treatment of *Plasmodium falciparum* infections. A trial in a rural population in northern Nigeria. *Bull World Health Organ* 1973; **49**(3): 275-82.
185. Fasan PO, Kofie BA, Adeleye I, Cooke A. Orally administered amodiaquine base suspensions in the treatment of acute malaria infections of young children in Nigeria. *J Trop Med Hyg* 1974; **77**(10): 239-43.
186. Fasan PO, Mabadeje AF. A controlled trial of a combination of chloroquine with paracetamol in the treatment of acute malaria in a semi-immune population. *J Trop Med Hyg* 1980; **83**(5): 191-3.
187. Aderounmu AF, Salako LA, Walker O. Chloroquine sensitivity of *Plasmodium falciparum* in Ibadan, Nigeria: II. Correlation of *in vitro* with *in vivo* sensitivity. *Trans R Soc Trop Med Hyg* 1981; **75**(5): 637-9.
188. Olatunde A, Salako LA, Walker O. The in vivo sensitivity of *Plasmodium falciparum* to chloroquine and to sulphadoxine-pyrimethamine combination in Ibadan, Nigeria. *Trans R Soc Trop Med Hyg* 1981; **75**(6): 848-50.
189. Kofi Ekue JM, Ulrich AM, Rwabwogo-Atenyi J, Sheth UK. A double-blind comparative clinical trial of mefloquine and chloroquine in symptomatic falciparum malaria. *Bull World Health Organ* 1983; **61**(4): 713-8.
190. Schwartz IK, Payne D, Campbell CC, Khatib OJ. *In-vivo* and *in-vitro* assessment of chloroquine-resistant *Plasmodium falciparum* malaria in Zanzibar. *Lancet* 1983; **1**(8332): 1003-5.
191. Magloire R, Nguyen-Dinh P. Chloroquine susceptibility of *Plasmodium falciparum* in Haiti. *Bull World Health Organ* 1983; **61**(6): 1017-20.
192. Spencer HC, Watkins WM, Sixsmith DG, Koech DK, Chulay JD. A new *in vitro* test for pyrimethamine/sulfadoxine susceptibility of *Plasmodium falciparum* and its correlation with *in vivo* resistance in Kenya. *Bull World Health Organ* 1984; **62**(4): 615-21.
193. Wood R. Chloroquine resistant *Plasmodium falciparum* malaria confirmed by *in vivo* testing in Lusaka. *Med J Zambia* 1984; **18**(4): 5-6.
194. Walker O, Salako LA, Obih PO, Bademosi K, Sodeinde O. The sensitivity of *Plasmodium falciparum* to chloroquine and amodiaquine in Ibadan, Nigeria. *Trans R Soc Trop Med Hyg* 1984; **78**(6): 782-4.
195. Nguyen-Dinh P, Zevallos-Ipenza A, Magloire R. *Plasmodium falciparum* in Haiti: susceptibility to pyrimethamine and sulfadoxine-pyrimethamine. *Bull World Health Organ* 1984; **62**(4): 623-6.
196. Ngimbi NP, Wery M, Henry MC, Mulumba MP. [*In vivo* response to chloroquine during the treatment of *Plasmodium falciparum* malaria in a suburban region of Kinshasa, Zaire]. *Ann Soc Belg Med Trop* 1985; **65 Suppl 2**: 123-35.
197. Nguyen-Dinh P, Schwartz IK, Sexton JD, et al. *In vivo* and *in vitro* susceptibility to chloroquine of *Plasmodium falciparum* in Kinshasa and Mbuji-Mayi, Zaire. *Bull World Health Organ* 1985; **63**(2): 325-30.
198. Ekue JM, Simooya OO, Sheth UK, Wernsdorfer WH, Njelesani EK. A double-blind clinical trial of a combination of mefloquine, sulfadoxine and pyrimethamine in symptomatic falciparum malaria. *Bull World Health Organ* 1985; **63**(2): 339-43.
199. Bjorkman A, Rombo L, Willcox M, Hanson AP, Bengtsson E. *In vivo* response of *Plasmodium falciparum* to different doses of chloroquine in semi-immune children in Liberia, West Africa. *Ann Trop Med Parasitol* 1986; **80**(1): 1-6.
200. Bjorkman A, Willcox M. *In vivo* and *in vitro* susceptibility of *Plasmodium falciparum* to sulphadoxine/pyrimethamine in Liberia, West Africa. *Trans R Soc Trop Med Hyg* 1986; **80**(4): 572-4.
201. Ekue JM, Phiri DE, Sheth UK, Mukunyandela M. A double-blind trial of a fixed combination of mefloquine plus sulfadoxine-pyrimethamine compared with sulfadoxine-pyrimethamine alone in symptomatic falciparum malaria. *Bull World Health Organ* 1987; **65**(3): 369-73.
202. Steketee RW, Brandling-Bennett AD, Kaseje DC, Schwartz IK, Churchill FC. *In vivo* response of *Plasmodium falciparum* to chloroquine in pregnant and non-pregnant women in Siaya District, Kenya. *Bull World Health Organ* 1987; **65**(6): 885-90.
203. Salako LA, Aderounmu AF, Laoye JO, Makinde JM, Adio RA. Sensitivity of *Plasmodium falciparum* to mefloquine-sulphadoxine-pyrimethamine (Fansimef*) in vivo* and to mefloquine alone *in vitro* in Nigeria. *Ann Trop Med Parasitol* 1988; **82**(4): 325-30.
204. Schapira A, Schwalbach JF. Evaluation of four therapeutic regimens for falciparum malaria in Mozambique, 1986. *Bull World Health Organ* 1988; **66**(2): 219-26.
205. Schapira A, Almeida Franco LT, Averkiev L, Omawale, Schwalbach JF, Suleimanov G. The *Plasmodium falciparum* chloroquine *in vivo* test: extended follow-up is more important than parasite counting. *Trans R Soc Trop Med Hyg* 1988; **82**(1): 39-43.
206. Lemnge MM, Inambao AW. *In vivo* and *in vitro* sensitivity of *Plasmodium falciparum* to chloroquine at Lubwe and Kalene in Zambia: use of amodiaquine as an alternative antimalarial drug. *Trans R Soc Trop Med Hyg* 1988; **82**(2): 194-6.
207. Salako LA, Sowunmi A, Laoye OJ. Evaluation of the sensitivity *in vivo* and *in vitro* of *Plasmodium falciparum* malaria to quinine in an area of full sensitivity to chloroquine. *Trans R Soc Trop Med Hyg* 1988; **82**(3): 366-8.
208. Watkins WM, Brandling-Bennett AD, Nevill CG, et al. Chlorproguanil/dapsone for the treatment of non-severe *Plasmodium falciparum* malaria in Kenya: a pilot study. *Trans R Soc Trop Med Hyg* 1988; **82**(3): 398-403.

209. Mbudi PK, Pela NN, Kalonji MW, Disu MM. [Comparative efficacy of alternative treatments in *Plasmodium falciparum* infections in Zaire]. *Ann Soc Belg Med Trop* 1989; **69**(1): 25-33.

210. Richard-Lenoble D, Kombila M, Martz M, et al. [Development of chloroquine resistance in *Plasmodium falciparum* in Gabon between 1984 and 1987-88 (in vivo evaluation in a school environment)]. *Ann Soc Belg Med Trop* 1989; **69**(2): 113-9.

211. Daniel HI, Molta NB. Efficacy of chloroquine in the treatment of malaria in children under five years in Baissa (Gongola State, Nigeria). *Ann Trop Med Parasitol* 1989; **83**(4): 331-8.

212. Petersen E, Hogh B, Hanson AP, Bjorkman A, Flacks H. *In vitro* and *in vivo* susceptibility of *Plasmodium falciparum* isolates from Liberia to pyrimethamine, cycloguanil and chlorcycloguanil. *Ann Trop Med Parasitol* 1990; **84**(6): 563-71.

213. Keuter M, van Eijk A, Hoogstrate M, et al. Comparison of chloroquine, pyrimethamine and sulfadoxine, and chlorproguanil and dapsone as treatment for falciparum malaria in pregnant and non-pregnant women, Kakamega District, Kenya. *BMJ* 1990; **301**(6750): 466-70.

214. Slutsker LM, Khoromana CO, Payne D, et al. Mefloquine therapy for *Plasmodium falciparum* malaria in children under 5 years of age in Malawi: *in vivo/in vitro* efficacy and correlation of drug concentration with parasitological outcome. *Bull World Health Organ* 1990; **68**(1): 53-9.

215. Raccurt CP, Arouko H, Djossou F, et al. [*In vivo* amodiaquine sensitivity of *Plasmodium falciparum* in the town of Cotonou and in the vicinity (Benin)]. *Med Trop (Mars)* 1990; **50**(1): 21-6.

216. Gazin P, Louis JP, Mulder L, et al. [Evaluation of *Plasmodium falciparum* susceptibility to chloroquine and amodiaquine using a simplified, *in vivo*, 7-day test in southern Cameroon]. *Med Trop (Mars)* 1990; **50**(1): 27-31.

217. Sowunmi A, Salako LA, Walker O, Ogundahunsi OA. Clinical efficacy of mefloquine in children suffering from chloroquine-resistant *Plasmodium falciparum* malaria in Nigeria. *Trans R Soc Trop Med Hyg* 1990; **84**(6): 761-4.

218. Salako LA, Sowunmi A, Walker O. Evaluation of the clinical efficacy and safety of halofantrine in falciparum malaria in Ibadan, Nigeria. *Trans R Soc Trop Med Hyg* 1990; **84**(5): 644-7.

219. Oloo AJ, Adoyo A, Kariuki D, et al. Sensitivity of falciparum malaria to chloroquine and amodiaquine in four districts of western Kenya (1985-1987). *East Afr Med J* 1991; **68**(8): 606-10.

220. Ezedinachi EN, Ejezie GC, Usanga EA, Gemade EI, Ikpatt NW, Alaribe AA. New trends in chloroquine efficacy in the treatment of malaria: chloroquine-resistant *Plasmodium falciparum* in Anambra and Benue States of Nigeria. *Cent Afr J Med* 1991; **37**(6): 180-6.

221. Mutabingwa TK, Malle LN, Mtui SN. Chloroquine therapy still useful in the management of malaria during pregnancy in Muheza, Tanzania. *Trop Geogr Med* 1991; **43**(1-2): 131-5.

222. Irare SG, Lemnge MM, Mhina JI. Falciparum malaria fully cleared by amodiaquine, pyrimethamine-sulfadoxine and pyrimethamine-sulfalene in areas of chloroquine resistance in Dodoma, Tanzania. *Trop Geogr Med* 1991; **43**(4): 352-6.

223. Sowunmi A, Salako LA. Evaluation of the relative efficacy of various antimalarial drugs in Nigerian children under five years of age suffering from acute uncomplicated falciparum malaria. *Ann Trop Med Parasitol* 1992; **86**(1): 1-8.

224. Rukaria RM, Ojwang SB, Oyieke JB, Kigondu CB. *In vivo* and *in vitro* response of *Plasmodium falciparum* to chloroquine in pregnant women in Kilifi district, Kenya. *East Afr Med J* 1992; **69**(6): 306-10.

225. Simooya O, Mutetwa S, Chandiwana S, Neill P, Mharakurwa S, Stein M. A comparative study of the schizontocidal efficacy and safety of artemether versus chloroquine in uncomplicated malaria. *Cent Afr J Med* 1992; **38**(7): 257-63.

226. McClean KL, Hitchman D, Shafran SD. Norfloxacin is inferior to chloroquine for falciparum malaria in northwestern Zambia: a comparative clinical trial. *J Infect Dis* 1992; **165**(5): 904-7.

227. Sowunmi A, Oduola AM, Salako LA, Ogundahunsi OA, Laoye OJ, Walker O. The relationship between the response of *Plasmodium falciparum* malaria to mefloquine in African children and its sensitivity *in vitro*. *Trans R Soc Trop Med Hyg* 1992; **86**(4): 368-71.

228. Vollenhoven I, Wetsteyn JC. Chloroquine resistance of *Plasmodium falciparum* in Bukumbi, Tanzania. An *in vivo* study. *Trop Geogr Med* 1992; **44**(1-2): 182-5.

229. Mvondo JL, James MA, Campbell CC. Malaria and pregnancy in Cameroonian women. Effect of pregnancy on *Plasmodium falciparum* parasitemia and the response to chloroquine. *Trop Med Parasitol* 1992; **43**(1): 1-5.

230. Fadat G, Louis FJ, Louis JP, Le Bras J. Efficacy of micronized halofantrine in semi-immune patients with acute uncomplicated falciparum malaria in Cameroon. *Antimicrob Agents Chemother* 1993; **37**(9): 1955-7.

231. Hagos B, Khan B, Ofulla AV, Kariuki D, Martin SK. Response of falciparum malaria to chloroquine and three second line antimalarial drugs in a Kenyan coastal school age population. *East Afr Med J* 1993; **70**(10): 620-3.

232. Kremsner PG, Winkler S, Brandts C, Graninger W, Bienzle U. Curing of chloroquine-resistant malaria with clindamycin. *Am J Trop Med Hyg* 1993; **49**(5): 650-4.

233. Fowler VG, Jr., Lemnge M, Irare SG, et al. Efficacy of chloroquine on *Plasmodium falciparum* transmitted at Amani, eastern Usambara Mountains, north-east Tanzania: an area where malaria has recently become endemic. *J Trop Med Hyg* 1993; **96**(6): 337-45.

234. Penali LK, Assi-Coulibaly L, Kaptue B, Konan D, Ehouman A. [Parasitological and clinical response to amodiaquine versus chloroquine in the treatment of *Plasmodium falciparum* malaria in children in an endemic area]. *Bull Soc Pathol Exot* 1994; **87**(4): 244-7.

235. Nevill CG, Verhoeff FH, Munafu CG, ten Hove WR, van der Kaay HJ, Were JB. A comparison of amodiaquine and chloroquine in the treatment therapy of falciparum malaria in Kenya. *East Afr Med J* 1994; **71**(3): 167-70.

236. Schultz LJ, Steketee RW, Macheso A, Kazembe P, Chitsulo L, Wirima JJ. The efficacy of antimalarial regimens containing sulfadoxine-pyrimethamine and/or chloroquine in preventing peripheral and placental *Plasmodium falciparum* infection among pregnant women in Malawi. *Am J Trop Med Hyg* 1994; **51**(5): 515-22.

237. Kremsner PG, Wildling E, Jenne L, Graninger W, Bienzle U. Comparison of micronized halofantrine with chloroquine-antibiotic combinations for treating *Plasmodium falciparum* malaria in adults from Gabon. *Am J Trop Med Hyg* 1994; **50**(6): 790-5.

238. Wildling E, Jenne L, Graninger W, Bienzle U, Kremsner PG. High dose chloroquine versus micronized halofantrine in chloroquine-resistant *Plasmodium falciparum* malaria. *J Antimicrob Chemother* 1994; **33**(4): 871-5.

239. Kremsner PG, Winkler S, Brandts C, Neifer S, Bienzle U, Graninger W. Clindamycin in combination with chloroquine or quinine is an effective therapy for uncomplicated *Plasmodium falciparum* malaria in children from Gabon. *J Infect Dis* 1994; **169**(2): 467-70.

240. Kamugisha J, Kipp W, Burnham G. *In vivo* sensitivity of *Plasmodium falciparum* to chloroquine, amodiaquine and sulfadoxine-pyrimethamine in western Uganda. *Trop Geogr Med* 1994; **46**(6): 364-5.

241. Del Nero L, Nebie I, Soudouem G, Pietra V. Chloroquine and sulfadoxine/pyrimethamine sensitivity in Burkina Faso. In vivo sensitivity of *Plasmodium falciparum* to chloroquine and sulfadoxine/pyrimethamine in Burkina Faso. *Trop Geogr Med* 1994; **46**(1): 8-10.

242. Ringwald P, Louis FJ, Bickii J, Ringwald C, Daban A, Ravinet L. Efficacy and tolerance in adults of a short (3 days) course of quinine for uncomplicated falciparum malaria. *Ann Soc Belg Med Trop* 1995; **75**(2): 141-3.

243. Metzger W, Mordmuller B, Graninger W, Bienzle U, Kremsner PG. High efficacy of short-term quinine-antibiotic combinations for treating adult malaria patients in an area in which malaria is hyperendemic. *Antimicrob Agents Chemother* 1995; **39**(1): 245-6.

244. Agnamey PP, Leroy G, Kouamouo J, Brasseur P. [Sensitivity *in vivo* and *in vitro* of *Plasmodium falciparum* to chloroquine and amodiaquine in Bangangte (west Cameroon)]. *Bull Soc Pathol Exot* 1995; **88**(4): 149-51.

245. Nevill CG, Ochen K, Munafu CG, Bekobita D, Sezi CL. Response of *Plasmodium falciparum* to chloroquine and Fansidar *in vivo* and chloroquine and amodiaquine *in vitro* in Uganda. *East Afr Med J* 1995; **72**(6): 349-54.

246. Mbori-Ngacha DA, Onyango FE, Chunge C, Luta M, Oloo AJ, Muga RO. Efficacy of halofantrine in the treatment of uncomplicated falciparum malaria. *East Afr Med J* 1995; **72**(12): 796-9.

247. Barennes H, Kahiatani D, Clavier F, et al. [Rectal quinine, an alternative to parenteral injections for the treatment of childhood malaria. Clinical, parasitological and pharmacological study]. *Med Trop (Mars)* 1995; **55**(4 Suppl): 91-4.

248. Alin MH, Kihamia CM, Bjorkman A, et al. Efficacy of oral and intravenous artesunate in male Tanzanian adults with *Plasmodium falciparum* malaria and in vitro susceptibility to artemisinin, chloroquine, and mefloquine. *Am J Trop Med Hyg* 1995; **53**(6): 639-45.

249. Blom GJ, Baboo KS, Athale UH, van der Werf TS. *Plasmodium falciparum* malaria *in vivo* drug sensitivity in Lusaka, Zambia. *Cent Afr J Med* 1995; **41**(1): 6-10.

250. Metzger W, Mordmuller B, Graninger W, Bienzle U, Kremsner PG. Sulfadoxine/pyrimethamine or chloroquine/clindamycin treatment of Gabonese school children infected with chloroquine resistant malaria. *J Antimicrob Chemother* 1995; **36**(4): 723-8.

251. Adagu SI, Okoyeh JN, Lege-Oguntoye L, et al. Efficacy of a 3-day oral regimen of quinine in an area of northern Nigeria with low-grade resistance of *Plasmodium falciparum* to chloroquine and sulphadoxine-pyrimethamine. *J Trop Med Hyg* 1995; **98**(5): 296-8.

252. Sowunmi A, Oduola AM. Open comparison of mefloquine, mefloquine/sulfadoxine/pyrimethamine and chloroquine in acute uncomplicated falciparum malaria in children. *Trans R Soc Trop Med Hyg* 1995; **89**(3): 303-5.

253. Okoyeh JN, Lege-Oguntoye L, Emembolu JO, Sarki U. Sensitivity of *Plasmodium falciparum* to reduced dose of mefloquine in pregnant women in Nigeria. *Acta Trop* 1996; **61**(1): 1-8.

254. Okeyeh JN, Lege-Oguntoye L, Emembolu JO, Agbo M. Malaria in pregnancy: efficacy of a low dose of mefloquine in an area holoendemic for multi-drug resistant *Plasmodium falciparum*. *Ann Trop Med Parasitol* 1996; **90**(3): 265-8.

255. Ramsay AR, Msaki EP, Kennedy N, Ngowi FI, Gillespie SH. Evaluation of the safety and efficacy of micronized halofantrine in the treatment of semi-immune patients with acute, *Plasmodium falciparum* malaria. *Ann Trop Med Parasitol* 1996; **90**(5): 461-6.

256. Sowunmi A, Oduola AM, Ilesanmi AO, Salako LA. Open comparison of artemether and mefloquine in uncomplicated *Plasmodium falciparum* hyperparasitaemia in children. *Ann Trop Paediatr* 1996; **16**(1): 5-9.

257. Anabwani GM, Esamai FO, Menya DA. A randomised controlled trial to assess the relative efficacy of chloroquine, amodiaquine, halofantrine and Fansidar in the treatment of uncomplicated malaria in children. *East Afr Med J* 1996; **73**(3): 155-8.

258. Rukaria-Kaumbutho RM, Ojwang SB, Oyieke JB. Resistance to chloroquine therapy in pregnant women with malaria parasitemia. *Int J Gynaecol Obstet* 1996; **53**(3): 235-41.

259. Radloff PD, Philipps J, Nkeyi M, Hutchinson D, Kremsner PG. Atovaquone and proguanil for *Plasmodium falciparum* malaria. *Lancet* 1996; **347**(9014): 1511-4.

260. Steketee RW, Wirima JJ, Slutsker L, et al. Malaria parasite infection during pregnancy and at delivery in mother, placenta, and newborn: efficacy of chloroquine and mefloquine in rural Malawi. *Am J Trop Med Hyg* 1996; **55**(1 Suppl): 24-32.

261. Radloff PD, Philipps J, Nkeyi M, Sturchler D, Mittelholzer ML, Kremsner PG. Arteflene compared with mefloquine for treating *Plasmodium falciparum* malaria in children. *Am J Trop Med Hyg* 1996; **55**(3): 259-62.

262. Ezedinachi E. *In vivo* efficacy of chloroquine, halofantrine, pyrimethamine-sulfadoxine and qinghaosu (artesunate) in the treatment of malaria in Calabar, Nigeria. *Cent Afr J Med* 1996; **42**(4): 109-11.

263. Mahomva AI, Peterson DE, Rakata L. Chloroquine resistant falciparum malaria in Mutare District, eastern Zimbabwe. *Cent Afr J Med* 1996; **42**(4): 112-3.

264. Hassan Alin M, Ashton M, Kihamia CM, Mtey GJ, Bjorkman A. Multiple dose pharmacokinetics of oral artemisinin and comparison of its efficacy with that of oral artesunate in falciparum malaria patients. *Trans R Soc Trop Med Hyg* 1996; **90**(1): 61-5.

265. Onyiorah E, van Hensbroek MB, Jah MS, Greenwood B. Early clinical failures after pyrimethamine-sulfadoxine treatment of uncomplicated falciparum malaria. *Trans R Soc Trop Med Hyg* 1996; **90**(3): 307-8.

266. Rogier C, Brau R, Tall A, Cisse B, Trape JF. Reducing the oral quinine-quinidine-cinchonin (Quinimax) treatment of uncomplicated malaria to three days does not increase the recurrence of attacks among children living in a highly endemic area of Senegal. *Trans R Soc Trop Med Hyg* 1996; **90**(2): 175-8.

267. Nwanyanwu OC, Ziba C, Kazembe P, et al. Efficacy of sulphadoxine/pyrimethamine for *Plasmodium falciparum* malaria in Malawian children under five years of age. *Trop Med Int Health* 1996; **1**(2): 231-5.

268. Muller O, van Hensbroek MB, Jaffar S, et al. A randomized trial of chloroquine, amodiaquine and pyrimethamine-sulphadoxine in Gambian children with uncomplicated malaria. *Trop Med Int Health* 1996; **1**(1): 124-32.

269. Henry MC, Eggelte TA, Watson P, Docters van Leeuwen B, Bakker DA, Kluin J. Response of childhood malaria to chloroquine and Fansidar in an area of intermediate chloroquine resistance in Cote d'Ivoire. *Trop Med Int Health* 1996; **1**(5): 610-5.

270. Sowunmi A, Ilesanmi AO, Oduola AMJ, Omitowoju GO, Ojengbede OA. Efficacy of mefloquine in uncomplicated chloroquine-resistant falciparum malaria during pregnancy. *J Obstet Gynaecol* 1996; **16**(5): 362-3.

271. Amukoye E, Winstanley PA, Watkins WM, et al. Chlorproguanil-dapsone: effective treatment for uncomplicated falciparum malaria. *Antimicrob Agents Chemother* 1997; **41**(10): 2261-4.

272. Wakibara JV, Mboera LE, Ndawi BT. Malaria in Mvumi, central Tanzania and the *in vivo* response of *Plasmodium falciparum* to chloroquine and sulphadoxine pyrimethamine. *East Afr Med J* 1997; **74**(2): 69-71.

273. Falaschi F, Ansaloni L. Chloroquine versus pyrimethamine/sulphadoxine in the treatment of uncomplicated *P. falciparum* malaria in northern Kenya. *East Afr Med J* 1997; **74**(5): 275-7.

274. Sheng WD, Jiddawi MS, Hong XQ, Abdulla SM. Treatment of chloroquine-resistant malaria using pyrimethamine in combination with berberine, tetracycline or cotrimoxazole. *East Afr Med J* 1997; **74**(5): 283-4.

275. Touze JE, Perret JL, Nicolas X, et al. Efficacy of low-dose halofantrine for second treatment of uncomplicated falciparum malaria. *Lancet* 1997; **349**(9047): 255-6.

276. Chambon R, Lemardeley P, Boudin C, Ringwald P, Chandenier J. [Surveillance of the *in vivo* sensitivity of *Plasmodium falciparum* to antimalarial agents: the results of initial tests of the OCEAC Malaria Network]. *Med Trop (Mars)* 1997; **57**(4): 357-60.

277. von Seidlein L, Jaffar S, Pinder M, et al. Treatment of African children with uncomplicated falciparum malaria with a new antimalarial drug, CGP 56697. *J Infect Dis* 1997; **176**(4): 1113-6.

278. Sowunmi A, Oduola AM. Comparative efficacy of chloroquine/chlorpheniramine combination and mefloquine for the treatment of chloroquine-resistant *Plasmodium falciparum* malaria in Nigerian children. *Trans R Soc Trop Med Hyg* 1997; **91**(6): 689-93.

279. Antia-Obong OE, Alaribe AA, Young MU, Bassy A, Etim BV. Chloroquine-resistant *Plasmodium falciparum* among children in Calabar, south eastern Nigeria. *Trop Doct* 1997; **27**(3): 146-9.

280. Okoyeh JN, Lege-Oguntoye L, Ugbode RO, Ogunrinde GO. Responses of multidrug-resistant *Plasmodium falciparum* parasites to mefloquine in Nigerian children. *Trop Med Int Health* 1997; **2**(4): 319-24.

281. Vaillant M, Millet P, Luty A, et al. Therapeutic efficacy of clindamycin in combination with quinine for treating uncomplicated malaria in a village dispensary in Gabon. *Trop Med Int Health* 1997; **2**(9): 917-9.

282. Basco LK, Tahar R, Ringwald P. Molecular basis of *in vivo* resistance to sulfadoxine-pyrimethamine in African adult patients infected with *Plasmodium falciparum* malaria parasites. *Antimicrob Agents Chemother* 1998; **42**(7): 1811-4.

283. Sowunmi A, Oduola AM, Ogundahunsi OA, et al. Randomised trial of artemether versus artemether and mefloquine for the treatment of chloroquine/sufadoxine-pyrimethamine-resistant falciparum malaria during pregnancy. *J Obstet Gynaecol* 1998; **18**(4): 322-7.

284. Sowunmi A, Fehintola FA, Ogundahunsi OA, Arowojolu AO, Oduola AM. Efficacy of chloroquine plus chlorpheniramine in chloroquine-resistant falciparum malaria during pregnancy in Nigerian women: a preliminary study. *J Obstet Gynaecol* 1998; **18**(6): 524-7.

285. Lell B, Lehman LG, Schmidt-Ott JR, Sturchler D, Handschin J, Kremsner PG. Malaria chemotherapy trial at a minimal effective dose of mefloquine/sulfadoxine/pyrimethamine compared with equivalent doses of sulfadoxine/pyrimethamine or mefloquine alone. *Am J Trop Med Hyg* 1998; **58**(5): 619-24.

286. von Seidlein L, Bojang K, Jones P, et al. A randomized controlled trial of artemether/benflumetol, a new antimalarial and pyrimethamine/sulfadoxine in the treatment of uncomplicated falciparum malaria in African children. *Am J Trop Med Hyg* 1998; **58**(5): 638-44.

287. Barduagni P, Schwartz U, Nyamayaro W, Chauke TL. *In vivo* testing of the therapeutic efficacy of chloroquine on falciparum malaria infections in Chirundu, Mashonaland West, Zimbabwe. *Cent Afr J Med* 1998; **44**(10): 251-4.

288. Bojang KA, Schneider G, Forck S, et al. A trial of Fansidar plus chloroquine or Fansidar alone for the treatment of uncomplicated malaria in Gambian children. *Trans R Soc Trop Med Hyg* 1998; **92**(1): 73-6.

289. Hatz C, Abdulla S, Mull R, et al. Efficacy and safety of CGP 56697 (artemether and benflumetol) compared with chloroquine to treat acute falciparum malaria in Tanzanian children aged 1-5 years. *Trop Med Int Health* 1998; **3**(6): 498-504.

290. Parola P, Ali I, Djermakoye F, et al. [Chloroquine sensitivity of *Plasmodium falciparum* at the Gamkalley Clinic and the Nigerian armed forces PMI (Niamey, Niger)]. *Bull Soc Pathol Exot* 1999; **92**(5): 317-9.

291. Mulenga M, Sukwa TY, Canfield CJ, Hutchinson DB. Atovaquone and proguanil versus pyrimethamine/sulfadoxine for the treatment of acute falciparum malaria in Zambia. *Clin Ther* 1999; **21**(5): 841-52.

292. Mutanda LN. Assessment of drug resistance to the malaria parasite in residents of Kampala, Uganda. *East Afr Med J* 1999; **76**(8): 421-4.

293. van Agtmael M, Bouchaud O, Malvy D, et al. The comparative efficacy and tolerability of CGP 56697 (artemether + lumefantrine) versus halofantrine in the treatment of uncomplicated falciparum malaria in travellers returning from the Tropics to The Netherlands and France. *Int J Antimicrob Agents* 1999; **12**(2): 159-69.

294. Ezedinachi EN, Ekanem OJ, Chukwuani CM, et al. Efficacy and tolerability of a low-dose mefloquine-sulfadoxine-pyrimethamine combination compared with chloroquine in the treatment of acute malaria infection in a population with multiple drug-resistant *Plasmodium falciparum*. *Am J Trop Med Hyg* 1999; **61**(1): 114-9.

295. Anabwani G, Canfield CJ, Hutchinson DB. Combination atovaquone and proguanil hydrochloride vs. halofantrine for treatment of acute *Plasmodium falciparum* malaria in children. *Pediatr Infect Dis J* 1999; **18**(5): 456-61.

296. Doherty JF, Sadiq AD, Bayo L, et al. A randomized safety and tolerability trial of artesunate plus sulfadoxine--pyrimethamine versus sulfadoxine-pyrimethamine alone for the treatment of uncomplicated malaria in Gambian children. *Trans R Soc Trop Med Hyg* 1999; **93**(5): 543-6.

297. Sowunmi A, Fehintola FA, Adedeji AA, et al. Comparative efficacy of chloroquine plus chlorpheniramine alone and in a sequential combination with sulfadoxine-pyrimethamine, for the treatment of acute, uncomplicated, falciparum malaria in children. *Ann Trop Med Parasitol* 2000; **94**(3): 209-17.

298. Adou-Bryn KD, Kouassi D, Ouhon J, Assoumou A, Kone M. [Clinical trial of amodiaquine in the community of Attecoube (Abidjan, Cote d'Ivoire)(May-December 1955)]. *Bull Soc Pathol Exot* 2000; **93**(2): 115-8.

299. von Seidlein L, Milligan P, Pinder M, et al. Efficacy of artesunate plus pyrimethamine-sulphadoxine for uncomplicated malaria in Gambian children: a double-blind, randomised, controlled trial. *Lancet* 2000; **355**(9201): 352-7.

300. Ogutu BR, Smoak BL, Nduati RW, Mbori-Ngacha DA, Mwathe F, Shanks GD. The efficacy of pyrimethamine-sulfadoxine (Fansidar) in the treatment of uncomplicated *Plasmodium falciparum* malaria in Kenyan children. *Trans R Soc Trop Med Hyg* 2000; **94**(1): 83-4.

301. Sowunmi A, Ayede AI, Falade AG, et al. Randomized comparison of chloroquine and amodiaquine in the treatment of acute, uncomplicated, *Plasmodium falciparum* malaria in children. *Ann Trop Med Parasitol* 2001; **95**(6): 549-58.

302. Guiyedi V, Koko J, Bouyou Akotet M, et al. [Evaluation of efficacy and tolerance of amodiaquine versus chloroquine in the treatment of uncomplicated malaria outbreak in children of Gabon]. *Bull Soc Pathol Exot* 2001; **94**(3): 253-7.

303. Wang W, Yang W, Micha ST. Efficacy of dihydroartemisinin-mefloquine on acute uncomplicated falciparum malaria. *Chin Med J (Engl)* 2001; **114**(6): 612-3.

304. Athan E, Durrheim DN, Barnes K, Mngomezulu NM, Mabuza A, Govere J. Effectiveness of short-course quinine and single-dose sulfadoxine-pyrimethamine in the treatment of *Plasmodium falciparum* malaria in Mpumalanga Province, South Africa. *S Afr Med J* 2001; **91**(7): 592-4.

305. Mabuza A, Govere J, Durrheim D, et al. Therapeutic efficacy of sulfadoxine-pyrimethamine in uncomplicated *Plasmodium falciparum* malaria 3 years after introduction in Mpumalanga. *S Afr Med J* 2001; **91**(11): 975-8.

306. von Seidlein L, Jawara M, Coleman R, Doherty T, Walraven G, Targett G. Parasitaemia and gametocytaemia after treatment with chloroquine, pyrimethamine/sulfadoxine, and pyrimethamine/sulfadoxine combined with artesunate in young Gambians with uncomplicated malaria. *Trop Med Int Health* 2001; **6**(2): 92-8.

307. Sowunmi A. A randomized comparison of chloroquine, amodiaquine and their combination with pyrimethamine-sulfadoxine in the treatment of acute, uncomplicated, *Plasmodium falciparum* malaria in children. *Ann Trop Med Parasitol* 2002; **96**(3): 227-38.

308. Adou-Bryn KD, Krelo K, Akoussi CF, et al. [Therapeutic efficacy of chloroquine in the uncomplicated malaria due to *Plasmodium falciparum* in hospital patients in central Ivory Coast (1997-2000)]. *Bull Soc Pathol Exot* 2002; **95**(4): 262-4.

309. Basco LK, Same-Ekobo A, Ngane VF, et al. Therapeutic efficacy of sulfadoxine-pyrimethamine, amodiaquine and the sulfadoxine-pyrimethamine-amodiaquine combination against uncomplicated *Plasmodium falciparum* malaria in young children in Cameroon. *Bull World Health Organ* 2002; **80**(7): 538-45.

310. Adjuik M, Agnamey P, Babiker A, et al. Amodiaquine-artesunate versus amodiaquine for uncomplicated *Plasmodium falciparum* malaria in African children: a randomised, multicentre trial. *Lancet* 2002; **359**(9315): 1365-72.

311. Henry MC, Niangue J, Kone M. [Which medication should be used to treat uncomplicated malaria when chloroquine becomes ineffective in Western Cote d'Ivoire?]. *Med Trop (Mars)* 2002; **62**(1): 55-7.

312. Schellenberg D, Kahigwa E, Drakeley C, et al. The safety and efficacy of sulfadoxine-pyrimethamine, amodiaquine, and their combination in the treatment of uncomplicated *Plasmodium falciparum* malaria. *Am J Trop Med Hyg* 2002; **67**(1): 17-23.

313. Kofoed PE, Lopez F, Johansson P, et al. Treatment of children with *Plasmodium falciparum* malaria with chloroquine in Guinea-Bissau. *Am J Trop Med Hyg* 2002; **67**(1): 28-31.

314. Kofoed PE, Co F, Johansson P, et al. Treatment of uncomplicated malaria in children in Guinea-Bissau with chloroquine, quinine, and sulfadoxine-pyrimethamine. *Trans R Soc Trop Med Hyg* 2002; **96**(3): 304-9.

315. Massougbodji A, Kone M, Kinde-Gazard D, Same-Ekobo A, Cambon N, Mueller EA. A randomized, double-blind study on the efficacy and safety of a practical three-day regimen with artesunate and mefloquine for the treatment of uncomplicated *Plasmodium falciparum* malaria in Africa. *Trans R Soc Trop Med Hyg* 2002; **96**(6): 655-9.

316. Checchi F, Durand R, Balkan S, et al. High *Plasmodium falciparum* resistance to chloroquine and sulfadoxine-pyrimethamine in Harper, Liberia: results *in vivo* and analysis of point mutations. *Trans R Soc Trop Med Hyg* 2002; **96**(6): 664-9.

317. Checchi F, Balkan S, Vonhm BT, et al. Efficacy of amodiaquine for uncomplicated *Plasmodium falciparum* malaria in Harper, Liberia. *Trans R Soc Trop Med Hyg* 2002; **96**(6): 670-3.

318. Driessen GJ, van Kerkhoven S, Schouwenberg BJ, Bonsu G, Verhave JP. Sulphadoxine/pyrimethamine: an appropriate first-line alternative for the treatment of uncomplicated falciparum malaria in Ghanaian children under 5 years of age. *Trop Med Int Health* 2002; **7**(7): 577-83.

319. Tarimo DS, Minjas JN, Bygbjerg IC. Sulfadoxine-pyrimethamine monotherapy in Tanzanian children gives rapid parasite clearance but slow fever clearance that is improved by chloroquine in combination therapy. *Trop Med Int Health* 2002; **7**(7): 592-8.

320. Sowunmi A. A randomized comparison of chloroquine and chloroquine plus ketotifen in the treatment of acute, uncomplicated, *Plasmodium falciparum* malaria in children. *Ann Trop Med Parasitol* 2003; **97**(2): 103-17.

321. Bergeri I, Zoguereh DD, Madji N, et al. [*In vivo* evaluation of chloroquine therapeutic efficacy in uncomplicated *Plasmodium falciparum* malaria in Central African Republic ine 1997 and 1998]. *Bull Soc Pathol Exot* 2003; **96**(1): 29-34.

322. Borrmann S, Faucher JF, Bagaphou T, et al. Atovaquone and proguanil versus amodiaquine for the treatment of *Plasmodium falciparum* malaria in African infants and young children. *Clin Infect Dis* 2003; **37**(11): 1441-7.

323. Rwagacondo CE, Niyitegeka F, Sarushi J, et al. Efficacy of amodiaquine alone and combined with sulfadoxine-pyrimethamine and of sulfadoxine pyrimethamine combined with artesunate. *Am J Trop Med Hyg* 2003; **68**(6): 743-7.

324. Sutherland CJ, Drakeley CJ, Obisike U, et al. The addition of artesunate to chloroquine for treatment of *Plasmodium falciparum* malaria in Gambian children delays, but does not prevent treatment failure. *Am J Trop Med Hyg* 2003; **69**(1): 19-25.

325. Priotto G, Kabakyenga J, Pinoges L, et al. Artesunate and sulfadoxine-pyrimethamine combinations for the treatment of uncomplicated *Plasmodium falciparum* malaria in Uganda: a randomized, double-blind, placebo-controlled trial. *Trans R Soc Trop Med Hyg* 2003; **97**(3): 325-30.

326. Sirima SB, Tiono AB, Konate A, et al. Efficacy of artesunate plus chloroquine for the treatment of uncomplicated malaria in children in Burkina Faso: a double-blind, randomized, controlled trial. *Trans R Soc Trop Med Hyg* 2003; **97**(3): 345-9.

327. Kofoed PE, Poulsen A, Co F, Hedegaard K, Aaby P, Rombo L. No benefits from combining chloroquine with artesunate for three days for treatment of *Plasmodium falciparum* in Guinea-Bissau. *Trans R Soc Trop Med Hyg* 2003; **97**(4): 429-33.

328. Obonyo CO, Ochieng F, Taylor WR, et al. Artesunate plus sulfadoxine-pyrimethamine for uncomplicated malaria in Kenyan children: a randomized, double-blind, placebo-controlled trial. *Trans R Soc Trop Med Hyg* 2003; **97**(5): 585-91.

329. Gil VS, Ferreira MC, d'Alva FS, et al. Efficacy of artesunate plus chloroquine for uncomplicated malaria in children in Sao Tome and Principe: a double-blind, randomized, controlled trial. *Trans R Soc Trop Med Hyg* 2003; **97**(6): 703-6.

330. Bousema JT, Gouagna LC, Meutstege AM, et al. Treatment failure of pyrimethamine-sulphadoxine and induction of *Plasmodium falciparum* gametocytaemia in children in western Kenya. *Trop Med Int Health* 2003; **8**(5): 427-30.

331. Plowe CV, Kublin JG, Dzinjalamala FK, et al. Sustained clinical efficacy of sulfadoxine-pyrimethamine for uncomplicated falciparum malaria in Malawi after 10 years as first line treatment: five year prospective study. *BMJ* 2004; **328**(7439): 545.

332. Kassi RR, Yavo W, Mokak DG, et al. [Efficacy of chloroquine in treatment of non-complicated malaria with *Plasmodium falciparum* in children at the San Pedro dispensary in Cote d'Ivoire]. *Bull Soc Pathol Exot* 2004; **97**(2): 129-30.

333. Assi SB, Henry MC, Nzeyimana I, Kone M. [Therapeutic efficiency of chloroquine in the savannah region in the north of Cote d'Ivoire (1997)]. *Bull Soc Pathol Exot* 2004; **97**(3): 177-9.

334. Djaman JA, Kauffy PC, Yavo W, Basco LK, Kone M. [*In vivo* evaluation of sulfadoxine-pyrimethamine efficacy during uncomplicated falciparum malaria in children of Yopougon (Abidjan, Cote d'Ivoire)]. *Bull Soc Pathol Exot* 2004; **97**(3): 180-2.

335. Fehintola FA, Adedeji AA, Tambo E, Fateye BB, Happi TC, Sowunmi A. Cotrimoxazole in the treatment of acute uncomplicated falciparum malaria in nigerian children : a controlled clinical trial. *Clin Drug Investig* 2004; **24**(3): 149-55.

336. Staedke SG, Mpimbaza A, Kamya MR, Nzarubara BK, Dorsey G, Rosenthal PJ. Combination treatments for uncomplicated falciparum malaria in Kampala, Uganda: randomised clinical trial. *Lancet* 2004; **364**(9449): 1950-7.

337. Adoubryn KD, Ouhon J, Yapo CG, et al. [Revaluation of chloroquine therapeutic efficacy on children with uncomplicated *Plasmodium falciparum* malaria in eastern Ivory Coast (1999-2002)]. *Med Mal Infect* 2004; **34**(3): 127-31.

338. Borrmann S, Adegnika AA, Matsiegui PB, et al. Fosmidomycin-clindamycin for *Plasmodium falciparum* Infections in African children. *J Infect Dis* 2004; **189**(5): 901-8.

339. Borrmann S, Issifou S, Esser G, et al. Fosmidomycin-clindamycin for the treatment of *Plasmodium falciparum* malaria. *J Infect Dis* 2004; **190**(9): 1534-40.

340. Talisuna AO, Nalunkuma-Kazibwe A, Bakyaita N, et al. Efficacy of sulphadoxine-pyrimethamine alone or combined with amodiaquine or chloroquine for the treatment of uncomplicated falciparum malaria in Ugandan children. *Trop Med Int Health* 2004; **9**(2): 222-9.

341. Barennes H, Nagot N, Valea I, et al. A randomized trial of amodiaquine and artesunate alone and in combination for the treatment of uncomplicated falciparum malaria in children from Burkina Faso. *Trop Med Int Health* 2004; **9**(4): 438-44.

342. Checchi F, Piola P, Kosack C, et al. Antimalarial efficacy of sulfadoxine-pyrimethamine, amodiaquine and a combination of chloroquine plus sulfadoxine-pyrimethamine in Bundi Bugyo, western Uganda. *Trop Med Int Health* 2004; **9**(4): 445-50.

343. Sowunmi A, Fehintola FA, Adedeji AA, et al. Open randomized study of pyrimethamine-sulphadoxine vs. pyrimethamine-sulphadoxine plus probenecid for the treatment of uncomplicated *Plasmodium falciparum* malaria in children. *Trop Med Int Health* 2004; **9**(5): 606-14.

344. Rwagacondo CE, Karema C, Mugisha V, et al. Is amodiaquine failing in Rwanda? Efficacy of amodiaquine alone and combined with artesunate in children with uncomplicated malaria. *Trop Med Int Health* 2004; **9**(10): 1091-8.

345. Koram KA, Abuaku B, Duah N, Quashie N. Comparative efficacy of antimalarial drugs including ACTs in the treatment of uncomplicated malaria among children under 5 years in Ghana. *Acta Trop* 2005; **95**(3): 194-203.

346. Borrmann S, Adegnika AA, Moussavou F, et al. Short-course regimens of artesunate-fosmidomycin in treatment of uncomplicated *Plasmodium falciparum* malaria. *Antimicrob Agents Chemother* 2005; **49**(9): 3749-54.

347. Adjetey TA, Affoumou GB, Loukou DD, et al. [Evaluation of the therapeutic efficacy of amodiaquine versus chloroquine in the treatment of uncomplicated malaria in Abie, Cote-d'Ivoire]. *Bull Soc Pathol Exot* 2005; **98**(3): 193-6.

348. Ramharter M, Oyakhirome S, Klein Klouwenberg P, et al. Artesunate-clindamycin versus quinine-clindamycin in the treatment of *Plasmodium falciparum* malaria: a randomized controlled trial. *Clin Infect Dis* 2005; **40**(12): 1777-84.

349. Martensson A, Stromberg J, Sisowath C, et al. Efficacy of artesunate plus amodiaquine versus that of artemether-lumefantrine for the treatment of uncomplicated childhood *Plasmodium falciparum* malaria in Zanzibar, Tanzania. *Clin Infect Dis* 2005; **41**(8): 1079-86.

350. Achidi EA, Ajua A, Kimbi HK, Sinju CM. *In vivo* efficacy study of quinine sulphate in the treatment of uncomplicated *P. falciparum* malaria in patients from Southwestern Cameroon. *East Afr Med J* 2005; **82**(4): 181-5.

351. Piola P, Fogg C, Bajunirwe F, et al. Supervised versus unsupervised intake of six-dose artemether-lumefantrine for treatment of acute, uncomplicated *Plasmodium falciparum* malaria in Mbarara, Uganda: a randomised trial. *Lancet* 2005; **365**(9469): 1467-73.

352. Mutabingwa TK, Anthony D, Heller A, et al. Amodiaquine alone, amodiaquine+sulfadoxine-pyrimethamine, amodiaquine+artesunate, and artemether-lumefantrine for outpatient treatment of malaria in Tanzanian children: a four-arm randomised effectiveness trial. *Lancet* 2005; **365**(9469): 1474-80.

353. Mugittu K, Abdulla S, Falk N, et al. Efficacy of sulfadoxine-pyrimethamine in Tanzania after two years as first-line drug for uncomplicated malaria: assessment protocol and implication for treatment policy strategies. *Malar J* 2005; **4**: 55.

354. Sutherland CJ, Ord R, Dunyo S, et al. Reduction of malaria transmission to Anopheles mosquitoes with a six-dose regimen of co-artemether. *PLoS Med* 2005; **2**(4): e92.

355. Yeka A, Banek K, Bakyaita N, et al. Artemisinin versus nonartemisinin combination therapy for uncomplicated malaria: randomized clinical trials from four sites in Uganda. *PLoS Med* 2005; **2**(7): e190.

356. Mabuza A, Govere J, La Grange K, et al. Therapeutic efficacy of sulfadoxine-pyrimethamine for *Plasmodium falciparum* malaria. *S Afr Med J* 2005; **95**(5): 346-9.

357. Bakyaita N, Dorsey G, Yeka A, et al. Sulfadoxine-pyrimethamine plus chloroquine or amodiaquine for uncomplicated falciparum malaria: a randomized, multisite trial to guide national policy in Uganda. *Am J Trop Med Hyg* 2005; **72**(5): 573-80.

358. Adegnika AA, Breitling LP, Agnandji ST, et al. Effectiveness of quinine monotherapy for the treatment of *Plasmodium falciparum* infection in pregnant women in Lambarene, Gabon. *Am J Trop Med Hyg* 2005; **73**(2): 263-6.

359. Schwarz NG, Oyakhirome S, Potschke M, et al. 5-day nonobserved artesunate monotherapy for treating uncomplicated Falciparum malaria in young Gabonese children. *Am J Trop Med Hyg* 2005; **73**(4): 705-9.

360. Zongo I, Dorsey G, Rouamba N, et al. Amodiaquine, sulfadoxine-pyrimethamine, and combination therapy for uncomplicated falciparum malaria: a randomized controlled trial from Burkina Faso. *Am J Trop Med Hyg* 2005; **73**(5): 826-32.

361. Falade C, Makanga M, Premji Z, Ortmann CE, Stockmeyer M, de Palacios PI. Efficacy and safety of artemether-lumefantrine (Coartem) tablets (six-dose regimen) in African infants and children with acute, uncomplicated falciparum malaria. *Trans R Soc Trop Med Hyg* 2005; **99**(6): 459-67.

362. Guthmann JP, Ampuero J, Fortes F, et al. Antimalarial efficacy of chloroquine, amodiaquine, sulfadoxine-pyrimethamine, and the combinations of amodiaquine + artesunate and sulfadoxine-pyrimethamine + artesunate in Huambo and Bie provinces, central Angola. *Trans R Soc Trop Med Hyg* 2005; **99**(7): 485-92.

363. Depoortere E, Guthmann JP, Presse J, et al. Efficacy and effectiveness of the combination of sulfadoxine/pyrimethamine and a 3-day course of artesunate for the treatment of uncomplicated falciparum malaria in a refugee settlement in Zambia. *Trop Med Int Health* 2005; **10**(2): 139-45.

364. Checchi F, Roddy P, Kamara S, et al. Evidence basis for antimalarial policy change in Sierra Leone: five *in vivo* efficacy studies of chloroquine, sulphadoxine-pyrimethamine and amodiaquine. *Trop Med Int Health* 2005; **10**(2): 146-53.

365. Mockenhaupt FP, Ehrhardt S, Dzisi SY, et al. A randomized, placebo-controlled, double-blind trial on sulfadoxine-pyrimethamine alone or combined with artesunate or amodiaquine in uncomplicated malaria. *Trop Med Int Health* 2005; **10**(6): 512-20.

366. Sowunmi A, Fehintola FA, Adedeji AA, et al. Open randomized study of artesunate-amodiaquine vs. chloroquine-pyrimethamine-sulfadoxine for the treatment of uncomplicated *Plasmodium falciparum* malaria in Nigerian children. *Trop Med Int Health* 2005; **10**(11): 1161-70.

367. Obua C, Gustafsson LL, Aguttu C, et al. Improved efficacy with amodiaquine instead of chloroquine in sulfadoxine/pyrimethamine combination treatment of falciparum malaria in Uganda: experience with fixed-dose formulation. *Acta Trop* 2006; **100**(1-2): 142-50.

368. Kofoed PE, Rodrigues A, Aaby P, Rombo L. Continued efficacy of sulfadoxine-pyrimethamine as second line treatment for malaria in children in Guinea-Bissau. *Acta Trop* 2006; **100**(3): 213-7.

369. Borrmann S, Lundgren I, Oyakhirome S, et al. Fosmidomycin plus clindamycin for treatment of pediatric patients aged 1 to 14 years with *Plasmodium falciparum* malaria. *Antimicrob Agents Chemother* 2006; **50**(8): 2713-8.

370. Bhatt KM, Samia BM, Bhatt SM, Wasunna KM. Efficacy and safety of an artesunate/mefloquine combination, (artequin) in the treatment of uncomplicated *P. falciparum* malaria in Kenya. *East Afr Med J* 2006; **83**(5): 236-42.

371. Bousema JT, Schneider P, Gouagna LC, et al. Moderate effect of artemisinin-based combination therapy on transmission of *Plasmodium falciparum*. *J Infect Dis* 2006; **193**(8): 1151-9.

372. Tagbor H, Bruce J, Browne E, Randal A, Greenwood B, Chandramohan D. Efficacy, safety, and tolerability of amodiaquine plus sulphadoxine-pyrimethamine used alone or in combination for malaria treatment in pregnancy: a randomised trial. *Lancet* 2006; **368**(9544): 1349-56.

373. Coulibaly SO, Nezien D, Traore S, Kone B, Magnussen P. Therapeutic efficacy of sulphadoxine-pyrimethamine and chloroquine for the treatment of uncomplicated malaria in pregnancy in Burkina Faso. *Malar J* 2006; **5**: 49.

374. Mulenga M, VangGeertruyden JP, Mwananyanda L, et al. Safety and efficacy of lumefantrine-artemether (Coartem) for the treatment of uncomplicated *Plasmodium falciparum* malaria in Zambian adults. *Malar J* 2006; **5**: 73.

375. Chanda P, Hawela M, Kango M, Sipilanyambe N. Assessment of the therapeutic efficacy of a paediatric formulation of artemether-lumefantrine (Coartesiane) for the treatment of uncomplicated *Plasmodium falciparum* in children in Zambia. *Malar J* 2006; **5**: 75.

376. van den Broek I, Kitz C, Al Attas S, Libama F, Balasegaram M, Guthmann JP. Efficacy of three artemisinin combination therapies for the treatment of uncomplicated *Plasmodium falciparum* malaria in the Republic of Congo. *Malar J* 2006; **5**: 113.

377. Dunyo S, Ord R, Hallett R, et al. Randomised trial of chloroquine/sulphadoxine-pyrimethamine in Gambian children with malaria: impact against multidrug-resistant *P. falciparum*. *PLoS Clin Trials* 2006; **1**(3): e14.

378. Bukirwa H, Yeka A, Kamya MR, et al. Artemisinin combination therapies for treatment of uncomplicated malaria in Uganda. *PLoS Clin Trials* 2006; **1**(1): e7.

379. Ouma P, Parise ME, Hamel MJ, et al. A randomized controlled trial of folate supplementation when treating malaria in pregnancy with sulfadoxine-pyrimethamine. *PLoS Clin Trials* 2006; **1**(6): e28.

380. Guthmann JP, Cohuet S, Rigutto C, et al. High efficacy of two artemisinin-based combinations (artesunate + amodiaquine and artemether + lumefantrine) in Caala, Central Angola. *Am J Trop Med Hyg* 2006; **75**(1): 143-5.

381. Sagara I, Dicko A, Djimde A, et al. A randomized trial of artesunate-sulfamethoxypyrazine-pyrimethamine versus artemether-lumefantrine for the treatment of uncomplicated *Plasmodium falciparum* malaria in Mali. *Am J Trop Med Hyg* 2006; **75**(4): 630-6.

382. Grandesso F, Bachy C, Donam I, et al. Efficacy of chloroquine, sulfadoxine-pyrimethamine and amodiaquine for treatment of uncomplicated *Plasmodium falciparum* malaria among children under five in Bongor and Koumra, Chad. *Trans R Soc Trop Med Hyg* 2006; **100**(5): 419-26.

383. de Radigues X, Diallo KI, Diallo M, et al. Efficacy of chloroquine and sulfadoxine/pyrimethamine for the treatment of uncomplicated falciparum malaria in Koumantou, Mali. *Trans R Soc Trop Med Hyg* 2006; **100**(11): 1013-8.

384. Karema C, Fanello CI, van Overmeir C, et al. Safety and efficacy of dihydroartemisinin/piperaquine (Artekin) for the treatment of uncomplicated *Plasmodium falciparum* malaria in Rwandan children. *Trans R Soc Trop Med Hyg* 2006; **100**(12): 1105-11.

385. Grandesso F, Hagerman A, Kamara S, et al. Low efficacy of the combination artesunate plus amodiaquine for uncomplicated falciparum malaria among children under 5 years in Kailahun, Sierra Leone. *Trop Med Int Health* 2006; **11**(7): 1017-21.

386. Swarthout TD, van den Broek IV, Kayembe G, Montgomery J, Pota H, Roper C. Artesunate + amodiaquine and artesunate + sulphadoxine-pyrimethamine for treatment of uncomplicated malaria in Democratic Republic of Congo: a clinical trial with determination of sulphadoxine and pyrimethamine-resistant haplotypes. *Trop Med Int Health* 2006; **11**(10): 1503-11.

387. Matsiegui PB, Missinou MA, Necek M, Issifou S, Kremsner PG. Short course of quinine plus a single dose of sulfadoxine/pyrimethamine for *Plasmodium falciparum* malaria. *Wien Klin Wochenschr* 2006; **118**(19-20): 610-4.

388. Ndounga M, Mayengue PI, Tahar R, et al. Efficacy of sulfadoxine-pyrimethamine, amodiaquine, and sulfadoxine-pyrimethamine-amodiaquine combination for the treatment of uncomplicated falciparum malaria in the urban and suburban areas of Brazzaville (Congo). *Acta Trop* 2007; **103**(3): 163-71.

389. Tinto H, Ouedraogo JB, Zongo I, et al. Sulfadoxine-pyrimethamine efficacy and selection of *Plasmodium falciparum* DHFR mutations in Burkina Faso before its introduction as intermittent preventive treatment for pregnant women. *Am J Trop Med Hyg* 2007; **76**(4): 608-13.

390. Sowunmi A, Balogun T, Gbotosho GO, Happi CT, Adedeji AA, Fehintola FA. Activities of amodiaquine, artesunate, and artesunate-amodiaquine against asexual- and sexual-stage parasites in falciparum malaria in children. *Antimicrob Agents Chemother* 2007; **51**(5): 1694-9.

391. Oyakhirome S, Issifou S, Pongratz P, et al. Randomized controlled trial of fosmidomycin-clindamycin versus sulfadoxine-pyrimethamine in the treatment of *Plasmodium falciparum* malaria. *Antimicrob Agents Chemother* 2007; **51**(5): 1869-71.

392. Zongo I, Dorsey G, Rouamba N, et al. Randomized comparison of amodiaquine plus sulfadoxine-pyrimethamine, artemether-lumefantrine, and dihydroartemisinin-piperaquine for the treatment of uncomplicated *Plasmodium falciparum* malaria in Burkina Faso. *Clin Infect Dis* 2007; **45**(11): 1453-61.

393. Dorsey G, Staedke S, Clark TD, et al. Combination therapy for uncomplicated falciparum malaria in Ugandan children: a randomized trial. *JAMA* 2007; **297**(20): 2210-9.

394. Zongo I, Dorsey G, Rouamba N, et al. Artemether-lumefantrine versus amodiaquine plus sulfadoxine-pyrimethamine for uncomplicated falciparum malaria in Burkina Faso: a randomised non-inferiority trial. *Lancet* 2007; **369**(9560): 491-8.

395. Oyakhirome S, Potschke M, Schwarz NG, et al. Artesunate--amodiaquine combination therapy for falciparum malaria in young Gabonese children. *Malar J* 2007; **6**: 29.

396. Bonnet M, Roper C, Felix M, Coulibaly L, Kankolongo GM, Guthmann JP. Efficacy of antimalarial treatment in Guinea: *in vivo* study of two artemisinin combination therapies in Dabola and molecular markers of resistance to sulphadoxine-pyrimethamine in N'Zerekore. *Malar J* 2007; **6**: 54.

397. Faye B, Ndiaye JL, Ndiaye D, Dieng Y, Faye O, Gaye O. Efficacy and tolerability of four antimalarial combinations in the treatment of uncomplicated *Plasmodium falciparum* malaria in Senegal. *Malar J* 2007; **6**: 80.

398. Kabanywanyi AM, Mwita A, Sumari D, Mandike R, Mugittu K, Abdulla S. Efficacy and safety of artemisinin-based antimalarial in the treatment of uncomplicated malaria in children in southern Tanzania. *Malar J* 2007; **6**: 146.

399. Nahum A, Erhart A, Gazard D, et al. Adding artesunate to sulphadoxine-pyrimethamine greatly improves the treatment efficacy in children with uncomplicated falciparum malaria on the coast of Benin, West Africa. *Malar J* 2007; **6**: 170.

400. Menan EI, Yavo W, Oga SS, et al. [Open radomized study comparing chloroquine and amodiaquine for treatment of uncomplicated *Plasmodium falciparum* malaria in children in Aboisso, Cote d' Ivoire]. *Med Trop (Mars)* 2007; **67**(3): 309-10.

401. Kamya MR, Yeka A, Bukirwa H, et al. Artemether-lumefantrine versus dihydroartemisinin-piperaquine for treatment of malaria: a randomized trial. *PLoS Clin Trials* 2007; **2**(5): e20.

402. Kalilani L, Mofolo I, Chaponda M, et al. A randomized controlled pilot trial of azithromycin or artesunate added to sulfadoxine-pyrimethamine as treatment for malaria in pregnant women. *PLoS One* 2007; **2**(11): e1166.

403. Fryauff DJ, Owusu-Agyei S, Utz G, et al. Mefloquine treatment for uncomplicated falciparum malaria in young children 6-24 months of age in northern Ghana. *Am J Trop Med Hyg* 2007; **76**(2): 224-31.

404. Sowunmi A, Gbotosho GO, Happi CT, et al. Therapeutic efficacy and effects of artemether-lumefantrine and amodiaquine-sulfalene-pyrimethamine on gametocyte carriage in children with uncomplicated *Plasmodium falciparum* malaria in southwestern Nigeria. *Am J Trop Med Hyg* 2007; **77**(2): 235-41.

405. Rulisa S, Gatarayiha JP, Kabarisa T, Ndayisaba G. Comparison of different artemisinin-based combinations for the treatment of *Plasmodium falciparum* malaria in children in Kigali, Rwanda, an area of resistance to sulfadoxine-pyrimethamine: artesunate plus sulfadoxine/pyrimethamine versus artesunate plus sulfamethoxypyrazine/pyrimethamine. *Am J Trop Med Hyg* 2007; **77**(4): 612-6.

406. Kofoed PE, Ursing J, Poulsen A, et al. Different doses of amodiaquine and chloroquine for treatment of uncomplicated malaria in children in Guinea-Bissau: implications for future treatment recommendations. *Trans R Soc Trop Med Hyg* 2007; **101**(3): 231-8.

407. Fanello CI, Karema C, van Doren W, Van Overmeir C, Ngamije D, D'Alessandro U. A randomised trial to assess the safety and efficacy of artemether-lumefantrine (Coartem) for the treatment of uncomplicated *Plasmodium falciparum* malaria in Rwanda. *Trans R Soc Trop Med Hyg* 2007; **101**(4): 344-50.

408. Oduro AR, Anyorigiya T, Koram K, Anto F, Atobrah P, Hodgson A. Amodiaquine in future combination treatment of malaria in Ghana. *Trop Doct* 2007; **37**(3): 154-6.

409. Aubouy A, Fievet N, Bertin G, et al. Dramatically decreased therapeutic efficacy of chloroquine and sulfadoxine-pyrimethamine, but not mefloquine, in southern Benin. *Trop Med Int Health* 2007; **12**(7): 886-94.

410. Tagbor H, Bruce J, Ord R, et al. Comparison of the therapeutic efficacy of chloroquine and sulphadoxine-pyremethamine in children and pregnant women. *Trop Med Int Health* 2007; **12**(11): 1288-97.

411. Ndounga M, Tahar R, Basco LK, Casimiro PN, Malonga DA, Ntoumi F. Therapeutic efficacy of sulfadoxine-pyrimethamine and the prevalence of molecular markers of resistance in under 5-year olds in Brazzaville, Congo. *Trop Med Int Health* 2007; **12**(10): 1164-71.

412. Fehintola FA, Adedeji AA, Balogun ST, Sowunmi A. Comparative study of efficacy of amodiaquine-cotrimoxazole and amodiaquine-pyrimethamine-sulphadoxine in the treatment of malaria in Nigerian children. *J Med Sci* 2007; **7**(8): 1290-6.

413. Agomo PU, Mustapha RAS, Omoloye BG, et al. Efficacy and safety of Artesunate + Mefloquine (Artequin) in the treatment of uncomplicated Falciparum malaria in Ijede community, Ikorodu LGA, Lagost State, Nigeria. *J Med Sci* 2007; **7**(5): 816-24.

414. Brasseur P, Agnamey P, Gaye O, Vaillant M, Taylor WR, Olliaro PL. Efficacy and safety of artesunate plus amodiaquine in routine use for the treatment of uncomplicated malaria in Casamance, southern Senegal. *Malar J* 2007; **6**: 150.

415. Oduro AR, Anyorigiya T, Anto F, et al. A randomized, comparative study of supervised and unsupervised artesunate-amodiaquine, for the treatment of uncomplicated malaria in Ghana. *Ann Trop Med Parasitol* 2008; **102**(7): 565-76.

416. Koram K, Quaye L, Abuaku B. Efficacy of amodiaquine/artesunate combination therapy for uncomplicated malaria in children under five years in ghana. *Ghana Med J* 2008; **42**(2): 55-60.

417. Penali LK, Jansen FH. Single-day, three-dose treatment with fixed dose combination artesunate/sulfamethoxypyrazine/pyrimethamine to cure *Plasmodium falciparum* malaria. *Int J Infect Dis* 2008; **12**(4): 430-7.

418. Fehintola FA, Adedeji AA, Gbotosho GO, et al. Effects of artesunate-cotrimoxazole and amodiaquine-artesunate against asexual and sexual stages of *Plasmodium falciparum* malaria in Nigerian children. *J Infect Chemother* 2008; **14**(3): 188-94.

419. Kaye DK, Nshemerirwe R, Mutyaba TS, Ndeezi G. A randomized clinical trial comparing safety, clinical and parasitological response to artemether-lumefantrine and chlorproguanil-dapsone in treatment of uncomplicated malaria in pregnancy in Mulago hospital, Uganda. *J Infect Dev Ctries* 2008; **2**(2): 135-9.

420. Abdulla S, Sagara I, Borrmann S, et al. Efficacy and safety of artemether-lumefantrine dispersible tablets compared with crushed commercial tablets in African infants and children with uncomplicated malaria: a randomised, single-blind, multicentre trial. *Lancet* 2008; **372**(9652): 1819-27.

421. Nsimba B, Guiyedi V, Mabika-Mamfoumbi M, et al. Sulphadoxine/pyrimethamine versus amodiaquine for treating uncomplicated childhood malaria in Gabon: a randomized trial to guide national policy. *Malar J* 2008; **7**: 31.

422. Mandi G, Mockenhaupt FP, Coulibaly B, Meissner P, Muller O. Efficacy of amodiaquine in the treatment of uncomplicated falciparum malaria in young children of rural north-western Burkina Faso. *Malar J* 2008; **7**: 58.

423. Maiteki-Sebuguzi C, Jagannathan P, Yau VM, et al. Safety and tolerability of combination antimalarial therapies for uncomplicated falciparum malaria in Ugandan children. *Malar J* 2008; **7**: 106.

424. Adjei GO, Kurtzhals JA, Rodrigues OP, et al. Amodiaquine-artesunate vs artemether-lumefantrine for uncomplicated malaria in Ghanaian children: a randomized efficacy and safety trial with one year follow-up. *Malar J* 2008; **7**: 127.

425. Agomo PU, Meremikwu MM, Watila IM, et al. Efficacy, safety and tolerability of artesunate-mefloquine in the treatment of uncomplicated Plasmodium falciparum malaria in four geographic zones of Nigeria. *Malar J* 2008; **7**: 172.

426. Mens PF, Sawa P, van Amsterdam SM, et al. A randomized trial to monitor the efficacy and effectiveness by QT-NASBA of artemether-lumefantrine versus dihydroartemisinin-piperaquine for treatment and transmission control of uncomplicated *Plasmodium falciparum* malaria in western Kenya. *Malar J* 2008; **7**: 237.

427. Falade CO, Ogunkunle OO, Dada-Adegbola HO, et al. Evaluation of the efficacy and safety of artemether-lumefantrine in the treatment of acute uncomplicated *Plasmodium falciparum* malaria in Nigerian infants and children. *Malar J* 2008; **7**: 246.

428. Kobbe R, Klein P, Adjei S, et al. A randomized trial on effectiveness of artemether-lumefantrine versus artesunate plus amodiaquine for unsupervised treatment of uncomplicated *Plasmodium falciparum* malaria in Ghanaian children. *Malar J* 2008; **7**: 261.

429. Juma EA, Obonyo CO, Akhwale WS, Ogutu BR. A randomized, open-label, comparative efficacy trial of artemether-lumefantrine suspension versus artemether-lumefantrine tablets for treatment of uncomplicated *Plasmodium falciparum* malaria in children in western Kenya. *Malar J* 2008; **7**: 262.

430. Ogungbamigbe TO, Ojurongbe O, Ogunro PS, Okanlawon BM, Kolawole SO. Chloroquine resistant *Plasmodium falciparum* malaria in Osogbo Nigeria: efficacy of amodiaquine + sulfadoxine-pyrimethamine and chloroquine + chlorpheniramine for treatment. *Mem Inst Oswaldo Cruz* 2008; **103**(1): 79-84.

431. Bell DJ, Nyirongo SK, Mukaka M, et al. Sulfadoxine-pyrimethamine-based combinations for malaria: a randomised blinded trial to compare efficacy, safety and selection of resistance in Malawi. *PLoS One* 2008; **3**(2): e1578.

432. Zoungrana A, Coulibaly B, Sie A, et al. Safety and efficacy of methylene blue combined with artesunate or amodiaquine for uncomplicated falciparum malaria: a randomized controlled trial from Burkina Faso. *PLoS One* 2008; **3**(2): e1630.

433. Yeka A, Dorsey G, Kamya MR, et al. Artemether-lumefantrine versus dihydroartemisinin-piperaquine for treating uncomplicated malaria: a randomized trial to guide policy in Uganda. *PLoS One* 2008; **3**(6): e2390.

434. Owusu-Agyei S, Asante KP, Owusu R, et al. An open label, randomised trial of artesunate+amodiaquine, artesunate+chlorproguanil-dapsone and artemether-lumefantrine for the treatment of uncomplicated malaria. *PLoS One* 2008; **3**(6): e2530.

435. Djimde AA, Fofana B, Sagara I, et al. Efficacy, safety, and selection of molecular markers of drug resistance by two ACTs in Mali. *Am J Trop Med Hyg* 2008; **78**(3): 455-61.

436. Sagara I, Diallo A, Kone M, et al. A randomized trial of artesunate-mefloquine versus artemether-lumefantrine for treatment of uncomplicated *Plasmodium falciparum* malaria in Mali. *Am J Trop Med Hyg* 2008; **79**(5): 655-61.

437. Fanello CI, Karema C, Ngamije D, et al. A randomised trial to assess the efficacy and safety of chlorproguanil/dapsone + artesunate for the treatment of uncomplicated *Plasmodium falciparum* malaria. *Trans R Soc Trop Med Hyg* 2008; **102**(5): 412-20.

438. Falade CO, Ogundele AO, Yusuf BO, Ademowo OG, Ladipo SM. High efficacy of two artemisinin-based combinations (artemether-lumefantrine and artesunate plus amodiaquine) for acute uncomplicated malaria in Ibadan, Nigeria. *Trop Med Int Health* 2008; **13**(5): 635-43.

439. Achan J, Tibenderana JK, Kyabayinze D, et al. Effectiveness of quinine versus artemether-lumefantrine for treating uncomplicated falciparum malaria in Ugandan children: randomised trial. *BMJ* 2009; **339**: b2763.

440. Gansane A, Nebie I, Soulama I, et al. [Change of antimalarial first-line treatment in Burkina Faso in 2005]. *Bull Soc Pathol Exot* 2009; **102**(1): 31-5.

441. Sykes A, Hendriksen I, Mtove G, et al. Azithromycin plus artesunate versus artemether-lumefantrine for treatment of uncomplicated malaria in Tanzanian children: a randomized, controlled trial. *Clin Infect Dis* 2009; **49**(8): 1195-201.

442. Arinaitwe E, Sandison TG, Wanzira H, et al. Artemether-lumefantrine versus dihydroartemisinin-piperaquine for falciparum malaria: a longitudinal, randomized trial in young Ugandan children. *Clin Infect Dis* 2009; **49**(11): 1629-37.

443. Sinou V, Malaika LT, Taudon N, et al. Pharmacokinetics and pharmacodynamics of a new ACT formulation: Artesunate/Amodiaquine (TRIMALACT) following oral administration in African malaria patients. *Eur J Drug Metab Pharmacokinet* 2009; **34**(3-4): 133-42.

444. Asante KP, Owusu R, Dosoo D, et al. Adherence to Artesunate-Amodiaquine Therapy for Uncomplicated Malaria in Rural Ghana: A Randomised Trial of Supervised versus Unsupervised Drug Administration. *J Trop Med* 2009; **2009**: 529583.

445. Kayentao K, Maiga H, Newman RD, et al. Artemisinin-based combinations versus amodiaquine plus sulphadoxine-pyrimethamine for the treatment of uncomplicated malaria in Faladje, Mali. *Malar J* 2009; **8**: 5.

446. Tekete M, Djimde AA, Beavogui AH, et al. Efficacy of chloroquine, amodiaquine and sulphadoxine-pyrimethamine for the treatment of uncomplicated falciparum malaria: revisiting molecular markers in an area of emerging AQ and SP resistance in Mali. *Malar J* 2009; **8**: 34.

447. Nahum A, Erhart A, Ahounou D, et al. Extended high efficacy of the combination sulphadoxine-pyrimethamine with artesunate in children with uncomplicated falciparum malaria on the Benin coast, West Africa. *Malar J* 2009; **8**: 37.

448. Sirima SB, Tiono AB, Gansane A, et al. The efficacy and safety of a new fixed-dose combination of amodiaquine and artesunate in young African children with acute uncomplicated *Plasmodium falciparum*. *Malar J* 2009; **8**: 48.

449. Ndiaye JL, Randrianarivelojosia M, Sagara I, et al. Randomized, multicentre assessment of the efficacy and safety of ASAQ--a fixed-dose artesunate-amodiaquine combination therapy in the treatment of uncomplicated *Plasmodium falciparum* malaria. *Malar J* 2009; **8**: 125.

450. Allen EN, Little F, Camba T, et al. Efficacy of sulphadoxine-pyrimethamine with or without artesunate for the treatment of uncomplicated *Plasmodium falciparum* malaria in southern Mozambique: a randomized controlled trial. *Malar J* 2009; **8**: 141.

451. Toure OA, Penali LK, Yapi JD, et al. A comparative, randomized clinical trial of artemisinin/naphtoquine twice daily one day versus artemether/lumefantrine six doses regimen in children and adults with uncomplicated falciparum malaria in Cote d'Ivoire. *Malar J* 2009; **8**: 148.

452. Bonnet M, Broek I, van Herp M, et al. Varying efficacy of artesunate+amodiaquine and artesunate+sulphadoxine-pyrimethamine for the treatment of uncomplicated falciparum malaria in the Democratic Republic of Congo: a report of two *in-vivo* studies. *Malar J* 2009; **8**: 192.

453. Bell DJ, Wootton D, Mukaka M, et al. Measurement of adherence, drug concentrations and the effectiveness of artemether-lumefantrine, chlorproguanil-dapsone or sulphadoxine-pyrimethamine in the treatment of uncomplicated malaria in Malawi. *Malar J* 2009; **8**: 204.

454. Gesase S, Gosling RD, Hashim R, et al. High resistance of *Plasmodium falciparum* to sulphadoxine/pyrimethamine in northern Tanzania and the emergence of dhps resistance mutation at Codon 581. *PLoS One* 2009; **4**(2): e4569.

455. Mutabingwa TK, Muze K, Ord R, et al. Randomized trial of artesunate+amodiaquine, sulfadoxine-pyrimethamine+amodiaquine, chlorproguanal-dapsone and SP for malaria in pregnancy in Tanzania. *PLoS One* 2009; **4**(4): e5138.

456. Premji Z, Umeh RE, Owusu-Agyei S, et al. Chlorproguanil-dapsone-artesunate versus artemether-lumefantrine: a randomized, double-blind phase III trial in African children and adolescents with uncomplicated *Plasmodium falciparum* malaria. *PLoS One* 2009; **4**(8): e6682.

457. Bassat Q, Mulenga M, Tinto H, et al. Dihydroartemisinin-piperaquine and artemether-lumefantrine for treating uncomplicated malaria in African children: a randomised, non-inferiority trial. *PLoS One* 2009; **4**(11): e7871.

458. Tiono AB, Dicko A, Ndububa DA, et al. Chlorproguanil-dapsone-artesunate versus chlorproguanil-dapsone: a randomized, double-blind, phase III trial in African children, adolescents, and adults with uncomplicated *Plasmodium falciparum* malaria. *Am J Trop Med Hyg* 2009; **81**(6): 969-78.

459. Sowunmi A, Gbotosho GO, Happi C, et al. Therapeutic efficacy and effects of artesunate-mefloquine and mefloquine alone on malaria-associated anemia in children with uncomplicated *Plasmodium falciparum* malaria in southwest Nigeria. *Am J Trop Med Hyg* 2009; **81**(6): 979-86.

460. Sasi P, Abdulrahaman A, Mwai L, et al. In vivo and in vitro efficacy of amodiaquine against *Plasmodium falciparum* in an area of continued use of 4-aminoquinolines in East Africa. *J Infect Dis* 2009; **199**(11): 1575-82.

461. Faucher JF, Aubouy A, Adeothy A, et al. Comparison of sulfadoxine-pyrimethamine, unsupervised artemether-lumefantrine, and unsupervised artesunate-amodiaquine fixed-dose formulation for uncomplicated plasmodium falciparum malaria in Benin: a randomized effectiveness noninferiority trial. *J Infect Dis* 2009; **200**(1): 57-65.

462. Moon KT, Kim YK, Ko DH, Park I, Shin DC, Kim C. Recurrence rate of vivax malaria in the Republic of Korea. *Trans R Soc Trop Med Hyg* 2009; **103**(12): 1245-9.

463. Thwing JI, Odero CO, Odhiambo FO, et al. *In-vivo* efficacy of amodiaquine-artesunate in children with uncomplicated *Plasmodium falciparum* malaria in western Kenya. *Trop Med Int Health* 2009; **14**(3): 294-300.

464. Rujumba J, Mworozi EA, Maganda AK, Kiguba R, Rwakimali B, Nsobya S. A comparative study of Arco and Coartem in the treatment of uncomplicated malaria in patients aged 4 months to 16 years attending Mulago hospital, Kampala, Uganda. *Int J Infect Dis* 2010; **14**: e332.

465. Okafor HU, Shu EN, Oguonu T. Therapeutic efficacy and effect on gametocyte carriage of an artemisinin and a non-based combination treatment in children with uncomplicated *P. falciparum* malaria, living in an area with high-level chloroquine resistance. *J Trop Pediatr* 2010; **56**(6): 398-406.

466. Mbacham WF, Evehe MS, Netongo PM, et al. Efficacy of amodiaquine, sulphadoxine-pyrimethamine and their combination for the treatment of uncomplicated *Plasmodium falciparum* malaria in children in Cameroon at the time of policy change to artemisinin-based combination therapy. *Malar J* 2010; **9**: 34.

467. Whegang SY, Tahar R, Foumane VN, et al. Efficacy of non-artemisinin- and artemisinin-based combination therapies for uncomplicated falciparum malaria in Cameroon. *Malar J* 2010; **9**: 56.

468. Michael OS, Gbotosho GO, Folarin OA, et al. Early variations in *Plasmodium falciparum* dynamics in Nigerian children after treatment with two artemisinin-based combinations: implications on delayed parasite clearance. *Malar J* 2010; **9**: 335.

469. Ayede IA, Falade AG, Sowunmi A, Jansen FH. An open randomized clinical trial in comparing two artesunate-based combination treatments on *Plasmodium falciparum* malaria in Nigerian children: artesunate/sulphamethoxypyrazine/pyrimethamine (fixed dose over 24 hours) versus artesunate/amodiaquine (fixed dose over 48 hours). *Malar J* 2010; **9**: 378.

470. Clark TD, Njama-Meya D, Nzarubara B, et al. Incidence of malaria and efficacy of combination antimalarial therapies over 4 years in an urban cohort of Ugandan children. *PLoS One* 2010; **5**(7): e11759.

471. Faye B, Ndiaye JL, Tine R, et al. A randomized trial of artesunate mefloquine versus artemether lumefantrine for the treatment of uncomplicated *Plasmodium falciparum* malaria in Senegalese children. *Am J Trop Med Hyg* 2010; **82**(1): 140-4.

472. Tietche F, Chelo D, Mina Ntoto NK, et al. Tolerability and efficacy of a pediatric granule formulation of artesunate-mefloquine in young children from Cameroon with uncomplicated falciparum malaria. *Am J Trop Med Hyg* 2010; **82**(6): 1034-40.

473. Faye B, Offianan AT, Ndiaye JL, et al. Efficacy and tolerability of artesunate-amodiaquine (Camoquin plus) versus artemether-lumefantrine (Coartem) against uncomplicated *Plasmodium falciparum* malaria: multisite trial in Senegal and Ivory Coast. *Trop Med Int Health* 2010; **15**(5): 608-13.

474. Bouyou-Akotet MK, Ramharter M, Ngoungou EB, et al. Efficacy and safety of a new pediatric artesunate-mefloquine drug formulation for the treatment of uncomplicated falciparum malaria in Gabon. *Wien Klin Wochenschr* 2010; **122**(5-6): 173-8.

475. Ngasala BE, Malmberg M, Carlsson AM, et al. Efficacy and effectiveness of artemether-lumefantrine after initial and repeated treatment in children <5 years of age with acute uncomplicated *Plasmodium falciparum* malaria in rural Tanzania: a randomized trial. *Clin Infect Dis* 2011; **52**(7): 873-82.

476. Sowunmi A, Akinrinola IA, Gbotosho GO, Okuboyejo TM, Happi CT. A simple dose regimen of artesunate and amodiaquine based on arm span- or age range for childhood falciparum malaria: a preliminary evaluation. *J Trop Pediatr* 2012; **58**(4): 263-8.

477. Rasheed A, Khan SM, Awan MY, Khurshid U, Ahmad K. Efficacy and safety of artemehter-lumefantrine in uncomplicated falciparum malaria in Liberia. *J Pak Med Assoc* 2011; **61**(2): 131-4.

478. Nambozi M, Van Geertruyden JP, Hachizovu S, et al. Safety and efficacy of dihydroartemisinin-piperaquine versus artemether-lumefantrine in the treatment of uncomplicated *Plasmodium falciparum* malaria in Zambian children. *Malar J* 2011; **10**: 50.

479. Ngasala BE, Malmberg M, Carlsson AM, et al. Effectiveness of artemether-lumefantrine provided by community health workers in under-five children with uncomplicated malaria in rural Tanzania: an open label prospective study. *Malar J* 2011; **10**: 64.

480. Menan H, Faye O, Same-Ekobo A, et al. Comparative study of the efficacy and tolerability of dihydroartemisinin-piperaquine-trimethoprim versus artemether-lumefantrine in the treatment of uncomplicated *Plasmodium falciparum* malaria in Cameroon, Ivory Coast and Senegal. *Malar J* 2011; **10**: 185.

481. Yavo W, Faye B, Kuete T, et al. Multicentric assessment of the efficacy and tolerability of dihydroartemisinin-piperaquine compared to artemether-lumefantrine in the treatment of uncomplicated *Plasmodium falciparum* malaria in sub-Saharan Africa. *Malar J* 2011; **10**: 198.

482. Four Artemisinin-Based Combinations Study G. A head-to-head comparison of four artemisinin-based combinations for treating uncomplicated malaria in African children: a randomized trial. *PLoS Med* 2011; **8**(11): e1001119.

483. Dunyo S, Sirugo G, Sesay S, et al. Randomized trial of safety and effectiveness of chlorproguanil-dapsone and lumefantrine-artemether for uncomplicated malaria in children in the Gambia. *PLoS One* 2011; **6**(6): e17371.

484. Borrmann S, Sasi P, Mwai L, et al. Declining responsiveness of *Plasmodium falciparum* infections to artemisinin-based combination treatments on the Kenyan coast. *PLoS One* 2011; **6**(11): e26005.

485. Gbotosho GO, Sowunmi A, Okuboyejo TM, et al. Therapeutic efficacy and effects of artemether-lumefantrine and artesunate-amodiaquine coformulated or copackaged on malaria-associated anemia in children with uncomplicated *Plasmodium falciparum* malaria in Southwest Nigeria. *Am J Trop Med Hyg* 2011; **84**(5): 813-9.

486. Gbotosho GO, Sowunmi A, Happi CT, Okuboyejo TM. Therapeutic efficacies of artemisinin-based combination therapies in Nigerian children with uncomplicated falciparum malaria during five years of adoption as first-line treatments. *Am J Trop Med Hyg* 2011; **84**(6): 936-43.

487. Ursing J, Kofoed PE, Rodrigues A, et al. Similar efficacy and tolerability of double-dose chloroquine and artemether-lumefantrine for treatment of *Plasmodium falciparum* infection in Guinea-Bissau: a randomized trial. *J Infect Dis* 2011; **203**(1): 109-16.

488. Toure OA, Kouame MG, Didier YJ, et al. Artesunate/mefloquine paediatric formulation vs. artemether/lumefantrine for the treatment of uncomplicated *Plasmodium falciparum* in Anonkoua koute, Cote d'Ivoire. *Trop Med Int Health* 2011; **16**(3): 290-7.

489. Lanaspa M, Moraleda C, Machevo S, et al. Inadequate efficacy of a new formulation of fosmidomycin-clindamycin combination in Mozambican children less than three years old with uncomplicated *Plasmodium falciparum* malaria. *Antimicrob Agents Chemother* 2012; **56**(6): 2923-8.

490. Siribie M, Diarra A, Tiono AB, Soulama I, Sirima SB. [Efficacy of artemether-lumefantrine in the treatment of uncomplicated malaria in children living in a rural area of Burkina Faso in 2009]. *Bull Soc Pathol Exot* 2012; **105**(3): 202-7.

491. Kinde-Gazard D, Ogouyemi-Hounto A, Capo-Chichi L, Gbaguidi J, Massougbodji A. [A randomized clinical trial comparing the effectiveness and tolerability of artemisinine-naphthoquine (Arco(R)) and artemether-lumefantrine (Coartem(R)) in the treatment of uncomplicated malaria in Benin]. *Bull Soc Pathol Exot* 2012; **105**(3): 208-14.

492. Gbotosho GO, Sowunmi A, Okuboyejo TM, Happi CT. Oral artesunate-amodiaquine and artemether-lumefantrine in the treatment of uncomplicated hyperparasitaemic *Plasmodium falciparum* malaria in children. *J Trop Pediatr* 2012; **58**(2): 151-3.

493. Kamugisha E, Jing S, Minde M, et al. Efficacy of artemether-lumefantrine in treatment of malaria among under-fives and prevalence of drug resistance markers in Igombe-Mwanza, north-western Tanzania. *Malar J* 2012; **11**: 58.

494. Mombo-Ngoma G, Kleine C, Basra A, et al. Prospective evaluation of artemether-lumefantrine for the treatment of non-falciparum and mixed-species malaria in Gabon. *Malar J* 2012; **11**: 120.

495. Espie E, Lima A, Atua B, et al. Efficacy of fixed-dose combination artesunate-amodiaquine versus artemether-lumefantrine for uncomplicated childhood *Plasmodium falciparum* malaria in Democratic Republic of Congo: a randomized non-inferiority trial. *Malar J* 2012; **11**: 174.

496. Shaukat AM, Gilliams EA, Kenefic LJ, et al. Clinical manifestations of new versus recrudescent malaria infections following anti-malarial drug treatment. *Malar J* 2012; **11**: 207.

497. Dorkenoo MA, Barrette A, Agbo YM, et al. Surveillance of the efficacy of artemether-lumefantrine and artesunate-amodiaquine for the treatment of uncomplicated *Plasmodium falciparum* among children under five in Togo, 2005-2009. *Malar J* 2012; **11**: 338.

498. Abuaku B, Duah N, Quaye L, Quashie N, Koram K. Therapeutic efficacy of artemether-lumefantrine combination in the treatment of uncomplicated malaria among children under five years of age in three ecological zones in Ghana. *Malar J* 2012; **11**: 388.

499. Faye B, Kuete T, Kiki-Barro CP, et al. Multicentre study evaluating the non-inferiority of the new paediatric formulation of artesunate/amodiaquine versus artemether/lumefantrine for the management of uncomplicated *Plasmodium falciparum* malaria in children in Cameroon, Ivory Coast and Senegal. *Malar J* 2012; **11**: 433.

500. Vaughan-Williams CH, Raman J, Raswiswi E, et al. Assessment of the therapeutic efficacy of artemether-lumefantrine in the treatment of uncomplicated *Plasmodium falciparum* malaria in northern KwaZulu-Natal: an observational cohort study. *Malar J* 2012; **11**: 434.

501. Laufer MK, Thesing PC, Dzinjalamala FK, et al. A longitudinal trial comparing chloroquine as monotherapy or in combination with artesunate, azithromycin or atovaquone-proguanil to treat malaria. *PLoS One* 2012; **7**(8): e42284.

502. Maiga AW, Fofana B, Sagara I, et al. No evidence of delayed parasite clearance after oral artesunate treatment of uncomplicated falciparum malaria in Mali. *Am J Trop Med Hyg* 2012; **87**(1): 23-8.

503. Sagara I, Fofana B, Gaudart J, et al. Repeated artemisinin-based combination therapies in a malaria hyperendemic area of Mali: efficacy, safety, and public health impact. *Am J Trop Med Hyg* 2012; **87**(1): 50-6.

504. Sowunmi A, Gbotosho GO, Happi CT, et al. Therapeutic efficacy of artesunate-amodiaquine combinations and the plasma and saliva concentrations of desethylamodiaquine in children with acute uncomplicated *Plasmodium falciparum* malaria. *Am J Ther* 2013; **20**(1): 48-56.

505. Chijioke-Nwauche I, van Wyk A, Nwauche C, Beshir KB, Kaur H, Sutherland CJ. HIV-positive nigerian adults harbor significantly higher serum lumefantrine levels than HIV-negative individuals seven days after treatment for *Plasmodium falciparum* infection. *Antimicrob Agents Chemother* 2013; **57**(9): 4146-50.

506. Sylla K, Abiola A, Tine RC, et al. Monitoring the efficacy and safety of three artemisinin based-combinations therapies in Senegal: results from two years surveillance. *BMC Infect Dis* 2013; **13**: 598.

507. Ojurongbe O, Lawal OA, Abiodun OO, Okeniyi JA, Oyeniyi AJ, Oyelami OA. Efficacy of artemisinin combination therapy for the treatment of uncomplicated falciparum malaria in Nigerian children. *J Infect Dev Ctries* 2013; **7**(12): 975-82.

508. Eziefula AC, Bousema T, Yeung S, et al. Single dose primaquine for clearance of *Plasmodium falciparum* gametocytes in children with uncomplicated malaria in Uganda: a randomised, controlled, double-blind, dose-ranging trial. *Lancet Infect Dis* 2014; **14**(2): 130-9.

509. Ali IM, Netongo PM, Atogho-Tiedeu B, et al. Amodiaquine-Artesunate versus Artemether-Lumefantrine against Uncomplicated Malaria in Children Less Than 14 Years in Ngaoundere, North Cameroon: Efficacy, Safety, and Baseline Drug Resistant Mutations in *pfcrt*, *pfmdr1*, and *pfdhfr* Genes. *Malar Res Treat* 2013; **2013**: 234683.

510. Ndounga M, Mayengue PI, Casimiro PN, et al. Artesunate-amodiaquine efficacy in Congolese children with acute uncomplicated falciparum malaria in Brazzaville. *Malar J* 2013; **12**: 53.

511. Schramm B, Valeh P, Baudin E, et al. Tolerability and safety of artesunate-amodiaquine and artemether-lumefantrine fixed dose combinations for the treatment of uncomplicated *Plasmodium falciparum* malaria: two open-label, randomized trials in Nimba County, Liberia. *Malar J* 2013; **12**: 250.

512. Schramm B, Valeh P, Baudin E, et al. Efficacy of artesunate-amodiaquine and artemether-lumefantrine fixed-dose combinations for the treatment of uncomplicated *Plasmodium falciparum* malaria among children aged six to 59 months in Nimba County, Liberia: an open-label randomized non-inferiority trial. *Malar J* 2013; **12**: 251.

513. Agarwal A, McMorrow M, Onyango P, et al. A randomized trial of artemether-lumefantrine and dihydroartemisinin-piperaquine in the treatment of uncomplicated malaria among children in western Kenya. *Malar J* 2013; **12**: 254.

514. Nambei WS, Lango Yaya E, Pounguinza S, et al. [Efficacy and safety of antimalarial combinations for treatment of uncomplicated malaria in children in Bangui, Central African Republic]. *Med Sante Trop* 2013; **23**(3): 313-9.

515. Yeka A, Tibenderana J, Achan J, D'Alessandro U, Talisuna AO. Efficacy of quinine, artemether-lumefantrine and dihydroartemisinin-piperaquine as rescue treatment for uncomplicated malaria in Ugandan children. *PLoS One* 2013; **8**(1): e53772.

516. Sahr F, Smith SJ, Kamara A, Warsame M, Sillah J, Swarray A. Assessment of the therapeutic efficacy of two artemisinin-based combinations in the treatment of uncomplicated falciparum malaria amonf children under 5 years in four district hospital in Sierra Leone. *Sierra Leone J Biomed Res* 2013; **5**(1): 4-8.

517. Kakuru A, Jagannathan P, Arinaitwe E, et al. The effects of ACT treatment and TS prophylaxis on *Plasmodium falciparum* gametocytemia in a cohort of young Ugandan children. *Am J Trop Med Hyg* 2013; **88**(4): 736-43.

518. Charle P, Berzosa P, de Lucio A, Raso J, Nseng Nchama G, Benito A. Artesunate/amodiaquine malaria treatment for Equatorial Guinea (Central Africa). *Am J Trop Med Hyg* 2013; **88**(6): 1087-92.

519. Creek DJ, Bigira V, McCormack S, et al. Pharmacokinetic predictors for recurrent malaria after dihydroartemisinin-piperaquine treatment of uncomplicated malaria in Ugandan infants. *J Infect Dis* 2013; **207**(11): 1646-54.

520. Sawa P, Shekalaghe SA, Drakeley CJ, et al. Malaria transmission after artemether-lumefantrine and dihydroartemisinin-piperaquine: a randomized trial. *J Infect Dis* 2013; **207**(11): 1637-45.

521. Okonta MJ, Nduka SO, Ilodigwe EE, Anetoh MU. Comparative assessment of two artemisinin based combination therapies in the treatment of uncomplicated malaria among University students in Nigeria. *Int J Drug Dev Res* 2013; **5**(2): 211-21.

522. Oguche S, Okafor HU, Watila I, et al. Efficacy of artemisinin-based combination treatments of uncomplicated falciparum malaria in under-five-year-old Nigerian children. *Am J Trop Med Hyg* 2014; **91**(5): 925-35.

523. Mosha D, Guidi M, Mwingira F, et al. Population pharmacokinetics and clinical response for artemether-lumefantrine in pregnant and nonpregnant women with uncomplicated *Plasmodium falciparum* malaria in Tanzania. *Antimicrob Agents Chemother* 2014; **58**(8): 4583-92.

524. Onyamboko MA, Fanello CI, Wongsaen K, et al. Randomized comparison of the efficacies and tolerabilities of three artemisinin-based combination treatments for children with acute *Plasmodium falciparum* malaria in the Democratic Republic of the Congo. *Antimicrob Agents Chemother* 2014; **58**(9): 5528-36.

525. Kakuru A, Achan J, Muhindo MK, et al. Artemisinin-based combination therapies are efficacious and safe for treatment of uncomplicated malaria in HIV-infected Ugandan children. *Clin Infect Dis* 2014; **59**(3): 446-53.

526. Valea I, Tinto H, Traore-Coulibaly M, et al. Pharmacokinetics of co-formulated mefloquine and artesunate in pregnant and non-pregnant women with uncomplicated *Plasmodium falciparum* infection in Burkina Faso. *J Antimicrob Chemother* 2014; **69**(9): 2499-507.

527. Tahar R, Almelli T, Debue C, et al. Randomized trial of artesunate-amodiaquine, atovaquone-proguanil, and artesunate-atovaquone-proguanil for the treatment of uncomplicated falciparum malaria in children. *J Infect Dis* 2014; **210**(12): 1962-71.

528. Coulibaly B, Pritsch M, Bountogo M, et al. Efficacy and safety of triple combination therapy with artesunate-amodiaquine-methylene blue for falciparum malaria in children: a randomized controlled trial in Burkina Faso. *J Infect Dis* 2015; **211**(5): 689-97.

529. Djalle D, Njuimo SP, Manirakiza A, Laganier R, Le Faou A, Rogier C. Efficacy and safety of artemether + lumefantrine, artesunate + sulphamethoxypyrazine-pyrimethamine and artesunate + amodiaquine and sulphadoxine-pyrimethamine + amodiaquine in the treatment of uncomplicated falciparum malaria in Bangui, Central African Republic: a randomized trial. *Malar J* 2014; **13**: 9.

530. Muhindo MK, Kakuru A, Jagannathan P, et al. Early parasite clearance following artemisinin-based combination therapy among Ugandan children with uncomplicated *Plasmodium falciparum* malaria. *Malar J* 2014; **13**: 32.

531. Ogutu BR, Onyango KO, Koskei N, et al. Efficacy and safety of artemether-lumefantrine and dihydroartemisinin-piperaquine in the treatment of uncomplicated *Plasmodium falciparum* malaria in Kenyan children aged less than five years: results of an open-label, randomized, single-centre study. *Malar J* 2014; **13**: 33.

532. Coulibaly SO, Kayentao K, Taylor S, et al. Parasite clearance following treatment with sulphadoxine-pyrimethamine for intermittent preventive treatment in Burkina-Faso and Mali: 42-day in vivo follow-up study. *Malar J* 2014; **13**: 41.

533. Maganda BA, Minzi OM, Kamuhabwa AA, Ngasala B, Sasi PG. Outcome of artemether-lumefantrine treatment for uncomplicated malaria in HIV-infected adult patients on anti-retroviral therapy. *Malar J* 2014; **13**: 205.

534. Nhama A, Bassat Q, Enosse S, et al. *In vivo* efficacy of artemether-lumefantrine and artesunate-amodiaquine for the treatment of uncomplicated falciparum malaria in children: a multisite, open-label, two-cohort, clinical trial in Mozambique. *Malar J* 2014; **13**: 309.

535. Adjei GO, Goka BQ, Enweronu-Laryea CC, et al. A randomized trial of artesunate-amodiaquine versus artemether-lumefantrine in Ghanaian paediatric sickle cell and non-sickle cell disease patients with acute uncomplicated malaria. *Malar J* 2014; **13**: 369.

536. Shayo A, Mandara CI, Shahada F, Buza J, Lemnge MM, Ishengoma DS. Therapeutic efficacy and safety of artemether-lumefantrine for the treatment of uncomplicated falciparum malaria in North-Eastern Tanzania. *Malar J* 2014; **13**: 376.

537. Hamainza B, Masaninga F, Moonga H, et al. Therapeutic efficacy of artemether-lumefantrine on treatment of uncomplicated *Plasmodium falciparum* mono-infection in an area of high malaria transmission in Zambia. *Malar J* 2014; **13**: 430.

538. Toure OA, Assi SB, N'Guessan TL, et al. Open-label, randomized, non-inferiority clinical trial of artesunate-amodiaquine versus artemether-lumefantrine fixed-dose combinations in children and adults with uncomplicated falciparum malaria in Cote d'Ivoire. *Malar J* 2014; **13**: 439.

539. Sagara I, Oduro AR, Mulenga M, et al. Efficacy and safety of a combination of azithromycin and chloroquine for the treatment of uncomplicated *Plasmodium falciparum* malaria in two multi-country randomised clinical trials in African adults. *Malar J* 2014; **13**: 458.

540. Ouldabdallahi M, Alew I, Salem MS, et al. Efficacy of artesunate-amodiaquine for the treatment of acute uncomplicated falciparum malaria in southern Mauritania. *Malar J* 2014; **13**: 496.

541. Ogutu B, Juma E, Obonyo C, et al. Fixed dose artesunate amodiaquine - a phase IIb, randomized comparative trial with non-fixed artesunate amodiaquine. *Malar J* 2014; **13**: 498.

542. Falade CO, Dada-Adegbola HO, Ogunkunle OO, Oguike MC, Nash O, Ademowo OG. Evaluation of the comparative efficacy and safety of artemether-lumefantrine, artesunate-amodiaquine and artesunate-amodiaquine-chlorpheniramine (Artemoclo) for the treatment of acute uncomplicated malaria in Nigerian children. *Med Princ Pract* 2014; **23**(3): 204-11.

543. Zongo I, Some FA, Somda SA, et al. Efficacy and day 7 plasma piperaquine concentrations in African children treated for uncomplicated malaria with dihydroartemisinin-piperaquine. *PLoS One* 2014; **9**(8): e103200.

544. Yeka A, Lameyre V, Afizi K, et al. Efficacy and safety of fixed-dose artesunate-amodiaquine vs. artemether-lumefantrine for repeated treatment of uncomplicated malaria in Ugandan children. *PLoS One* 2014; **9**(12): e113311.

545. Tinto H, Diallo S, Zongo I, et al. Effectiveness of artesunate-amodiaquine vs. artemether-lumefantrine for the treatment of uncomplicated falciparum malaria in Nanoro, Burkina Faso: a non-inferiority randomised trial. *Trop Med Int Health* 2014; **19**(4): 469-75.

546. Okech BA, Existe A, Romain JR, et al. Therapeutic efficacy of chloroquine for the treatment of uncomplicated *Plasmodium falciparum* in Haiti after many decades of its use. *Am J Trop Med Hyg* 2015; **92**(3): 541-5.

547. Plucinski MM, Talundzic E, Morton L, et al. Efficacy of artemether-lumefantrine and dihydroartemisinin-piperaquine for treatment of uncomplicated malaria in children in Zaire and Uige Provinces, angola. *Antimicrob Agents Chemother* 2015; **59**(1): 437-43.

548. Siribie M, Diarra A, Tiono AB, Soulama I, Sirima SB. [Effect of a large scale community-based distribution of artemether-lumefantrine on its therapeutic efficacy among children living in a rural area of Burkina Faso]. *Bull Soc Pathol Exot* 2015; **108**(2): 120-3.

549. Ukah M, Badejoko O, Ogunniyi S, Loto O, Aboderin O, Fatusi A. A randomized trial of artesunate-amodiaquine versus artemether-lumefantrine for the treatment of acute uncomplicated malaria in pregnancy. *Int J Gynaecol Obstet* 2015; **131**(1): 41-4.

550. Held J, Supan C, Salazar CL, et al. Ferroquine and artesunate in African adults and children with *Plasmodium falciparum* malaria: a phase 2, multicentre, randomised, double-blind, dose-ranging, non-inferiority study. *Lancet Infect Dis* 2015; **15**(12): 1409-19.

551. Gutman J, Mwandama D, Wiegand RE, et al. *In vivo* efficacy of sulphadoxine-pyrimethamine for the treatment of asymptomatic parasitaemia in pregnant women in Machinga District, Malawi. *Malar J* 2015; **14**: 197.

552. Sondo P, Derra K, Diallo-Nakanabo S, et al. Effectiveness and safety of artemether-lumefantrine versus artesunate-amodiaquine for unsupervised treatment of uncomplicated falciparum malaria in patients of all age groups in Nanoro, Burkina Faso: a randomized open label trial. *Malar J* 2015; **14**: 325.

553. Ndounga M, Pembe Issamou M, Casimiro PN, et al. Artesunate-amodiaquine versus artemether-lumefantrine for the treatment of acute uncomplicated malaria in Congolese children under 10 years old living in a suburban area: a randomized study. *Malar J* 2015; **14**: 423.

554. Kiaco K, Teixeira J, Machado M, do Rosario V, Lopes D. Evaluation of artemether-lumefantrine efficacy in the treatment of uncomplicated malaria and its association with *pfmdr1*, *pfatpase6* and K13-propeller polymorphisms in Luanda, Angola. *Malar J* 2015; **14**: 504.

555. Yavo W, Konate A, Kassi FK, et al. Efficacy and Safety of Artesunate-Amodiaquine versus Artemether-Lumefantrine in the Treatment of Uncomplicated *Plasmodium falciparum* Malaria in Sentinel Sites across Cote d'Ivoire. *Malar Res Treat* 2015; **2015**: 878132.

556. Nji AM, Ali IM, Moyeh MN, et al. Randomized non-inferiority and safety trial of dihydroartemisin-piperaquine and artesunate-amodiaquine versus artemether-lumefantrine in the treatment of uncomplicated *Plasmodium falciparum* malaria in Cameroonian children. *Malar J* 2015; **14**: 27.

557. Kapisi J, Bigira V, Clark T, et al. Efficacy and safety of artemether-lumefantrine for the treatment of uncomplicated malaria in the setting of three different chemopreventive regimens. *Malar J* 2015; **14**: 53.

558. Chandra R, Ansah P, Sagara I, et al. Comparison of azithromycin plus chloroquine versus artemether-lumefantrine for the treatment of uncomplicated *Plasmodium falciparum* malaria in children in Africa: a randomized, open-label study. *Malar J* 2015; **14**: 108.

559. Tiono AB, Tinto H, Alao MJ, et al. Increased systemic exposures of artemether and dihydroartemisinin in infants under 5 kg with uncomplicated *Plasmodium falciparum* malaria treated with artemether-lumefantrine (Coartem(R)). *Malar J* 2015; **14**: 157.

560. Maiga H, Djimde AA, Beavogui AH, et al. Efficacy of sulphadoxine-pyrimethamine + artesunate, sulphadoxine-pyrimethamine + amodiaquine, and sulphadoxine-pyrimethamine alone in uncomplicated falciparum malaria in Mali. *Malar J* 2015; **14**: 64.

561. Dambe R, Sande J, Ali D, et al. Monitoring the efficacy of artemether-lumefantrine for the treatment of uncomplicated malaria in Malawian children. *Malar J* 2015; **14**: 175.

562. Sow D, Ndiaye JL, Sylla K, et al. Evaluation of the efficacy and safety of three 2-drug combinations for the treatment of uncomplicated *Plasmodium falciparum* malaria in Senegal: artesunate-amodiaquine, dihydroartemisinin-piperaquine, and artemether-lumefantrine. *Med Sante Trop* 2015.

563. Niare K, Dara A, Sagara I, et al. *In Vivo* Efficacy and Parasite Clearance of Artesunate + Sulfadoxine-Pyrimethamine Versus Artemether-Lumefantrine in Mali. *Am J Trop Med Hyg* 2016; **94**(3): 634-9.

564. Dieye B, Affara M, Sangare L, et al. West Africa International Centers of Excellence for Malaria Research: Drug Resistance Patterns to Artemether-Lumefantrine in Senegal, Mali, and The Gambia. *Am J Trop Med Hyg* 2016; **95**(5): 1054-60.

565. Nyunt MM, Nguyen VK, Kajubi R, et al. Artemether-Lumefantrine Pharmacokinetics and Clinical Response is Minimally Altered in Ugandan Pregnant Women Treated for Uncomplicated *Plasmodium Falciparum* Malaria. *Antimicrob Agents Chemother* 2016; **60**(3): 1274-82.

566. Sowunmi A, Akano K, Ayede AI, et al. Clinical illness and outcomes in Nigerian children with late-appearing anaemia after artemisinin-based combination treatments of uncomplicated falciparum malaria. *BMC Infect Dis* 2016; **16**: 240.

567. Goncalves BP, Tiono AB, Ouedraogo A, et al. Single low dose primaquine to reduce gametocyte carriage and *Plasmodium falciparum* transmission after artemether-lumefantrine in children with asymptomatic infection: a randomised, double-blind, placebo-controlled trial. *BMC Med* 2016; **14**: 40.

568. Yeka A, Kigozi R, Conrad MD, et al. Artesunate/Amodiaquine Versus Artemether/Lumefantrine for the Treatment of Uncomplicated Malaria in Uganda: A Randomized Trial. *J Infect Dis* 2016; **213**(7): 1134-42.

569. Sagara I, Beavogui AH, Zongo I, et al. Safety and efficacy of re-treatments with pyronaridine-artesunate in African patients with malaria: a substudy of the WANECAM randomised trial. *Lancet Infect Dis* 2016; **16**(2): 189-98.

570. Dicko A, Brown JM, Diawara H, et al. Primaquine to reduce transmission of *Plasmodium falciparum* malaria in Mali: a single-blind, dose-ranging, adaptive randomised phase 2 trial. *Lancet Infect Dis* 2016; **16**(6): 674-84.

571. Sirima SB, Ogutu B, Lusingu JPA, et al. Comparison of artesunate-mefloquine and artemether-lumefantrine fixed-dose combinations for treatment of uncomplicated *Plasmodium falciparum* malaria in children younger than 5 years in sub-Saharan Africa: a randomised, multicentre, phase 4 trial. *Lancet Infect Dis* 2016; **16**(10): 1123-33.

572. Abuaku B, Duah N, Quaye L, et al. Therapeutic efficacy of artesunate-amodiaquine and artemether-lumefantrine combinations in the treatment of uncomplicated malaria in two ecological zones in Ghana. *Malar J* 2016; **15**: 6.

573. Singana BP, Bogreau H, Matondo BD, et al. Malaria burden and anti-malarial drug efficacy in Owando, northern Congo. *Malar J* 2016; **15**: 16.

574. Ogouyemi-Hounto A, Azandossessi C, Lawani S, et al. Therapeutic efficacy of artemether-lumefantrine for the treatment of uncomplicated falciparum malaria in northwest Benin. *Malar J* 2016; **15**: 37.

575. Dorkenoo AM, Yehadji D, Agbo YM, et al. Therapeutic efficacy trial of artemisinin-based combination therapy for the treatment of uncomplicated malaria and investigation of mutations in k13 propeller domain in Togo, 2012-2013. *Malar J* 2016; **15**: 331.

576. de Wit M, Funk AL, Moussally K, et al. In vivo efficacy of artesunate-amodiaquine and artemether-lumefantrine for the treatment of uncomplicated falciparum malaria: an open-randomized, non-inferiority clinical trial in South Kivu, Democratic Republic of Congo. *Malar J* 2016; **15**: 455.

577. Paczkowski M, Mwandama D, Marthey D, et al. *In vivo* efficacy of artemether-lumefantrine and artesunate-amodiaquine for uncomplicated *Plasmodium falciparum* malaria in Malawi, 2014. *Malar J* 2016; **15**: 236.

578. Mutagonda RF, Kamuhabwa AA, Minzi OM, Massawe SN, Maganda BA, Aklillu E. Malaria prevalence, severity and treatment outcome in relation to day 7 lumefantrine plasma concentration in pregnant women. *Malar J* 2016; **15**(1): 278.

579. Mwaiswelo R, Ngasala B, Jovel I, et al. Adding a single low-dose of primaquine (0.25 mg/kg) to artemether-lumefantrine did not compromise treatment outcome of uncomplicated *Plasmodium falciparum* malaria in Tanzania: a randomized, single-blinded clinical trial. *Malar J* 2016; **15**(1): 435.

580. Muhindo Mavoko H, Kalabuanga M, Delgado-Ratto C, et al. Uncomplicated Clinical Malaria Features, the Efficacy of Artesunate-Amodiaquine and Their Relation with Multiplicity of Infection in the Democratic Republic of Congo. *PLoS One* 2016; **11**(6): e0157074.

581. Ursing J, Rombo L, Rodrigues A, Kofoed PE. Artemether-Lumefantrine versus Dihydroartemisinin-Piperaquine for Treatment of Uncomplicated *Plasmodium falciparum* Malaria in Children Aged Less than 15 Years in Guinea-Bissau - An Open-Label Non-Inferiority Randomised Clinical Trial. *PLoS One* 2016; **11**(9): e0161495.

582. Pregact Study Group, Pekyi D, Ampromfi AA, et al. Four Artemisinin-Based Treatments in African Pregnant Women with Malaria. *N Engl J Med* 2016; **374**(10): 913-27.

583. Denoeud-Ndam L, Dicko A, Baudin E, et al. Efficacy of artemether-lumefantrine in relation to drug exposure in children with and without severe acute malnutrition: an open comparative intervention study in Mali and Niger. *BMC Med* 2016; **14**(1): 167.

584. Okebe J, Bousema T, Affara M, et al. The Gametocytocidal Efficacy of Different Single Doses of Primaquine with Dihydroartemisinin-piperaquine in Asymptomatic Parasite Carriers in The Gambia: A Randomized Controlled Trial. *EBioMedicine* 2016; **13**: 348-55.

585. Apinjoh TO, Anchang-Kimbi JK, Ajonina MU, et al. *In Vivo* Efficacy of Artesunate/Sulphadoxine-Pyrimethamine versus Artesunate/Amodiaquine in the Treatment of Uncomplicated *P. falciparium* Malaria in Children around the Slope of Mount Cameroon: A Randomized Controlled Trial. *Biomedicines* 2016; **4**(1).

586. Grandesso F, Nabasumba C, Nyehangane D, et al. Performance and time to become negative after treatment of three malaria rapid diagnostic tests in low and high malaria transmission settings. *Malar J* 2016; **15**(1): 496.

587. Mavoko HM, Nabasumba C, da Luz RI, et al. Efficacy and safety of re-treatment with the same artemisinin-based combination treatment (ACT) compared with an alternative ACT and quinine plus clindamycin after failure of first-line recommended ACT (QUINACT): a bicentre, open-label, phase 3, randomised controlled trial. *Lancet Glob Health* 2017; **5**(1): e60-e8.

588. Gargano N, Madrid L, Valentini G, et al. Efficacy and Tolerability Outcomes of a Phase II, Randomized, Open-Label, Multicenter Study of a New Water-Dispersible Pediatric Formulation of Dihydroartemisinin-Piperaquine for the Treatment of Uncomplicated *Plasmodium falciparum* Malaria in African Infants. *Antimicrob Agents Chemother* 2018; **62**(1).

589. Mombo-Ngoma G, Remppis J, Sievers M, et al. Efficacy and safety of fosmidomycin-piperaquine as non-artemisinin-based combination therapy for uncomplicated falciparum malaria - A single-arm, age-de-escalation proof of concept study in Gabon. *Clin Infect Dis* 2017.

590. Galatas B, Nhamussua L, Candrinho B, et al. *In-Vivo* Efficacy of Chloroquine to Clear Asymptomatic Infections in Mozambican Adults: A Randomized, Placebo-controlled Trial with Implications for Elimination Strategies. *Sci Rep* 2017; **7**(1): 1356.

591. Held J, Supan C, Salazar CLO, et al. Safety and efficacy of the choline analogue SAR97276 for malaria treatment: results of two phase 2, open-label, multicenter trials in African patients. *Malar J* 2017; **16**(1): 188.

592. Iribhogbe OI, Emmanuel I, Odianosen M. Comparative analysis of the safety and tolerability of fixed-dose artesunate/amodiaquine versus artemether/lumefantrine combinations for uncomplicated falciparum malaria in pregnancy: a randomized open label study. *Clin Pharmacol* 2017; **9**: 45-54.

593. Nambozi M, Kabuya JB, Hachizovu S, et al. Artemisinin-based combination therapy in pregnant women in Zambia: efficacy, safety and risk of recurrent malaria. *Malar J* 2017; **16**(1): 199.

594. Plucinski MM, Dimbu PR, Macaia AP, et al. Efficacy of artemether-lumefantrine, artesunate-amodiaquine, and dihydroartemisinin-piperaquine for treatment of uncomplicated *Plasmodium falciparum* malaria in Angola, 2015. *Malar J* 2017; **16**(1): 62.

595. Sowunmi A, Akano K, Ntadom G, et al. Therapeutic efficacy and effects of artemisinin-based combination treatments on uncomplicated *Plasmodium falciparum* malaria -associated anaemia in Nigerian children during seven years of adoption as first-line treatments. *Infect Dis Poverty* 2017; **6**(1): 36.

596. Talisuna AO, Oburu A, Githinji S, et al. Efficacy of text-message reminders on paediatric malaria treatment adherence and their post-treatment return to health facilities in Kenya: a randomized controlled trial. *Malar J* 2017; **16**(1): 46.

597. Tine RC, Sylla K, Faye BT, et al. Safety and Efficacy of Adding a Single Low Dose of Primaquine to the Treatment of Adult Patients With *Plasmodium falciparum* Malaria in Senegal, to Reduce Gametocyte Carriage: A Randomized Controlled Trial. *Clin Infect Dis* 2017; **65**(4): 535-43.

598. Koita OA, Sangare L, Miller HD, et al. AQ-13, an investigational antimalarial, versus artemether plus lumefantrine for the treatment of uncomplicated *Plasmodium falciparum* malaria: a randomised, phase 2, non-inferiority clinical trial. *Lancet Infect Dis* 2017; **17**(12): 1266-75.

599. Supan C, Mombo-Ngoma G, Kombila M, et al. Phase 2a, Open-Label, 4-Escalating-Dose, Randomized Multicenter Study Evaluating the Safety and Activity of Ferroquine (SSR97193) Plus Artesunate, versus Amodiaquine Plus Artesunate, in African Adult Men with Uncomplicated *Plasmodium falciparum* Malaria. *Am J Trop Med Hyg* 2017; **97**(2): 514-25.

600. Osarfo J, Tagbor H, Cairns M, Alifrangis M, Magnussen P. Dihydroartemisinin-piperaquine versus artesunate-amodiaquine for treatment of malaria infection in pregnancy in Ghana: an open-label, randomised, non-inferiority trial. *Trop Med Int Health* 2017; **22**(8): 1043-52.

601. Salvador C, Rafael B, Matsinhe F, et al. Efficacy and safety of artemether-lumefantrine for the treatment of uncomplicated falciparum malaria at sentinel sites in Mozambique, 2015. *Acta Trop* 2017; **171**: 146-50.

602. McCarthy JS, Lotharius J, Ruckle T, et al. Safety, tolerability, pharmacokinetics, and activity of the novel long-acting antimalarial DSM265: a two-part first-in-human phase 1a/1b randomised study. *Lancet Infect Dis* 2017; **17**(6): 626-35.

603. Gbotosho GO, Sowunmi A, Okuboyejo TM, Happi CT, Folarin OO, Adewoye EO. A simple dose regimen of artesunate and amodiaquine based on age or body weight range for uncomplicated falciparum malaria in children: comparison of therapeutic efficacy with standard dose regimen of artesunate and amodiaquine and artemether-lumefantrine. *Am J Ther* 2012; **19**(4): e122-31.

604. Shiff C. Efficacy of sulfadoxine-pyrimethamine as first line treatment for malaria declining in Malawi. *Evidence-based Healthcare and Public Health* 2004; **8**(5): 280-3.

605. Staedke SG, Mpimbaza A, Kamya MR, Nzarubara BK, Dorsey G, Rosenthal PJ. Amodiaquine based combination therapy is more effective than chloroquine-based therapy in children with uncomplicated falciparum malaria. *Evidence-based Healthcare and Public Health* 2005; **9**(3): 214-42.

606. Allloueche A, Bailey W, Barton S, et al. Chlorproguanil-dapsone is more effective, but causes more adverse events compared with sulfadoxine pyrimethamine in children with uncomplicated falciparum malaria. *Evidence-based Healthcare and Public Health* 2004; **8**(6): 373-4.

607. Piola P, Nabasumba C, Turyakira E, et al. Efficacy and safety of artemether-lumefantrine compared with quinine in pregnant women with uncomplicated *Plasmodium falciparum* malaria: an open-label, randomised, non-inferiority trial. *Lancet Infect Dis* 2010; **10**(11): 762-9.

608. Sowunmi A, Sowunmi CO, Adedeji AA, Oduola AMJ. Comparison of artemether and artemether plus mefloquine in children with malaria and effects on viability of *Plasmodium falciparum ex vivo*. *Clin Drug Inv* 2001; **21**(1): 33-40.

609. Dharmawardena P, Rodrigo C, Mendis K, et al. Response of imported malaria patients to antimalarial medicines in Sri Lanka following malaria elimination. *PLoS One* 2017; **12**(11): e0188613.

610. Comer RD, Young MD, Porter JA, Jr., Gauld JR, Merritt W. Chloroquine resistance in *Plasmodium falciparum* malaria on the Pacific coast of Colombia. *Am J Trop Med Hyg* 1968; **17**(6): 795-9.

611. Walker AJ, Lopez-Antunano FJ. Response to drugs of South American strains of *Plasmodium falciparum*. *Trans R Soc Trop Med Hyg* 1968; **62**(5): 654-67.

612. Martelo OJ, Smoller M, Saladin TA. Malaria in American soldiers. *Arch Intern Med* 1969; **123**(4): 383-7.

613. Fisher GU, Gordon MP, Lobel HO, Runcik K. Malaria in soldiers returning from Vietnam. Epidemiologic, therapeutic, and clinical studies. *Am J Trop Med Hyg* 1970; **19**(1): 27-39.

614. Doberstyn EB, Hall AP, Vetvutanapibul K, Sonkon P. Single-dose therapy of Falciparum malaria using pyrimethamine in combination with diformyldapsone or sulfadoxine. *Am J Trop Med Hyg* 1976; **25**(1): 14-9.

615. Dondero TJ, Jr., Parsons RE, Ponnampalam JT. Studies on the resistance of malaria to chloroquine and to a combination of chloroquine and pyrimethamine in Peninsular Malaysia. *Trans R Soc Trop Med Hyg* 1976; **70**(2): 145-8.

616. Sucharit P, Harinasuta T, Chongsuphajaisiddhi T, Tongprasroeth N, Kasemsuth R. *In vivo* and *in vitro* studies of chloroquine-resistant malaria in Thailand. *Ann Trop Med Parasitol* 1977; **71**(4): 401-5.

617. Omer AH. Response of *Plasmodium falciparum* in Sudan to oral chloroquine. *Am J Trop Med Hyg* 1978; **27**(5): 853-7.

618. Dwivedi SR, Sahu H, Yadava RL, Roy RG, Pattanayak S. *In vivo* chloroquine sensitivity tests of *Plasmodium falciparum* in some parts of Uttar Pradesh and Haryana States. *Indian J Med Res* 1979; **70 Suppl**: 20-2.

619. Sucharit P, Suntharasamai P, Chintana T, Harinasuta T. *In vivo* and *in vitro* studies of quinine sensitivity of *Plasmodium falciparum* in Thailand. *Southeast Asian J Trop Med Public Health* 1979; **10**(1): 138-41.

620. Chongsuphajaisiddhi T, Sabchareon A. Sulfadoxine-pyrimethamine resistant falciparum malaria in Thai children. *Southeast Asian J Trop Med Public Health* 1981; **12**(3): 418-21.

621. Chongsuphajaisiddhi T, Sabcharoen A, Attanath P. *In vivo* and *in vitro* sensitivity of Falciparum malaria to quinine in Thai children. *Ann Trop Paediatr* 1981; **1**(1): 21-6.

622. Reacher M, Campbell CC, Freeman J, Doberstyn EB, Brandling-Bennett AD. Drug therapy for *Plasmodium falciparum* malaria resistant to pyrimethamine-sulfadoxine (Fansidar). A study of alternate regimens in Eastern Thailand, 1980. *Lancet* 1981; **2**(8255): 1066-9.

623. Dixon KE, Williams RG, Pongsupat T, Pitaktong U, Phintuyothin P. A comparative trial of Mefloquine and Fansidar in the treatment of falciparum malaria: failure of Fansidar. *Trans R Soc Trop Med Hyg* 1982; **76**(5): 664-7.

624. Tin F, Hlaing N, Lasserre R. Single-dose treatment of falciparum malaria with mefloquine: field studies with different doses in semi-immune adults and children in Burma. *Bull World Health Organ* 1982; **60**(6): 913-7.

625. Al-Tawil N, Akood MA. Response of falciparum malaria to a standard regimen of chloroquine in Khartoum Province, Sudan. *East Afr Med J* 1983; **60**(10): 663-8.

626. Chongsuphajaisiddhi T, Sabchareon A, Attanath P. Treatment of quinine resistant falciparum malaria in Thai children. *Southeast Asian J Trop Med Public Health* 1983; **14**(3): 357-62.

627. Noeypatimanond S, Malikul S, Benjapong W, Duriyananda D, Ungkasrithongkul M. Treatment of *Plasmodium falciparum* malaria with a combination of amodiaquine and tetracycline in Central Thailand. *Trans R Soc Trop Med Hyg* 1983; **77**(3): 338-40.

628. Phillips RE, Looareesuwan S, Karbwang J, et al. Failure of chloroquine-erythromycin and chloroquine-tetracycline combinations in treatment of chloroquine-resistant falciparum malaria in eastern Thailand. *Lancet* 1984; **1**(8372): 300-2.

629. Lwin M, Htut Y, Oo M. The *in vivo* and *in vitro* sensitivity of *Plasmodium falciparum* to quinine. *Southeast Asian J Trop Med Public Health* 1985; **16**(2): 214-8.

630. Tin F, Hlaing N, Tun T, Win S, Lasserre R. Falciparum malaria treated with a fixed combination of mefloquine, sulfadoxine and pyrimethamine: a field study in adults in Burma. *Bull World Health Organ* 1985; **63**(4): 727-30.

631. de Souza JM, Sheth UK, Wernsdorfer WH, Trigg PI, Doberstyn EB. A phase II/III double-blind, dose-finding clinical trial of a combination of mefloquine, sulfadoxine, and pyrimethamine (Fansimef) in falciparum malaria. *Bull World Health Organ* 1987; **65**(3): 357-61.

632. Rieckmann K, Suebsaeng L, Rooney W. Response of *Plasmodium falciparum* infections to pyrimethamine-sulfadoxine in Thailand. *Am J Trop Med Hyg* 1987; **37**(2): 211-6.

633. Tin F, Nyunt H, Lim MA, Win S, Lasserre R. Double-blind trial to find dose range using a fixed combination of mefloquine, sulfadoxine and pyrimethamine in falciparum malaria: a field study on adults in Burma. *Trans R Soc Trop Med Hyg* 1987; **81**(6): 910-2.

634. Asthana OP, Tangri AN, Nityanand S. Clinical trial of fansimef in Indian patients of *P. falciparum* malaria. *Indian J Malariol* 1988; **25**(2): 89-96.

635. Guo XB, Arnold K, Fu LC, Chen PQ, Li GQ. Double-blind dose finding study of mefloquine-sulfadoxine-pyrimethamine in children with acute falciparum malaria. *Trans R Soc Trop Med Hyg* 1988; **82**(4): 538-40.

636. Huang OL, Ouyang WC, Zhou JX, et al. Effectiveness of amodiaquine, sulfadoxine-pyrimethamine, and combinations of these drugs for treating chloroquine-resistant falciparum malaria in Hainan Island, China. *Bull World Health Organ* 1988; **66**(3): 353-8.

637. Kremsner PG, Zotter GM, Feldmeier H, Graninger W, Rocha RM, Wiedermann G. A comparative trial of three regimens for treating uncomplicated falciparum malaria in Acre, Brazil. *J Infect Dis* 1988; **158**(6): 1368-71.

638. Sabchareon A, Chongsuphajaisiddhi T, Sinhasivanon V, Chanthavanich P, Attanath P. *In vivo* and *in vitro* responses to quinine and quinidine of *Plasmodium falciparum*. *Bull World Health Organ* 1988; **66**(3): 347-52.

639. Thet N, Hla W, Yin YN. Falciparum malaria and pregnancy: relationship and treatment response. *Southeast Asian J Trop Med Public Health* 1988; **19**(2): 253-8.

640. Tin S, Pe TM, Ye H, Win M, Lin S. The effect of mefloquine-artemether compared with quinine on patients with complicated falciparum malaria. *Trans R Soc Trop Med Hyg* 1988; **82**(5): 665-6.

641. Bunnag D, Harinasuta T, Looareesuwan S, et al. A combination of quinine, quinidine and cinchonine (LA 40221) in the treatment of chloroquine resistant falciparum malaria in Thailand: two double-blind trials. *Trans R Soc Trop Med Hyg* 1989; **83**(1): 66.

642. Chitchang S, Wongteptien S. A clinical trial of halofantrine in acute uncomplicated malaria in Thai soldiers. *Parasit Today* 1989; **5**(Suppl): 21-6.

643. Julvez J, Galtier J. [*In vivo* study of chloroquine resistance of *Plasmodium falciparum* malaria in Mayotte]. *Med Trop (Mars)* 1989; **49**(1): 63-5.

644. Maynadie M, Peceno C, Noriega PL, Yarzabal L. Susceptibility of *Plasmodium falciparum* strains to chloroquine and mefloquine in the Amazonas Federal Territory of Venezuela. *Trans R Soc Trop Med Hyg* 1989; **83**(5): 586-8.

645. Rab SM, Sheikhani MS, Mahmoud SA, Jaffary SIH. The efficacy of halofantrine hydrochloride in acute malaria: A study of 74 patients from Karachi, Pakistan. *Parasit Today* 1989; **5**(Suppl): 37-44.

646. Schuurkamp GJ, Kereu RK. Resistance of *Plasmodium falciparum* to chemotherapy with 4-aminoquinolines in the Ok Tedi area of Papua New Guinea. *P N G Med J* 1989; **32**(1): 33-44.

647. Arnold K, Tran TH, Nguyen TC, Nguyen HP, Pham P. A randomized comparative study of artemisinine (qinghaosu) suppositories and oral quinine in acute falciparum malaria. *Trans R Soc Trop Med Hyg* 1990; **84**(4): 499-502.

648. Arora U, Sharma RS, Singh G, Dasgupta RK, Narasimham MV. *In vivo* test for sensitivity of *Plasmodium falciparum* to chloroquine in Faridabad complex of Haryana (India). *Indian J Malariol* 1990; **27**(4): 205-8.

649. Harinasuta T, Bunnag D, Lasserre R. Quinine resistant falciparum malaria treated with mefloquine. *Southeast Asian J Trop Med Public Health* 1990; **21**(4): 552-7.

650. Pandya AP, Barkakaty BN, Narasimham MV, Mahapatra PK. *Plasmodium falciparum*--chloroquine *in vivo* test in northeast India: reclassification and extended follow-up till day 14. *Indian J Malariol* 1990; **27**(4): 223-9.

651. Thimasarn K, Pinichpongse S, Malikul S, Rooney W, Tansophalaks S. Phase III double-blind comparative study of Fansimef and Lariam for the curative treatment of *Plasmodium falciparum* infections in Thailand. *Southeast Asian J Trop Med Public Health* 1990; **21**(3): 404-11.

652. Hien TT, Tam DT, Cuc NT, Arnold K. Comparative effectiveness of artemisinin suppositories and oral quinine in children with acute falciparum malaria. *Trans R Soc Trop Med Hyg* 1991; **85**(2): 210-1.

653. Nosten F, ter Kuile F, Chongsuphajaisiddhi T, et al. Mefloquine-resistant falciparum malaria on the Thai-Burmese border. *Lancet* 1991; **337**(8750): 1140-3.

654. Salazar NP, Carlos CC, Bustos DG, Quiambao BP, Saniel MC, Santos MN. Halofantrine in the treatment of acute uncomplicated falciparum malaria in the Philippines. *Southeast Asian J Trop Med Public Health* 1991; **22**(3): 386-92.

655. Shao BR, Huang ZS, Shi XH, Meng F. A 5-year surveillance of sensitivity *in vivo* of *Plasmodium falciparum* to pyronaridine/sulfadoxine/pyrimethamine in Diaoluo area, Hainan Province. *Southeast Asian J Trop Med Public Health* 1991; **22**(1): 65-7.

656. de Andrade JG, de Andrade AL, Araujo ES, et al. A randomized clinical trial with high dose of chloroquine for treatment of *Plasmodium falciparum* malaria in Brazil. *Rev Inst Med Trop Sao Paulo* 1992; **34**(5): 467-73.

657. Ketrangsee S, Vijaykadga S, Yamokgul P, Jatapadma S, Thimasarn K, Rooney W. Comparative trial on the response of *Plasmodium falciparum* to halofantrine and mefloquine in Trat Province, eastern Thailand. *Southeast Asian J Trop Med Public Health* 1992; **23**(1): 55-8.

658. Looareesuwan S, Viravan C, Vanijanonta S, et al. Randomised trial of artesunate and mefloquine alone and in sequence for acute uncomplicated falciparum malaria. *Lancet* 1992; **339**(8797): 821-4.

659. Looareesuwan S, Wilairatana P, Vanijanonta S, Kyle D, Webster K. Efficacy of quinine-tetracycline for acute uncomplicated falciparum malaria in Thailand. *Lancet* 1992; **339**(8789): 369.

660. ter Kuile FO, Nosten F, Thieren M, et al. High-dose mefloquine in the treatment of multidrug-resistant falciparum malaria. *J Infect Dis* 1992; **166**(6): 1393-400.

661. Weinke T, Loscher T, Fleischer K, et al. The efficacy of halofantrine in the treatment of acute malaria in nonimmune travelers. *Am J Trop Med Hyg* 1992; **47**(1): 1-5.

662. Bunnag D, Viravan C, Karbwang J, et al. Clinical trials with halofantrine in acute uncomplicated falciparum malaria in Thailand. *Southeast Asian J Trop Med Public Health* 1993; **24**(1): 43-8.

663. Edrissian GH, Afshar A, Sayedzadeh A, Mohsseni G, Satvat MT. Assessment of the response *in vivo* and *in vitro* of *Plasmodium falciparum* to sulphadoxine-pyrimethamine in the malarious areas of Iran. *J Trop Med Hyg* 1993; **96**(4): 237-40.

664. Elhassan IM, Satti GH, Ali AE, et al. The efficacy of artemether in the treatment of *Plasmodium falciparum* malaria in Sudan. *Trans R Soc Trop Med Hyg* 1993; **87**(6): 685-6.

665. Jagota SC. Halofantrine in the treatment of acute malaria: a multi-centre study in 268 patients. *Curr Med Res Opin* 1993; **13**(3): 140-4.

666. Nguyen DS, Dao BH, Nguyen PD, et al. Treatment of malaria in Vietnam with oral artemisinin. *Am J Trop Med Hyg* 1993; **48**(3): 398-402.

667. Nosten F, ter Kuile F, Thwai KL, Maelankirri L, White NJ. Spiramycin does not potentiate quinine treatment of falciparum malaria in pregnancy. *Trans R Soc Trop Med Hyg* 1993; **87**(3): 305-6.

668. Rao KS, Kamalakar KV. Efficacy and safety of halofantrine in acute malaria. *J Assoc Physicians India* 1993; **41**(8): 507-8.

669. Smithuis FM, van Woensel JB, Nordlander E, Vantha WS, ter Kuile FO. Comparison of two mefloquine regimens for treatment of *Plasmodium falciparum* malaria on the northeastern Thai-Cambodian border. *Antimicrob Agents Chemother* 1993; **37**(9): 1977-81.

670. ter Kuile FO, Dolan G, Nosten F, et al. Halofantrine versus mefloquine in treatment of multidrug-resistant falciparum malaria. *Lancet* 1993; **341**(8852): 1044-9.

671. Tripathi KD, Sharma AK, Valecha N, Kulpati DD. Curative efficacy of norfloxacin in falciparum malaria. *Indian J Med Res* 1993; **97**: 176-8.

672. Bouchaud O, Basco LK, Gillotin C, et al. Clinical efficacy and pharmacokinetics of micronized halofantrine for the treatment of acute uncomplicated falciparum malaria in nonimmune patients. *Am J Trop Med Hyg* 1994; **51**(2): 204-13.

673. Fontanet AL, Johnston BD, Walker AM, Bergqvist Y, Hellgren U, Rooney W. Falciparum malaria in eastern Thailand: a randomized trial of the efficacy of a single dose of mefloquine. *Bull World Health Organ* 1994; **72**(1): 73-8.

674. Kain KC, Gopinath R, Yau Y, Temahivong T, Wongsrichanalai C. *In vivo* response of falciparum malaria to chloroquine in southern Thailand. *J Infect Dis* 1994; **170**(1): 258-9.

675. Luxemburger C, ter Kuile FO, Nosten F, et al. Single day mefloquine-artesunate combination in the treatment of multi-drug resistant falciparum malaria. *Trans R Soc Trop Med Hyg* 1994; **88**(2): 213-7.

676. Nosten F, Luxemburger C, ter Kuile FO, et al. Treatment of multidrug-resistant *Plasmodium falciparum* malaria with 3-day artesunate-mefloquine combination. *J Infect Dis* 1994; **170**(4): 971-7.

677. Tran TH, Arnold K, Nguyen TH, et al. Single dose artemisinin-mefloquine treatment for acute uncomplicated falciparum malaria. *Trans R Soc Trop Med Hyg* 1994; **88**(6): 688-91.

678. Watt G, Loesuttiviboon L, Jongsakul K, et al. Efficacy and tolerance of extended-dose halofantrine for drug-resistant falciparum malaria in Thailand. *Am J Trop Med Hyg* 1994; **50**(2): 187-92.

679. Chiodini PL, Conlon CP, Hutchinson DB, et al. Evaluation of atovaquone in the treatment of patients with uncomplicated *Plasmodium falciparum* malaria. *J Antimicrob Chemother* 1995; **36**(6): 1073-8.

680. Luxemburger C, Nosten F, Raimond SD, Chongsuphajaisiddhi T, White NJ. Oral artesunate in the treatment of uncomplicated hyperparasitemic falciparum malaria. *Am J Trop Med Hyg* 1995; **53**(5): 522-5.

681. Mishra SK, Asthana OP, Mohanty S, et al. Effectiveness of alpha,beta-arteether in acute falciparum malaria. *Trans R Soc Trop Med Hyg* 1995; **89**(3): 299-301.

682. Misra SP, Nandi J, Lal S. Chloroquine versus amodiaquine in the treatment of *Plasmodium falciparum* malaria in northeast India. *Indian J Med Res* 1995; **102**: 119-23.

683. Price RN, Nosten F, Luxemburger C, et al. Artesunate versus artemether in combination with mefloquine for the treatment of multidrug-resistant falciparum malaria. *Trans R Soc Trop Med Hyg* 1995; **89**(5): 523-7.

684. Soto J, Martinez R, Fonschiffrey G, Dember J. Efficacy of Fansidar for acute uncomplicated malaria due to *Plasmodium falciparum* in Colombia. *Trans R Soc Trop Med Hyg* 1995; **89**(3): 306.

685. Thimasarn K, Sirichaisinthop J, Vijaykadga S, et al. In vivo study of the response of *Plasmodium falciparum* to standard mefloquine/sulfadoxine/pyrimethamine (MSP) treatment among gem miners returning from Cambodia. *Southeast Asian J Trop Med Public Health* 1995; **26**(2): 204-12.

686. Baird JK, Caneta-Miguel E, Masbar S, et al. Survey of resistance to chloroquine of falciparum and vivax malaria in Palawan, The Philippines. *Trans R Soc Trop Med Hyg* 1996; **90**(4): 413-4.

687. Bich NN, De Vries PJ, Van Thien H, et al. Efficacy and tolerance of artemisinin in short combination regimens for the treatment of uncomplicated falciparum malaria. *Am J Trop Med Hyg* 1996; **55**(4): 438-43.

688. Handunnetti SM, Gunewardena DM, Pathirana PP, Ekanayake K, Weerasinghe S, Mendis KN. Features of recrudescent chloroquine-resistant *Plasmodium falciparum* infections confer a survival advantage on parasites and have implications for disease control. *Trans R Soc Trop Med Hyg* 1996; **90**(5): 563-7.

689. Lokman HS, Sharifah RSW, Zurkurnai Y, et al. *Plasmodium falciparum*: increased proportion of severe resistance (RII and RIII) to chloroquine and high rate of resistance to sulfadoxine-pyrimethamine in Peninsular Malaysia after two decades. *Trans R Soc Trop Med Hyg* 1996; **90**(3): 294-7.

690. Looareesuwan S, Viravan C, Vanijanonta S, Wilairatana P, Pitisuttithum P, Andrial M. Comparative clinical trial of artesunate followed by mefloquine in the treatment of acute uncomplicated falciparum malaria: two- and three-day regimens. *Am J Trop Med Hyg* 1996; **54**(2): 210-3.

691. Baird JK, Wiady I, Fryauff DJ, et al. *In vivo* resistance to chloroquine by *Plasmodium vivax* and *Plasmodium falciparum* at Nabire, Irian Jaya, Indonesia. *Am J Trop Med Hyg* 1997; **56**(6): 627-31.

692. Fryauff DJ, Baird JK, Basri H, et al. Halofantrine and primaquine for radical cure of malaria in Irian Jaya, Indonesia. *Ann Trop Med Parasitol* 1997; **91**(1): 7-16.

693. Fryauff DJ, Baird JK, Candradikusuma D, et al. Survey of *in vivo* sensitivity to chloroquine by *Plasmodium falciparum* and *P. vivax* in Lombok, Indonesia. *Am J Trop Med Hyg* 1997; **56**(2): 241-4.

694. Le NN, de Vries PJ, Le TD, et al. Single dose artemisinin-mefloquine versus mefloquine alone for uncomplicated falciparum malaria. *Trans R Soc Trop Med Hyg* 1997; **91**(2): 191-4.

695. Lwin M, Aung S, Kyaw MP, et al. A simplified *in vivo* drug sensitivity test for malaria in the field. *Southeast Asian J Trop Med Public Health* 1997; **28**(2): 247-53.

696. Price RN, Nosten F, Luxemburger C, et al. Artesunate/mefloquine treatment of multi-drug resistant falciparum malaria. *Trans R Soc Trop Med Hyg* 1997; **91**(5): 574-7.

697. Rowland M, Durrani N, Hewitt S, Sondorp E. Resistance of falciparum malaria to chloroquine and sulfadoxine-pyrimethamine in Afghan refugee settlements in western Pakistan: surveys by the general health services using a simplified *in vivo* test. *Trop Med Int Health* 1997; **2**(11): 1049-56.

698. Segurado AA, di Santi SM, Shiroma M. *In vivo* and *in vitro Plasmodium falciparum* resistance to chloroquine, amodiaquine and quinine in the Brazilian Amazon. *Rev Inst Med Trop Sao Paulo* 1997; **39**(2): 85-90.

699. Smithuis FM, Monti F, Grundl M, et al. *Plasmodium falciparum*: sensitivity *in vivo* to chloroquine, pyrimethamine/sulfadoxine and mefloquine in western Myanmar. *Trans R Soc Trop Med Hyg* 1997; **91**(4): 468-72.

700. Villalobos Salcedo JM, Aranha Camargo LM, De Fatima Vital Braga M, Soares De Maria P, De Oliveira Macedo V. Effectiveness evaluation of artesunate associated with tetracycline in falciparum malaria therapy. *Rev Soc Bras Med Trop* 1997; **30**(3): 215-22.

701. Wilairatana P, Kyle DE, Looareesuwan S, et al. Poor efficacy of antimalarial biguanide-dapsone combinations in the treatment of acute, uncomplicated, falciparum malaria in Thailand. *Ann Trop Med Parasitol* 1997; **91**(2): 125-32.

702. Ezzet F, Mull R, Karbwang J. Population pharmacokinetics and therapeutic response of CGP 56697 (artemether + benflumetol) in malaria patients. *Br J Clin Pharmacol* 1998; **46**(6): 553-61.

703. Fryauff DJ, Soekartono, Tuti S, et al. Survey of resistance *in vivo* to chloroquine of *Plasmodium falciparum* and *P. vivax* in North Sulawesi, Indonesia. *Trans R Soc Trop Med Hyg* 1998; **92**(1): 82-3.

704. Fryauff DJ, Tuti S, Mardi A, et al. Chloroquine-resistant *Plasmodium vivax* in transmigration settlements of West Kalimantan, Indonesia. *Am J Trop Med Hyg* 1998; **59**(4): 513-8.

705. Hong-Ping G, Yao Z, Qian D, Jin-Hua Y, Kai-Xun J, Min Z. The *in vivo* sensitivity of *Plasmodium falciparum* to chloroquine in the Red River basin, Yunnan, China. *Southeast Asian J Trop Med Public Health* 1998; **29**(4): 692-5.

706. McGready R, Cho T, Cho JJ, et al. Artemisinin derivatives in the treatment of falciparum malaria in pregnancy. *Trans R Soc Trop Med Hyg* 1998; **92**(4): 430-3.

707. Price R, Luxemburger C, van Vugt M, et al. Artesunate and mefloquine in the treatment of uncomplicated multidrug-resistant hyperparasitaemic falciparum malaria. *Trans R Soc Trop Med Hyg* 1998; **92**(2): 207-11.

708. Price R, van Vugt M, Nosten F, et al. Artesunate versus artemether for the treatment of recrudescent multidrug-resistant falciparum malaria. *Am J Trop Med Hyg* 1998; **59**(6): 883-8.

709. Sabchareon A, Attanath P, Chanthavanich P, et al. Comparative clinical trial of artesunate suppositories and oral artesunate in combination with mefloquine in the treatment of children with acute falciparum malaria. *Am J Trop Med Hyg* 1998; **58**(1): 11-6.

710. Singh N, Shukla MM, Asthana OP, Sharma VP. Effectiveness of alpha-beta arteether in clearing *Plasmodium falciparum* parasitemia in central India (Madhya Pradesh). *Southeast Asian J Trop Med Public Health* 1998; **29**(2): 225-7.

711. Bustos DG, Canfield CJ, Canete-Miguel E, Hutchinson DB. Atovaquone-proguanil compared with chloroquine and chloroquine-sulfadoxine-pyrimethamine for treatment of acute *Plasmodium falciparum* malaria in the Philippines. *J Infect Dis* 1999; **179**(6): 1587-90.

712. Fryauff DJ, Sumawinata I, Purnomo, et al. *In vivo* responses to antimalarials by *Plasmodium falciparum* and *Plasmodium vivax* from isolated Gag Island off northwest Irian Jaya, Indonesia. *Am J Trop Med Hyg* 1999; **60**(4): 542-6.

713. Kaneko A, Bergqvist Y, Takechi M, et al. Intrinsic efficacy of proguanil against falciparum and vivax malaria independent of the metabolite cycloguanil. *J Infect Dis* 1999; **179**(4): 974-9.

714. Looareesuwan S, Wilairatana P, Chokejindachai W, et al. A randomized, double-blind, comparative trial of a new oral combination of artemether and benflumetol (CGP 56697) with mefloquine in the treatment of acute *Plasmodium falciparum* malaria in Thailand. *Am J Trop Med Hyg* 1999; **60**(2): 238-43.

715. McGready R, Nosten F. The Thai-Burmese border: drug studies of *Plasmodium falciparum* in pregnancy. *Ann Trop Med Parasitol* 1999; **93 Suppl 1**: S19-23.

716. Bouchaud O, Monlun E, Muanza K, et al. Atovaquone plus proguanil versus halofantrine for the treatment of imported acute uncomplicated *Plasmodium falciparum* malaria in non-immune adults: a randomized comparative trial. *Am J Trop Med Hyg* 2000; **63**(5-6): 274-9.

717. de Vries PJ, Bich NN, Van Thien H, et al. Combinations of artemisinin and quinine for uncomplicated falciparum malaria: efficacy and pharmacodynamics. *Antimicrob Agents Chemother* 2000; **44**(5): 1302-8.

718. Gogtay NJ, Desai S, Kadam VS, Kamtekar KD, Dalvi SS, Kshirsagar NA. A randomized, parallel-group study in Mumbai (Bombay), comparing chloroquine with chloroquine plus sulfadoxine-pyrimethamine in the treatment of adults with acute, uncomplicated, *Plasmodium falciparum* malaria. *Ann Trop Med Parasitol* 2000; **94**(4): 309-12.

719. Alecrim MG, Carvalho LM, Fernandes MC, et al. [Malaria treatment with artesunate (retocaps) in children of the Brazilian Amazon]. *Rev Soc Bras Med Trop* 2000; **33**(2): 163-8.

720. van Vugt M, Looareesuwan S, Wilairatana P, et al. Artemether-lumefantrine for the treatment of multidrug-resistant falciparum malaria. *Trans R Soc Trop Med Hyg* 2000; **94**(5): 545-8.

721. Giao PT, Binh TQ, Kager PA, et al. Artemisinin for treatment of uncomplicated falciparum malaria: is there a place for monotherapy? *Am J Trop Med Hyg* 2001; **65**(6): 690-5.

722. Huong NM, Hewitt S, Davis TM, et al. Resistance of *Plasmodium falciparum* to antimalarial drugs in a highly endemic area of southern Viet Nam: a study *in vivo* and *in vitro*. *Trans R Soc Trop Med Hyg* 2001; **95**(3): 325-9.

723. Llanos-Cuentas A, Campos P, Clendenes M, Canfield CJ, Hutchinson DB. Atovaquone and proguani hydrochloride compared with chloroquine or pyrimethamine/sulfodaxine for treatment of acute *Plasmodium falciparum* malaria in Peru. *Braz J Infect Dis* 2001; **5**(2): 67-72.

724. Parola P, Ranque S, Badiaga S, et al. Controlled trial of 3-day quinine-clindamycin treatment versus 7-day quinine treatment for adult travelers with uncomplicated falciparum malaria imported from the tropics. *Antimicrob Agents Chemother* 2001; **45**(3): 932-5.

725. Singh N, Saxena A, Sharma VP. Status of chloroquine efficacy against *Plasmodium falciparum* in pregnant women in a tribal area of central India. *Curr Science* 2001; **80**(5): 618-20.

726. Trung TN, Davis TM, Hewitt S, et al. Treatment of falciparum malaria in Vietnamese children: the need for combination therapy and optimized dosage regimens. *Ann Trop Paediatr* 2001; **21**(4): 307-12.

727. Baird JK, Wiady I, Sutanihardja A, et al. Short report: therapeutic efficacy of chloroquine combined with primaquine against *Plasmodium falciparum* in northeastern Papua, Indonesia. *Am J Trop Med Hyg* 2002; **66**(6): 659-60.

728. Guthmann JP, Kasparian S, Phetsouvanh R, et al. The efficacy of chloroquine for the treatment of acute, uncomplicated, *Plasmodium falciparum* malaria in Laos. *Ann Trop Med Parasitol* 2002; **96**(6): 553-7.

729. Krudsood S, Buchachart K, Chalermrut K, et al. A comparative clinical trial of combinations of dihydroartemisinin plus azithromycin and dihydroartemisinin plus mefloquine for treatment of multidrug resistant falciparum malaria. *Southeast Asian J Trop Med Public Health* 2002; **33**(3): 525-31.

730. McGready R, Thwai KL, Cho T, et al. The effects of quinine and chloroquine antimalarial treatments in the first trimester of pregnancy. *Trans R Soc Trop Med Hyg* 2002; **96**(2): 180-4.

731. Weerasinghe KL, Galappaththy G, Fernando WP, Wickremasinghe DR, Faizal HM, Wickremasinghe AR. A safety and efficacy trial of artesunate, sulphadoxine-pyrimethamine and primaquine in *P falciparum* malaria. *Ceylon Med J* 2002; **47**(3): 83-5.

732. Biswas S, Valecha N, Tyagi PK, et al. Assessment of therapeutic efficacy of chloroquine and sulphadoxine-pyrimethamine in uncomplicated falciparum malaria. *J Vector Borne Dis* 2003; **40**(3-4): 92-9.

733. Doan HN, Taylor WR, Nguyen DT, et al. Short report: *in vivo* sensitivity of *Plasmodium falciparum* to halofantrine in southern central Vietnam. *Am J Trop Med Hyg* 2003; **69**(5): 553-4.

734. Gomez EA, Jurado MH, Cambon N. Randomised efficacy and safety study of two 3-day artesunate rectal capsule/mefloquine regimens versus artesunate alone for uncomplicated malaria in Ecuadorian children. *Acta Trop* 2003; **89**(1): 47-53.

735. Karunajeewa HA, Kemiki A, Alpers MP, et al. Safety and therapeutic efficacy of artesunate suppositories for treatment of malaria in children in Papua New Guinea. *Pediatr Infect Dis J* 2003; **22**(3): 251-6.

736. Marquino W, Huilca M, Calampa C, et al. Efficacy of mefloquine and a mefloquine-artesunate combination therapy for the treatment of uncomplicated *Plasmodium falciparum* malaria in the Amazon Basin of Peru. *Am J Trop Med Hyg* 2003; **68**(5): 608-12.

737. Mayxay M, Newton PN, Khanthavong M, et al. Chloroquine versus sulfadoxine-pyrimethamine for treatment of *Plasmodium falciparum* malaria in Savannakhet Province, Lao People's Democratic Republic: an assessment of national antimalarial drug recommendations. *Clin Infect Dis* 2003; **37**(8): 1021-8.

738. Mohapatra PK, Namchoom NS, Prakash A, Bhattacharya DR, Goswami BK, Mahanta J. Therapeutic efficacy of anti-malarials in *Plasmodium falciparum* malaria in an Indo-Myanmar border area of Arunachal Pradesh. *Indian J Med Res* 2003; **118**: 71-6.

739. Rojanawatsirivej C, Vijaykadga S, Amklad I, Wilairatna P, Looareesuwan S. Monitoring the therapeutic efficacy of antimalarials against uncomplicated falciparum malaria in Thailand. *Southeast Asian J Trop Med Public Health* 2003; **34**(3): 536-41.

740. Wattanakoon Y, Chittamas S, Pornkulprasit V, et al. Six-years monitoring the efficacy of the combination of artesunate and mefloquine for the treatment of uncomplicated falciparum malaria. *Southeast Asian J Trop Med Public Health* 2003; **34**(3): 542-5.

741. Adam I, Elwasila E, Mohammed Ali DA, Elansari E, Elbashir MI. Artemether in the treatment of falciparum malaria during pregnancy in eastern Sudan. *Trans R Soc Trop Med Hyg* 2004; **98**(9): 509-13.

742. Adam I, Ibrahim MH, IA Ae, Elbashir MI. Low-dose quinine for treatment of chloroquine-resistant falciparum malaria in Sudanese pregnant women. *East Mediterr Health J* 2004; **10**(4-5): 554-9.

743. Adam I, Idris HM, Elbashir MI. Quinine for chloroquine-resistant falciparum malaria in pregnant Sudanese women in the first trimester. *East Mediterr Health J* 2004; **10**(4-5): 560-5.

744. Adam I, Osman ME, Elghzali G, Ahmed GI, Gustafssons LL, Elbashir MI. Efficacies of chloroquine, sulfadoxine-pyrimethamine and quinine in the treatment of uncomplicated, *Plasmodium falciparum* malaria in eastern Sudan. *Ann Trop Med Parasitol* 2004; **98**(7): 661-6.

745. Avila JC, Villaroel R, Marquino W, Zegarra J, Mollinedo R, Ruebush TK. Efficacy of mefloquine and mefloquine-artesunate for the treatment of uncomplicated *Plasmodium falciparum* malaria in the Amazon region of Bolivia. *Trop Med Int Health* 2004; **9**(2): 217-21.

746. Giao PT, de Vries PJ, Hung le Q, Binh TQ, Nam NV, Kager PA. CV8, a new combination of dihydroartemisinin, piperaquine, trimethoprim and primaquine, compared with atovaquone-proguanil against falciparum malaria in Vietnam. *Trop Med Int Health* 2004; **9**(2): 209-16.

747. Hapuarachchi HA, Dayanath MY, Abeysundara S, Bandara KB, Abeyewickreme W, de Silva NR. Chloroquine resistant falciparum malaria among security forces personnel in the Northern Province of Sri Lanka. *Ceylon Med J* 2004; **49**(2): 47-51.

748. Hau I, Seringe S, Aberrane S, et al. Halofantrine efficacy in non-immune children with imported acute *Plasmodium falciparum* malaria Infection. *Eur J Pediatr* 2004; **163**(1): 22-4.

749. Hung lQ, de Vries PJ, Binh TQ, et al. Artesunate with mefloquine at various intervals for non-severe *Plasmodium falciparum* malaria. *Am J Trop Med Hyg* 2004; **71**(2): 160-6.

750. Ibrahim MH, Elbashir MI, Naser A, Aelbasit IA, Kheir MM, Adam I. Low-dose quinine is effective in the treatment of chloroquine-resistant *Plasmodium falciparum* malaria in eastern Sudan. *Ann Trop Med Parasitol* 2004; **98**(5): 441-5.

751. Kamtekar KD, Gogtay NJ, Dalvi SS, et al. A prospective study evaluating the efficacy of a single, 45-mg dose of primaquine, as a gametocytocidal agent, in patients with *Plasmodium falciparum* malaria in Mumbai, India. *Ann Trop Med Parasitol* 2004; **98**(5): 453-8.

752. Rahman M, Rahman R, Bangali M, Das S, Talukder MR, Ringwald P. Efficacy of combined chloroquine and sulfadoxine-pyrimethamine in uncomplicated *Plasmodium falciparum* malaria in Bangladesh. *Trans R Soc Trop Med Hyg* 2004; **98**(7): 438-41.

753. Randrianasolo L, Randriamanantena A, Ranarivelo L, Ratsimbasoa A, Domarle O, Randrianarivelojosia M. Monitoring susceptibility to sulfadoxine-pyrimethamine among cases of uncomplicated, *Plasmodium falciparum* malaria in Saharevo, Madagascar. *Ann Trop Med Parasitol* 2004; **98**(6): 551-4.

754. Rojanawatsirivet C, Congpuong K, Vijaykadga S, et al. Declining mefloquine sensitivity of *Plasmodium falciparum* along the Thai-Myanmar border. *Southeast Asian J Trop Med Public Health* 2004; **35**(3): 560-5.

755. Stivanello E, Cavailler P, Cassano F, et al. Efficacy of chloroquine, sulphadoxine-pyrimethamine and amodiaquine for treatment of uncomplicated *Plasmodium falciparum* malaria in Kajo Keji county, Sudan. *Trop Med Int Health* 2004; **9**(9): 975-80.

756. Thybo S, Gjorup I, Ronn AM, Meyrowitsch D, Bygberg IC. Atovaquone-proguanil (malarone): an effective treatment for uncomplicated *Plasmodium falciparum* malaria in travelers from Denmark. *J Travel Med* 2004; **11**(4): 220-3.

757. Tran TH, Dolecek C, Pham PM, et al. Dihydroartemisinin-piperaquine against multidrug-resistant *Plasmodium falciparum* malaria in Vietnam: randomised clinical trial. *Lancet* 2004; **363**(9402): 18-22.

758. Adam I, A-Elbasit IE, Elbashir MI. Efficacies of mefloquine alone and of artesunate followed by mefloquine, for the treatment of uncomplicated, *Plasmodium falciparum* malaria in eastern Sudan. *Ann Trop Med Parasitol* 2005; **99**(2): 111-7.

759. Adam I, IE AE, Idris SM, Malik EM, Elbashir MI. A comparison of the efficacy of artesunate plus sulfadoxine-pyrimethamine with that of sulfadoxine-pyrimethamine alone, in the treatment of uncomplicated, *Plasmodium falciparum* malaria in eastern Sudan. *Ann Trop Med Parasitol* 2005; **99**(5): 449-55.

760. Adam I, Ali DM, Noureldien W, Elbashir MI. Quinine for the treatment of chloroquine-resistant *Plasmodium falciparum* malaria in pregnant and non-pregnant Sudanese women. *Ann Trop Med Parasitol* 2005; **99**(4): 427-9.

761. Adam I, Salih I, Elbashir MI. Quinine for the treatment of uncomplicated *Plasmodium falciparum* malaria in eastern Sudan. *Trans R Soc Trop Med Hyg* 2005; **99**(10): 736-8.

762. Dunne MW, Singh N, Shukla M, et al. A multicenter study of azithromycin, alone and in combination with chloroquine, for the treatment of acute uncomplicated *Plasmodium falciparum* malaria in India. *J Infect Dis* 2005; **191**(10): 1582-8.

763. Hamour S, Melaku Y, Keus K, et al. Malaria in the Nuba Mountains of Sudan: baseline genotypic resistance and efficacy of the artesunate plus sulfadoxine-pyrimethamine and artesunate plus amodiaquine combinations. *Trans R Soc Trop Med Hyg* 2005; **99**(7): 548-54.

764. Jima D, Tesfaye G, Medhin A, Kebede A, Argaw D, Babaniyi O. Safety and efficacy of artemether-lumefantrine in the treatment of uncomplicated falciparum malaria in Ethiopia. *East Afr Med J* 2005; **82**(8): 387-90.

765. Jima D, Tesfaye G, Medhin A, Kebede A, Argaw D, Babaniyi O. Efficacy of sulfadoxine-pyrimethamine for the treatment of uncomplicated falciparum malaria in Ethiopia. *East Afr Med J* 2005; **82**(8): 391-5.

766. Matteelli A, Saleri N, Bisoffi Z, et al. Mefloquine versus quinine plus sulphalene-pyrimethamine (metakelfin) for treatment of uncomplicated imported falciparum malaria acquired in Africa. *Antimicrob Agents Chemother* 2005; **49**(2): 663-7.

767. Silachamroon U, Krudsood S, Thanachartwet W, et al. An open, randomized trial of three-day treatment with artesunate combined with a standard dose of mefloquine divided over either two or three days, for acute, uncomplicated falciparum malaria. *Southeast Asian J Trop Med Public Health* 2005; **36**(3): 591-6.

768. Tangpukdee N, Krudsood S, Thanachartwet W, et al. An open randomized clinical trial of Artekin vs artesunate-mefloquine in the treatment of acute uncomplicated falciparum malaria. *Southeast Asian J Trop Med Public Health* 2005; **36**(5): 1085-91.

769. van den Broek I, Amsalu R, Balasegaram M, et al. Efficacy of two artemisinin combination therapies for uncomplicated falciparum malaria in children under 5 years, Malakal, Upper Nile, Sudan. *Malar J* 2005; **4**: 14.

770. Wijeyaratne PM, Chand PB, Valecha N, et al. Therapeutic efficacy of antimalarial drugs along the eastern Indo-Nepal border: a cross-border collaborative study. *Trans R Soc Trop Med Hyg* 2005; **99**(6): 423-9.

771. IE A-E, Elbashir MI, Khalil IF, Alifrangis M, Giha HA. The efficacy of sulfadoxine-pyrimethamine alone and in combination with chloroquine for malaria treatment in rural Eastern Sudan: the interrelation between resistance, age and gametocytogenesis. *Trop Med Int Health* 2006; **11**(5): 604-12.

772. Adam I, Ali DM, Abdalla MA. Artesunate plus sulfadoxine-pyrimethamine in the treatment of uncomplicated *Plasmodium falciparum* malaria during pregnancy in eastern Sudan. *Trans R Soc Trop Med Hyg* 2006; **100**(7): 632-5.

773. Adam I, Magzoub M, Osman ME, Khalil IF, Alifrangis M, Elmardi KA. A fixed-dose 24-hour regimen of artesunate plus sulfamethoxypyrazine-pyrimethamine for the treatment of uncomplicated *Plasmodium falciparum* malaria in eastern Sudan. *Ann Clin Microbiol Antimicrob* 2006; **5**: 18.

774. Blair S, Carmona-Fonseca J, Pineros JG, et al. Therapeutic efficacy test in malaria falciparum in Antioquia, Colombia. *Malar J* 2006; **5**: 14.

775. Campbell P, Baruah S, Narain K, Rogers CC. A randomized trial comparing the efficacy of four treatment regimens for uncomplicated falciparum malaria in Assam state, India. *Trans R Soc Trop Med Hyg* 2006; **100**(2): 108-18.

776. Mohamed AO, Eltaib EH, Ahmed OA, Elamin SB, Malik EM. The efficacies of artesunate-sulfadoxine-pyrimethamine and artemether-lumefantrine in the treatment of uncomplicated, *Plasmodium falciparum* malaria, in an area of low transmission in central Sudan. *Ann Trop Med Parasitol* 2006; **100**(1): 5-10.

777. Noedl H, Krudsood S, Chalermratana K, et al. Azithromycin combination therapy with artesunate or quinine for the treatment of uncomplicated *Plasmodium falciparum* malaria in adults: a randomized, phase 2 clinical trial in Thailand. *Clin Infect Dis* 2006; **43**(10): 1264-71.

778. Raeisi A, Ringwald P, Safa O, et al. Monitoring of the therapeutic efficacy of chloroquine for the treatment of uncomplicated, *Plasmodium falciparum* malaria in Iran. *Ann Trop Med Parasitol* 2006; **100**(1): 11-6.

779. Vijaykadga S, Rojanawatsirivej C, Cholpol S, Phoungmanee D, Nakavej A, Wongsrichanalai C. *In vivo* sensitivity monitoring of mefloquine monotherapy and artesunate-mefloquine combinations for the treatment of uncomplicated falciparum malaria in Thailand in 2003. *Trop Med Int Health* 2006; **11**(2): 211-9.

780. Ashley EA, Stepniewska K, Lindegardh N, et al. Pharmacokinetic study of artemether-lumefantrine given once daily for the treatment of uncomplicated multidrug-resistant falciparum malaria. *Trop Med Int Health* 2007; **12**(2): 201-8.

781. Diem Thuy le T, Na-Bangchang K, Hung le N, et al. Clinical efficacy of high dose monotherapy of oral dihydroartemisinin in uncomplicated falciparum malaria in viet nam. *Jpn J Infect Dis* 2007; **60**(4): 161-6.

782. Ejaz A, Haqnawaz K, Hussain Z, Butt R, Awan ZI, Bux H. Treatment of uncomplicated *plasmodium falciparum* malaria with quinine-doxycycline combination therapy. *J Pak Med Assoc* 2007; **57**(10): 502-5.

783. Elamin SB, Malik el FM, Ahmed el DS, Elabadil EK, Mohamad TA. Efficacy of chloroquine and sulfadoxine/pyrimethamine mono- and combined therapy against falciparum malaria in Sudan. *East Mediterr Health J* 2007; **13**(1): 25-34.

784. Ibrahium AM, Kheir MM, Osman ME, et al. Efficacies of artesunate plus either sulfadoxine-pyrimethamine or amodiaquine, for the treatment of uncomplicated, *Plasmodium falciparum* malaria in eastern Sudan. *Ann Trop Med Parasitol* 2007; **101**(1): 15-21.

785. Krudsood S, Patel SN, Tangpukdee N, et al. Efficacy of atovaquone-proguanil for treatment of acute multidrug-resistant *Plasmodium falciparum* malaria in Thailand. *Am J Trop Med Hyg* 2007; **76**(4): 655-8.

786. Krudsood S, Tangpukdee N, Thanchatwet V, et al. Dose ranging studies of new artemisinin-piperaquine fixed combinations compared to standard regimens of artemisisnin combination therapies for acute uncomplicated falciparum malaria. *Southeast Asian J Trop Med Public Health* 2007; **38**(6): 971-8.

787. Menard D, Andrianina NN, Ramiandrasoa Z, et al. Randomized clinical trial of artemisinin versus non-artemisinin combination therapy for uncomplicated falciparum malaria in Madagascar. *Malar J* 2007; **6**: 65.

788. Mukhtar EA, Gadalla NB, El-Zaki SE, et al. A comparative study on the efficacy of artesunate plus sulphadoxine/pyrimethamine versus artemether-lumefantrine in eastern Sudan. *Malar J* 2007; **6**: 92.

789. Na-Bangchang K, Ruengweerayut R, Karbwang J, Chauemung A, Hutchinson D. Pharmacokinetics and pharmacodynamics of fosmidomycin monotherapy and combination therapy with clindamycin in the treatment of multidrug resistant falciparum malaria. *Malar J* 2007; **6**: 70.

790. Osorio L, Gonzalez I, Olliaro P, Taylor WR. Artemisinin-based combination therapy for uncomplicated *Plasmodium falciparum* malaria in Colombia. *Malar J* 2007; **6**: 25.

791. Ranque S, Parola P, Adehossi E, Brouqui P, Delmont J. Mefloquine versus 3-day oral quinine-clindamycin in uncomplicated imported falciparum malaria. *Travel Med Infect Dis* 2007; **5**(5): 306-9.

792. Singh RK. Efficacy of combined treatment with alpha/beta-arteether and sulfadoxine-pyrimethamine, for cases of *Plasmodium falciparum* malaria in Jharkhand, India. *Ann Trop Med Parasitol* 2007; **101**(3): 271-3.

793. Sirivichayakul C, Sabchareon A, Pengsaa K, et al. Comparative study of the effectiveness and pharmacokinetics of two rectal artesunate/oral mefloquine combination regimens for the treatment of uncomplicated childhood falciparum malaria. *Ann Trop Paediatr* 2007; **27**(1): 17-24.

794. Kerketta AS, Mohapatra SS, Kar SK. Assessment of the therapeutic efficacy of chloroquine in the treatment of uncomplicated *Plasmodium falciparum* malaria in a tribal block of the Kalahandi district of Orissa, India. *Trop Doct* 2008; **38**(2): 82-4.

795. Le Thi DT, Le NH, Nguyen CH, Phan Thi D, Na-Bangchang K. Pharmacokinetics of a five-day oral dihydroartemisinin monotherapy regimen in patients with uncomplicated falciparum malaria. *Drug Metab Pharmacokinet* 2008; **23**(3): 158-64.

796. Menard D, Ratsimbasoa A, Randrianarivelojosia M, et al. Assessment of the efficacy of antimalarial drugs recommended by the National Malaria Control Programme in Madagascar: up-dated baseline data from randomized and multi-site clinical trials. *Malar J* 2008; **7**: 55.

797. Rahman MM, Dondorp AM, Day NP, et al. Adherence and efficacy of supervised versus non-supervised treatment with artemether/lumefantrine for the treatment of uncomplicated *Plasmodium falciparum* malaria in Bangladesh: a randomised controlled trial. *Trans R Soc Trop Med Hyg* 2008; **102**(9): 861-7.

798. Srivastava HC, Yadav RS, Joshi H, et al. Therapeutic responses of *Plasmodium vivax* and *P. falciparum* to chloroquine, in an area of western India where *P. vivax* predominates. *Ann Trop Med Parasitol* 2008; **102**(6): 471-80.

799. Al-Kabsi AM, Al-Shamahy HA, Al-Harazy AH, Harmal NS. The therapeutic efficacy of sulfadoxine/pyrimethamine against *Plasmodium falciparum* in Yemen. *Med Princ Pract* 2009; **18**(1): 62-6.

800. Asih PB, Dewi RM, Tuti S, et al. Efficacy of artemisinin-based combination therapy for treatment of persons with uncomplicated *Plasmodium falciparum* malaria in West Sumba District, East Nusa Tenggara Province, Indonesia, and genotypic profiles of the parasite. *Am J Trop Med Hyg* 2009; **80**(6): 914-8.

801. Gutman J, Green M, Durand S, et al. Mefloquine pharmacokinetics and mefloquine-artesunate effectiveness in Peruvian patients with uncomplicated *Plasmodium falciparum* malaria. *Malar J* 2009; **8**: 58.

802. Hombhanje FW, Linge D, Saweri A, et al. Artemisinin-naphthoquine combination (ARCO) therapy for uncomplicated falciparum malaria in adults of Papua New Guinea: a preliminary report on safety and efficacy. *Malar J* 2009; **8**: 196.

803. Kefyalew T, Animut A, Tamene T, Jima D, Hailemariam A, Legesse M. Efficacy of six-dose regimen of artemether-lumefantrine for the treatment of uncomplicated falciparum malaria, three years after its introduction into Ethiopia. *Parasite* 2009; **16**(2): 129-34.

804. Nateghpour M, Edrissian G, Torabi A, et al. Monitoring of *Plasmodium vivax* and *Plasmodium falciparum* response to chloroquine in Bandar-Abbas district, Hormozgan province, Iran. [Arabic]. *Tehran Uni Med J* 2009; **67**(3): 178-83.

805. Sagara I, Rulisa S, Mbacham W, et al. Efficacy and safety of a fixed dose artesunate-sulphamethoxypyrazine-pyrimethamine compared to artemether-lumefantrine for the treatment of uncomplicated falciparum malaria across Africa: a randomized multi-centre trial. *Malar J* 2009; **8**: 63.

806. Thanh NX, Trung TN, Phong NC, et al. Open label randomized comparison of dihydroartemisinin-piperaquine and artesunate-amodiaquine for the treatment of uncomplicated *Plasmodium falciparum* malaria in central Vietnam. *Trop Med Int Health* 2009; **14**(5): 504-11.

807. Trung TN, Tan B, Van Phuc D, Song JP. A randomized, controlled trial of artemisinin-piperaquine vs dihydroartemisinin-piperaquine phosphate in treatment of falciparum malaria. *Chin J Integr Med* 2009; **15**(3): 189-92.

808. Valecha N, Joshi H, Mallick PK, et al. Low efficacy of chloroquine: time to switchover to artemisinin-based combination therapy for falciparum malaria in India. *Acta Trop* 2009; **111**(1): 21-8.

809. Valecha N, Srivastava P, Mohanty SS, et al. Therapeutic efficacy of artemether-lumefantrine in uncomplicated falciparum malaria in India. *Malar J* 2009; **8**: 107.

810. Warsame M, Atta H, Klena JD, et al. Efficacy of monotherapies and artesunate-based combination therapies in children with uncomplicated malaria in Somalia. *Acta Trop* 2009; **109**(2): 146-51.

811. Adam I, Salah MT, Eltahir HG, Elhassan AH, Elmardi KA, Malik EM. Dihydroartemisinin-piperaquine versus artemether-lumefantrine, in the treatment of uncomplicated *Plasmodium falciparum* malaria in central Sudan. *Ann Trop Med Parasitol* 2010; **104**(4): 319-26.

812. Bharti PK, Alam MT, Boxer R, et al. Therapeutic efficacy of chloroquine and sequence variation in *pfcrt* gene among patients with falciparum malaria in central India. *Trop Med Int Health* 2010; **15**(1): 33-40.

813. Elamin SB, Awad AI, Eltayeb IB, et al. Descriptive study on the efficacy of artemether-lumefantrine in the treatment of uncomplicated *Plasmodium falciparum* malaria in Sudan. *Eur J Clin Pharmacol* 2010; **66**(3): 231-7.

814. Kochar DK, Gupta V, Kochar A, et al. Comparison of quinine and rabeprazole with quinine monotherapy in the treatment of uncomplicated falciparum malaria. *J Vector Borne Dis* 2010; **47**(3): 140-4.

815. Singh H, Dulhani N, Kumar BN, Tiwari P. A comparative study of artesunate-lumefantrine vs. chloroquine-pyrimethamine-sulfadoxine efficacy for the treatment of uncomplicated *Plasmodium falciparum* in tribal population in Bastar (Chhattisgarh). *Internet J Pharm* 2010; **8**: 1.

816. Valecha N, Phyo AP, Mayxay M, et al. An open-label, randomised study of dihydroartemisinin-piperaquine versus artesunate-mefloquine for falciparum malaria in Asia. *PLoS One* 2010; **5**(7): e11880.

817. de Oliveira AM, Chavez J, de Leon GP, et al. Efficacy and effectiveness of mefloquine and artesunate combination therapy for uncomplicated *Plasmodium falciparum* malaria in the Peruvian Amazon. *Am J Trop Med Hyg* 2011; **85**(3): 573-8.

818. Gadalla NB, Adam I, Elzaki SE, et al. Increased *pfmdr1* copy number and sequence polymorphisms in *Plasmodium falciparum* isolates from Sudanese malaria patients treated with artemether-lumefantrine. *Antimicrob Agents Chemother* 2011; **55**(11): 5408-11.

819. Gautam A, Ahmed T, Sharma P, et al. Pharmacokinetics and pharmacodynamics of arterolane maleate following multiple oral doses in adult patients with *P. falciparum* malaria. *J Clin Pharmacol* 2011; **51**(11): 1519-28.

820. Howard N, Durrani N, Sanda S, Beshir K, Hallett R, Rowland M. Clinical trial of extended-dose chloroquine for treatment of resistant falciparum malaria among Afghan refugees in Pakistan. *Malar J* 2011; **10**: 171.

821. Kawai A, Arita N, Matsumoto Y, Kawabata M, Chowdhury MS, Saito-Ito A. Efficacy of chloroquine plus primaquine treatment and *pfcrt* mutation in uncomplicated falciparum malaria patients in Rangamati, Bangladesh. *Parasitol Int* 2011; **60**(4): 341-6.

822. Makanga M, Bassat Q, Falade CO, et al. Efficacy and safety of artemether-lumefantrine in the treatment of acute, uncomplicated *Plasmodium falciparum* malaria: a pooled analysis. *Am J Trop Med Hyg* 2011; **85**(5): 793-804.

823. Marko JL, Chourishi A, Pandey SP. Comparison of efficacy of chloroquine alone, azithromycin alone, and chloroquine azithromycin combination for the treatment of uncomplicated *Plasmodium Falciparum* Malaria. *Int J Pharm Sci Rev Res* 2011; **11**(1): 7-12.

824. Marko JL, Pandey SP, Chourishi A. A comparative study of efficacy of Azithromycin and chloroquin for the treatment of uncomplicated *Plasmodium falciparum* malaria. *Int J Pharm Sci Rev Res* 2011; **3**(3): 221-4.

825. Mullick S, Das S, Guha SK, et al. Efficacy of chloroquine and sulphadoxine-pyrimethamine either alone or in combination before introduction of ACT as first-line therapy in uncomplicated *Plasmodium falciparum* malaria in Jalpaiguri District, West Bengal, India. *Trop Med Int Health* 2011; **16**(8): 929-35.

826. Song J, Socheat D, Tan B, et al. Randomized trials of artemisinin-piperaquine, dihydroartemisinin-piperaquine phosphate and artemether-lumefantrine for the treatment of multi-drug resistant falciparum malaria in Cambodia-Thailand border area. *Malar J* 2011; **10**: 231.

827. Toma H, Hatabu T, Vanisaveth V, et al. Efficacy of mefloquine treatment and genetic profiles in uncomplicated *Plasmodium falciparum* malaria in southern Lao PDR. *Southeast Asian J Trop Med Public Health* 2011; **42**(4): 759-63.

828. Anvikar AR, Sharma B, Shahi BH, et al. Artesunate-amodiaquine fixed dose combination for the treatment of *Plasmodium falciparum* malaria in India. *Malar J* 2012; **11**: 97.

829. Bouchaud O, Muhlberger N, Parola P, et al. Therapy of uncomplicated falciparum malaria in Europe: MALTHER - a prospective observational multicentre study. *Malar J* 2012; **11**: 212.

830. Carrasquilla G, Baron C, Monsell EM, et al. Randomized, prospective, three-arm study to confirm the auditory safety and efficacy of artemether-lumefantrine in Colombian patients with uncomplicated *Plasmodium falciparum* malaria. *Am J Trop Med Hyg* 2012; **86**(1): 75-83.

831. De la Hoz Restrepo F, Porras Ramirez A, Rico Mendoza A, Cordoba F, Rojas DP. Artesunate + amodiaquine versus artemether-lumefantrine for the treatment of uncomplicated *Plasmodium falciparum* malaria in the Colombian Pacific region: a noninferiority trial. *Rev Soc Bras Med Trop* 2012; **45**(6): 732-8.

832. Gargano N, Ubben D, Tommasini S, et al. Therapeutic efficacy and safety of dihydroartemisinin-piperaquine versus artesunate-mefloquine in uncomplicated *Plasmodium falciparum* malaria in India. *Malar J* 2012; **11**: 233.

833. Huang F, Tang L, Yang H, Zhou S, Sun X, Liu H. Therapeutic efficacy of artesunate in the treatment of uncomplicated *Plasmodium falciparum* malaria and anti-malarial, drug-resistance marker polymorphisms in populations near the China-Myanmar border. *Malar J* 2012; **11**: 278.

834. Kolaczinski K, Leslie T, Ali I, et al. Defining *Plasmodium falciparum* treatment in South West Asia: a randomized trial comparing artesunate or primaquine combined with chloroquine or SP. *PLoS One* 2012; **7**(1): e28957.

835. Ratsimbasoa A, Ravony H, Vonimpaisomihanta JA, et al. Compliance, safety, and effectiveness of fixed-dose artesunate-amodiaquine for presumptive treatment of non-severe malaria in the context of home management of malaria in Madagascar. *Am J Trop Med Hyg* 2012; **86**(2): 203-10.

836. Saha P, Guha SK, Das S, et al. Comparative efficacy of Artemisinin Combination Therapies (ACTs) in *P. falciparum* malaria and polymorphism of *PfATPase6*, *Pfcrt*, *Pfdhfr* and *Pfdhps* genes in tea gardens of Jalpaiguri district, India. *Antimicrob Agents Chemother* 2012; **56**(5): 2511-7.

837. Satimai W, Sudathip P, Vijaykadga S, et al. Artemisinin resistance containment project in Thailand. II: Responses to mefloquine-artesunate combination therapy among falciparum malaria patients in provinces bordering Cambodia. *Malar J* 2012; **11**: 300.

838. Valecha N, Mohanty S, Srivastava P, et al. Efficacy of artemether-lumefantrine in area of high malaria endemicity in India and its correlation with blood concentration of lumefantrine. *Am J Trop Med Hyg* 2012; **86**(3): 395-7.

839. Vijaykadga S, Alker AP, Satimai W, MacArthur JR, Meshnick SR, Wongsrichanalai C. Delayed *Plasmodium falciparum* clearance following artesunate-mefloquine combination therapy in Thailand, 1997-2007. *Malar J* 2012; **11**: 296.

840. Carrara VI, Lwin KM, Phyo AP, et al. Malaria burden and artemisinin resistance in the mobile and migrant population on the Thai-Myanmar border, 1999-2011: an observational study. *PLoS Med* 2013; **10**(3): e1001398.

841. Das D, Tripura R, Phyo AP, et al. Effect of high-dose or split-dose artesunate on parasite clearance in artemisinin-resistant falciparum malaria. *Clin Infect Dis* 2013; **56**(5): e48-58.

842. Das S, Chakraborty SP, Hati AK, Roy S. Association between Prevalence of Chloroquine Resistance and Unusual Mutation in *pfmdr*-I and *pfcrt* Genes in India. *Am J Trop Med Hyg* 2013; **88**(5): 828-34.

843. Das S, Chakraborty SP, Hati A, Roy S. Malaria treatment failure with novel mutation in the *Plasmodium falciparum* dihydrofolate reductase (*pfdhfr*) gene in Kolkata, West Bengal, India. *Int J Antimicrob Agents* 2013; **41**(5): 447-51.

844. Gadalla NB, Abdallah TM, Atwal S, Sutherland CJ, Adam I. Selection of *pfdhfr/pfdhps* alleles and declining artesunate/sulphadoxine-pyrimethamine efficacy against *Plasmodium falciparum* eight years after deployment in eastern Sudan. *Malar J* 2013; **12**: 255.

845. Leang R, Ros S, Duong S, et al. Therapeutic efficacy of fixed dose artesunate-mefloquine for the treatment of acute, uncomplicated *Plasmodium falciparum* malaria in Kampong Speu, Cambodia. *Malar J* 2013; **12**: 343.

846. Pareek A, Chandurkar N, Srivastav V, et al. Comparative evaluation of efficacy and safety of artesunate-lumefantrine vs. artemether-lumefantrine fixed-dose combination in the treatment of uncomplicated *Plasmodium falciparum* malaria. *Trop Med Int Health* 2013; **18**(5): 578-87.

847. Saha P, Naskar A, Ganguly S, et al. Therapeutic efficacy of artemisinin combination therapies and prevalence of S769N mutation in *PfATPase6* gene of *Plasmodium falciparum* in Kolkata, India. *Asian Pac J Trop Med* 2013; **6**(6): 443-8.

848. Sutanto I, Suprijanto S, Kosasih A, et al. The effect of primaquine on gametocyte development and clearance in the treatment of uncomplicated falciparum malaria with dihydroartemisinin-piperaquine in South sumatra, Western indonesia: an open-label, randomized, controlled trial. *Clin Infect Dis* 2013; **56**(5): 685-93.

849. Mejia Torres RE, Banegas EI, Mendoza M, et al. Efficacy of chloroquine for the treatment of uncomplicated *Plasmodium falciparum* malaria in Honduras. *Am J Trop Med Hyg* 2013; **88**(5): 850-4.

850. Ashley EA, Dhorda M, Fairhurst RM, et al. Spread of artemisinin resistance in *Plasmodium falciparum* malaria. *N Engl J Med* 2014; **371**(5): 411-23.

851. Jullien V, Valecha N, Srivastava B, Sharma B, Kiechel JR. Population pharmacokinetics of mefloquine, administered as a fixed-dose combination of artesunate-mefloquine in Indian patients for the treatment of acute uncomplicated *Plasmodium falciparum* malaria. *Malar J* 2014; **13**: 187.

852. Moore BR, Benjamin JM, Salman S, et al. Effect of coadministered fat on the tolerability, safety, and pharmacokinetic properties of dihydroartemisinin-piperaquine in Papua New Guinean children with uncomplicated malaria. *Antimicrob Agents Chemother* 2014; **58**(10): 5784-94.

853. Thriemer K, Hong NV, Rosanas-Urgell A, et al. Delayed parasite clearance after treatment with dihydroartemisinin-piperaquine in *Plasmodium falciparum* malaria patients in central Vietnam. *Antimicrob Agents Chemother* 2014; **58**(12): 7049-55.

854. Adeel AA, Saeed NA, Aljasari A, et al. High efficacy of two artemisinin-based combinations: artesunate + sulfadoxine-pyrimethamine and artemether-lumefantrine for falciparum malaria in Yemen. *Malar J* 2015; **14**: 449.

855. Basu A, Saha S, Guha SK. Comparative Study of Effectiveness and Resistance Profile of Chloroquine and Sulfadoxine-Pyrimethamine in Uncomplicated *Plasmodium falciparum* Malaria in Kolkata. *J Assoc Physicians India* 2015; **63**(5): 32-7.

856. Getnet G, Fola AA, Alemu A, Getie S, Fuehrer HP, Noedl H. Therapeutic efficacy of artemether-lumefantrine for the treatment of uncomplicated *Plasmodium falciparum* malaria in Enfranze, north-west Ethiopia. *Malar J* 2015; **14**: 258.

857. Huang F, Takala-Harrison S, Jacob CG, et al. A Single Mutation in K13 Predominates in Southern China and Is Associated With Delayed Clearance of *Plasmodium falciparum* Following Artemisinin Treatment. *J Infect Dis* 2015; **212**(10): 1629-35.

858. Leang R, Taylor WR, Bouth DM, et al. Evidence of *Plasmodium falciparum* Malaria Multidrug Resistance to Artemisinin and Piperaquine in Western Cambodia: Dihydroartemisinin-Piperaquine Open-Label Multicenter Clinical Assessment. *Antimicrob Agents Chemother* 2015; **59**(8): 4719-26.

859. Liu H, Yang H, Tang L, et al. *In vivo* monitoring of dihydroartemisinin-piperaquine sensitivity in *Plasmodium falciparum* along the China-Myanmar border of Yunnan Province, China from 2007 to 2013. *Malar J* 2015; **14**(47).
860. Chatterjee M, Ganguly S, Saha P, et al. No Polymorphism in Plasmodium falciparum K13 Propeller Gene in Clinical Isolates from Kolkata, India. *J Pathogen* 2015; **2015**: 374354.
861. Toure OA, Rulisa S, Anvikar AR, et al. Efficacy and safety of fixed dose combination of arterolane maleate and piperaquine phosphate dispersible tablets in paediatric patients with acute uncomplicated *Plasmodium falciparum* malaria: a phase II, multicentric, open-label study. *Malar J* 2015; **14**: 469.
862. Gupta R, Mishra N, Kumar A, et al. Monitoring artemisinin resistance in *Plasmodium falciparum*: comparison of parasite clearance time by microscopy and real-time PCR and evaluation of mutations in *Pfatpase6* gene in Odisha state of India. *Parasitol Res* 2015; **114**(9): 3487-96.
863. Wang SQ, Wang GZ, Li YC, et al. Sensitivity of *Plasmodium falciparum* to antimalarial drugs in Hainan Island, China. *Korean J Parasitol* 2015; **53**(1): 35-41.
864. Warsame M, Hassan AM, Barrette A, et al. Treatment of uncomplicated malaria with artesunate plus sulfadoxine-pyrimethamine is failing in Somalia: evidence from therapeutic efficacy studies and *Pfdhfr* and *Pfdhps* mutant alleles. *Trop Med Int Health* 2015; **20**(4): 510-7.
865. Wang Y, Yang Z, Yuan L, et al. Clinical Efficacy of Dihydroartemisinin-Piperaquine for the Treatment of Uncomplicated *Plasmodium falciparum* Malaria at the China-Myanmar Border. *Am J Trop Med Hyg* 2015; **93**(3): 577-83.
866. Phyo AP, Jittamala P, Nosten FH, et al. Antimalarial activity of artefenomel (OZ439), a novel synthetic antimalarial endoperoxide, in patients with *Plasmodium falciparum* and *Plasmodium vivax* malaria: an open-label phase 2 trial. *Lancet Infect Dis* 2016; **16**(1): 61-9.
867. Moore BR, Benjamin JM, Auyeung SO, et al. Safety, tolerability and pharmacokinetic properties of coadministered azithromycin and piperaquine in pregnant Papua New Guinean women. *Br J Clin Pharmacol* 2016; **82**(1): 199-212.
868. Bharti PK, Shukla MM, Ringwald P, et al. Therapeutic efficacy of artemether-lumefantrine for the treatment of uncomplicated *Plasmodium falciparum* malaria from three highly malarious states in India. *Malar J* 2016; **15**(1): 498.
869. Lo E, Nguyen J, Oo W, et al. Examining *Plasmodium falciparum* and *P. vivax* clearance subsequent to antimalarial drug treatment in the Myanmar-China border area based on quantitative real-time polymerase chain reaction. *BMC Infect Dis* 2016; **16**: 154.
870. Ley B, Alam MS, Thriemer K, et al. G6PD Deficiency and Antimalarial Efficacy for Uncomplicated Malaria in Bangladesh: A Prospective Observational Study. *PLoS One* 2016; **11**(4): e0154015.
871. White NJ, Duong TT, Uthaisin C, et al. Antimalarial Activity of KAF156 in Falciparum and Vivax Malaria. *N Engl J Med* 2016; **375**(12): 1152-60.
872. Nega D, Assefa A, Mohamed H, et al. Therapeutic Efficacy of Artemether-Lumefantrine (Coartem(R)) in Treating Uncomplicated *P. falciparum* Malaria in Metehara, Eastern Ethiopia: Regulatory Clinical Study. *PLoS One* 2016; **11**(4): e0154618.
873. Phyo AP, Ashley EA, Anderson TJC, et al. Declining Efficacy of Artemisinin Combination Therapy Against *P. Falciparum* Malaria on the Thai-Myanmar Border (2003-2013): The Role of Parasite Genetic Factors. *Clin Infect Dis* 2016; **63**(6): 784-91.
874. Vreden SG, Bansie RD, Jitan JK, Adhin MR. Assessing parasite clearance during uncomplicated *Plasmodium falciparum* infection treated with artesunate monotherapy in Suriname. *Infect Drug Resist* 2016; **9**: 261-7.
875. Irawati N, Kurniawan B, Suwandi JF, Hasmiwati, Tjong DH, Kanedi M. Determination of the Falciparum Malaria Resistance to Artemisinin-based Combination Therapies in Pesawaran, Lampung, Indonesia. *Asian J Epi* 2017; **10**(1): 19-25.
876. Macintyre F, Adoke Y, Tiono AB, et al. A randomised, double-blind clinical phase II trial of the efficacy, safety, tolerability and pharmacokinetics of a single dose combination treatment with artefenomel and piperaquine in adults and children with uncomplicated *Plasmodium falciparum* malaria. *BMC Med* 2017; **15**(1): 181.
877. Thanh NV, Thuy-Nhien N, Tuyen NT, et al. Rapid decline in the susceptibility of *Plasmodium falciparum* to dihydroartemisinin-piperaquine in the south of Vietnam. *Malar J* 2017; **16**(1): 27.
878. Valera CV, Shute GT. Preliminary studies on the response of *Plasmodium falciparum* to chloroquine in the Philippines, with the *in vitro* technique. *Bull World Health Organ* 1976; **53**(4): 391-8.
879. Mohammed AO, Tewolde S, Estifanos D, Tekeste Y, Osman MH. Therapeutic efficacy of artesunate - amiodaquine for treating uncomplicated falciparum malaria at Ghindae Zonal Referral Hospital, Eritrea. *Acta Trop* 2018; **177**: 94-6.
880. Bayoumi RA, Dar FK, Tanira MO, et al. Effect of previous chloroquine intake on *in vivo P. falciparum* drug sensitivity. *East Afr Med J* 1997; **74**(5): 278-82.
881. Grynberg S, Lachish T, Kopel E, Meltzer E, Schwartz E. Artemether-lumefantrine compared to atovaquone-proguanil as a treatment for uncomplicated *Plasmodium falciparum* malaria in travelers. *Am J Trop Med Hyg* 2015; **92**(1): 13-7.
882. Ebisawa I, Ohara H. A combination of sulfamonomethoxine and pyrimethamine versus other drugs for the treatment of malaria. *Jpn J Exp Med* 1986; **56**(5): 213-9.
883. Hasugian AR, Purba HL, Kenangalem E, et al. Dihydroartemisinin-piperaquine versus artesunate-amodiaquine: superior efficacy and posttreatment prophylaxis against multidrug-resistant *Plasmodium falciparum* and *Plasmodium vivax* malaria. *Clin Infect Dis* 2007; **44**(8): 1067-74.
884. Tjitra E, Hasugian AR, Siswantoro H, et al. Efficacy and safety of artemisinin-naphthoquine versus dihydroartemisinin-piperaquine in adult patients with uncomplicated malaria: a multi-centre study in Indonesia. *Malar J* 2012; **11**: 153.

885. Pedro RS, Guaraldo L, Campos DP, Costa AP, Daniel-Ribeiro CT, Brasil P. *Plasmodium vivax* malaria relapses at a travel medicine centre in Rio de Janeiro, a non-endemic area in Brazil. *Malar J* 2012; **11**: 245.

886. Senn N, Rarau P, Manong D, et al. Effectiveness of artemether/lumefantrine for the treatment of uncomplicated *Plasmodium vivax* and *P. falciparum* malaria in young children in Papua New Guinea. *Clin Infect Dis* 2013; **56**(10): 1413-20.

887. Toure OA, Valecha N, Tshefu AK, et al. A Phase 3, Double-Blind, Randomized Study of Arterolane Maleate-Piperaquine Phosphate vs Artemether-Lumefantrine for Falciparum Malaria in Adolescent and Adult Patients in Asia and Africa. *Clin Infect Dis* 2016; **62**(8): 964-71.

888. Laman M, Benjamin JM, Moore BR, et al. Artemether-lumefantrine versus artemisinin-naphthoquine in Papua New Guinean children with uncomplicated malaria: a six months post-treatment follow-up study. *Malar J* 2015; **14**: 121.

889. White NJ, Pukrittayakamee S, Phyo AP, et al. Spiroindolone KAE609 for falciparum and vivax malaria. *N Engl J Med* 2014; **371**(5): 403-10.

890. Rueangweerayut R, Phyo AP, Uthaisin C, et al. Pyronaridine-artesunate versus mefloquine plus artesunate for malaria. *N Engl J Med* 2012; **366**(14): 1298-309.

891. Ashley EA, Lwin KM, McGready R, et al. An open label randomized comparison of mefloquine-artesunate as separate tablets vs. a new co-formulated combination for the treatment of uncomplicated multidrug-resistant falciparum malaria in Thailand. *Trop Med Int Health* 2006; **11**(11): 1653-60.

892. Krudsood S, Silachamroon U, Wilairatana P, et al. A randomized clinical trial of combinations of artesunate and azithromycin for treatment of uncomplicated *Plasmodium falciparum* malaria in Thailand. *Southeast Asian J Trop Med Public Health* 2000; **31**(4): 801-7.

893. McGready R, Cho T, Hkirijaroen L, et al. Quinine and mefloquine in the treatment of multidrug-resistant *Plasmodium falciparum* malaria in pregnancy. *Ann Trop Med Parasitol* 1998; **92**(6): 643-53.

894. Parkinson D, Balmer V, Ajdukiewicz A, Korinohowa A, Kere N. The effectiveness of halofantrine for the treatment of acute malaria in adults in the Solomon Islands. *Parasit Today* 1989; **15**(Suppl): 27-35.

895. McGready R, Brockman A, Cho T, et al. Randomized comparison of mefloquine-artesunate versus quinine in the treatment of multidrug-resistant falciparum malaria in pregnancy. *Trans R Soc Trop Med Hyg* 2000; **94**(6): 689-93.

896. McGready R, Cho T, Samuel, et al. Randomized comparison of quinine-clindamycin versus artesunate in the treatment of falciparum malaria in pregnancy. *Trans R Soc Trop Med Hyg* 2001; **95**(6): 651-6.

897. McGready R, Keo NK, Villegas L, White NJ, Looareesuwan S, Nosten F. Artesunate-atovaquone-proguanil rescue treatment of multidrug-resistant *Plasmodium falciparum* malaria in pregnancy: a preliminary report. *Trans R Soc Trop Med Hyg* 2003; **97**(5): 592-4.

898. McGready R, Ashley EA, Moo E, et al. A randomized comparison of artesunate-atovaquone-proguanil versus quinine in treatment for uncomplicated falciparum malaria during pregnancy. *J Infect Dis* 2005; **192**(5): 846-53.

899. McGready R, Tan SO, Ashley EA, et al. A randomised controlled trial of artemether-lumefantrine versus artesunate for uncomplicated *Plasmodium falciparum* treatment in pregnancy. *PLoS Med* 2008; **5**(12): e253.

900. Rijken MJ, McGready R, Boel ME, et al. Dihydroartemisinin-piperaquine rescue treatment of multidrug-resistant *Plasmodium falciparum* malaria in pregnancy: a preliminary report. *Am J Trop Med Hyg* 2008; **78**(4): 543-5.

901. Perez MA, Cortes LJ, Guerra AP, Knudson A, Usta C, Nicholls RS. [Efficacy of the amodiaquine+sulfadoxine-pyrimethamine combination and of chloroquine for the treatment of malaria in Cordoba, Colombia, 2006]. *Biomedica* 2008; **28**(1): 148-59.

902. McGready R, Cho T, Keo NK, et al. Artemisinin antimalarials in pregnancy: a prospective treatment study of 539 episodes of multidrug-resistant *Plasmodium falciparum*. *Clin Infect Dis* 2001; **33**(12): 2009-16.

903. Shah NK, Schapira A, Juliano JJ, et al. Nonrandomized controlled trial of artesunate plus sulfadoxine-pyrimethamine with or without primaquine for preventing posttreatment circulation of *Plasmodium falciparum* gametocytes. *Antimicrob Agents Chemother* 2013; **57**(7): 2948-54.

904. Duparc S, Borghini-Fuhrer I, Craft CJ, et al. Safety and efficacy of pyronaridine-artesunate in uncomplicated acute malaria: an integrated analysis of individual patient data from six randomized clinical trials. *Malar J* 2013; **12**: 70.

905. Laochan N, Zaloumis SG, Imwong M, et al. Intervals to *Plasmodium falciparum* recurrence after anti-malarial treatment in pregnancy: a longitudinal prospective cohort. *Malar J* 2015; **14**: 221.

906. Tshefu AK, Gaye O, Kayentao K, et al. Efficacy and safety of a fixed-dose oral combination of pyronaridine-artesunate compared with artemether-lumefantrine in children and adults with uncomplicated *Plasmodium falciparum* malaria: a randomised non-inferiority trial. *Lancet* 2010; **375**(9724): 1457-67.

907. Valecha N, Looareesuwan S, Martensson A, et al. Arterolane, a new synthetic trioxolane for treatment of uncomplicated *Plasmodium falciparum* malaria: a phase II, multicenter, randomized, dose-finding clinical trial. *Clin Infect Dis* 2010; **51**(6): 684-91.

908. Burns M, Baker J, Auliff AM, Gatton ML, Edstein MD, Cheng Q. Efficacy of sulfadoxine-pyrimethamine in the treatment of uncomplicated *Plasmodium falciparum* malaria in East Timor. *Am J Trop Med Hyg* 2006; **74**(3): 361-6.

909. Rahman MR, Paul DC, Rashid M, et al. A randomized controlled trial on the efficacy of alternative treatment regimens for uncomplicated falciparum malaria in a multidrug-resistant falciparum area of Bangladesh--narrowing the options for the National Malaria Control Programme? *Trans R Soc Trop Med Hyg* 2001; **95**(6): 661-7.

910. Hatz C, Soto J, Nothdurft HD, et al. Treatment of acute uncomplicated falciparum malaria with artemether-lumefantrine in nonimmune populations: a safety, efficacy, and pharmacokinetic study. *Am J Trop Med Hyg* 2008; **78**(2): 241-7.

911. Minodier P, Noel G, Tall M, et al. Mefloquine for uncomplicated *Plasmodium falciparum* malaria in children. *Pediatr Infect Dis J* 2011; **30**(10): 883-6.

912. Kayentao K, Doumbo OK, Penali LK, et al. Pyronaridine-artesunate granules versus artemether-lumefantrine crushed tablets in children with *Plasmodium falciparum* malaria: a randomized controlled trial. *Malar J* 2012; **11**: 364.

913. Toure OA, Mwapasa V, Sagara I, et al. Assessment of Efficacy and Safety of Arterolane Maleate-Piperaquine Phosphate Dispersible Tablets in Comparison With Artemether-Lumefantrine Dispersible Tablets in Pediatric Patients With Acute Uncomplicated *Plasmodium falciparum* Malaria: A Phase 3, Randomized, Multicenter Trial in India and Africa. *Clin Infect Dis* 2017; **65**(10): 1711-20.

914. Taylor WR, Widjaja H, Richie TL, et al. Chloroquine/doxycycline combination versus chloroquine alone, and doxycycline alone for the treatment of *Plasmodium falciparum* and *Plasmodium vivax* malaria in northeastern Irian Jaya, Indonesia. *Am J Trop Med Hyg* 2001; **64**(5-6): 223-8.

915. Raichowdhuri AN, Sinha S, Bhuyya, Gajanana A, Baishya KK. Chloroquine resistance of *Plasmodium falciparum* in Sonapur PHC Area (Assam) detected by micro *in vitro* and *in vivo* tests. *Indian J Malariol* 1984; **21**(1): 25-30.

916. Sarma PS, Mandal AK, Khamis HJ. Allopurinol as an additive to quinine in the treatment of acute complicated falciparum malaria. *Am J Trop Med Hyg* 1998; **58**(4): 454-7.

917. Vanijanonta S, Chantra A, Phophak N, Chindanond D, Clemens R, Pukrittayakamee S. Therapeutic effects of chloroquine in combination with quinine in uncomplicated falciparum malaria. *Ann Trop Med Parasitol* 1996; **90**(3): 269-75.

918. Darlow B, Vrbova H, Gibney S, Jolley D, Stace J, Alpers M. Sulfadoxine-pyrimethamine for the treatment of acute malaria in children of Papua New Guinea. II. *Plasmodium vivax*. *Am J Trop Med Hyg* 1982; **31**(1): 10-3.

919. de Souza JM. A phase I clinical trial of mefloquine in Brazilian male subjects. *Bull World Health Organ* 1983; **61**(5): 809-14.

920. de Souza JM, Sheth UK, de Oliveira RM, Gomes AT, Cavalcante EQ. A phase I clinical trial of Fansimef (mefloquine plus sulfadoxine-pyrimethamine) in Brazilian male subjects. *Bull World Health Organ* 1985; **63**(3): 611-5.
